# A randomised, double-blind clinical phase II trial of the efficacy, safety, tolerability and pharmacokinetics of a single dose combination treatment with artefenomel and piperaquine in adults and children with uncomplicated *Plasmodium falciparum* malaria

**DOI:** 10.1186/s12916-017-0940-3

**Published:** 2017-10-09

**Authors:** Fiona Macintyre, Yeka Adoke, Alfred B. Tiono, Tran Thanh Duong, Ghyslain Mombo-Ngoma, Marielle Bouyou-Akotet, Halidou Tinto, Quique Bassat, Saadou Issifou, Marc Adamy, Helen Demarest, Stephan Duparc, Didier Leroy, Bart E. Laurijssens, Sophie Biguenet, Afizi Kibuuka, Antoinette Kitoto Tshefu, Melnick Smith, Chanelle Foster, Illse Leipoldt, Peter G. Kremsner, Bui Quang Phuc, Alphonse Ouedraogo, Michael Ramharter, on behalf of the OZ-Piperaquine Study Group, Nguyen Van Hong, Christelle Offouga Mbouoronde, Joy Luzingu Kinko, Joseph Atibu Losoma, Rella Zoleko Manego, Mirjam Groger, Anna Klicpera, Johannes Mischlinger, Aissata Barry, San Maurice Ouattara, Sam Coulibaly, Kabore Moïse, Olivier Sombié, Joel Dofinissery Bognini, Antonio Sitoe, Rosauro Varo, Myriam El Gaaloul, Nathalie Gobeau, Eugene H Cox, John T Maringwa, Alfredo Mayor, Gloria Matambisso

**Affiliations:** 1 Medicines for Malaria Venture, Geneva, Switzerland; 2 Infectious Diseases Research Collaboration, Tororo Hospital, Tororo, Uganda; 3 Centre National de Recherche et de Formation sur le Paludisme, Ouagadougou, Burkina Faso; 4 National Institute of Malariology, Parasitology and Entomology, Hanoi, Vietnam; 5 Centre de Recherches Médicales de Lambaréné, Lambaréné, Gabon; 6 Institut für Tropenmedizin, Universität Tübingen, Tübingen, Germany; 7 Universite des Sciences de la Sante Gabon, Département de Parasitology, Malaria Clinical and Operational Research Unit, Melen Hospital, Libreville, Gabon; 8 Institut de Recherche en Sciences de la Santé – Unité de Recherche Clinique de Nanoro, Ouagadougou, Burkina Faso; 9 ISGlobal, Barcelona Ctr. Int. Health Res. (CRESIB), Hospital Clínic - Universitat de Barcelona, Barcelona, Spain; 10 Centro de Investigação em Saúde de Manhiça (CISM), Maputo, Mozambique; 11 ICREA, Pg. Lluís Companys 23, 08010 Barcelona, Spain; 12 Pediatric Infectious Diseases Unit, Pediatrics Department, Hospital Sant Joan de Déu (University of Barcelona), Barcelona, Spain; 13 Universidad Europea de Madrid, Madrid, Spain; 14 Centre de Recherche sur le Paludisme Associé à la Grossesse et l’Enfance, Faculte Des Sciences De La Sante, Cotonou, Benin; 15 BEL Pharm Consulting, Chambonas, France; 16 Centre de Recherche du Centre Hospitalier de Mont Amba, Kinshasa School of Public Health, University of Kinshasa, Kinshasa, Democratic Republic of the Congo; 17 QuintilesIMS, Department: Biostatistics, Bloemfontein, South Africa; 18 Department of Medicine I, Division of Infectious Diseases, Medical University of Vienna, Vienna, Austria; 19 Bernhard Nocht Hospital for Tropical Diseases, Bernhard Nocht Institute for Tropical Medicine and University Medical Center Hamburg-Eppendorf, Hamburg, Germany

**Keywords:** Artefenomel, OZ439, Piperaquine, Single dose combination treatment, Pharmacokinetics, Dose–response, modelling and simulation, Phase II B, Uncomplicated *Plasmodium falciparum* malaria, Children

## Abstract

**Background:**

The clinical development of a single encounter treatment for uncomplicated malaria has the potential to significantly improve the effectiveness of antimalarials. Exploratory data suggested that the combination of artefenomel and piperaquine phosphate (PQP) has the potential to achieve satisfactory cure rates as a single dose therapy. The primary objective of the study was to determine whether a single dose of artefenomel (800 mg) plus PQP in ascending doses is an efficacious treatment for uncomplicated *Plasmodium falciparum* malaria in the 'target' population of children ≤ 5 years of age in Africa as well as Asian patients of all ages.

**Methods:**

Patients in six African countries and in Vietnam were randomised to treatment with follow-up for 42–63 days. Efficacy, tolerability, safety and pharmacokinetics were assessed. Additional key objectives were to characterise the exposure–response relationship for polymerase chain reaction (PCR)-adjusted adequate clinical and parasitological response at day 28 post-dose (ACPR28) and to further investigate Kelch13 mutations. Patients in Africa (*n* = 355) and Vietnam (*n* = 82) were included, with 85% of the total population being children < 5 years of age.

**Results:**

ACPR28 in the per protocol population (95% confidence interval) was 70.8% (61.13–79.19), 68.4% (59.13–76.66) and 78.6% (70.09–85.67) for doses of 800 mg artefenomel with 640 mg, 960 mg and 1440 mg of PQP respectively. ACPR28 was lower in Vietnamese than in African patients (66.2%; 54.55–76.62 and 74.5%; 68.81–79.68) respectively. Within the African population, efficacy was lowest in the youngest age group of ≥ 0.5 to ≤ 2 years, 52.7% (38.80–66.35). Initial parasite clearance was twice as long in Vietnam than in Africa. Within Vietnam, the frequency of the Kelch13 mutation was 70.1% and was clearly associated with parasite clearance half-life (PCt1/2). The most significant tolerability finding was vomiting (28.8%).

**Conclusions:**

In this first clinical trial evaluating a single encounter antimalarial therapy, none of the treatment arms reached the target efficacy of > 95% PCR-adjusted ACPR at day 28. Achieving very high efficacy following single dose treatment is challenging, since > 95% of the population must have sufficient concentrations to achieve cure across a range of parasite sensitivities and baseline parasitaemia levels. While challenging, the development of tools suitable for deployment as single encounter curative treatments for adults and children in Africa and to support elimination strategies remains a key development goal.

**Trial registration:**

ClinicalTrials.gov, NCT02083380. Registered on 7 March 2014.

**Electronic supplementary material:**

The online version of this article (doi:10.1186/s12916-017-0940-3) contains supplementary material, which is available to authorized users.

## Background

Since 2000 the incidence of malaria has fallen by 41% and mortality rates have declined by 62% globally, due to increased deployment of new interventions including artemisinin-based combination treatments and insecticide treated bed nets (WHO 2016) [1]. However, despite these gains, in 2015 there were 212 million new cases and an estimated 429,000 malaria-related deaths, with Africa continuing to bear the heaviest burden, accounting for approximately 9 in 10 malaria cases and deaths, the vast majority of which were in young children [1]. The high death rate in young children is believed to be linked to low acquired immunity, coupled with greater vulnerability to the infection. Since lower immunity may mean that young children require higher drug exposures than older patients to achieve cure, it is important to tailor dose selection to this population. Patients in Asia may also have lower immunity due to low endemicity.

Numerous studies have suggested that in 'real-life' community settings, poor to moderate adherence to current standard 3-day treatment regimens is common, and this could impact morbidity and mortality as well as drive the development of resistance, although definitive data are difficult to obtain [2–4]. Availability of a highly efficacious 'single encounter treatment' would be expected to improve effectiveness of malaria treatment and delay selection of resistant parasites. An effective cure that can be administered as a single treatment, directly observed if required, would also provide an important tool to support malaria elimination efforts [5, 6]. Medicines for Malaria Venture (MMV) and its partners have undertaken to develop single dose treatments for malaria [7].

Exploratory clinical data suggested that artefenomel (OZ439) plus piperaquine phosphate (PQP) in combination could be efficacious as a single encounter cure. Artefenomel, a novel synthetic trioxolane, contains a similar peroxidic pharmacophore to artemisinins and has demonstrated rapid parasite clearance in patients, with a median parasite reduction ratio at 24 h (log10) post-treatment (PRR24) for *Plasmodium falciparum* ranging from 0.9 to 1.88 [8]. PQP is a long-acting antimalarial currently marketed in a fixed dose combination with dihydroartemisinin (DHA), administered once daily for 3 days.

We report the results of the first clinical efficacy study of the combination of artefenomel and PQP in a design which allowed rapid progression from adult African patient to children ≤ 5 years of age and Asian patients of all ages to ensure that dose finding for phase III is carried out in populations most likely to require the highest exposures to achieve cure. The study also employed interim futility analyses in order to drop doses with a low probability of success early.

## Methods

### Study objectives

The primary objective of the study was to determine whether a single dose combination of artefenomel plus PQP is an efficacious treatment for uncomplicated *P. falciparum* malaria.

Secondary and exploratory objectives included determination of the incidence of recurrence, recrudescence and new infection, estimation of parasite clearance kinetics and exploration of the relationship between Kelch13 genotype and parasite clearance half-life (PCt1/2) in Asian patients.

An additional key exploratory objective was to characterise the dose/exposure–response relationship for the combination for the primary efficacy endpoint across the patient population and to identify significant covariates influencing efficacy. Safety, tolerability and pharmacokinetics (PK) were also assessed. Details of the study objectives, design and endpoints are summarised in Additional file 1: S1 Study protocol, Section 1 Study synopsis and in more detail in Sections 4, 5.1 and 5.10 respectively.

The study was conducted at nine study sites across six African countries, Benin (Cotonou), Burkina Faso (Nanoro, Banfora and Niangoloko) [9, 10], Democratic Republic of Congo (Kinshasa) [11], Gabon (Libreville, Lambaréné) [12], Mozambique (Manhiça) [13] and Uganda (Tororo) [14], and four sites in Vietnam (Quang tri, Gia Lai, Khanh Hoa, Binh Phuoc) [15]. Malaria prevalence is hyperendemic to holoendemic, and transmission is perennial in all sites with seasonal variation. Drug resistance of *P. falciparum* against chloroquine and sulfadoxine-pyrimethamine is widespread at all African sites, and evidence of artemisinin resistance was confirmed at the Vietnamese sites [16].

### Study design, participants and interventions

This was a randomised, double-blind, single dose study to investigate the efficacy, safety, tolerability and PK of artefenomel 800 mg in loose combination with three doses of PQP (640, 960, 1440 mg) in male and female patients aged ≥ 6 months to < 70 years (body weight ≥ 5 kg to ≤ 90 kg) with uncomplicated *P. falciparum* malaria. The artefenomel dose of 800 mg was expected to deliver close to the maximum well-tolerated exposure, and PQP doses were selected to span a range of adequate clinical and parasitological response at day 28 (ACPR28) values, with the highest dose estimated to give a mean maximum placebo corrected change from baseline QTcF of 18 ms [17].

Patients presenting with microscopically confirmed *P. falciparum* mono-infection in the range 1000 to 100,000 asexual parasites/μL of blood, and with fever (axillary temperature ≥ 37.5 °C) or history of fever in the preceding 24 h, were included following their submittal of written informed consent, and all eligible patients were randomised via an Interactive Web Response System (IWRS) in a ratio of 1:1:1 to one of the three treatment arms (see supplementary material). Important exclusion criteria were the presence of severe malaria (according to the WHO definition [18]), haemoglobin below 8 g/dL, exclusions relating to cardiac and hepatic safety and prior antimalarial treatment within specified time frames. Full inclusion/exclusion criteria are given in Additional file 1: S1 Study protocol, Sections 1 Study synopsis and 6 Selection of patients.

**Fig. 1:**
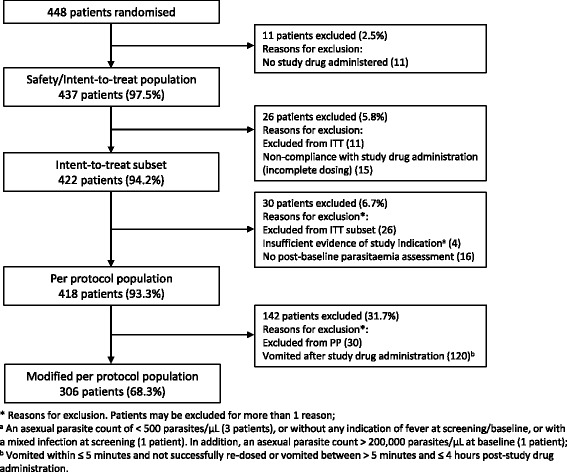
Analysis sets and exclusions

The study was initiated in patients aged > 15 years, and following review of safety data by an Independent Safety Monitoring Board (ISMB), sequentially younger patients were recruited in a step-down procedure described in Additional file 1: S1 Study protocol, Section 6.5.2 and illustrated in Figure 1 (S1 Study protocol), Step-down procedure. The aim was to recruit a population predominantly of African children ≤ 5 years of age and also to include Asian patients (the most important target populations). Patient recruitment and follow-up were conducted between July 2014 and August 2015.

Fasted patients ≥ 35 kg received artefenomel 800 mg in loose combination with PQP doses of 640, 960 or 1440 mg at day 0. Patients who weighed < 35 kg received body weight-adjusted doses [19] within weight bands predicted to achieve similar exposure ranges to patients ≥ 35 kg. The dose for a given weight band was adjusted by scaling clearance allometrically, using the relationship CL = (body weight/70)^0.75^. Artefenomel was administered as a suspension formulation containing α-tocopherol polyethylene glycol (TPGS). PQP was included in the suspension (for patients < 24 kg) or was administered as separate tablets (patients ≥ 24 kg), with blinding maintained by administering matching placebo tablets. For the lowest weight band the dosing volume was 75 mL (plus 2× 15 mL rinses). Patients who vomited within 5 min of start of dosing were re-dosed once. Details of study drug treatments and administration are given in Additional file 1: S1 Study protocol, Section 10 Treatment.

Following drug administration, patients were followed for 42 days or 63 days at some centres (patients were consented separately for days > 42 to 63). Patients remained in the clinical unit for a minimum of 48 (African patients > 5 years old) or 72 h (African patients ≤ 5 years old and all Asian patients) and were discharged provided parasite and fever clearance had been achieved. Patients returned for assessment on days 3, 5, 7, 10, 14, 21, 28, 42 and 63 at selected centres. Blood films (thick and thin) were prepared and axillary temperatures were measured at screening/pre-dose, 6, 12, 18, 24, 30, 36, 48, 72 h and days 5, 7, 10, 14, 21, 28, 42 and 63. Assessments for safety included haematology, clinical chemistry, urinalysis and a triplicate 12-lead electrocardiogram (ECG). Clinical assessments were taken according to the schedule presented in detail in Additional file 1: S1 Study protocol, Section 2 Schedule of assessments.

For screening, thick blood films were stained with 10% Giemsa for 10 min, and thick and thin films for baseline to follow-up were stained with 2% Giemsa for 30 min. Expert microscopists determined parasite densities and examined thick blood films for parasites and thin blood films for non-*falciparum* infections. A second microscopist, blinded to initial readings, re-read all slides, and a third resolved discrepant readings. A slide was considered negative in the absence of asexual parasites per 1000 counted leukocytes using a 100× magnification oil immersion objective. Parasite density was calculated as follows: (number of counted parasites/counted leukocytes) × most recent absolute leukocyte count per microliter. Details of additional methods are given in the supplementary material.

### Analysis populations

The intention to treat (ITT) and safety analysis sets were identical and included all patients who provided informed consent, received the study drug (entire or partial dose) and had a confirmed positive blood film for *P. falciparum* asexual parasitaemia at inclusion. The ITT subset defined for the Kaplan–Meier (KM) estimates of recurrence, recrudescence and new infection rate included only those who consumed the entire dose.

The per protocol (PP) set was the primary analysis set and included all patients comprising the ITT set who consumed the entire dose and were without major protocol deviations. The modified PP analysis set in addition excluded patients who vomited between > 5 min and ≤ 4 h after start of drug administration. Patients could be excluded from a population for more than one reason (see Fig. 1). The PK population included all patients in the ITT set with at least one evaluable PK sample after treatment administration.

### Ethical considerations

The study (MMV_OZ439_13_003) conformed to the Declaration of Helsinki and Standard Operating Procedures that meet current regulatory requirements and guidelines established by the International Conference on Harmonization for Good Clinical Practice in Clinical Studies. It was approved by the relevant Independent Ethics Committees (IECs), national Institutional Review Boards and, where relevant, local regulatory authorities at each of the participating sites (for more details see the supplementary material). The study protocol was registered and the study results are reported on ClinicalTrials.gov (NCT02083380).

### Endpoints

The primary efficacy endpoint was polymerase chain reaction (PCR)-adjusted ACPR28 in the PP analysis set. We also report the following secondary and exploratory endpoints: PCR-adjusted ACPR at day 42 (ACPR42) and day 63 (ACPR63) and crude ACPR at days 28, 42 and 63 for the ITT and PP analysis sets and Kaplan–Meier incidence rate of recrudescence over 63 days (ITT subset); in the PP analysis set, percentage of patients achieving parasite clearance at 72 h post-dose, PCt1/2, Kelch13 genotype and the relationship between Kelch13 genotype and PCt1/2 are also reported.

ACPR was defined according to the WHO [20] as absence of parasitaemia on day X, irrespective of axillary temperature, in patients who did not previously meet any of the criteria of early treatment failure (ETF), late clinical failure (LCF) or late parasitological failure (LPF). The definition of ETF was a slight modification of the WHO definition [20] (see Additional file 2: S2 Statistical analysis plan, Section 15 Efficacy outcomes). The derivation of crude (unadjusted) and PCR-adjusted ACPR for both the ITT and PP populations (for details see Additional file 2: S2 Statistical analysis plan, Section 15 Efficacy outcomes) and of re-emergence, recrudescence and new infection was also done according to the principles set down by the WHO and MMV [20, 21]. The PCR methodology was in accordance with the procedures to identify parasite populations recommended by the WHO and MMV [22]. Three polymorphic genetic markers, MSP1, MSP2 and GluRP, were used to distinguish recrudescence from new infections, according to WHO-recommended procedures and as previously described by Snounou et al. [21, 23]. Recrudescence was defined as at least one identical allele for each of the three markers in the pre-treatment and post-treatment samples. New infections were diagnosed when all alleles for at least one of the markers differed between the two samples. Kelch13 genotyping (baseline) was determined by the method of the Pasteur Institute [24], PCt1/2 was calculated using the WWARN calculator [25] and the relationship between Kelch13 genotype and PCt1/2 was explored by site and by mutation graphically, and summary statistics reported. Details of the derivation and definitions of other endpoints are provided in Additional file 2: S2 Statistical analysis plan, Section 15 Efficacy outcomes).

Safety and tolerability endpoints included incidence of adverse events (AEs) and serious AEs (SAEs), vital signs, physical measurements, laboratory safety measurements, liver function tests (LFT) increase, cases fulfilling the Hy's law definition and ECG abnormalities including absolute QTc value categorisation and change from baseline QTc. Further details are given in Additional file 2: S2 Statistical analysis plan, Section 16 Safety outcome.

Treatment emergent adverse events of special interest (TEAESIs), requiring rapid reporting, were also defined in the protocol to ensure careful monitoring:

1. Hepatic: Hy's law definition cases; any alanine transaminase (ALT) or aspartate transaminase (AST) ≥ 5 × the upper limit of the normal range (ULN); any elevation in total bilirubin ≥ 2.5 × ULN (> 35% direct); any AST or ALT ≥ 3 × ULN with the appearance of fatigue, nausea, vomiting, right upper quadrant pain or tenderness, fever, rash and/or eosinophilia (eosinophil percent or count above the ULN); or ALT ≥ 3 × ULN that persisted for > 4 weeks
2. Cardiac: QTcF prolongation from baseline of > 60 ms; QTcF at any time; QTcF > 450 ms; T-wave liability or T-wave morphologic changes during therapy; bundle branch block; and any arrhythmia
3. Haematological: Haemoglobin (Hb) drop > 2 g/dL from baseline; Hb drop < 5 g/dL; absolute neutrophil count (ANC) < 1000/μL
4. Pregnancy.

### Statistical considerations

The aim of the study was to determine whether any of the treatment arms reached a target PCR-adjusted ACPR28 of ≥ 95% (PP analysis set). The study was not powered for comparison between dosing arms. ACPR28 was categorised as Cure and Failure according to the WHO method [20]. Descriptive statistics and a 95% Clopper–Pearson two-sided confidence interval (CI) were constructed around the single binomial proportion per treatment arm and for all treatment arms combined. Similar analyses were performed for the ITT and modified per protocol population (mPP) analysis sets across all days, and for crude ACPR. Descriptive summary statistics were also produced for all secondary and exploratory endpoints. Further details can be found in Additional file 2: S2 Statistical analysis plan, Section 15 Efficacy outcomes and Section 6.6 Statistical tests.

Trial simulations suggested that a treatment arm size of 106 should be required if the dose combination is effective [26]. Recruitment was to be capped at a maximum of 150 patients from the target population (patients ≤ 5 years old in Africa or patients of any age in Asia) per treatment arm. The study design was adaptive, allowing interim assessment of response for the purpose of concluding futility after recruitment of 50 evaluable patients per arm (target population). Futility was to be concluded if the probability that ACPR28 was ≤ 90% was greater than or equal to 0.3 [27]. Additionally, African patients > 5 years old were recruited during the age step-down, and these patients were included in the final analysis but not the interim futility analysis. Further details are given in Additional file 1: S1 Study protocol, Section 11 Statistical methods and data management.

### Pharmacokinetic analysis

In adult patients (weighing > 35 kg) blood samples for pharmacological analysis were collected at 15 to 16 time points: pre-dose, 2, 4, 6, 12, 24, 48, 72 h and days 5, 7, 10, 14, 21, 28, 42, 63. In paediatric patients the number was between 3 and 10 samples. Artefenomel and piperaquine PK data were analysed separately using non-linear mixed effect modelling (population PK analysis) in Monolix (version 4.3.3) or NONMEM (version 7.3 or later) respectively. For artefenomel, additional data from two mono-therapy clinical phase II studies in adult Asian patients were included in order to extend the dose range (100–1200 mg); see Additional file 3: S3 Pharmacokinetic analysis details.

Subsequently, exposures of artefenomel and piperaquine for each patient were derived from the individual PK parameters estimated in the population PK analysis. Maximum plasma concentration (Cmax), time to reach maximum concentration (Tmax) and concentration on day 7 (Cday7) were obtained from the simulated profiles, and the area under the curve (AUC) extrapolated to infinity (AUCinf) was calculated directly from the estimated PK parameters. More details are provided in Additional file 3: S3 Pharmacokinetic analysis details.

### Exposure–response analysis

The relationship of the binary outcome of ACPR28 response to the estimated artefenomel and piperaquine Cday7 and other covariates was evaluated in a logistic regression model using the statistical software R (version 3.2.2). Within the single dose setting of the study, Cday7 is highly correlated with other exposure variables, such as AUCinf or Cday14. However, Cday7 was preferred to allow future extrapolation to multi-dose regimens as well for its scientific rationale (concentrations of any given drug may be required to exceed the minimum parasiticidal concentration for at least 7 days to achieve full parasite clearance). Additional covariates evaluated were presumed immunity status (low for African patients ≤ 5 years and Asian patients of all ages), region, baseline parasitaemia, age and Kelch13 genotype. All patients in the ITT set with ACPR28 values and exposure for both drugs were included in the analysis. More details are provided in Additional file 4: S4 Exposure–response analysis details.

### Dose–response simulations

The objective of the simulations was to evaluate the dose–response relationship for single dose combination treatment with artefenomel and PQP based on the developed population PK and exposure–response models. The simulations were performed for a range of single dose combination doses (for a TPGS formulation) for the African population ≤ 5 years of age. Actual doses assumed the same body weight bands and dose adjustments applied to this study. For further details see Additional file 4: S4 Exposure–response analysis details.

## Results

### Interim analysis

An interim futility analysis was carried out after recruitment of approximately 50 evaluable patients from the target population per treatment arm as planned. All doses were concluded to have reached the futility criteria (probability of ACPR28 < 90% was 0.9999); hence, the study was stopped. Recruitment to the study continued during the futility analysis process.

### Final analysis

#### Analysis populations

The analysis populations are shown in Fig. 1.

#### Patient disposition and demographics

A total of 448 patients were randomised equally to the three treatment groups (randomised set, Table 2), and 437, *n* = 355 in Africa and *n* = 82 in Asia (Vietnam) received the study drug (ITT/safety set, Table 1). Demographics and patient characteristics by region are given in Table 1.

**Table 1:** Demographics and patient characteristics (safety set)

All patients		800:640 (*N* = 143)	800:960 (*N* = 148)	800:1440 (*N* = 146)	Total (*N* = 437)
Africa	Number (*n*)	116	121	118	355
Age (years) (derived)	Median	3.30	3.20	2.90	3.10
	(Min., max.)	(0.5, 54.3)	(0.8, 44.6)	(0.5, 37.7)	(0.5, 54.3)
> 15.0 years	*n* (%)	15 (12.9)	16 (13.2)	14 (11.9)	45 (12.7)
> 5.0 to ≤ 15.0 years	*n* (%)	7 (6.0)	7 (5.8)	8 (6.8)	22 (6.2)
> 2.0 to ≤ 5.0 years	*n* (%)	69 (59.5)	72 (59.5)	70 (59.3)	211 (59.4)
≥ 0.5 to ≤ 2.0 years	*n* (%)	25 (21.6)	26 (21.5)	26 (22.0)	77 (21.7)
Male	*n* (%)	56 (48.3)	63 (52.1)	70 (59.3)	189 (53.2)
Vietnam	*n*	27	27	28	82
Age (years) (derived)	Median	27.30	27.30	28.60	27.45
	(Min., max.)	(12.5, 48.5)	(9.7, 60.0)	(13.3, 57.6)	(9.7, 60.0)
> 15.0 years	*n* (%)	26 (96.3)	26 (96.3)	27 (96.4)	79 (96.3)
> 5.0 to ≤ 15.0 years	*n* (%)	1 (3.7)	1 (3.7)	1 (3.6)	3 (3.7)
> 2.0 to ≤ 5.0 years	*n* (%)	0	0	0	0
≥ 0.5 to ≤ 2.0 years	*n* (%)	0	0	0	0
Male	*n* (%)	27 (100.0)	22 (81.5)	28 (100.0)	77 (93.9)

*n* number of patients in each category/%)

Demographic characteristics were similar across treatment arms and analysis sets. Age (and hence weight) differed by region due to the enrolment structure. For the ITT/safety set, in Africa, 81.1% of patients were ≤ 5 years old, whereas in Asia 96.3% were > 15 years old. No patients ≤ 5 years old were recruited in Asia. There was also a difference in sex; 48.3% of patients in Africa and 93.9% in Asia were male.

Median baseline asexual parasitaemia across all treatment arms was 12,913/μL (range 187/μL to 220,240/μL), was similar in Asian and African patients ≤ 5 years, 13,140/μL (range 1065/μL to 123,080/μL) and 14,029/μL (range 187/μL to 229,240/μL) respectively and was slightly lower in African patients > 5 years, 9714/μL (range 835/μL to 160,040 μL).

Of those randomised, 178 (39.7%) completed the study up to day 42 (or 63) (Table 2). Of the 270 patients (60.3%) prematurely discontinued from the study, the majority (47.1%) were discontinued due to meeting the multiple criteria to receive antimalarial rescue treatment, either from failure to clear baseline parasitaemia (1.6%) or from parasite recurrence (i.e. treatment failure; 45.5%). Thus, premature study discontinuation prior to day 28 (and prior to day 42 or 63) is linked with the efficacy endpoint ACPR.

**Table 2:** Patient disposition (randomised set)

All patients		800:640 (*N* = 148)	800:960 (*N* = 151)	800:1440 (*N* = 149)	Total (*N* = 448)
Treated	*n* (%)	143 (96.6)	148 (98.0)	146 (98.0)	437 (97.5)
Completed	*n* (%)	57 (38.5)	56 (37.1)	65 (43.6)	178 (39.7)
Premature study discontinuation	*n* (%)	91 (61.5)	95 (62.9)	84 (56.4)	270 (60.3)
Primary reason for premature study discontinuation					
Criteria met for established anti-malarial treatment	*n* (%)	68 (45.9)	79 (52.3)	64 (43.0)	211 (47.1)
Study drug discontinued	*n* (%)	0	4 (2.6)	3 (2.0)	7 (1.6)
Withdrawal of consent	*n* (%)	9 (6.1)	3 (2.0)	7 (4.7)	19 (4.2)
Investigator’s opinion	*n* (%)	1 (0.7)	1 (0.7)	0	2 (0.4)
Patient non-compliant	*n* (%)	0	1 (0.7)	0	1 (0.2)
Adverse event	*n* (%)	0	0	1 (0.7)	1 (0.2)
Lost to follow-up	*n* (%)	5 (3.4)	3 (2.0)	3 (2.0)	11 (2.5)
Other	*n* (%)	8 (5.4)	4 (2.6)	6 (4.0)	18 (4.0)

*n* number of patients in each category/%)

#### Compliance

Protocol defined compliance; consumption of the total volume of study drug without vomiting (or successful re-dosing in the event of vomiting within 5 min of dosing) was 65% in the African population and 91.5% in the Asian population. Non-compliance was predominantly due to vomiting, with an overall incidence of vomiting of 28.8% (35% in Africa and 7% in Asia). Compliance and vomiting incidence were similar in the African population across age. There were anecdotal reports that, in some centres, young children were unable to ingest the full study dose, despite a reported success rate of administration of study drug (with or without subsequent vomiting) of > 95% in all populations.

#### Efficacy: ACPR

Crude and PCR-adjusted ACPR results are reported in Table 3 for the ITT analysis set. Re-emergence, crude and PCR-adjusted ACPR results for the PP analysis set are reported in Table 4.

**Table 3:** Crude and PCR-adjusted ACPR by day: ITT analysis set

		800:640 (*N* = 143)	800:960 (*N* = 148)	800:1440 (*N* = 146)	Total (*N* = 437)
Day 28					
Crude ACPR	*n*/*r* (%)	76/143 (53.1)	79/148 (53.4)	92/146 (63.0)	247/437 (56.5)
	95% CI^a^	[44.63; 61.53]	[45.01; 61.61]	[54.64; 70.85]	[51.73; 61.23]
PCR-adjusted ACPR	*n*/*r* (%)	77/143 (53.8)	82/148 (55.4)	95/146 (65.1)	254/437 (58.1)
	95% CI^a^	[45.32; 62.21]	[47.02; 63.57]	[56.75; 72.76]	[53.34; 62.79]
Day 42					
Crude ACPR	*n*/*r* (%)	63/143 (44.1)	66/148 (44.6)	68/146 (46.6)	197/437 (45.1)
	95% CI^a^	[35.77; 52.59]	[36.43; 52.98]	[38.29; 55.01]	[40.35; 49.88]
PCR-adjusted ACPR	*n*/*r* (%)^a^	67/143 (46.9)	72/148 (48.6)	73/146 (50.0)	212/437 (48.5)
	95% CI^a^	[38.47; 55.37]	[40.36; 56.99]	[41.62; 58.38]	[43.74; 53.31]
Day 63^b^					
Crude ACPR	*n*/*r* (%)^a^	50/136 (36.8)	49/140 (35.0)	58/135 (43.0)	157/411 (38.2)
	95% CI^a^	[28.67; 45.45]	[27.14; 43.51]	[34.48; 51.76]	[33.48; 43.09]
PCR-adjusted ACPR	*n*/*r* (%)^a^	50/136 (36.8)	54/140 (38.6)	59/135 (43.7)	163/411 (39.7)
	95% CI^a^	[28.67; 45.45]	[30.47; 47.16]	[35.19; 52.50]	[34.90; 44.57]

*n* number of patients in each category achieving ACPR, *r* total number of patients in the relevant analysis set with a defined response of Cure or Failure, *N* total number of patients in relevant analysis set

^a^Clopper–Pearson

^b^Patients followed up to day 63 consented separately from the patients followed up to day 42; hence, total patient population is lower for day 63

**Table 4:** Re-emergence, crude and PCR-adjusted ACPR by day: PP analysis set

		800:640 (*N* = 139)	800:960 (*N* = 140)	800:1440 (*N* = 139)	Total (*N* = 418)
Day 28					
Re-emergence	*n*/*r* (%)	48/129 (37.2)	56/136 (41.2)	39/133 (29.3)	143/398 (35.9)
Recrudescence	*n*/*r* (%)	25/129 (19.4)	37/136 (27.2)	22/133 (16.5)	84/398 (21.1)
	95% CI^a^	[12.95; 27.26]	[19.93; 35.50]	[10.67; 23.97]	[17.20; 25.45]
New infection	*n*/*r* (%)	11/129 (8.5)	10/136 (7.4)	12/133 (9.0)	33/398 (8.3)
	95% CI^a^	[4.33; 14.75]	[3.58; 13.11]	[4.75; 15.23]	[5.78; 11.45]
Indeterminate	*n*/*r* (%)	3/129 (2.3)	0	0	3/398 (0.8)
Negative	*n*/*r* (%)	2/129 (1.6)	1/136 (0.7)	0	3/398 (0.8)
Missing	*n*/*r* (%)	7/129 (5.4)	8/136 (5.9)	5/133 (3.8)	20/398 (5.0)
Crude ACPR	*n*/*r* (%)	74/129 (57.4)	77/136 (56.6)	89/133 (66.9)]	240/398 (60.3)
	95% CI^a^	[48.36; 66.03]	[47.85; 65.09]	[58.23; 74.83	[55.31; 65.14]
PCR-adjusted ACPR	*n*/*r* (%)	75/106 (70.8)	80/117 (68.4)	92/117 (78.6)	247/340 (72.6)
	95% CI^a^	[61.13; 79.19]	[59.13; 76.66]	[70.09; 85.67]	[67.58; 77.32]
Day 42					
Re-emergence	*n*/*r* (%)	59/127 (46.5)	64/134 (47.8)	56/130 (43.1)	179/391 (45.8)
Recrudescence	*n*/*r* (%)	29/127 (22.8)	37/134 (27.6)	25/130 (19.2)	91/391 (23.3)
	95% CI^a^	[15.86; 31.12]	[20.24; 36.00]	[12.85; 27.07]	[19.17; 27.78]
New infection	*n*/*r* (%)	16/127 (12.6)	17/134 (12.7)	18/130 (13.8)	51/391 (13.0)
	95% CI^a^	[7.38; 19.65]	[7.57; 19.53]	[8.42; 21.00]	[9.87; 16.79]
Indeterminate	*n*/*r* (%)	3/127 (2.4)	1/134 (0.7)	3/130 (2.3)	7/391 (1.8)
Negative	*n*/*r* (%)	3/127 (2.4)	1/134 (0.7)	0	4/391 (1.0)
Missing	*n*/*r* (%)	8/127 (6.3)	8/134 (6.0)	10/130 (7.7)	26/391 (6.6)
Crude ACPR	*n*/*r* (%)	61/127 (48.0)	65/134 (48.5)	67/130 (51.5)	193/391 (49.4)
	95% CI^a^	[39.09; 57.07]	[39.79; 57.29]	[42.62; 60.39]	[44.30; 54.43]
PCR-adjusted ACPR	*n*/*r* (%)^a^	65/100 (65.0)	71/108 (65.7)	72/100 (72.0)	208/308 (67.5)
	95% CI^a^	[54.82; 74.27]	[55.99; 74.60]	[62.13; 80.52]	[61.99; 72.73]
Day 63^b^					
Re-emergence	*n*/*r* (%)	59/114 (51.8)	70/122 (57.4)	53/116 (45.7)	182/352 (51.7)
Recrudescence	*n*/*r* (%)	29/114 (25.4)	37/122 (30.3)	23/116 (19.8)	89/352 (25.3)
	95% CI^a^	[17.75; 34.45]	[22.33; 39.30]	[13.00; 28.25]	[20.83; 30.16]
New infection	*n*/*r* (%)	15/114 (13.2)	20/122 (16.4)	16/116 (13.8)	51/352 (14.5)
	95% CI^a^	[7.56; 20.77]	[10.31; 24.18]	[8.09; 21.43]	[10.98; 18.61]
Indeterminate	*n*/*r* (%)	4/114 (3.5)	3/122 (2.5)	4/116 (3.4)	11/352 (3.1)
Negative	*n*/*r* (%)	3/114 (2.6)	1/122 (0.8)	0	4/352 (1.1)
Missing	*n*/*r* (%)	8/114 (7.0)	9/122 (7.4)	10/116 (8.6)	27/352 (7.7)
Crude ACPR	*n*/*r* (%)^a^	48/114 (42.1)	48/122 (39.3)	57/116 (49.1)	153/352 (43.5)
	95% CI^a^	[32.92; 51.71]	[30.62; 48.59]	[39.74; 58.58]	[38.22; 48.82]
PCR-adjusted ACPR	*n*/*r* (%)^a^	48/83 (57.8)	53/90 (58.9)	58/84 (69.0)	159/257 (61.9)
	95% CI^a^	[46.49; 68.60]	[48.02; 69.16]	[58.02; 78.69]	[55.63; 67.83]

*n* number of patients in each category achieving ACPR, *r* total number of patients in the relevant analysis set with a defined response of Cure or Failure, *N* total number of patients in relevant analysis set

^a^Clopper–Pearson

^b^Patients followed up to day 63 consented separately from the patients followed up to day 42; hence, total patient population is lower for day 63

**Fig. 2:**
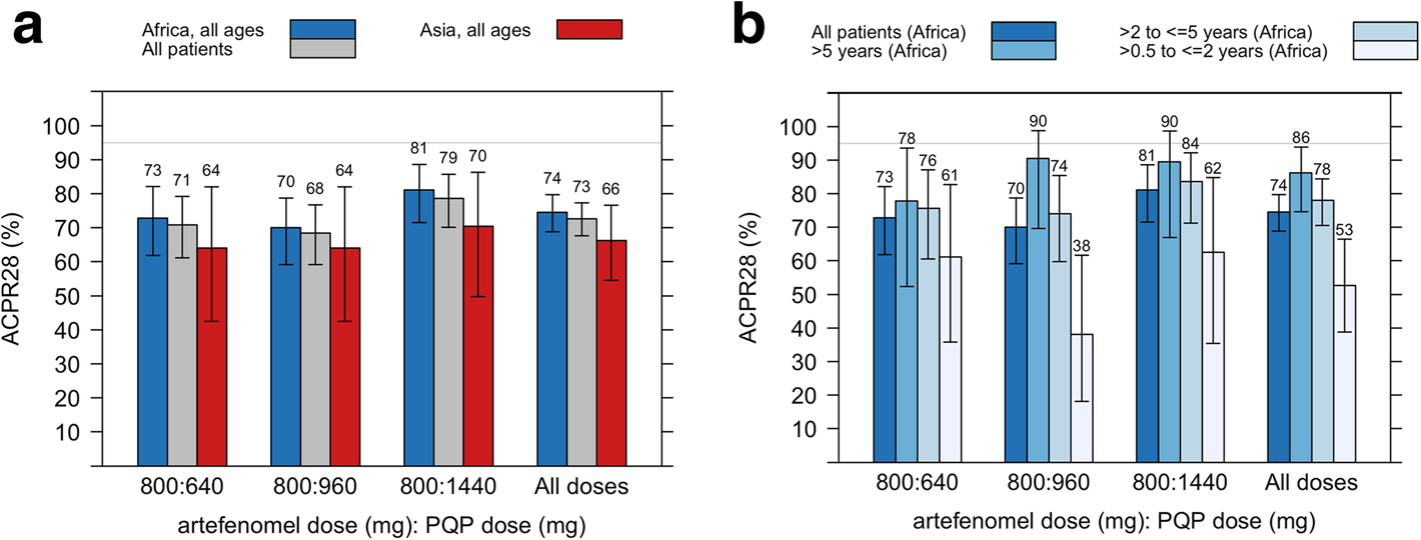
Efficacy: PCR-adjusted ACPR28 in the PP population, **a** by region (all ages), **b** by age in African patients, percentage in each category with 95% confidence intervals. The numbers presented above the bars are the percent ACPR28. The majority of treatment failures were late parasitological failures (32.9% across the populations and treatment arms), with one early treatment failure in an African patient > 5 years (PQP 640 mg arm)

For the primary analysis set (PP) and endpoint (ACPR28), none of the treatment arms reached the target efficacy of ≥ 95% and there was no clear dose–response relationship, although efficacy was highest for PQP 1440 mg (78.6%; 95% CI 70.09–85.67) across the populations (Table 4; Fig. 2b).

Efficacy in the mPP population (excluding patients who vomited) was similar to that in the PP population; e.g. for a PQP dose of 1440 mg, ACPR28 was (80.0%; 95% CI 69.92–87.90) across the populations.

ACPR28 appeared lower in Asian (Vietnamese) than in African patients; thus, for all treatment arms combined, ACPR28 was 66.2% (95% CI 54.6–76.6) and 74.5% (95% CI 7.81–79.7) respectively (Fig. 2a). In the African population, efficacy was lowest in the youngest age group (≥ 0.5 to ≤ 2 years), 52.7% (95% CI 38.8–66.4) for all treatment arms combined (Fig. 2b).

In the PP population, recurrence in African and Vietnamese patients was 37.0% and 31.6% respectively at day 28, with all but one determined by PCR to be recrudescence in the Vietnamese population, whereas in the African population, approximately one third of recurring parasites was determined to be a new infection at day 28.

In Asia, in the PP population, the number and percentage of recrudescences and new infections, with 95% CIs, was 11/80 (13.8%; CI 7.07–23.27) and 0/80 at day 14, 18/79 (22.8%; CI 14.10–33.60) and 0/79 at day 21, 23/79 (29.1%; CI 19.43–40.42) and 1/79 (1.3%; CI 0.03–6.85) at day 28, 26/78 (33.3%; CI 23.06–44.92) and 1/78 (1.3%; CI 0.03–6.94) at day 42, and 27/75 (36.0%; CI 25.23–47.91) and 2/75 (2.7%; CI 0.32–9.30) at day 63.

In Africa, in the PP population, the number and percentage of recrudescences and new infections, with 95% CIs, was 34/325 (10.5%; CI 7.35–14.31) and 1/325 (0.3%) at day 14, 57/321 (17.8%; CI 13.74–22.39) and 19/321 (5.9%; CI 3.60–9.09) at day 21, 61/319 (19.1%; CI 14.95–23.87) and 32/319 (10.0%; CI 6.96–13.87) at day 28, 65/313 (20.8%; CI 16.41–25.69) and 50/313 (16.0%; CI 12.09–20.51) at day 42, and 62/277 (22.4%; CI 17.61–27.75) and 49/277 (17.7%; 13.38–22.70) at day 63.

**Fig. 3:**
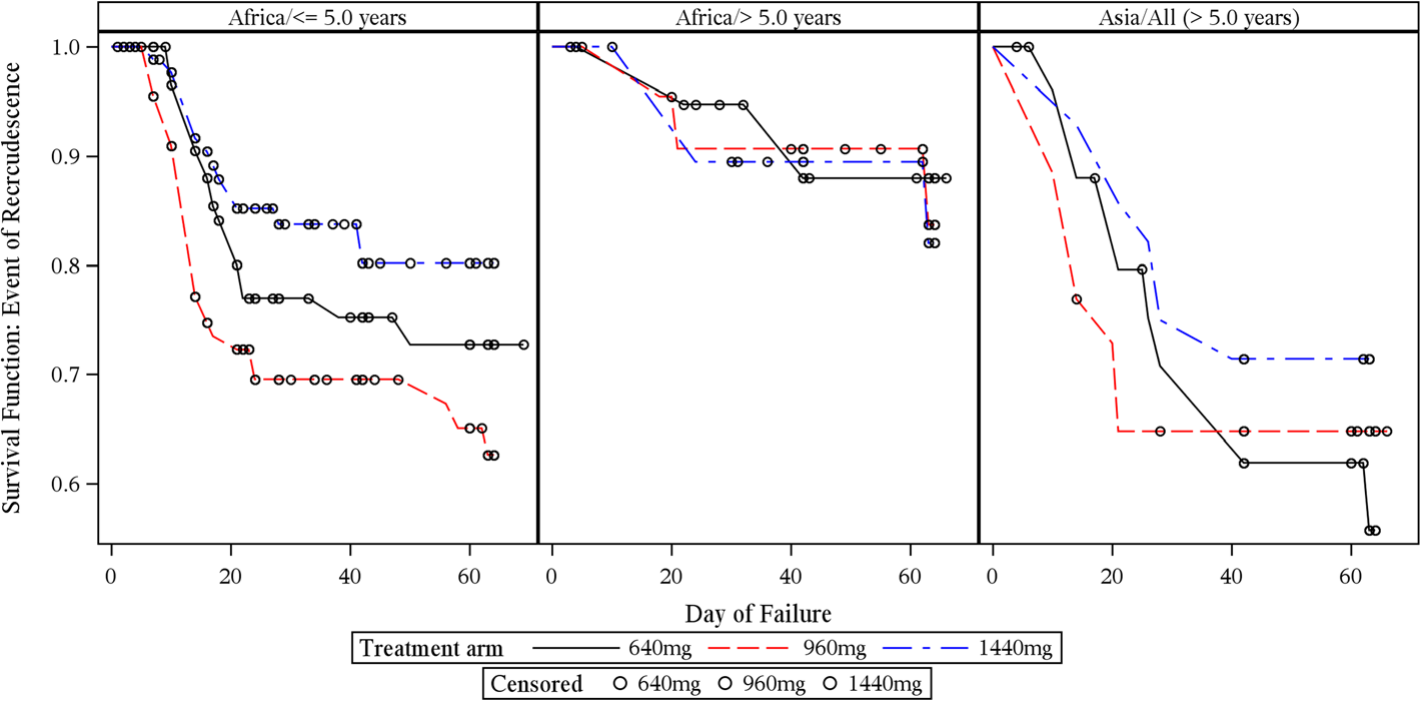
Kaplan–Meier population at risk of recrudescence by region and age group over time (ITT subset). Note that the *y*-axis is expanded (survival range 0.5–1.0) to clearly visualise the failure rates

**Fig. 4:**
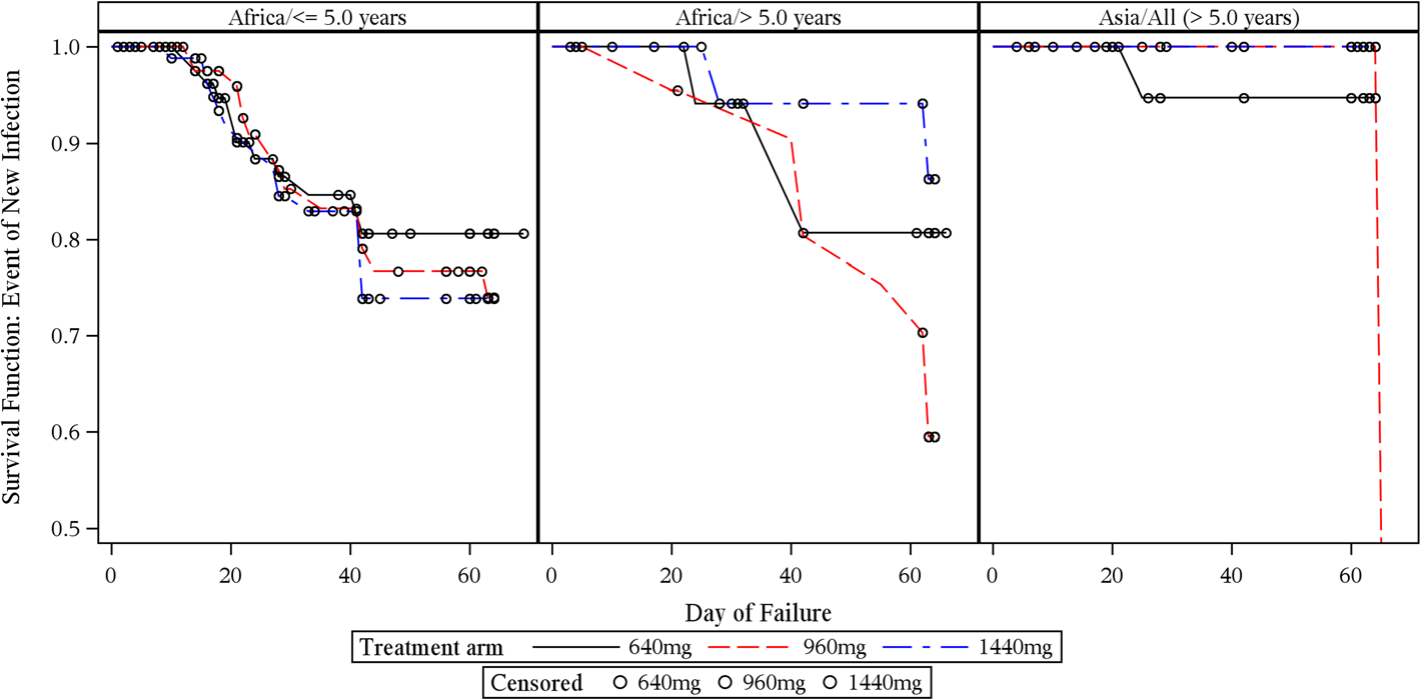
Kaplan–Meier population at risk of new infection by region and age group over time (ITT subset). Note that the *y*-axis is expanded (survival range 0.5–1.0) to clearly visualise the failure rates

Kaplan–Meier estimates of the fraction of patients with recrudescence and new infection over time by region and age group are presented in Figs. 3 and 4 respectively.

#### Parasite clearance and association with Kelch13 mutation

A regional difference in the percentage of patients who cleared parasites by 72 h post-dose was evident, with 92.9% (95% CI 89.6–95.4) of African patients achieving parasite clearance by 72 h post-dose compared with 35.0% (95% CI 24.7–46.5) in Vietnamese patients. There was no clear difference in the percentage of patients who cleared parasites across the different piperaquine doses or in the African population > 5 years compared with those ≤ 5 years.

The median time to 50%, 90% and 99% parasite clearance was approximately twice as long in Asia compared with Africa (e.g. time to 90% parasite clearance was 23.6 (interquartile range, IQR 16.20–29.10) h in Asian patients and 14.30 (IQR 10.90–17.60) h in African patients.

Initial clearance of parasites was rapid in the African population. Median parasite clearance half-life (PCt1/2) was calculated using the WWARN Parasite Clearance Estimator (PCE), details of which are published [25]. PCt1/2 is the estimated time for parasitaemia to decrease by half, derived from the clearance rate constant 1/h. Parasite clearance values were reported only for results with *R* ^2^ > 0.75. PCt1/2 was longer in Vietnam versus Africa (6.1 h [minimum 1.1, maximum 12.7] versus 3.5 h [minimum 1.2, maximum 7.7]). Within Vietnam, PCt1/2 was similar across the four study centres.

A total of 20 known Kelch13 genotypes were tested for (in Africa four new genotypes were identified at low frequency, 0.3–1.7%: A578S, A626V, M562T, Y541F, none associated with artemisinin resistance) [28]. In Vietnam, a high frequency of Kelch13 mutation was observed (70.1%). Five mutations were detected, four of which, C580Y, I543T, P553L and V568G, are defined according to the WHO [28] as validated or candidate markers for partial artemisinin resistance. The exception is C469P, which is not known to be associated with artemisinin resistance [28].

**Fig. 5:**
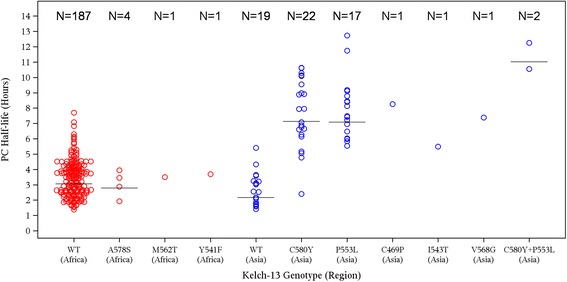
Association of parasite clearance half-life and Kelch13 Status (PP analysis set). Vietnam (*blue circles*), Africa (*red circles*), median (*black line*), C580Y + P553L polyclonal infection. Note that the plot includes only patients with both genotyping and PCt1/2 results

PCt1/2 appeared to be associated with Kelch13 genotype; median PCt1/2 values for C580Y and P553L, the two most frequently occurring mutations, were 7.9 (*N* = 24; minimum 2.4, maximum 12.3 h) and 8.1 h (*N* = 19; minimum 5.5, maximum 12.7) respectively, versus 2.6 h (*N* = 19; minimum 1.4, maximum 5.4) for wild type (WT) (Fig. 5). Mutations that were present at a lower frequency (1/67, 1.5%) also had greater PCt1/2 values; C469P (8.3 h), I543T (5.5 h) and V568G (7.4 h). PCt1/2 in patients with Kelch13 WT in Vietnam was similar to that in the African population (Fig. 5).

#### Safety and tolerability

No deaths were reported. One TEAE of vomiting occurring 19 min post-dose (treatment-related) was reported to have led to study treatment discontinuation.

Six treatment emergent serious adverse events (TESAEs) were reported in four patients. One patient had severe anaemia (2 days post-dose), one patient had a reversible haemoglobin drop < 5 g/dL (26 days post-dose) and one presented with febrile convulsions (7 h post-dose). One patient had three TESAEs, two of reversible grade 3 transaminase elevations and one of neutropenia (28 days post-dose). None of these TESAEs led to premature study discontinuation. Five of the six TESAEs were considered to be potentially related to the study treatment, the exception being that of febrile convulsion which was considered not related. No AEs due to drug-induced liver toxicity (Hy’s law or increase of ALT/AST with clinical symptoms for more than 4 weeks) were reported.

Table 5 gives the incidence of TEAEs occurring in ≥ 5% of the study population up to 28 days post-dose, by System Organ Class. All events of transaminase elevations were reversible, except in one subject who withdrew consent, interrupting the liver function test follow-up.

**Table 5:** Incidence of treatment-emergent adverse events in ≥ 5% of study population up to day 28 post-dose (safety analysis set)

	Artefenomel mg: PQP mg			
System Organ Class Preferred term	800:640 (*N* = 143)	800:960 (*N* = 148)	800:1440 (*N* = 146)	Total (*N* = 437)
At least 1 TEAE (*n* (%) *E*)	115 (80.4) 266	127 (85.8) 324	122 (83.6) 308	364 (83.3) 898
Infections and infestations (*n* (%) *E*)	74 (51.7) 102	76 (51.4) 106	63 (43.2) 82	213 (48.7) 290
Malaria	43 (30.1) 44	45 (30.4) 48	34 (23.3) 34	122 (27.9) 126
Bronchitis	13 (9.1) 16	13 (8.8) 19	10 (6.8) 11	36 (8.2) 46
Rhinitis	11 (7.7) 11	9 (6.1) 10	10 (6.8) 10	30 (6.9) 31
*Plasmodium falciparum* infection	9 (6.3) 9	10 (6.8) 10	7 (4.8) 7	26 (5.9) 26
Investigations (*n* (%) *E*)	58 (40.6) 75	68 (45.9) 100	70 (47.9) 95	196 (44.9) 270
Electrocardiogram QT prolonged	27 (18.9) 29	41 (27.7) 48	44 (30.1) 56	112 (25.6) 133
Neutrophil count decreased	18 (12.6) 18	11 (7.4) 11	12 (8.2) 13	41 (9.4) 42
Haemoglobin decreased	9 (6.3) 9	20 (13.5) 22	11 (7.5) 11	40 (9.2) 42
Gastrointestinal disorders (*n* (%) *E*)	31 (21.7) 39	47 (31.8) 60	44 (30.1) 54	122 (27.9) 153
Diarrhoea	11 (7.7) 12	21 (14.2) 21	20 (13.7) 20	52 (11.9) 53
Vomiting	14 (9.8) 14	20 (13.5) 20	16 (11.0) 16	50 (11.4) 50
Abdominal pain	5 (3.5) 5	8 (5.4) 9	12 (8.2) 13	25 (5.7) 27
General disorders and administration site conditions (*n* (%) *E*)	11 (7.7) 11	14 (9.5) 16	17 (11.6) 21	42 (9.6) 48
Pyrexia	5 (3.5) 5	12 (8.1) 13	9 (6.2) 11	26 (5.9) 29

*N* number of subjects affected (%), *E* number of events

The most frequently reported TEAESI was QT prolongation in ECG: 24 (16.8%), 37 (25.0%) and 38 (26.0%) of patients in the PQP 640 mg, 960 mg and 1440 mg treatment arms respectively. A first degree atrioventricular (AV) block reported as grade 1 at 48 h post-dose (PR interval 226.33 ms, heart rate 55 beats per min and QTcB and QTcF within the normal range) was reported in one patient (PQP 640 mg). The event resolved at day 7 post-dose (PR interval 185 ms and heart rate 78 beats per min). One patient (PQP 1440 mg) had a mild reversible sinus bradycardia which resolved in 4 days. QTcF increase from baseline of 30–60 ms occurred in 55 (38.7%), 59 (40.4%) and 71 (49.7%) and of > 60 ms in 8 (5.6%), 11 (7.5%) and 27 (18 .9%) of patients respectively in the 640, 960 and 1440 mg PQP dosing arms. All but one QTcF value was < 480 ms (QTc value = 501 ms).

One patient (PQP 1440 mg) experienced a reversible TEAESI of hyperbilirubinaemia (total bilirubin > 2.5 × ULN) in the System Organ Class hepatobiliary disorders. This event was associated with a TESAE of anaemia (Hb drop > 2 g/dL from baseline). Other frequent TEAESIs were neutrophil count decreased < 1000/μL (41 patients; 9.4%) and Hb decreased (drop > 2 g/dL from baseline or Hb < 5 g/dL: 40 patients; 9.2%). The most significant tolerability finding was vomiting (28.8%) according to the compliance data. The high rate of vomiting is thought to be partly related to the 'high volume' TPGS formulation used in the study (although the reason for the regional difference in vomiting rate is unclear).

#### Pharmacokinetic results

The final artefenomel and piperaquine population PK models, including details of the analysis and model diagnostics, are provided in Additional file 3: S3 Pharmacokinetic analysis details.

The PK of both artefenomel and piperaquine in adult and paediatric patients could be described by three compartment disposition models. All (apparent) clearance and volume parameters were related to body weight allometrically. Additional covariates identified for the PK of artefenomel were vomiting, artefenomel dose and age. In particular, relative bioavailability was a function of age; it was 40% lower for a patient of 1 year versus 20 years of age. For the PK of piperaquine, the only additional covariate identified was vomiting. No covariate effects of region, age (for piperaquine), sex, protocol-defined non-compliance or actual or adult equivalent PQP dose were identified.

**Fig. 6:**
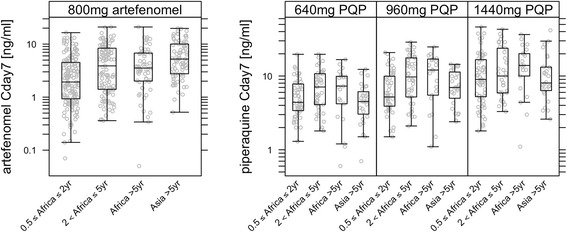
Estimated Cday7 for artefenomel (**a**) and PQP (**b**) by region/age group. Patients who vomited are included. Median (horizontal line), box (interquartile range) and whiskers represent the most extreme individual point which is not more than 1.5× the length of the box

Individual artefenomel and piperaquine exposures were estimated for 427 and 426 patients respectively (including patients who vomited and were not successfully re-dosed). Summaries of the individual estimated exposures by region and age group are provided in Additional file 3: S3 Pharmacokinetic analysis details). Cday7 is summarised across region and age group in Fig. 6.

Geometric mean artefenomel exposure was lowest in the African population 0.5 to ≤ 2 years of age (which includes body weight bands 5–14.9 kg). Thus, AUCinf and Cday7 were 7.6 μg*h/mL (coefficient of variation, CV 105%) and 2.0 ng/mL (CV 147%) respectively compared with 10.0 μg*h/mL (CV 111%) and 3.3 ng/mL (CV 141%) for African patients > 5 years. Asian patients > 5 years (all but one over 35 kg) had higher mean exposures than the African patients; AUCinf of 16.9 μg*h/mL (CV 66%) and Cday7 of 5.1 ng/mL (CV 95%) (Fig. 6a). Artefenomel exposures and between-patient variability were similar across the three PQP treatment arms.

Piperaquine exposures increased approximately proportionally with dose, although there was considerable overlap between the treatment arms. Exposures tended to be lower in the African population ≤ 2 years of age (including body weight band 5–9.9 kg) and in the Asian population (Fig. 6b). Exposures of artefenomel and piperaquine were about 50% and 70% lower respectively in patients who vomited relative to those who did not. See Additional file 3: S3 Pharmacokinetic analysis details for further details and Additional file 4: S4 Exposure–response analysis details for two patient exposure examples.

#### Exposure–response relationship

**Fig. 7:**
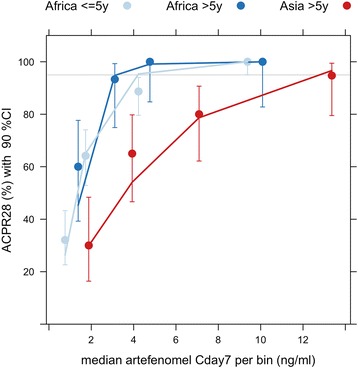
Summary of the observed ACPR28 by estimated artefenomel concentration at day 7 together with the logistic regression model predictions. The *dots* and *error bars* represent the observed ACPR28 with 90% CI for five artefenomel concentration bins (categories). The *lines* represent the final logistic regression model predictions taking into account the median artefenomel Cday7, as well as the median piperaquine Cday7 and median baseline parasitaemia, for each bin. Number of patients per bin: Africa ≤ 5 years *n* = 53, Africa > 5 years *n* = 15, Asia *n* = 20

When exploring the observed ACPR28 by artefenomel exposure bins (categorical Cday7 ranges) rather than dose, the relationship between exposure and ACPR28 is clearly visible (Fig. 7). The relationship was different between the two regions but similar for the two African age groups. Thus, in the Asian population, a lower ACPR28 was achieved for the same artefenomel Cday7 compared with the African population. Both the region effect and lack of age effect were confirmed in the subsequent statistical analysis (logistic regression).

The relation between artefenomel and piperaquine exposure and ACPR28 was described with a logistic regression model. Details of the model, analysis and model diagnostics are provided in Additional file 4: S4 Exposure–response analysis details. The analysis data set comprised 348 patients.

The probability of achieving ACPR28 (pACPR) was found to be a function of artefenomel and piperaquine exposures, the baseline parasitaemia (*p* ≤ 0.0001) and region (interaction between region and artefenomel Cday7) (*p* = 0.002):

$$ \log \left(\frac{p}{1-p}\right)=3.23+{0.22}^{\ast } Cday{7}_{PQ}+{\left(0.73-0.59\left[ if Asia\right]\right)}^{\ast } Cday{7}_{OZ}-{1.27}^{\ast }\ \mathit{\log}10(BasePar)+0.46\ \left[ if Asia\right] $$

where *p* is the probability of ACPR28 and *BasePar* is the baseline parasitaemia (parasites/μL).

There was no statistically significant influence of either Kelch13 genotype or age on pACPR, and (once region was in the model) there was no additional influence of presumed immunity status. In addition, no interaction between the exposures of the two drugs was identified.

**Fig. 8:**
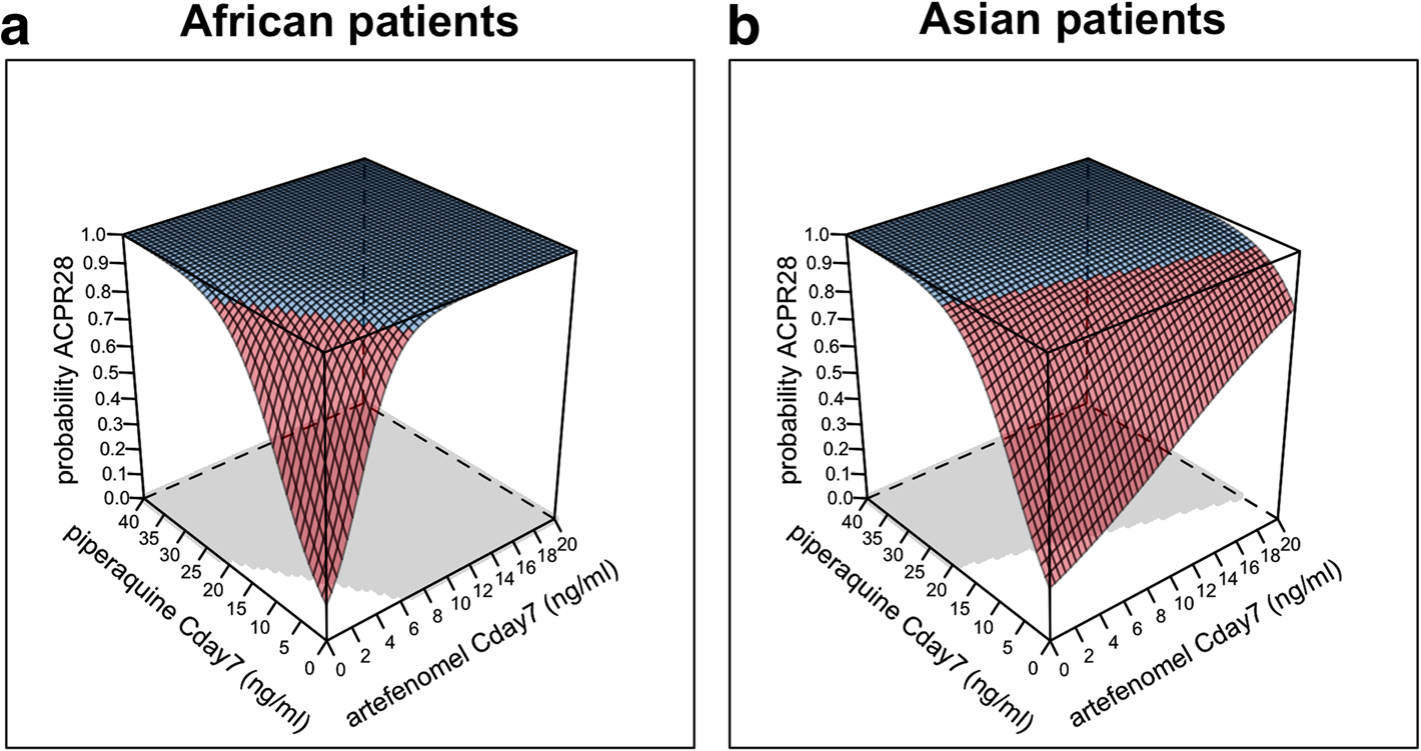
Estimated exposure–ACPR28 relationship for the combination of artefenomel with piperaquine for a baseline parasitaemia of 10,000 parasites/μL. **a** African patients, **b** Vietnamese patients, 3D representation; *blue* highlights the ACPR28 > 0.95. The *shaded area* shows the concentrations required to achieve a probability of ACPR28 > 0.95

Three-dimensional (3D) graphical representations of the exposure–ACPR28 model are shown in Fig. 8 for African and Vietnamese patients, assuming a baseline parasitaemia of 10,000 parasites/μL. These plots show that both artefenomel and piperaquine exposure (Cday7) contribute to efficacy (ACPR28) in a concentration-dependent manner, and that this relationship is different for African versus Vietnamese patients; that is, higher artefenomel exposure (but not higher piperaquine exposure) is required to achieve the same ACPR28 in Vietnamese (Asian) versus African patients (see the equation).

**Fig. 9:**
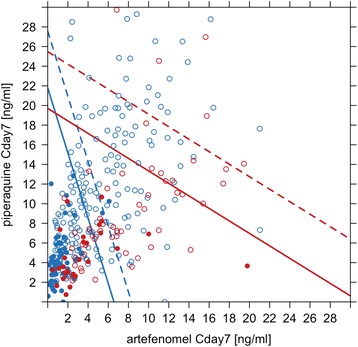
Concentrations associated with a probability of ACPR28 of 0.95: model-predicted isobolograms by region and baseline parasitaemia. Asian patients = *red*, African patients = *blue*. Baseline parasitaemia 10,000 parasites/μL, *solid isobole*; baseline parasitaemia 100,000 parasites/μL, *dotted isobole*. Includes actual estimated individual exposures associated with Cure (ACPR28), *open symbol* or Failure (recrudescence), *closed symbol*

This difference in sensitivity to artefenomel (about five times lower in Asian versus African patients) is illustrated by the difference in the isoboles plotted in Fig. 9 (red versus blue isoboles).

For example, if each drug were administered by itself (Cday7 = 0 for the partner drug), assuming a baseline parasitaemia of 100,000 parasites/μL in African patients, an artefenomel Cday7 of 8 ng/mL would be required for a 0.95 probability of achieving ACPR28, compared to 40 ng/mL in Asian patients. For each presented scenario, any exposure combination of artefenomel and piperaquine to the right of the isobole lines is associated with > 0.95 probability of achieving ACPR28.

Figure 9 also shows the observed individual exposure combinations flagged as Cure (open symbols) or Failure (closed symbols). These exposures are associated with a wide range of baseline parasitaemia levels. The data points are the actual data used to estimate the model, which is represented by the isoboles, and thus it is clear which part of the isobole is supported directly by the data and which is an extrapolation. For example, the inference that in African patients for a baseline parasitaemia of 100,000 parasites/μL an artefenomel Cday7 of 8 ng/ml or higher (administered alone) is predicted to have pACPR > 0.95 is an extrapolation, since there are no patient data supporting this scenario.

#### Dose–response relationship

The projected ACPR28 simulations for various dose combinations, taking into account between-subject variability as well as model parameter uncertainty in both PK and exposure response, are shown in Table 6. The identified age effect on the relative bioavailability of artefenomel was not included in the simulations. Thus, hypothetically, if similar exposures to African patients > 5 years were achieved in patients ≤ 5 years (e.g. alternative formulation or alternative dose adjustment), the doses highlighted in Table 6 may achieve an ACPR28 > 95%. Note that the projections for several dose combinations in the table are extrapolations from the model, both the mono-therapy (exposure–response component) and the higher dose levels (PK component).

**Table 6:** Simulated % ACPR28 with 90% confidence intervals for single dose combinations of artefenomel and PQP in non-vomiting African children ≤ 5 years old

PQP adult-equivalent dose, mg		Artefenomel adult-equivalent dose, mg					
		0	200	400	800	1200	1600
	0	NR (–)	22 (14–32)	39 (29–49)	70 (59–79)	86 (78–92)	94 (88–97)
	320	27 (18–37)	35 (28–44)	52 (45–59)	79 (73–84)	91 (86–94)	**96 (93–98)**
	640	40 (29–52)	48 (39–58)	64 (58–70)	85 (81–88)	94 (91–96)	**97 (95–99)**
	960	51 (38–64)	59 (48–69)	72 (65–78)	89 (85–92)	**95 (93–97)**	**98 (97–99)**
	1440	64 (49–76)	70 (59–80)	80 (73–86)	92 (89–95)	**97 (95–98)**	**99 (98–99)**
	2000	74 (59–85)	79 (67–88)	86 (80–91)	**95 (93–97)**	**98 (97–99)**	**99 (98–100)**
	2800	83 (70–91)	86 (76–93)	91 (85–95)	**97 (95–98)**	**99 (98–100)**	**> 99 (99–100)**
	3600	88 (78–95)	91 (83–96)	94 (90–97)	**98 (96–99)**	**99 (99–100)**	**> 99 (99–100)**

For the stimulations, the identified age effect on artefenomel PK was not included; it was assumed, therefore, that exposures in the youngest patients were similar to those in the older age groups. *NR* not reported

Predicted ACPR28 > 95%; outcome predicted with a lower bound > 95%

## Discussion

We report the results of the first phase II dose-ranging study to assess the potential of a single encounter curative treatment (artefenomel plus PQP) for uncomplicated *P. falciparum* malaria in adults and children in Africa and Asia (Vietnam).

None of the treatment arms reached the pre-specified target efficacy of ≥ 95% PCR-adjusted ACPR28 in the overall population or in any of the subpopulations, including African patients > 5 years of age, demonstrating that a single encounter treatment with artefenomel 800 mg plus up to 1440 mg PQP does not provide sufficient exposure for a sufficient duration to achieve the required efficacy. Efficacy appeared lower in Vietnamese than in African patients overall; however, the lowest efficacy was observed among the youngest African age group (> 0.5 to ≤ 2 years old).

The study was not powered to compare outcomes between treatment arms, and no clear PQP dose trend was identified in the primary analysis for ACPR28. This was due to large exposure variability and the limited dose range studied, resulting in overlapping exposures between treatment arms, coupled with the binary nature of the clinical endpoint. However, this large exposure range allowed establishment of an exposure–response relationship for both drugs in combination, which in turn resulted in identification of factors influencing efficacy, thereby providing a fuller understanding of the study results.

Thus, the exposure–response analysis demonstrated that both drugs contribute to efficacy (ACPR28) in a concentration-dependent manner, and as might be expected, higher baseline parasitaemia requires higher exposures to provide the same ACPR28.

Within Africa, the concentration–response relationship for artefenomel and piperaquine did not appear to differ with age (or presumed immunity). While age was not identified as a significant covariate for ACPR28, the numbers of African patients > 5 years old was relatively small and may have been insufficient to identify a difference. Instead, the lower efficacy in the youngest African patients appeared due to lower exposure to artefenomel (and to a lesser extent piperaquine). There was insufficient information to be able to identify the reason for the lower exposure. This may have been a consequence of incorrect dose adjustment to account for clearance/bioavailability differences across the age range; however, failure of the youngest children to consume the entire dose (there were anecdotal reports of this despite high reported compliance) or vomiting may have contributed.

The exposure–response analysis indicated that the lower efficacy (ACPR28) in the Vietnamese relative to the African population was due to lower sensitivity to artefenomel (but not to piperaquine), that is, a regional difference in the concentration–response relationship. The mechanism of this lower sensitivity is not known.

Kelch13 genotyping indicated a high frequency of patients infected with artemisinin resistant parasites within the Vietnamese sites. The most common Kelch13 genotypes were C580Y, the predominant validated marker of artemisinin resistance across the Greater Mekong Subregion, and P553L, a candidate marker of artemisinin resistance found in the Western Greater Mekong Subregion [28].

Artemisinin resistance is characterised by a decrease in the rate of parasite clearance following artemisinin mono-therapy or artemisinin-containing combination treatments in patients infected with parasites with mutations in the Kelch13 gene [29]. In vitro this is manifested by a decreased sensitivity to artemisinin of the early rings stage of the parasite lifecycle [30], and as such is considered partial resistance. Artemisinin partial resistance has not been shown to reduce the cure rate unless partner drug resistance is also present.

The current study is the first to fully evaluate the efficacy of a combination containing a synthetic endoperoxide (artefenomel) in patients infected with artemisinin resistant parasites, and the association between PCt1/2 following artefenomel/PQP treatment and Kelch13 mutation suggests that these mutations may drive a similar decrease in the rate of parasite clearance for artefenomel-containing combinations as with artemisinin-containing combinations [29]. In vitro data suggest that, similar to DHA, there is reduced sensitivity of early mutant rings to artefenomel [30]. However, Kech13 mutation was not identified as a significant covariate for ACPR28 in the model. Caution should be exercised here, as the sample size may well not have been sufficient to identify any association between Kelch13 and ACPR28 in the exposure–response analysis.

High rates of DHA–piperaquine treatment failures are now reported in the Greater Mekong Subregion, suggesting co-segregation, or at least coexistence of artemisinin and piperaquine resistance. Three of the four sites in Vietnam involved in the study (Gia Lai, Binh Phauc and Khanh Hoa) are located in provinces bordering Cambodia, and so conceivably artemisinin and piperaquine resistant genes could coexist. It is noteworthy that no difference was detected in the sensitivity (exposure–response relationship) for piperaquine between Vietnam and Africa. However, the sensitivity to piperaquine of *P. falciparum* parasites collected in this study is not currently known. Recent work to identify genetic markers of piperaquine resistance has confirmed that increased copy numbers of plasmepsin 2 and plasmepsin 3 genes, along with Pfmdr1 gene de-amplification, are independently associated with resistance to piperaquine, and that these markers of piperaquine resistance are prevalent in Cambodia and coexist with Kelch13 [31, 32]. We intend to investigate the frequency of genetic markers of piperaquine resistance in samples collected during this study and to investigate the relationship between Kelch13 and genetic markers of resistance, and PCt1/2 and ACPR.

We are also currently investigating the efficacy of artefenomel in combination with ferroquine. Both parent drug and circulating active metabolite have significantly longer half-life values than piperaquine, and hence this combination has a greater probability of achieving the target efficacy. Ex vivo studies performed with Cambodian clinical isolates suggest negligible impact of piperaquine resistance on ferroquine potency [33].

The significant rate of vomiting and the high dosing volume, particularly for young children, were problematic and may have contributed to the lower drug exposures in the youngest children. The development of age-appropriate formulations is key to the success of individual studies and to development programmes as a whole. Asymptomatic QTc increases from baseline were frequently reported as expected for PQP.

The study results also illustrate the challenges in developing a single encounter combination treatments. Firstly, the administered dose needs to be higher to achieve the required duration of exposure compared to multiple day treatments; therefore, the ratio of Cmax to overall exposure will be greater. Secondly, high between-subject variability in drug exposures, due in part to a limited number of body weight bands for dosing (and which in this study may have been compounded by challenges in administering large dosing volumes to sick children), in addition to a large between-subject variability in baseline parasitaemia and potentially parasite sensitivity, means that the majority of patients will be required to be 'overdosed' if a very high cure rate is to be achieved with a single (adult equivalent) dose level. Both factors mean that a wide therapeutic window is required.

### Limitation of the study

A significant limitation of the study was that, although the formulation used had been tested in adult healthy subjects, it had not been tested in adult and, more importantly, paediatric malaria patients. It is possible that the palatability of the formulation and/or volume of administration contributed to the higher than expected rate of vomiting in the study. In addition, although compliance data on drug consumption were collected, insufficient detail was recorded to truly capture compliance, since despite a high reported success rate of drug administration, there were anecdotal reports that young children were unable to ingest the full dose. The effect of this was that the study was unable to conclude on the best weight-based dose adjustment for patients weighing < 35 kg; i.e. drug exposure in young children was lower than in adults. However, it could not be concluded whether this was due to the weight-based dose adjustments or because the intended dosage was not successfully administered.

However, the dose–response simulations suggest that, even if the young children had similar exposures to the adults as well as no vomiting, the target efficacy would not have been achieved.

## Conclusions

None of the treatment arms reached the target efficacy of > 95% PCR-adjusted ACPR at day 28. Achieving very high efficacy following single dose treatment is challenging since > 95% of the population must have sufficient concentrations to achieve cure across a range of parasite sensitivities and baseline parasitaemia levels, meaning that a significant number of patients are 'overdosed'. Drugs with a substantial therapeutic window are therefore required. Projected single dose combination doses of artefenomel plus PQP that may achieve the target efficacy in the African population were not markedly higher than those tested in this study, but are most likely not clinically viable due to tolerability and practical dose size.

A high frequency of patients in Vietnam were infected with artemisinin resistant parasites, and an association between PCt1/2 following artefenomel/PQP treatment and Kelch13 mutation suggests that these mutations may drive a similar decrease in the rate of parasite clearance for artefenomel-containing combinations as observed with artemisinin-containing combinations [28].

While challenging, the development of tools suitable for deployment as single encounter curative treatments for adults, and particularly children in Africa, and to support elimination strategies remains a key development goal. There is currently no evidence that full artemisinin resistance has emerged in Southeast Asia; i.e. there is no evidence that reduced sensitivity of the early ring stage has progressed to full resistance [28]. The results of the efficacy study of artefenomel and ferroquine will inform us of the potential of artefenomel-containing combinations to treat malaria in both Africa and Southeast Asia where partial resistance to artemisinin and resistance to piperaquine is widespread.

## Additional files

Additional file 1: S1. Study Protocol. (PDF 1822 kb)

Additional file 2: S2. Statistical analysis plan. (PDF 1515 kb)

Additional file 3: S3. Pharmacokinetic analysis details. (DOCX 714 kb)

Additional file 4: S4. Exposure–response analysis details. (DOCX 274 kb)

## Abbreviations

ACPR: Adequate clinical and parasitological response
ACPR28: Adequate clinical and parasitological response on day 28
AE: Adverse event
ALT: Alanine transaminase
ANC: Absolute neutrophil count
AST: Aspartate transaminase
AUCinf: Area under the plasma concentration curve extrapolated to infinity
Cday7: Plasma concentration on day 7
CI: Confidence interval
Cmax: Maximum plasma concentration
CV: Coefficient of variation
DHA: Dihydroartemisinin
E: Number of events
ECG: Electrocardiogram
ETF: Early treatment failure
Hb: Haemoglobin
IEC: Independent Ethics Committee
IQR: Interquartile range
ISMB: Independent Safety Monitoring Board
ITT: Intention to treat
IWRS: Interactive Web Response System
KM: Kaplan–Meier
LCF: Late clinical failure
LPF: Late parasitological failure
MMV: Medicines for Malaria Venture
mPP: Modified per protocol population
n: Number of patients
OZ439: artefenomel
P.: Plasmodium
pACPR: probability of achieving adequate clinical and parasitological response
PCR: Polymerase chain reaction
PCt1/2: Parasite clearance half-life
PK: Pharmacokinetics
PP: Per protocol
PQP: Piperaquine phosphate
PRR24: Parasite reduction ratio at 24 hours (log10) post-treatment
SAE: Serious adverse event
TEAE: Treatment emergent adverse event
TEAESI: Treatment emergent adverse event of special interest
Tmax: Time to reach maximum plasma concentration
TPGS: α-tocopherol polyethylene glycol
ULN: Upper limit of the normal range
WHO: World Health Organization
WT: Wild type
