# Supplementary material for: A randomised, double-blind clinical phase II trial of the efficacy, safety, tolerability and pharmacokinetics of a single dose combination treatment with artefenomel and piperaquine in adults and children with uncomplicated Plasmodium falciparum malaria
Source: BMC Med. 2017 Oct 9;15:181. doi: 10.1186/s12916-017-0940-3 (PMC5632828; doi:10.1186/s12916-017-0940-3)
Supplement: Supplementary file 1 — Study Protocol. (PDF 1822 kb) [file 12916_2017_940_MOESM1_ESM.pdf]

CLINICAL STUDY PROTOCOL

**Product:**	OZ439 (Artefenomel) and Piperaquine Phosphate
**Sponsor:**	Medicines for Malaria Venture
**Protocol Number:**	MMV_OZ439_13_003
**Study Title:**	A Randomised, Double-blind, Phase IIb Study to Investigate the Efficacy, Safety, Tolerability and Pharmacokinetics of a Single Dose Regimen of Artefenomel (OZ439) in Loose Combination with Piperaquine Phosphate in Adults and Children with Uncomplicated *Plasmodium falciparum* Malaria.
**Short Title:**	Phase IIb Study to Investigate the Efficacy of OZ439 & PQP Co-administered to Adults & Children with Uncomplicated *P. falciparum* Malaria.
**IND Number:**	104549
**Development Phase:**	Phase IIb
**Final Protocol Date:**	30 Jan 2014
**Amendment 1 Date:**	26 May 2014
**Amendment 2 Date:**	**27** Aug 2014
**Amendment 3 Date:**	07 Jan 2015
**Amendment 4 Date:**	17 Jul 2015

Property of Medicines for Malaria Venture (MMV)
May not be used, divulged or published without the consent of MMV

SIGNATURE PAGE 1 (SPONSOR PROJECT DIRECTOR)

The signature below constitutes approval of this protocol by the signatory and provides the necessary assurances that this study will be conducted according to all stipulations of the protocol including all statements regarding confidentiality.

Signed: F. Macintyre

Fiona Macintyre, PhD
Project Director
Medicines for Malaria Venture (MMV)

Date: 17/07/15

SIGNATURE PAGE 2 (SPONSOR MEDICAL ADVISOR)

The signatures below constitute approval of this protocol by the signatory.

Signed: ______________________________

Stephan Duparc, MD
Chief Medical Officer
Medicines for Malaria Venture (MMV)

Date: 17/07/2015

SIGNATURE PAGE 3 (STATISTICIAN)

The signatures below constitute approval of this protocol by the signatory.

Signed: ______________________________

Ilze Crous
Senior Statistician
Quintiles

Date: 17 Jul 2015

SIGNATURE PAGE 4 (CO-ORDINATING INVESTIGATOR)

The signature below constitutes approval of this protocol by the signatory and provides the necessary assurance that this study will be conducted at his investigational site according to all stipulations of the protocol including all statements regarding confidentiality.

Address of Institution: CERMEL (Centre de Recherches Médicale de Lambaréné), Albert Schweitzer Hospital, Lambaréné, GABON

Signed: Ramharter

Print Name and Title: Assoc. Prof. Dr. Michael Ramharter

Date: 20.07.15

SIGNATURE PAGE 5 (INVESTIGATOR)

The signature of the below constitutes agreement of this protocol by the signatory and provides the necessary assurance that this study will be conducted at his/her investigational site according to all stipulations of the protocol including all statements regarding confidentiality.

Address of Institution: ____

____

____

Signed: ____

Print Name and Title: ____

Date: ______________________

CONTACT LIST

Sponsor

Medicines for Malaria Venture, ICC, 20, route de Pré-Bois, PO Box 1826, 1215 Geneva 15, Switzerland
Tel: +41 22 555 03 00 Fax: +41 22 555 03 69

Sponsor (Project Director)

Dr Fiona Macintyre
Medicines for Malaria Venture, ICC, 20, route de Pré-Bois, PO Box 1826, 1215 Geneva 15, Switzerland
Tel: +41 22 555 03 19
Mob: +41 79 934 39 24
Email: macintyref@mmv.org

Sponsor (Medical Advisor)

Dr Stephan Duparc, MD
Medicines for Malaria Venture, ICC, 20, route de Pré-Bois, PO Box 1826, 1215 Geneva 15, Switzerland
Tel: +41 22 555 03 51
Mob: +41 79 446 29 56
Email: duparcs@mmv.org

Sponsor (Operations Manager)

Dr Helen Demarest,
Medicines for Malaria Venture, ICC, 20, route de Pré-Bois, PO Box 1826, 1215 Geneva 15, Switzerland
Tel: +41 22 555 03 24
Mob: +41 79 914 07 95
Email: demaresth@mmv.org

Co-ordinating Investigator

Assoc. Prof. Dr. Michael Ramharter
Department of Medicin I, Div. of Infectious Diseases and Tropical Medicine
Medical University of Vienna, Währinger Gürtel 18-20, 1090 Vienna, AUSTRIA
and
CERMEL (Centre de Recherches Médicale de Lambaréné)
Albert Schweitzer Hospital, Lambaréné, GABON
Tel.: +43-1-40400-4440
Fax.: +43-1-40400-4418
Email: michael.ramharter@meduniwien.ac.at

Clinical Research Organisation

Quintiles
500 Brook Drive, Green Park, Reading, Berkshire, RG2 6UU, UK
Tel: +44 (0) 118 450 8000

Jamie Moss
Clinical Project Manager
Tel: + 44 (0) 118 450 8478
Email: jamie.moss@quintiles.com

IMPORTANT

INSTRUCTIONS FOR RAPID NOTIFICATION OF SERIOUS ADVERSE EVENTS

A serious adverse event (SAE) is defined as any untoward medical occurrence, that at any dose:

- Results in death;
- Is life-threatening*;
- Requires hospitalisation or prolongation of existing inpatient's hospitalisation;
- Results in persistent or significant disability or incapacity;
- Is a congenital abnormality or birth defect;
- Medical judgement should be exercised in deciding whether an adverse event/reaction is serious in other situations. Important adverse events/reactions that are not immediately life-threatening, or do not result in death or hospitalisation but may jeopardise a patient, or may require intervention to prevent one of the other outcomes listed in the definition above should also be considered serious;
- Is a suspected case of drug induced liver toxicity (Hy's Law)

* 'Life-threatening' in the definition of 'serious' refers to an event in which the patient was at risk of death at the time of the event; it does not refer to an event which hypothetically might have caused death if it were more severe.

Any SAE occurring in a patient receiving Investigational Medicinal Product (IMP) must be reported to the Sponsor via Quintiles Lifecycle Safety within 24 hours, even if the SAE does not appear to be IMP-related. This should be done by faxing the completed SAE Report Form using the contact details below.

Contact Details:

Susanne Riester
Quintiles Lifecycle Safety
Quintiles AG
Hochstr. 50
CH-4053 Basel
Switzerland

Office: + 41 61 270 81 81
Fax: + 41 61 270 81 80
QLS-MMV@quintiles.com

GLOSSARY OF ABBREVIATIONS

ACT	Artemisinin based combination therapies
ACPR	Adequate Clinical and Parasitological Response
AE	Adverse event
ALP	Alkaline phosphatase
ALT	Alanine transaminase
AS	Artesunate
AUC	Area under the plasma concentration time curve
AUC_{inf}	Area under plasma concentration-time curve from zero to infinity
AUC_{0-t}	Area under plasma concentration-time curve from zero to time t of the last measured concentration above the limit of quantification
ß-HCG	Beta-human chorionic g gonadotropin
BLQ	Below level of Quantification
BP	Blood Pressure
BUN	Blood Urea Nitrogen
CBC	Complete Blood Count
Cmax	Maximum Peak Observed Concentration
CNS	Central Nervous System
CRA	Clinical Research Associate
CYP3A4	Cytochrome P450 3A4
DHA	Dihydroaretmisinin
ECG	Electrocardiogram
ETF	Early Treatment Failure
eCRF	electronic Case Report Form
EDTA	Ethylene-diamine-tetraacetic acid
FCT	Fever Clearance Time
FDA	Food and Drug Administration
GCP	Good Clinical Practice
GMP	Good Manufacturing Practice
HAV	Hepatitis A virus
Hb	Haemoglobin
HBsAg	Hepatitis B surface antigen
HCG	Human Chorionic Gonadotropin
HCV	Hepatitis C virus
HIV	Human immunodeficiency virus
HR	Heart rate
ICH	International Conference on Harmonisation
IEC	Independent Ethics Committee
IMP	Investigational medicinal product
IRB	Institutional Review Board
ISMB	Independent Safety Monitoring Board
ITT	Intent to Treat
LAR	Legally Acceptable Representative (individual or juridical or other body authorised under applicable law to consent, on behalf of a prospective patient, to the patient's participation in a clinical study)
LCF	Late Clinical Failure
LPF	Late Parasitological Failure
MCHC	Mean cell haemoglobin concentration
MCM	Malaria Challenge Model

MCV	Mean cell volume
MPC	Minimum Parasiticidal Concentration
MQ	Mefloquine
MMV	Medicines for Malaria Venture
PCR	Polymerase Chain Reaction
PCT	Parasite Clearance Time
PD	Pharmacodynamic
Pf	*Plasmodium falciparum, P. falciparum*
PI	Principal Investigator
PIB	Powder In Bottle
PK	Pharmacokinetic
PP	Per Protocol
PoC	Proof of Concept
PQP	Piperaquine Phosphate
PQ	Piperaquine
PRR	Parasite Reduction Rate
Pv	*Plasmodium vivax, P. vivax*
qPCR	Quantitative Polymerase Chain Reaction
QTcB	QTc interval Bazett's correction
QTcF	QTcF interval Fridericia's correction
QTcW	QTcF interval Wernicke's correction
RDT	Rapid diagnostic test for malaria
RT-PCR	Reverse-transcriptase Polymerase Chain Reaction
SAE	Serious adverse event
SAP	Statistical analysis plan
SOP	Standard operating procedure
SUSAR	Suspected unexpected serious adverse reaction
TPGS	alpha tocopherol polyethylene glycol 1000 succinate
t½	Half-life of elimination
ULN	Upper Limit of Normal
WBC	White Blood Cell
WHO	World Health Organisation

GLOSSARY OF TERMS

qPCR
A polymerase chain reaction procedure is used to quantify the amount of *P falciparum*-specific DNA present and is transformed into number of parasites/µL. Where the presence of gametocytes is suspected the sample can be tested for the presence of Psf25 transcripts using an RT-PCR method or the sample can be concentrated for detection by microscopy.

Re-emergence/Recurrence
Re-emergence (recrudescence and re-infection) is defined as the appearance of asexual parasites after clearance of initial infection irrespective of genotype. Recrudescence or re-infection must be confirmed by microscopy (positive blood smear) and PCR analysis.

Recrudescence

Recrudescence is defined as the appearance of asexual parasites after clearance of initial infection with a genotype identical to that of parasites present at baseline. Recrudescence must be confirmed by microscopy (positive blood smear) and PCR analysis.

Re-infection

Re-infection is defined as the appearance of asexual parasites after clearance of initial infection with a genotype that differs from that of parasites present at baseline. Re-infection must be confirmed by microscopy (positive blood smear) and PCR analysis. Confirmed new infection will not be regarded as treatment failure or recrudescence.

Outcome Classification

Treatment outcome is established according to a modified standard WHO classification:

Early treatment failure (ETF)

- danger signs or severe malaria on day 1, 2 or 3, in the presence of parasitaemia;•
- parasitaemia on day 2 higher than on day 0, irrespective of axillary temperature;•
- parasitaemia on day 3 with axillary temperature ≥ 37.5 °C; and
- parasitaemia on day 3 ≥ 25% of count on day 0.

Late clinical failure (LCF)

- danger signs or severe malaria in the presence of parasitaemia on any day between day 4 and day 28 (day 42) in patients who did not previously meet any of the criteria of early treatment failure; and
- presence of parasitaemia on any day between day 4 and day 28 (day 42) with axillary temperature ≥ 37.5 °C (or history of fever) in patients who did not previously meet any of the criteria of early treatment failure.
- At 96 hours (day 4) post dose: failure to achieve parasite clearance irrespective of axillary temperature in patients who did not previously meet any of the criteria of ETF.

Late parasitological failure (LPF)

- presence of parasitaemia on any day between day 7 and day 28 (day 42) with axillary temperature < 37.5 °C in patients who did not previously meet any of the criteria of early treatment failure or late clinical failure.

Adequate clinical and parasitological response (ACPR)

- absence of parasitaemia on day 28 (day 42), irrespective of axillary temperature, in patients who did not previously meet any of the criteria of early treatment failure, late clinical failure or late parasitological failure

TABLE OF CONTENTS

Page

TITLE PAGE 1
SIGNATURE PAGES 2
CONTACT LIST 7
INSTRUCTIONS FOR RAPID NOTIFICATION OF SERIOUS ADVERSE EVENTS 9
GLOSSARY OF ABBREVIATIONS 10
GLOSSARY OF TERMS 11
TABLE OF CONTENTS 13

1. STUDY SYNOPSIS 17
2. SCHEDULE OF ASSESSMENTS: SCREENING TO DAY 63 26
3. BACKGROUND 30
3.1. INTRODUCTION 30
3.2. CLINICAL DATA 32
3.3. PK/PD SUMMARY 40
3.4. TOLERABILITY SUMMARY 41
3.5. STUDY RATIONALE 42
3.6. STUDY POPULATION RATIONALE 43
3.7. DOSE RATIONALE 43
4. STUDY OBJECTIVES 44
4.1 PRIMARY OBJECTIVE 44
4.2 SECONDARY AND EXPLORATORY OBJECTIVES 44
5 OVERALL STUDY DESIGN 45
5.1 STUDY DESIGN 45
5.2 STUDY PLAN 46
5.3 PRE-DOSE ACTIVITIES AND ASSESSMENTS 46
5.3.1 Informed Consent 46
5.3.2 Screening 47
5.3.3 Pre-dose Assessments 47
5.4 POST-DOSE ACTIVITIES AND ASSESSMENTS 48
5.4.1 Treatment 48
5.4.2 Assessments post-dose up to and including Day 14 48
5.4.3 Assessments between Day 14 up to and including Day 28 48
5.4.4 Assessments on Day 42 (and Day 63 at selected sites) 49
5.5 RE-DOSING IN THE EVENT OF VOMITING 49
5.6 DISCHARGE CRITERIA 49
5.7 RE-ADMISSION CRITERIA 50
5.8 CIRCUMSTANCES FOR ESTABLISHED ANTI-MALARIA TREATMENT 50
5.9 BLINDING AND RANDOMISATION 51
5.10 ENDPOINT 51
5.10.1 Primary Efficacy Endpoint 51
5.10.2 SECONDARY AND EXPLORATORY ENDPOINTS 51
6 SELECTION OF PATIENTS 53
6.1 NUMBER OF PATIENTS 53
6.2 RECRUITMENT METHODS 53

6.3 INCLUSION CRITERIA 53
6.4 EXCLUSION CRITERIA 53
6.5 PATIENT POPULATION 55
6.5.1 Patient Sub-populations 55
6.5.2 Age Range Step-down Procedure 56
6.6 PATIENT RESTRICTIONS 56
6.7 CONTRACEPTIVE REQUIREMENTS FOR MALE AND FEMALE PATIENTS56

7 PATIENT ASSESSMENTS 56

7.1 DEMOGRAPHIC AND BASELINE VARIABLES 56
7.2 MEDICAL HISTORY 57
7.3 PRIOR MEDICATION 57
7.4 CONCOMITANT MEDICATION 57
7.5 BODY TEMPERATURE 58
7.6 VITAL SIGNS (BLOOD PRESSURE / PULSE RATE) 59
7.7 PARASITAEMIA (THICK AND THIN BLOOD FILMS) 59
7.7.1 Screening and Pre-dose Blood Films 59
7.7.2 Post-dose Blood Films 60
7.8 DRY BLOOD SPOT SAMPLES FOR QUANTITATIVE PCR, PARASITE GENOTYPING (PCR CORRECTION) AND ANALYSIS OF PARASITE GENOTYPES OF INTEREST THAT MAY BE ASSOCIATED WITH DRUG RESISTANCE 61
7.9 GAMETOCYTE DETECTION (RT-PCR) 61
7.10 MALARIA RAPID DIAGNOSTIC TEST 62
7.11 PHYSICAL EXAMINATION AND MALARIA SIGNS AND SYMPTOMS 62
7.12 12-LEAD ECG 62
7.13 LABORATORY SAFETY EVALUATIONS 63
7.13.1 Pregnancy Test 65
7.13.2 FSH 65
7.14 PHARMACOKINETICS 65
7.15 BLOOD SAMPLE VOLUMES 66
7.16 ADVERSE EVENTS (AE) 66
7.16.1 Adverse Event Description 66
7.16.2 Severity of Adverse Events 67
7.16.3 Causality of Adverse Events 67
7.16.5 Serious Adverse Events 69

8 INTERIM ASSESSMENTS 69

8.1 SAFETY EVALUATIONS FOR INDEPENDENT SAFETY MONITORING BOARD (ISMB) 69
8.2 INTERIM EXTRACTION OF PK/PD DATA 70
8.3 EFFICACY/FUTILITY INTERIM ANALYSES 70

9 PATIENT FOLLOW UP IN THE EVENT OF HEPATIC ENZYME ABNORMALITIES 70

9.1 CIRCUMSTANCE REQUIRING CLOSE MONITORING OF HEPATIC SAFETY 70
9.2 PREMATURE DISCONTINUATION OF INDIVIDUAL PATIENTS 71

10 TREATMENT 72

10.1 INVESTIGATIONAL MEDICINAL PRODUCT DETAILS 72
10.2 LABELLING, STORAGE AND DISPENSING 72
10.3 DOSAGE, DURATION AND COMPLIANCE 73
10.4 ACCOUNTABILITY 75
10.5 CODE-BREAKING 76

10.6 TREATMENT OF OVERDOSE 76

11 STATISTICAL METHODS AND DATA MANAGEMENT 76

11.1 STATISTICAL ANALYSIS PLAN 76
11.2 ANALYSIS SETS 77
11.3 SAMPLE SIZE 78
11.4 DATA ANALYSIS 79
11.5 MISSING, UNUSED AND SPURIOUS DATA 80
11.6 STATISTICAL CRITERIA FOR TREATMENT ARM AND STUDY TERMINATION 80
11.7 BASELINE CHARACTERISTICS 80

***MEDICAL HISTORY* 81**

***CONCOMITANT MEDICATIONS* 81**

***STUDY DRUG EXPOSURE AND COMPLIANCE* 81**

11.8 EFFICACY ANALYSIS 81
11.8.1 Primary efficacy analysis 81
11.8.2 Secondary Endpoints 82
11.8.2.1 Efficacy Analysis 82
PARASITE REDUCTION RATE (PRR) 83
11.8.2.2 Exploratory Analysis 84
11.8.2.3 Pharmacokinetic Analysis 85
11.9 SAFETY ANALYSIS 86
11.10 DATA MANAGEMENT 88

12 REGULATORY, ETHICAL AND LEGAL OBLIGATIONS 88

12.1 DECLARATION OF HELSINKI 88
12.2 GOOD CLINICAL PRACTICE 88
12.3 INDEPENDENT ETHICS COMMITTEE/INSTITUTIONAL REVIEW BOARD APPROVAL 88
12.3.1 Initial Approval 88
12.3.2 Approval of Amendments 88
12.3.3 Reporting of Suspected Unexpected Serious Adverse Reactions 89
12.3.4 Periodic Safety Reports and End of Study Notification 89
12.4 REGULATORY AUTHORITY APPROVAL 89
12.5 OTHER REQUIRED APPROVALS 89
12.6 INSURANCE 89
12.7 PRE-STUDY DOCUMENTATION REQUIREMENTS 89
12.8 INFORMED CONSENT 90
12.9 CONTACT WITH GENERAL PRACTITIONER/PRIMARY CARE PHYSICIAN90
12.10 PATIENT CONFIDENTIALITY 90
12.11 DATA PROTECTION 90
12.12 END OF STUDY 90
12.13 STUDY DOCUMENTATION AND DATA STORAGE 90
12.14 DISCLOSURE OF INFORMATION 91
12.15 PUBLICATION 91

13 ADMINISTRATIVE OBLIGATIONS 91

13.1 SOURCE DATA 91
13.2 LANGUAGE 92
13.3 DATA COLLECTION 92
13.4 MONITORING 92
13.5 QUALITY CONTROL AND QUALITY ASSURANCE 92

14 REFERENCES 93

ATTACHMENT 1: TOXICITY GRADING SCALES FOR DETERMINING SEVERITY OF ADVERSE EVENTS 94

ATTACHMENT 2: HY'S LAW PROCEDURES: 116

ATTACHMENT 3: SEVERE FALCIPARUM MALARIA 119

Tables

Table 1: Studies to Date: OZ439 or PQP alone, OZ439/PQP loose Combination 33
Table 2: Key Studies - Drug Exposures OZ439 and OZ439 / PQP 34
Table 3: Clinical Laboratory Blood Tests 64
Table 4 Blood Sample Volumes 66
Table 5: Treatments and Dosage 73

Figures

Step-down procedure 29

1. STUDY SYNOPSIS

Product:	OZ439 and Piperaquine phosphate (PQP)
Study Title:	A Randomised, Double-blind, Phase IIb Study to Investigate the Efficacy, Safety, Tolerability and Pharmacokinetics of a Single Dose Regimen of Artefenomel (OZ439) in Loose Combination with Piperaquine Phosphate in Adults and Children with Uncomplicated *Plasmodium falciparum* Malaria.
Short Title:	Phase IIb Study to Investigate the Efficacy of OZ439 & PQP Co-administered to Adults & Children with Uncomplicated *P. falciparum* Malaria.
Development Phase:	Phase IIb
Objectives:	Primary Objective<br>• To determine whether a single dose combination of OZ439/PQP is an efficacious treatment for uncomplicated *P. falciparum* malaria in adults and children<br><br>Secondary and Exploratory Objectives<br>• To evaluate the efficacy of OZ439/PQP:<br>o To determine the incidence of recrudescence and re-infection<br>o To determine the time to relief of fever and parasite clearance<br>• To further explore efficacy of OZ439/PQP:<br>o To evaluate the proportion of patients with gametocytes at each assessment<br>o To characterise gametocyte carriage<br>o To examine the relationship between ACPR and exposure to OZ439/PQP (using logistic regression)<br>o To examine the relationship between Kelch-13 genotype and additional parasite genotypes of interest that may be identified and parasite clearance kinetics/efficacy<br>• To evaluate the pharmacokinetics of OZ439/PQP:<br>o To determine Cmax, Tmax and AUC of OZ439 and PQP in patients ≥35 kg<br>o To characterise the pharmacokinetics and potential covariates in all patients (using population PK analysis)<br>• To evaluate the safety and tolerability of OZ439/PQP
Design:	A randomised, double-blind single-dose (loose combination) study in patients with uncomplicated *Plasmodium falciparum* malaria. The study will test for efficacy/futility through analyses, using Bayesian methodology. Adults and children will be included through progressive step-down in age following safety analyses.
Inclusion Criteria:	1. Male or female patient age ≥6 months <70 years<br>2. Body weight ≥5 kg ≤90 kg

	3. Presence of mono-infection of *P. falciparum* with:<br>a. Fever, as defined by axillary temperature ≥ 37.5°C or oral/rectal/tympanic temperature ≥ 38°C, or history of fever in the previous 24 hours (history of fever must be documented) and,<br>b. Microscopically confirmed parasite infection, in range 1,000 to 100,000 asexual parasites /µL of blood<br>4. Written informed consent provided by the adult patient, or parent or legally acceptable representative (LAR) of the minor patient or by an impartial witness (if the patient or patient's LAR is illiterate), stating that the information has been read and/or is understood, and by the medically qualified Investigator. Children will be asked to provide assent where appropriate. The age from which this will be sought will be defined by local legislation.
Exclusion Criteria:	1. Presence of severe malaria (according to WHO definition – WHO 2013)<br>2. Anti-malarial treatment:<br>a) With piperaquine -based compound, mefloquine, naphthoquine or sulphadoxine/pyrimethamine (SP) within the previous 6 weeks (after their inhibition of new infections has fallen below 50%).<br>b) With amodiaquine or chloroquine within the previous 4 weeks.<br>c) With quinine, halofantrine, lumefantrine, artemisinin-based compounds and any other anti-malarial treatment or antibiotics with antimalarial activity (including cotrimoxazole, tetracyclines, quinolones and fluoroquinolones, and azithromycin) within the past 14 days.<br><br>For other concomitant medication restrictions see Section 7.4<br><br>3. Known history or evidence of clinically significant disorders such as cardiovascular (see 4, 5, 6 & 7 below), respiratory (including active tuberculosis), hepatic, renal, gastrointestinal, immunological (including active HIV-AIDS), neurological (including auditory), endocrine, infectious, malignancy, psychiatric, history of convulsions or other abnormality

	(including head trauma).<br>4. Family history of sudden death or of congenital or clinical conditions known to prolong QTcB or QTcF interval or e.g. family history of symptomatic cardiac arrhythmias, with clinically relevant bradycardia or severe cardiac disease.<br>5. History of symptomatic cardiac arrhythmias or with clinically relevant bradycardia or with severe cardiac disease.<br>6. Any predisposing cardiac conditions for arrhythmia such as severe hypertension, left ventricular hypertrophy (including hypertrophic cardiomyopathy) or congestive cardiac failure accompanied by reduced left ventricle ejection fraction.<br>7. QTcB or QTcF >450ms at Screening (Note patients with QTcB or QTcF >450ms pre-dose should be withdrawn prior to dosing)<br>8. Electrolyte disturbances, particularly hypokalaemia, hypocalcaemia or hypomagnesaemia.<br>9. Any treatment which can induce a lengthening of QT interval, such as:<br>a. Antiarrhythmics (e.g. amiodarone, disopyramide, dofetilide, ibutilide, procainamide, quinidine, hydroquinidine, sotalol),<br>b. Neuroleptics (e.g. phenothiazines, sertindole, sultopride, chlorpromazine, haloperidol, mesoridazine, pimozide, or thioridazine),<br>c. Anti-depressive agents, certain antimicrobial agents, including agents of the following classes macrolides (e.g. erythromycin, clarithromycin), fluoroquinolones (e.g. moxifloxacin, sparfloxacin), imidazole and triazole antifungal agents, and also pentamidine and saquinavir,<br>d. Certain non-sedating antihistamines (e.g. terfenadine, astemizole, mizolastine), cisapride, droperidol, domperidone, bepridil, diphemanil, probucol, levomethadyl, methadone, vinca alkaloids, arsenic trioxide.<br>e. Anti-emetics with known QT prolongation potential such as domperidone<br>10. Mixed *Plasmodium* infection<br>11. Severe vomiting, defined as more than three times in the 24 hours prior to inclusion in the study or inability to tolerate oral treatment, or severe diarrhoea defined as 3 or more watery stools per day

	12. Severe malnutrition (defined for subjects aged ten years or less as the weight-for-height being below -3 standard deviation or less than 70% of median of the NCHS/WHO normalised reference values, and for subjects aged greater than ten years, a body mass index (BMI) of less than 16 (WFP Manual, Chapter 1)).<br>13. Known history of hypersensitivity, allergic or adverse reactions to piperaquine or other aminoquinolones or to OZ439 or OZ277<br>14. Known active Hepatitis A IgM (HAV-IgM), Hepatitis B surface antigen (HBsAg) or Hepatitis C antibody (HCV Ab).<br>15. If Total Bilirubin is normal, exclude the patient if liver function tests AST/ALT ≥ 2xULN.<br>16. If Total Bilirubin is > 1 and ≤ 1.5xULN, exclude the patient if AST/ALT >1.5xULN.<br>17. Total Bilirubin > 1.5xULN<br>18. Hb level below 8 g/dL.<br>19. Serum creatinine levels equal to or more than 2xULN<br>20. Female patients of child bearing potential must be neither pregnant (as demonstrated by a negative pregnancy test) nor lactating, and must be willing to take measures not to become pregnant during the study period and safety follow-up period.<br>21. Have received an investigational drug within the past 4 weeks.<br>22. Previous participation in any malaria vaccine study or received malaria vaccine in any other circumstance.<br>23. Refusal to participate and to provide written or witnessed informed consent or assent
Sample Size:	The study will be performed using interim assessments of efficacy / futility. There will be three treatment arms. The target maximum number of patients recruited will be 165 per treatment arm consisting of 150 patients to be included in the interim analyses for efficacy/ futility (i.e. Asian/Latin American patients and African patients ≤ 5 years) and an additional 15 African patients > 5 years which will not be included in the interim analyses for efficacy/ futility. Recruitment would be capped at 150 + 15 patients per treatment arm.
Efficacy/ Futility Analyses:	Analyses will be performed for efficacy / futility on a regular basis. At the early stages of the study, recruitment will continue during these analyses, however during later analyses, recruitment may be paused dependent on recruitment rate at the time. Recruitment of sub-populations will be stopped when the pre-determined numbers are reached.

	The study will follow a group sequential design with up to a total of 6 analyses (5 interim analyses) based on the predictive probability methodology of Lee and Liu (2008).<br>At each interim analysis, the treatment arm can be stopped for futility if the posterior probability of $\boldsymbol{H_0}$, given the data accumulated at the look for the treatment arm in question is too large, $\mathbf{Pr}(\boldsymbol{H_0}\|\ \boldsymbol{data}) \geq \mathbf{0.3},$ can be is stopped for efficacy if the posterior probability of $\boldsymbol{H_1}$ given the data accumulated at the look for the treatment arm in question is large, $\mathbf{Pr}(\boldsymbol{H_1}\|\ \boldsymbol{data}) \geq \mathbf{0.95},$ or can be advanced to the next stage if neither of the above conditions are met.<br><br>The procedure's operating characteristics were evaluated through simulations for a range of plausible scenarios for the true value of *p* (ranging from 0.85 to 0.98) and the maximum sample size was chosen to correspond to the most pessimistic of the aforementioned scenarios (yielding a maximum sample size of 208 per treatment arm, and corresponding average sample size of 104 per arm).<br><br>Interim assessment of efficacy or futility will occur after recruitment of approximately 50 evaluable patients per dose cohort, further interim analyses may be performed, the timing of which will depend on the recruitment rate.<br><br>The Independent Safety Monitoring Board (ISMB) can request considerations of an earlier first efficacy/ futility interim analysis (i.e. at <50 evaluable patients per treatment arm) if there is evidence of possible high treatment failure based on the data reviewed by the ISMB e.g frequency and timing of rescue medication administration.<br><br>These outcomes, taken with numbers used in prior malaria combination studies (106 per cohort), indicated that recruitment of 120 to 150 patients per treatment arm would be prudent. If the combination is efficacious, 120 per arm should suffice. 150 per arm provides an upper limit of recruitment when results are consistently between the futility and success criteria and represent when a ACPR28 can be calculated and taken forward acknowledging that the value is neither predictive of clear futility or predictive of overwhelming efficacy (Lee, J. L. and Liu, D. D., 2008).
Patient Population:	<u>*Lower immunity population*</u><br>This is the primary population of interest.

	Patients with lower potential for immunity i.e. Asia/Latin America: all age groups and Africa: younger children (less than or equal to 5 years).<br>*Higher immunity population*<br>Patients with higher potential for immunity i.e. African patients > 5 years to < 70 years.<br><br>The PK/PD (efficacy) exposure-response relationship will be assessed only in the Lower immunity population of interest i.e. Asian/Latin American population and African patients less than or equal to 5 years of age. However all patients will be assessed for safety.
Age-range and Step-down Procedure (ISMB):	Safety in the older age range (Asian, Latin American and African) will be assessed before proceeding down to the younger age range (see Figure 1)<br>o 30 patients (> 15 years) will be assessed for safety before opening recruitment in the > 5 and ≤ 15 year age range<br>o 20 additional patients (> 5 to ≤ 15 years) will be assessed for safety before opening recruitment in the > 2 and ≤ 5 year age range<br>o 20 additional patients (> 2 to ≤ 5 years) will be assessed for safety before opening recruitment in the ≥ 6 month to ≤ 2 year age range<br>The Safety evaluation will be performed by an Independent Safety Monitoring Board (ISMB).<br>.
Interim Extraction of PK/PD Data	At approximately the same time as the first interim assessment for efficacy / futility, a preliminary PK/PD analysis of the study data will be performed to assist clinical development. This additional modelling verification will not influence the conduct of the study.
Treatments groups:	Patients ≥35kg<br>A) OZ439 800 mg: PQP 1440 mg<br>B) OZ439 800 mg: PQP 960 mg<br>C) OZ439 800 mg: PQP 640 mg<br>Patients < 35kg will receive weight-adjusted doses predicted to achieve similar exposure ranges to patients ≥ 35 kg

Study Duration:	Patients will be admitted to the Clinical Unit for Screening and if eligible and give Informed Consent and/or Assent (as applicable) will be recruited to the study. Following drug administration, patients will remain in the Clinical Unit for a minimum of 48 or 72 hours (depending on region, age, parasitaemia and temperature). Patients may remain in the Clinical Unit until Day 7 procedures are completed if more convenient. The overall duration of the study (Screening to final assessment) will range from 42 days to 63 days depending on whether recruited to a site carrying out assessments to Day 42 or Day 63. The number of visits will range from approximately 9 to 12 depending on time of discharge and whether the patient returns to the Clinical Unit for assessments during Intervening periods or has assessments 'remote from the Clinical Unit'.
Efficacy/ Pharmaco-dynamic Assessments:	Blood films (thick and thin) and axillary temperature (single):<br>Screening/pre-dose, 6, 12, 18, 24, 30, 36, 48, 72 hours, Days 5, 7, 10, 14, 21, 28, 42, and 63 (selected sites only) Rapid diagnostic tests may be employed between Days 14 to 21, and 21 to 28.<br>A reduced number of assessments will be taken from patients <35kg
Pharmaco-kinetic Assessments:	Non-compartmental analysis (as data allows, ≥35 kg)<br>Nonlinear mixed effect modeling (all patients)
Safety Assessments:	Haematology, including haemolysis, clinical chemistry and urinalysis, physical examination, 12-lead ECG (triple), vital signs (single), adverse events
Primary Efficacy Endpoint:	PCR-adjusted adequate clinical and parasitological response (ACPR) at Day 28.
Secondary & Exploratory Efficacy Endpoints:	Efficacy Endpoints<br>o PCR - adjusted ACPR at Day 42 and 63*.<br>o PCR - crude ACPR at Day 28, 42 and 63*.<br>o Kaplan Meier analysis presentation for incidence rate of 1) re-emergence, 2) recrudescence and 3) re-infection at Day 28, 42 and 63*.<br>o Parasite clearance time (PCT).<br>o Fever clearance time (FCT).<br>o PRR.<br>Exploratory Endpoints<br>o Kaplan-Meier presentation of the risk of having gametocytes for:<br>- Patients with gametocytes at baseline to time to clearance of gametocytes.<br>- Patients with no gametocytes at baseline to time to appearance of gametocytes.<br>o Integrated number of gametocytes (AUC) at 28 and 42 days for (pre-specified endpoints calculated and reported outside of CSR):<br>- Patients with gametocytes at baseline.<br>- Patients with no gametocytes at baseline that develop gametocytes during the study.<br>o Correlation between response (ACPR at Day 28 and 42) and

	exposure (Day 7) to OZ439/PQP<br>o Correlation between Kelch-13 genotype status and additional parasite genotypes of interest that may be identified, and parasite clearance kinetics/efficacy.<br>o Exposure response evaluation including pharmacokinetic / pharmacodynamics modelling.
PK Endpoints:	o Non-compartmental analysis (NCA) of concentrations of OZ439 and piperaquine:<br>- Performed on patients whose PK data describe the Cmax and elimination phase**.<br>- Parameters are: Cmax, Tmax, AUC, CL/f, elimination half-life ($t_{1/2}$)**.<br>- Concentration at Day 7, 14, 28, 42 and 63* (summarised from patients who have those time points sampled)**.<br>o Non-linear mixed effect modelling (pre-specified endpoints calculated and reported outside of CSR)<br>- Performed across all patients.<br>- An extension of existing PK models - where weight is a dosing covariate**.**<br>- Including analysis of covariates for allometric scaling by weight, gender, disease, ethnicity, site effects and others if deemed relevant.
Safety Endpoints	o Incidence of adverse events.<br>o Laboratory variables including change from baseline.<br>o Haemoglobin drop<br>- Hb drop > 2 g/dL from baseline<br>- Hb ≤ 5g/dL<br>o Absolute Neutrophil count < 1000/µL<br>o Proportion of patients meeting the Hy's law definition (see Section 9.1).<br>o LFT changes:<br>- Any ALT or AST ≥5x ULN<br>- Any AST or ALT ≥3x ULN with the appearance of fatigue, nausea, vomiting, right upper quadrant pain or tenderness, fever, rash and/or eosinophilia (eosinophil percent or count above the ULN)<br>- ALT ≥3x ULN persisted for >4 weeks<br>o Clinically significant ECG abnormalities including incidence of QT/QTc as follows:<br>- QT/QTc < 450 ms<br>- 450 ms ≤ QT/QTc < 480 ms<br>- 480 ms ≤ QT/QTc < 500 ms<br>- QT/QTc ≥ 500 ms.<br>And change from baseline in QT/QTc:<br>- < 30 ms increase from baseline.

	- ≥ 30 ms and < 60 ms increase from baseline.<br>- ≥ 60 ms increase from baseline.<br>o Vital signs including change from baseline.<br>o Physical examination and clinical signs and symptoms related to uncomplicated *P. falciparum* malaria (Fever, Dizziness, Headache, Nausea, Anorexia, Vomiting, Diarrhoea, Itching, Urticaria, Skin Rash, Abdominal Pain, Joint Pain, Muscle Pain, Palpitations, Sleep Problems, Confusion, Hearing Problems, Vision Problems, Fatigue).<br><br>** Data at Day 63 collected at selected sites only.*<br>*** Patients <35kg will have 'sparse sampling' and may have insufficient data.*
Statistical Methods:	For Interim Analysis: An ongoing evaluation will be performed at each efficacy/futility interim analysis calculating the **posterior** probability that PCR-adjusted ACPR28>95% or ACPR28<90%<br><br>For the Final Analysis: A final binary assessment will be made across the PP analysis set where the proportion of lower immunity patients successfully treated by Day 28 will be calculated. This will be stratified by treatment arm. Complete details will be available in the SAP prior to database lock.

2. SCHEDULE OF ASSESSMENTS: SCREENING TO DAY 63

Day [b]		0							1			2[a]	3[a]	5[a]	7[a]	10	14	15-20[h]	21	22-27[h]	28	42	63 [m]	Un-scheduled [n]
Hours **post-dose (measured from start of dosing)**	Screen / Pre-dose	0	1	2	4	6	12	18	24	30	36	48	72											
Informed consent	X																							
Inclusion/ exclusion criteria	X																							
Physical exam & malaria signs & symptoms	X						X		X		X	X	X		X						X	X	X	X
Demography, medical history	X																							
Prior and concomitant medication	X		→																					→
Pregnancy test [c]	X																				X			
FSH (women potentially menopausal)	X																							
Clinical laboratory safety [d]	X											X	X	X	X		X				X			(X)
Asexual & gametocyte parasite count (thick & thin blood films) ≥35kg [f,]<br>Patients <35kg (see notes) [f,]	X [e]					X	X	X	X	X	X	X	X	X	X	X	X	(X)	X	(X)	X	X	X	X
Blood spot (for qPCR and parasite genotyping [i] and [p]) for patients ≥ 35 kg [f, g]<br>Patients <35kg (see notes) [f, g]	X					X	X	X	X	X	X	X	X	X	X	X	X	(X)	X	(X)	X	X	X	X
Blood sample for RT-PCR gametocyte detection (for patients ≥ 35 kg only) [f, g]	X					X	X	X	X	X	X	X	X		X									
Malaria RDT (taken 'in field') [h]																		(X)		(X)				
12-Lead ECG (triplicate) [j]	X			X		X	X		X			(X)	(X)		X									(X)
Vital signs (single) [j]	X			X		X	X		X			(X)	(X)		X		X		X		X	X	X	X
Temperature (single) [f,]	X [h]		X	X		X	X	X	X	X	X	X	X	X	X	X	X	X	X	X	X	X	X	X
PK sampling for patients ≥ 35 kg [k]	X			X	X	X	X		X			X	X	X	X	X	X		X		X	X	X	X
Dosing (OZ439 and PQP) [l]		X																						
AEs	→																						→	

[a] Discharge at 48 or 72 hours (or up to Day 7) depending on age, parasite and fever clearance, clinical judgment and patient convenience. If discharged prior to Day 7, will return to Clinical Unit for further assessment on all scheduled times up to Day 7.

[b] At each patient contact post dose, circumstances for established anti-malarial treatment (see section 5.8) will be assessed.

[c] The patient´s menstrual and contraceptive history will be taken, and a test for the presence of HCG will be performed at Screening, to exclude pregnancy. Where feasible a quantitative serum HCG will be performed, failing which a qualitative serum HCG will be performed, failing which a qualitative urine HCG will be performed; a urine HCG is the minimum acceptable test. Result must be confirmed negative prior to dosing.

[d] Laboratory safety: haematology, including haemolysis, clinical chemistry, and urinalysis (See Table 3 for details). For children ≤5 years: Screening (baseline) and Day 2, 7, 14 and 28. D3 should be done if results are abnormal at D2, and D5 should be done if results are abnormal on D3.

[e] Measurement required within 4 hours prior to dosing

[f] Blood films (thick and thin) and temperature measurements need to be confirmed as follows: when 1st parasite clearance and 1st temperature <37.5 °C, measurements need to be confirmed with second reading 6 to 12 hours after the 1st measurement (i.e. to determine Parasite Clearance and Fever Clearance). The first measurement (if confirmed) will be considered the 'Clearance Time'. Patients <35kg: samples for blood films will be taken at Screening/pre-dose, 6, 18, 36, 48 and 72 hours only; thereafter the same as adult schedule.

Additional temperature recordings and blood films may therefore be taken in order to confirm fever and parasite clearance and reported using the unscheduled visit form..

Axillary temperature should be recorded, if the axillary method is not possible, an alternative route (oral, tympanic, rectal) may be used. Within an individual patient the same method of temperature measure should be used throughout the study.

[g] All blood spot samples will be taken at the same time points as the blood films for patients ≥35kg and patients <35kg. Dry blood spot samples (for qPCR in patients ≥ 35kg only; parasite genotyping (PCR correction), Kelch-13 and other parasite genotype of interest analysis on all weights of patient) will be applied to the same card according to the schedule and the instructions within the Study Procedures Manual. qPCR analysis will be performed on the samples taken in the first 72 hours from patients ≥ 35kg. Blood samples for RT-PCR gametocyte detection will be taken into EDTA coated microtainers for patients ≥ 35kg only at each scheduled timepoint up to and including 72 hours, and at Day 7.

[h] Patients will have one additional safety check in the period between Days 14 and 21, and between Days 21 and 28. Patients may either return to the Clinical Unit or tests can be performed in the field by trained persons. If returning to the Clinical Unit, a blood film (thick and thin) and genotyping sample and temperature should be taken. For patients remaining in the field, a Malaria RDT and temperature should be taken. Patients feeling unwell, with increased temperature (axillary temperature ≥37.5 °C) and/ or a positive RDT should return to the Clinical Unit for assessment (see below, note [n]) .

[i] Genotyping (PCR correction) will be performed on previously collected blood spot samples only in case of a positive blood film after initial parasite clearance: One pre-dose sample, and one sample at 18 or 24 hours post-dosing. A further sample will be analysed at the time point at which recrudescence/ re-infection occurs (if applicable).

[j] Patients should rest supine prior to measurement for a minimum of 10 minutes. Single ECG to be taken at screening. Thereafter Triplicate ECGs (measured within 5 minutes of 1st measurement). Triplicate ECG and single vital signs to be taken at discharge (48 or 72 hours or at Investigator's discretion if later) and according to the Schedule of Assessment

[k] PK samples should be taken by venepuncture. Where required, PK sampling should be performed after ECG, vital signs and temperature measurement to ensure that physiological measurements are not taken within 10 minutes of venepuncture or finger-prick.. PK sample should be taken at the nominal time.

PK sampling schedule applies to patients ≥ 35 kg. For patients <35kg, see below:

Patients ≥5 to < 10 kg 1 sample within 24 hours of dosing, 1 sample at Day 7 and 1 additional sample from 24 hours post-dose to Day 28

Patients ≥10 to <20 kg: 2 samples within 24 hours of dosing, 1 sample at Day 7 and 3 additional samples from 24 hours post-dose to Day 28

Patients ≥20 to < 35 kg: 4 samples within 24 hours of dosing, 1 sample at Day 7 and 5 additional samples from 24 hours post-dose to Day 28

In addition, for all patients where possible, a PK sample should be obtained when recrudescence / re-infection occurs.

Sampling times should be accurately recorded in the eCRF and source documents.

[l] Dosing following minimum 3 hours fasting.

[m] Day 63 at selected sites only

[n] Unscheduled visits should be completed if a patient is readmitted at any time during the study with elevated temperature ($\geq$ 37.5 ^{o}C)/positive RDT/feels unwell. Assessments to measure parasitaemia should be taken as scheduled and at the Investigator's discretion until parasite clearance or rescue medication (if required). Safety assessments should be performed at the Investigator's discretion.

[p] Analysis of parasite genotypes of interest associated with drug resistance will be carried out on pre-dose blood spot samples. If insufficient bloodspot samples available at baseline, alternative blood spots collected as specified in the schedule of assessments may be used.

Step-down procedure (Figure 1)

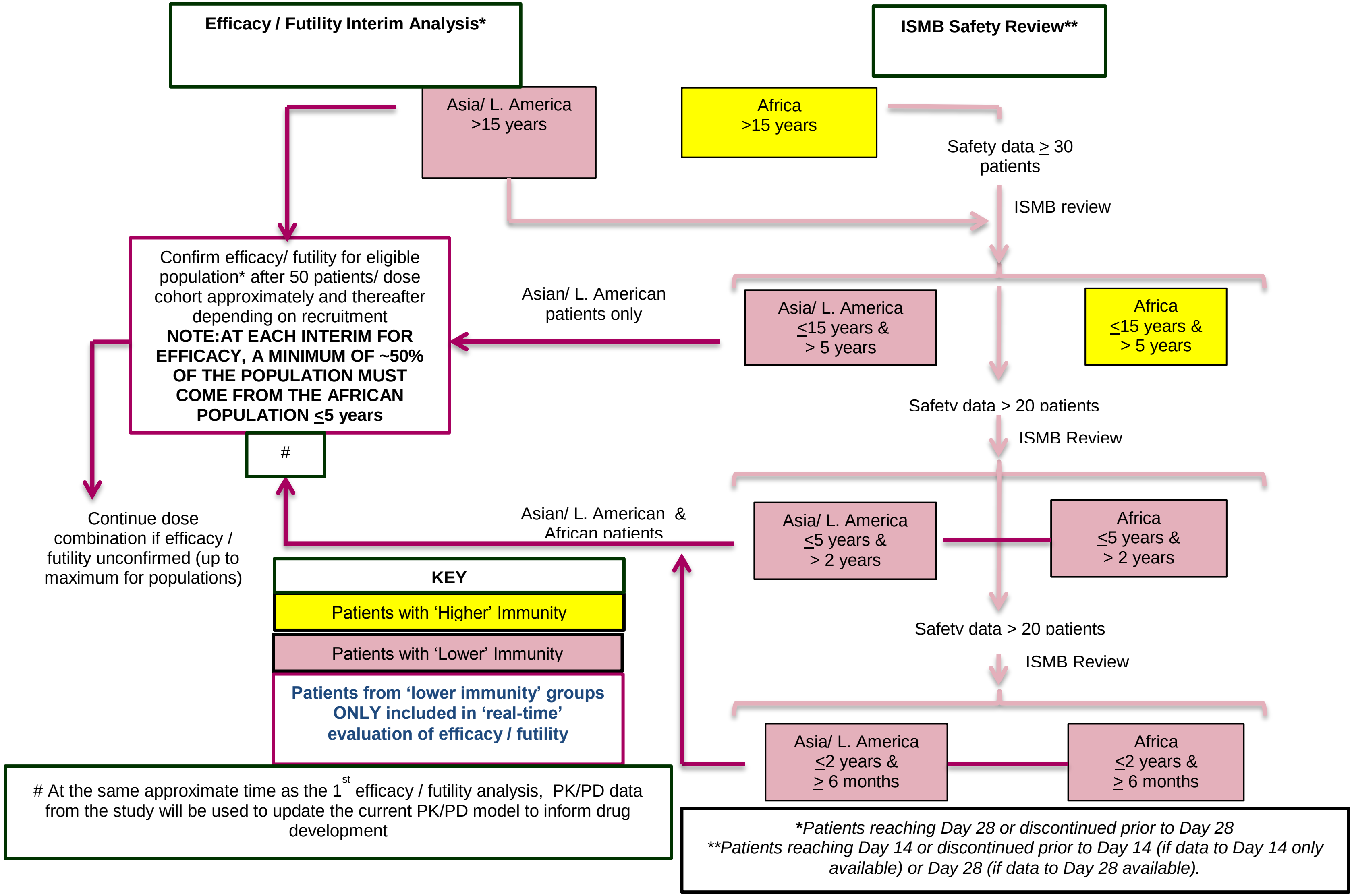

3. BACKGROUND

3.1. Introduction

Plasmodium falciparum malaria is a parasitic disease that kills over 600,000 people and results in up to 500 million cases annually affecting mainly young children and pregnant women. The economic consequences of malaria are enormous in endemic regions with an estimated USD 12 billion per year lost gross domestic product (GDP) and a loss of 45 million years of productive life due to deaths and disability. Malaria is curable and preventable. Principal control strategies including rapid diagnosis, effective treatment and personal protection with bed nets.

Current WHO guidelines (WHO 2010) recommend that artemisinin-based combination therapies are used to treat uncomplicated *P. falciparum* malaria to counter the threat of resistance of *P. falciparum* to monotherapies and to improve treatment outcome. The artemisinins produce rapid clearance of parasitaemia and resolution of symptoms, by reducing parasite numbers 100- to 1000-fold per asexual cycle of the parasite. Because artemisinin and its derivatives are eliminated rapidly, when given alone or in combination with rapidly eliminated compounds a 7-day course of treatment is required (WHO 2010).

The long duration of treatment with the artemisinins can be reduced to 3 days when given in combination with slowly eliminated anti-malarial drugs such as 4-aminoquinoline drugs. With this shorter 3 day course, the complete clearance of all parasites is dependent on the partner medicine being effective and persisting at parasiticidal concentrations until all infecting parasites have been killed.

However evidence suggests that compliance to the 3 day treatment course is low, which would be expected to reduce real-life effectiveness. In addition, evidence of emergence of potential plasmodial resistance to artemisinins suggests that artemisinin resistance may become an issue in the next years (Jambou et al. 2011, Ding et al. 2011). Thus new drug combinations are required, and effective single exposure combination treatments in particular are likely to lead to significant improvement in real-life effectiveness. Recently, mutations in a gene called Kelch13 (K13) have been highly associated with artemisinin resistance (delayed clearance phenotype) in *Plasmodium falciparum* strains isolated in South East Asia (Ariey *et al* 2014). As an exploratory objective, we will screen blood spots from patients from different areas. This analysis, combined with parasite clearance kinetics, will help us to understand if OZ/PQP is effective against artemisinin-resistant malaria. As parasite genotypes of interest associated with drug resistance may be identified or confirmed over the duration of the trial, additional analyses may be carried out on collected samples.

OZ439

OZ439 is a novel, synthetic trioxolane that shows promise as a peroxidic anti-malarial agent. Trioxolanes are closely related to artemisinins in that their chemical structure contains the peroxidic pharmacophore that leads to the potent anti-malarial activity of these agents.

In vitro, OZ439 is a potent inhibitor of both chloroquine-resistant and -sensitive forms of *P. falciparum*. Cross-resistance with other anti-malarial agents was also not observed with OZ439 when tested against a broad panel of *P. falciparum* strains from various

geographical regions. Moreover, it displayed additive anti-malarial activity in combination with mefloquine, piperaquine, pyronaridine or amodiaquine *in vitro*.

OZ439 showed embryo-foetal toxicity potential in rat but not rabbit. For further information on the non-clinical safety profile of OZ439, please refer to the current Investigator's Brochure.

OZ439 has demonstrated acute efficacy in a Phase IIa study carried out in Asian adults (MMV_OZ439_10_002), and in the Human Volunteer *P. falciparum* Blood Stage Challenge Model (QP12C10). These data have been integrated into a PK/PD model capable of predicting the anti-malarial effect of different OZ439 doses (see Section 3.4 PK/PD Summary).

Piperaquine Phosphate (PQP)

PQP is a currently marketed long acting anti-malarial, and has been in clinical use for at least 20 years. In the European Union, PQP is marketed in a fixed dose combination with the short acting anti-malarial dihydroartemisinin (DHA) with the brand name Eurartesim®. Dosing is based on body weight, according to a dosing table. The adult dose (for 36 to <75kg) is 3 tablets (960mg PQP and 120mg DHA) administered in the fasted state for 3 days.

The efficacy and safety of *Eurartesim* have been assessed in two large randomised, open-label clinical studies conducted in Africa and in Asia. *Eurartesim* treatment was compared with artemether-lumefantrine in the African study and with mefloquine + artesunate in the Asian study and shown to be non-inferior to the comparator treatments.

PQP has the potential to increase QTc. The potential for *Eurartesim* to prolong the QTc interval was investigated in parallel groups of healthy volunteers who took each dose (4x 320 mg PQP/ 40 mg DHA) with high (~1000 Kcal) or low (~400 Kcal) fat/calorie meals or in fasting conditions. Compared to placebo, the maximum mean increases in QTcF on Day 3 of dosing with *Eurartesim* were 45.2, 35.5 and 21.0 ms under these respective dosing conditions. No healthy subject dosed in fasting conditions showed a QTcF greater than 480 ms or an increase over baseline greater than 60 ms. The number of subjects with QTcF greater than 480 ms after dosing with a low fat meal was 3/64, while 10/64 had QTcF values over this threshold after dosing with a high fat meal. No subject had a QTcF value greater than 500 ms in any of the dosing conditions.

For further details on the safety and efficacy of piperaquine phosphate refer to the EMA EPAR for *Eurartesim*.

For safety and tolerability of PQP in combination with OZ439, see Sections 3.3 and 3.5.

Summary of Formulations used in Clinical Studies

Two types of formulation of OZ439 have been used in the majority of clinical studies to date; OZ439 'powder in bottle' (PIB) which is reconstituted to provide an oral suspension and OZ439 administered with TPGS (reconstituted to provide an oral solution/ suspension).

In order to achieve therapeutic exposure, OZ439 PIB must be administered with full fat milk. A TPGS containing formulation administered in the fasted state gives similar drug

exposure (~90%) to that obtained with OZ439 PIB administered with milk. A number of prototypes of TPGS containing formulations have been administered during development of the Phase IIb OZ439 + TPGS granules formulation. The Phase IIb study formulation consists of OZ439 granules plus TPGS granules, and the bioavailability of this formulation has been assessed in study MMV_OZ439_13_004 (co-administered with PQP), in order to confirm tolerability and that the expected exposure levels are achieved, prior to running the current study. Finally, a powder in capsule formulation of OZ439 was used only in the First In Human study and due to low systemic exposure was not used in further studies.

PQP tablets have been used in all studies (except Study MMV_OZ439_13_004). Co-administration of PQP with milk (i.e. with OZ439 PIB) results in a significant increase in exposure of PQ compared with the fasted state (i.e. with OZ439 + TPGS) (See Table 2 below). This leads to an increased effect on QTc which is discussed further below.

In the current Phase IIb study, OZ439 and PQP are co-administered in the fasted state. Two formulations of PQP are used in the Phase IIb study, PQP tablets (for patients able to swallow tablets) and a PQP granule formulation which is reconstituted together with the OZ439 + TPGS granules formulation to produce an oral suspension for children unable to swallow tablets. The bioavailability of these formulations was assessed in study MMV_OZ439_13_004 (co-administered with OZ439 + TPGS).

3.2. Clinical Data

A full list of completed studies conducted with OZ439 and / or PQP are listed in Table 1 (below). To date, OZ439 has been evaluated in four Phase I studies in healthy volunteers, and one Phase IIa study in patients with acute, uncomplicated *P. falciparum* or *P. vivax* malaria mono infection. In addition, two Phase I studies of OZ439 in loose combination with PQP have been completed and one bioavailability study of OZ439 in loose combination with PQP is ongoing.

OZ439 is also currently being investigated in a Phase II study over an extended observation period of 28 days to assess the effects of single doses of OZ439 on the recrudescence of *P. falciparum* malaria. At the time of writing this protocol, a total of 6 patients had been dosed with 100 mg of OZ439.

Furthermore, the PK/PD relationship of both OZ439 and PQP has been assessed in two separate studies using a Human Volunteer *P. falciparum* Blood Stage Challenge Inoculum Model (Challenge Model) to healthy volunteers.

Drug Exposure

Table 2 summarises the maximum drug exposures achieved in key studies, including those studies providing key efficacy and tolerability data supporting the dose rationale for this study. These exposures were generally well tolerated (see more detail below) and exposure in the current study is predicted not to exceed exposure levels investigated in prior studies. For further information see Section 3.8, Dose Rationale.

The highest OZ439 exposure (AUC) achieved in healthy subjects to date was in an OZ439 relative bioavailability study (MMV_OZ439_11_001) in which a prototype OZ439 formulation containing TPGS was compared with OZ439 PIB administered in the fed state. The highest exposure was achieved following administration of the 800mg OZ439 PIB

formulation in the fed state. Slightly lower exposure was obtained in Part B of the 'First in human' study (MMV_OZ439_09_001) following administration of 800mg OZ439 PIB with milk, and following administration of 1200mg PIB with milk to malaria patients in a Phase IIa study (MMV_OZ439_10_002).

Table 1: Studies to Date: OZ439 or PQP alone, OZ439/PQP loose Combination

**Abbreviated Title**	**Design**	**Treatment**	**No of Subjects**
**Phase I:**			
**MMV_OZ439_09_001**			
OZ439 'First in human' safety / PK study in HV<br>Part A: SD escalation<br>Part B: food effect<br>Part C: MD (3 days)	Part A: Randomised, double-blind placebo-controlled incomplete x-over<br>Part B: Randomised, double-blind, x-over<br>Part B: Randomised, double-blind, parallel group, placebo-controlled	A: OZ439 50-1600 mg<br>B: OZ439 800 mg (fed/ fasted)<br>C: OZ439 200-800 mg<br>OZ439 treatment<br>Placebo	24<br><br><br>72<br>24
**MMV_OZ439_11_001**			
OZ439 BA Study of two Prototype formulations vs reference* in HV	Relative BA of prototype OZ439 formulations vs reference formulation in fasted and fed* state & with milk	OZ439 800 mg (fed/fasted)	52
**MMV_OZ439_12_003**			
3-way randomised cross-over single-dose OZ439 in healthy subjects in HV	Relative BA of nanoparticulate OZ439 via Enterion™ caps (to proximal small bowel) vs ref.* & oral nanoparticulate	OZ439 120 mg	11
**MMV_OZ439_12_002**			
OZ439 + PQP safety /PK SD escalation study HV<br>(2 periods/cohort)<br>Period 1 OZ439 alone<br>Period 2: OZ439 + PQP	Placebo-controlled double-blind parallel group design of co-administered OZ439* and PQP tablets. 5 cohorts.	OZ439:PQP doses<br>100/160mg to 800/1440mg<br>OZ439 / PQP treatment<br>placebo	59<br><br>39<br>20
**MMV_OZ439_12_001**			
OZ439 + MQ safety /PK SD escalation study HV<br>(2 periods/cohort)<br>Period 1 OZ439 alone<br>Period 2: OZ439 + MQ	Placebo-controlled double-blind parallel group design of co-administered OZ439* and MQ tablets). 2 cohorts.	OZ439 : MQ doses<br>100/250mg &400/750mg<br>OZ439+MQ treatment<br>Placebo	24<br><br>18<br>6
**MMV_OZ439_12_005**			
Dose-escalation study of OZ439 daily for 3 days in HV	PK, safety, tolerability of OZ439* administered with milk	OZ439: 300, 600 & 700mg<br>OZ439 treatment<br>Placebo	34<br>22<br>12
**MMV_OZ439_13_002**			
SD PQP +OZ439+TPGS prototype formulation BA study vs reference PQP +OZ439* in HV	Relative BA study of co-administered OZ439 prototype formulation (TPGS) + PQP tablets (fasted). 3 Cohorts, parallel group	OZ439:PQP<br>800+TPGS:960mg<br>800 reference*:1440mg<br>800+TPGS:1440mg	24
**QP12C10**<br>**(Brisbane, Australia)**			
SD OZ439 in Blood stage *P. falciparum* challenge inoculum as model (HV)	SD, 2 cohort PK/PD study to assess the effect of OZ439 on clearance of blood stage *P. falciparum*	OZ439* 100 & 500mg	24
**QP13C05**<br>**(Brisbane, Australia)**			
SD PQP in Blood stage *P. falciparum* challenge inoculum as model (HV)	SD, 2 cohort PK/PD study to assess the effect of PQP on clearance of blood stage *P. falciparum*	PQP 640 & 960mg	24
**Phase II:**			
**MMV_OZ439_10_002**<br>**(Bangkok, Thailand and Tak, Thailand)**			
Open-label, SD efficacy in subjects with acute, uncomplicated *P. falciparum* or *P. vivax* malaria mono-infection	Preliminary acute efficacy, tolerability & PK of OZ439 in acute, uncomplicated *P. falciparum* or *P. vivax* (4 cohorts)	OZ439* 200-1200 mg	81

SD: Single dose, MD: Multiple dose, BA: Bioavailability, PQP: Piperaquine phosphate, MQ: Mefloquine, HV: Healthy volunteers.
*OZ439 powder in bottle formulation to produce oral suspension.

Table 2: Key Studies - Drug Exposures OZ439 and OZ439 / PQP

	Design	OZ439 Dose (mg)	Cmax ng/mL (CV%)	AUCinf ng.h/mL (CV%)	PQP Dose (mg)	Cmax ng/mL (CV%)	AUC168h ng.h/mL (CV%)	AUCinf ng.h/mL (CV%)
MMV_OZ439 _09_001	FIH, Part B	800mg* fed	2220 (52.6)	23100 (48.9)	NA	NA		NA
MMV_OZ439 _11_001	BA OZ439 + TPGS prototype BA	800mg* fed (reference)	1910 (32.9)	29200 (31.2)	NA	NA		NA
MMV_OZ439 _10_002	Phase IIa efficacy	1200mg*	1500 (90.0)	25100 (85.1)	NA	NA		NA
MMV_OZ439 _12_005	3 Day dose escalation (Exposures on Day 3)	700mg* milk	1790 (32.2)	20500 (32.0)**	NA	NA		NA
MMV_OZ439 _12_002	OZ439 + PQP safety/ DDI	800mg* milk<br>100mg* milk	1650 (26)<br>199 (27)	20700 (41)<br>1520 (26)***	1440mg milk<br>1440mg milk	356 (54)<br>393 (44)		13200 (54)<br>17500 (26)
MMV_OZ439 _13_002	OZ439+TPGS Phase IIb prototype + PQP BA	800mg* milk (reference)<br>800mg +TPGS	1610 (37.2)<br>1540 (26.0)	18600 (45.4)<br>17500 (27.9)	1440mg milk<br>1440mg	630 (54.4)<br>202 (37.6)	12000 (41.3)<br>6510 (32.0)	29700 (48.0)<br>17200 (29.5)
MMV_OZ439 _13_004	OZ439+PQP Phase IIb formulation BA	800mg+ TPGS<br>800mg+ TPGS<br>800mg+ TPGS	1260 (35)<br>1500 (40)<br>1270 (29)	14200 (26)<br>15800 (35.7)<br>14100 (34.7)	1440mg Tabs<br>960mg Tabs<br>960mg Gran	249 (94)<br>113 (101)<br>114 (66)	6970 (52.5)<br>4220 (53.2)<br>4560 (46.0)	NA<br>NA<br>NA

Exposures are Geometric mean. *OZ439 PIB, ** Day 3 AUCτ, *** AUC168h
FIH= First in human study, BA= bioavailability study, DDI= drug-drug interaction study, Tabs = tablets, Gran= granules

Two Phase I studies of OZ439 in loose combination with PQP have been completed; a study to determine the safety, tolerability and PK of ascending combination doses (MMV_OZ439_12_002) using OZ439 PIB and PQP tablets, and a study of a prototype of the OZ439 Phase IIb formulation, containing TPGS plus PQP tablets (MMV_OZ439_13_002). A further bioavailability study (MMV_OZ439_13_004) is currently nearing completion of the clinical phase. This is a key study as it evaluates the safety, tolerability and PK of the formulations that will be used in the current Phase IIb study.

The OZ439 and PQP exposures achieved are noted in Table 2 above. The overall tolerability of the exposures achieved in these studies was considered acceptable (see below).

MMV_OZ439_09_001

This was a three-part first-in-man study conducted in a total of 63 male and female subjects to investigate the single dose (Part A) and multiple dose (Part B) tolerability of OZ439 and the effect of food (Part C) on the pharmacokinetics (PK) of OZ439 and its metabolites.

In Part A (powder in capsule formulation) the overall incidence of AEs was highest in the OZ439 1600mg group with specific AEs experienced by >1 subjects being diarrhoea 3/6 (50%), nausea 3/6 (50%) and gastrointestinal hypermotility 2/6 (33.3%). Two subjects out of 24 were prematurely discontinued due to AEs of syncope vaso-vagal (800mg OZ439) and atrio-ventricular block (placebo) that were considered to be related to study drug.

In Part B (800mg OZ439 PIB), the only AE experienced by >1 subjects was headache (fed regimen) 2/12 (16.7%). In Part C (PIB), the only AEs reported by >1 subject in any treatment group were headache (2/6) at 200mg and flushing (2/6) at OZ439 800mg.

Across the study, no clinically meaningful changes were observed in clinical laboratory variables, physical examination, vital signs and ECG findings (apart for those mentioned above) and the safety and tolerability of OZ439 was considered satisfactory.

MMV_OZ439_10_002

This Phase IIa exploratory, single dose study assessed the preliminary efficacy, tolerability and PK of OZ439 in subjects with acute, uncomplicated *Plasmodium falciparum* or *vivax* malaria mono-infection.

A total of 81 subjects received either 200, 400, 800 or 1200 mg single oral dose of OZ439 PIB with full fat milk and were followed for parasitaemia, tolerability and pharmacokinetics after drug administration.

OZ439 exposure increased in an approximately dose proportional manner and exposure was similar to that of healthy subjects.

Administration of OZ439 caused similar anti-parasitic effects at all doses investigated over the 36 hour period following dosing. The PRR24 for *P. falciparum* ranged from 1.38 to 1.71 leading to a 97.9% reduction in parasite concentrations by 36 hours. The PRR24 for *P. vivax* ranged from 1.96 to 2.18 leading to a 99.6% reduction in parasite concentrations by 36 hours. Estimated PRR48 was approximately 3.

The overall tolerability was good, with most changes in laboratory variables and AEs mild, reversible, not dose related, and compatible with acute malaria. No particular pattern of AEs was discernible.

The highest incidence of treatment-related AEs was reported in the 1200 mg cohort for both *P. falciparum* and *P. vivax* patients. The most frequently reported treatment-related clinical AEs were of the blood/lymphatic, cardiac, gastrointestinal and nervous systems; most were mild. There were 3 patients with AST/ALT increases >3x ULN but <5x ULN after 200 mg, 400 mg and 800 mg respectively. One patient had increases >5x ULN but <8x ULN and one patient had increases >8x ULN (both at the 400 mg dose level). There were no Hy's law cases.

There were 2 patients with QTcF >450 ms and the highest value observed was 506 ms (pre-dose 423 ms) in a patient in the 1200 mg cohort. QTcF increases from pre-dose baseline were observed in a total of 18 patients who displayed 26 instances of QTcF >30 ms; 3 patients had one instance each of QTcF >60 ms. In addition, three instances of reversible right bundle branch block were observed, one in a patient with accompanying T-wave changes compatible with pericarditis event at baseline.

In conclusion, OZ439 was well tolerated when administered as a single dose up to 1200 mg. Treatment-emergent increases in liver function tests and QTc prolongation were detected. It is possible that these findings were related to the underlying disease.

MMV_OZ439_12_005

This was a randomised, placebo-controlled, dose-escalation study to investigate safety and tolerability of OZ439 (OZ439 PIB with milk) dosed once daily for 3 days to healthy subjects. Doses were 300, 600 and 700mg. A total of 34 subjects were treated (55.9% female), 22 active treatment and 12 placebo.

For the limited dose range tested, the OZ439 exposure (as based on AUCτ) was roughly dose-proportional. Accumulation on Day 3 was around 1.6 fold.

Overall, the safety findings demonstrated OZ439 dosed once daily for 3 days was well tolerated. Of 16 treatment-related AEs, 14 were of mild intensity and two of moderate intensity (migraine and vomiting). The most common treatment-related AEs were gastrointestinal disorders. At the 700mg dose 6/8 subjects (75%) had gastrointestinal disorders; 4/8 (50%) nausea and 1/8 (12%) for each of abdominal pain, diarrhoea, constipation and vomiting. At 700mg, nausea associated with vomiting occurred for one subject. All instances of nausea were of mild intensity. The instance of nausea was numerically correlated with dose.

There were no clinically significant changes in clinical laboratory tests, physical examinations, vital signs, Holter ECGs, telemetry and 12-lead ECG parameters at any dose.

MMV_OZ439_12_002

This was placebo-controlled 2 period study of the safety, tolerability and PK of OZ439 (PIB with milk) co-administered with PQP tablets, in which OZ439 was administered alone in Period 1 and the same dose of OZ439 was administered in Period 2 to healthy subjects. Subjects allocated to placebo treatment received placebo on both Periods.

Five cohorts were dosed in ascending order: Cohort 1 - 100mg OZ439 + 160mg PQP; Cohort 2 - 100mg OZ439 + 480mg PQP; Cohort 3 - 100mg OZ439 + 1440mg PQP; Cohort 4 - 300mg OZ439 + 1440mg PQP; Cohort 5 - 800mg OZ439 + 1440mg PQP.

The highest exposure of OZ439 was seen in Cohort 5, Period 2, while the highest exposure of PQ occurred in Cohort 3 (see Table 2). A small effect of PQP on OZ439 exposure was demonstrated with the geometric mean ratio of Period 2 AUC to Period 1 AUC ranging from 1.07 (90% CI 0.82-1.39) to 1.67(90% CI 1.36-2.06).

The most common System Organ Class (SOC) for related treatment-emergent AEs (TEAEs) (>10 TEAEs) was gastrointestinal disorders (6 subjects in the active treatment groups and 1 subject in the placebo group) followed by General disorders (2 subjects with active treatment).

Twenty-four (24) of the 41 TEAEs; 11 with the active treatments and 13 with placebo, were considered treatment-related. These were nausea (5 subjects), diarrhoea (1 subject), malaise (2 subjects) and vomiting (1 subject with OZ439 800 mg/PQP 1440 mg). There were no deaths.

The highest frequency of related TEAEs was observed in the highest dose group (OZ439 800 mg/PQP1440mg) (1/6 [16.7%] subjects; 5 TEAEs), followed by the placebo group

(3/20 [15%] subjects, 13 TEAEs). Frequency in the other treatment groups was 8.3 – 14.3%.

One SAE was reported for one subject who received OZ439-matching placebo (Cohort 1/Period 1): the SAE consisted of clinically significant eosinophilia, which required hospitalisation and was associated with duodenitis with eosinophilic infiltration.

Clinical laboratory test results were in general within normal range (with the exception those associated with the SAE of duodenitis with eosinophilic infiltration). No clinically significant changes in vital signs occurred.

In this study, cardiac safety was closely monitored on an ongoing basis as PQP is known to prolong QTc. Triple 12-lead ECG tracings were collected throughout the study (up to 1008 hours post-dose, Period 2). Additionally, continuously recorded ECG was obtained for a 24 hour period prior to dosing on Period 1 and for 24 hours post-administration on Periods 1 and 2.

The effects of OZ439 on ECG were minimal (Period 1). At the highest dose of 800mg, a maximum mean increase (from baseline and placebo (ΔΔ)) of 8.5 ms was observed 6 hours post-dose.

Concomitant administration of OZ439 and PQP resulted in PQP-dose dependent increase in QTcF. QTcF prolongation was mild up to and including PQP doses of 480 mg (upper limit of confidence interval (CI) between 10 and 20 ms). For the 3 cohorts receiving the highest PQP dose (1440mg), the maximum mean QTcF prolongation (from baseline and placebo (ΔΔ)) ranged between 15.9 and 26.4 ms and the upper limit of CI reached 33.4 ms (Cohort OZ439 100mg/PQP 1440mg).

Categorical analysis showed no values of QTcF > 450 ms. Change from baseline in QTcF > 30 ms were recorded during Period 2 in subjects having received a dose of 1440mg PQP (cohorts 3 to 5, OZ439 100, 200 and 800 mg) and in one subject in the 100 mg OZ439 / 480mg PQP dosing group (cohort 2).

In conclusion, safety data confirmed a favourable safety profile for OZ439 administered at the single doses of 100, 300 and 800 mg in combination with PQP 160, 480 and 1440 mg, but with QTc prolongation observed in subjects who received OZ439 with 1440 mg PQP.

Of note, this study used OZ439 PIB formulation, administered with milk. As demonstrated in the bioavailability study described below (MMV_OZ439_13_002), co-administration of PQP with milk results in significantly higher PQ exposure compared with the fasted state (i.e. when co-administered with OZ439 + TPGS). The lower exposure of PQP when administered with OZ439 + TPGS was confirmed in study MMV_OZ439_13_004 in which the actual Phase IIb formulations were evaluated (see below).

The predicted QTc effect for the current Phase IIb study is discussed in Section 3.5, Tolerability Summary.

MMV_OZ439_13_002

The objectives of this open-label, parallel group study was to investigate the safety, tolerability and PK of co-administered PQP tablets (to be used in Phase IIb) with a

prototype of the OZ439 Phase IIb formulation (OZ439 + TPGS formulation) in the fasted state, compared with a reference combination formulation: PQP tablets with OZ439 PIB + milk in healthy subjects. Treatments A, B, and C were: OZ439 800mg + TPGS plus PQP 1440mg, OZ439 800mg + TPGS plus PQP 960mg and OZ439 800mg PIB + milk plus PQP 1440mg respectively.

OZ439 exposure was comparable between the different treatments; AUC_{inf} of 17500 (CV% 27.9), 16000 (CV% 24.7) and 18600ng.h/mL (CV% 45.4) respectively for treatments A, B and C) demonstrating that OZ439 +TPGS administered in the fasted state gives similar exposure to OZ439 PIB administered with milk and hence OZ439 +TPGS is a suitable formulation to ensure adequate exposure of OZ439 in the fasted state in Phase IIb.

PQP exposure is tabulated below:

**Treatment**	**A**	**B**	**C**
**Dose OZ439: PQP and formulation**	**800mg + TPGS: 1440mg**	**800mg + TPGS: 960mg**	**800mg PIB + milk: 1440mg**
**Number of subjects**	**8**	**8**	**8**
**Cmax (ng/mL)**	**202 (37.6)**	**122 (42.1)**	**630 (54.4)**
**AUCinf (ng.h/mL)**	**17200 (29.5)**	**13400 (23.1)**	**29700 (48.0)**

Geometric mean (CV%)

PQP (1440 mg) co-administered with milk (and OZ439) i.e. treatment group C, resulted in significantly higher exposure compared with 1440mg PQP when administered with OZ439 + TPGS (i.e. treatment A).

Subjects in treatment group C (1440mg PQP co-administered with milk) had higher QTcF values (mean maximum Δ27.5ms and individual maximum of 50ms) in comparison to treatment groups A (1440mg PQP fasted) (mean maximum Δ12.4ms and individual maximum of 32ms) and B (960mg PQP fasted) (mean maximum Δ15.6ms and individual maximum of 31ms).

This finding was mirrored in the categorical analysis of QTc Prolongation.

**Dose OZ439: PQP / formulation**	**800mg + TPGS: 1440mg**		**800mg + TPGS: 960mg**		**800mg PIB + milk: 1440mg**	
	**QTcB**	**QTcF**	**QTcB**	**QTcF**	**QTcB**	**QTcF**
**Absolute value > 450 ms**	**0**	**0**	**0**	**0**	**2**	**5**
**Change from baseline > 30 ms**	**2**	**2**	**5**	**1**	**6**	**9**
**Change from baseline > 60 ms**	**0**	**0**	**0**	**0**	**0**	**0**

There were no SAEs in this study and no withdrawals due to TEAEs. Out of the 28 TEAEs reported in 15 of 24 (62.5%) subjects, 21 were judged by the Investigator as probably or possibly treatment-related.

In treatment groups A and B the most commonly reported treatment-related TEAEs were gastrointestinal disorders (treatment group A: seven TEAEs reported in 4 of 8 [50.0%] subjects; treatment group B: four TEAEs reported in 3 of 8 [37.5%] subjects). No gastrointestinal related TEAEs were reported in treatment group C.

Two TEAEs of moderate intensity (nausea) and two of severe intensity (vomiting) were reported in treatment group A. No TEAEs of moderate or severe intensity were reported in treatment groups B or C. The reason for the higher level of gastrointestinal events in this study is unclear, however may be related to the taste of the TPGS formulation and the close proximity of the subjects dosed in Cohort A in the study.

There were no clinically significant changes in Laboratory safety measurements or Vital signs.

MMV_OZ439_13_004

This was an open-label bioavailability study to determine the safety, tolerability and PK exposure of the formulations that will be used in the current Phase IIb study. Treatments A, B and C were randomised within three cohorts. Clinical conduct of the study is ongoing. Fifteen subjects received Treatment A (1440mg PQP tablets plus 800mg OZ439 + TPGS granules) and 14 subjects each received Treatment B (960 mg PQP tablets plus 800 mg OZ439 + TPGS granules) and Treatment C (960mg PQP granules plus 800mg OZ439 + TPGS granules). Dosing of Cohort 4 (Treatment D: 800mg OZ439 + TPGS granules) is ongoing.

Preliminary PK data indicates that exposure of OZ439 and PQ for Treatments A, B and C is within the range obtained for the prototype formulations from which the predicted QTc prolongation and efficacy was obtained (MMV_OZ439_13_002).

Tolerability was generally good. Preliminary data indicates that nausea was the most frequently reported AE, occurring in 4/15 of subjects on treatment A, 3/14 on Treatment B and 4/14 on Treatment C. Other AEs occurring in 1 or 2 subjects were changes in bowel habit, bloating and epigastric pain. There were no clinically significant changes in Laboratory safety measurements except 1 subject with an ALT increase >2x ULN on Day 29, with AST <1.5 x ULN (treatment B) and 1 subject with ALT increase >1.5x ULN on Day 15, with AST >2 x ULN (treatment C).

The Mean and Mean Maximum ΔQTcF increases for Treatments A, B and C were, 16ms and 26ms; 10ms and 21ms and 10ms and 22ms i.e. as expected given the exposure levels.

OZ439 Malaria Challenge Study

The anti-malarial activity of OZ439 was investigated in the Healthy Volunteer *P. falciparum* Malaria Challenge Model (MCM) (Study QP12C10).

In this model, drug is administered when parasitaemia, measured by qPCR, reaches 1000 parasites/µL. Volunteers are naïve to malaria and therefore considered to have no immunity ('non-immune'). Riamet® is administered as standard of care where there are no signs of response to drug, where there are emerging signs and symptoms of malaria or where recurrence of parasites up to baseline levels occurs.

Three cohorts of 8 volunteers were inoculated with infected erythrocytes and were administered OZ439 doses of 100 mg, 200 mg and 500 mg (OZ439 PIB with milk).

OZ439 displayed linear kinetics in the MCM. The AUC and Cmax increased with dose and was similar to exposures observed in patients and volunteers.

OZ439 reduced parasitaemia in the volunteers in an exposure dependent manner: Data confirmed the minimum parasiticidal concentration (MPC) of OZ439 to be 3.0ng/mL (56%CV). PK/PD modeling allowed determination of anti-parasitic parameters (see Section 3.4, PK/PD summary).

PQP Malaria Challenge Study

The anti-malarial activity of PQP was investigated in the Malaria Challenge Model (QP13C05). Two cohorts were recruited; the 1st cohort (5 volunteers) received 960 mg and the 2nd cohort (7 volunteers) received 640 mg PQP.

PQP displayed linear kinetics that matched those observed in healthy volunteer trials. Data confirmed the MPC to be 12.4ng/mL (59% CV). The PRR observed for PQP was between 3 and 4 (see Section 3.4, PK/PD summary).

3.3. PK/PD summary

Pre-clinical data (in the case of OZ439) and clinical data (in the case of both OZ439 and PQP) as described above, combined with evaluation of literature data on PQP was used to establish a PK/PD relationship of the predicted efficacy for OZ439 and PQP alone. This data was integrated to estimate the MPC and parasite reduction ratio (PRR) for each drug as shown below.

OZ439 Parameters (based on pre-clinical, MCM and Phase IIa data)

Parameter	Unit	Value
PRR		3.0 ± 0.5
MPC	ng/mL	3.0 (56%CV)

PQP Parameters (based on pre-clinical, MCM and published data)

Parameter	Unit	Value
PRR		3.0 ± 0.9
MPC	ng/mL	12.4 (59% CV)

The results obtained from the Malaria Challenge Model were consistent with the anti-malarial effects observed in the Phase IIa study in Asian patients (MMV_OZ439_10_002) and published data (Price et al 2007) respectively for OZ439 and PQP. The PK/PD model was used to predict doses of each drug (monotherapy) leading to specific reductions in parasitaemia. The combined effect of co-administered OZ439 and PQP was estimated assuming that the two drugs would act independently with an additive effect on the parasite clearance rate.

The PK/PD model simulations used PK simulated from the population PK models derived for each drug including the slight inhibitory effect that PQP has on OZ439 clearance.

A range of dose combinations were 'tested', where a sufficient result was one where the predicted parasitaemia decreased by $>10^{11}$-fold on average for the lower 2.5th percentile of the population. Inclusion of the lower 2.5th percentile was to ensure that the predicted effect was to achieve efficacy of the drug combination for 97.5% of the population.

Simulations using the PK/PD model of OZ439 and PQP in combination (taking into consideration tolerability) indicated that the optimal dose combinations to determine a dose-response relationship of parasite clearance were 800 mg OZ439, plus 640, 960 and 1440 mg PQP (see Section 3.8, Dose Rationale).

3.4. Tolerability Summary

ΔΔQTcF

The cardiac safety of OZ439 plus PQP has been studied within human volunteer studies and in addition the cardiac safety of PQP was well characterised to support *Eurartesim* product registration.

In healthy subjects, the effect of OZ439 800mg alone on QTc was minimal, with a ΔΔ maximum mean QTcF increase of 8.5 ms (Study MMV_OZ439_ 12_002). In the Phase IIa study of OZ439 administered to Asian patients, QTc increases were observed. The highest absolute value was 506ms in a patient given 1200mg. Three patients had one instance of ΔQTcF >60ms. There were 3 instances of reversible right bundle branch block. It is possible that some of these effects could have been related to the underlying disease.

In Study MMV_OZ439_12_002, concomitant administration of OZ439 (PIB with milk) and PQP resulted in PQP-dose dependent increase in QTcF. For the 3 cohorts receiving the highest PQP dose (1440mg), the ΔΔ maximum mean QTcF prolongation ranged between 15.9 and 26.4 ms and the upper limit of confidence interval reached 33.4 ms (OZ439 100mg/PQP 1440mg) and was associated with the highest PQP exposure (Cmax 393ng/mL and AUCinf 17500ng.h/mL).

Similarly, following co-administration of 800mg OZ439 (PIB with milk) and 1440mg PQP in Study MMV_OZ439_13_002, the Δ mean maximum QTcF was 27.5ms (associated with a Cmax of 630ng.mL and AUC of 29,700ng.h/mL). However administration of PQP in the fasted state (i.e. with OZ439 + TPGS), results in lower QTcF increases: 1440mg PQP fasted = Δ mean maximum QTcF 12.4ms (associated with a lower PQ exposure, Cmax 202ng.mL and AUC 17200ng.h.mL) and 960mg PQP fasted Δ mean maximum QTcF 15.6ms.

A relationship between piperaquine (PQ) C_{max} and ΔΔQTcF was established using data from MMV_OZ439_12_002 (co-administered with OZ439 PIB with milk). The PQ $C_{max,}$ via a nonlinear relationship, determined the magnitude of ΔΔQTcF prolongation observed.

To take account of the effect of formulation on PQ exposure, the PQ C_{max} following administration of 1440mg PQP from MMV_OZ439_13_002 (co-administered with OZ439 + TPGS in the fasted state) was used within the PK/PD relationship established for MMV_OZ439_12_002, to predict ΔΔQTcF prolongation for the doses of PQP selected for the current Phase IIb (i.e. when administered with OZ439 + TPGS in the fasted state).

The predicted ΔΔ mean max QTcF was 13, 15 and 18ms for PQP doses of 640, 960 and 1440mg respectively.

Preliminary data from study MMV_OZ439_ 13_004, for the formulations that will used in current Phase IIb study, confirms that exposure is within the range of exposure obtained in study MMV_OZ439_13_002 and also confirms a similar QTc effect, with a Mean Maximum ΔQTcF increase for PQP doses of 1440mg (tablet), 960mg (tablet) and 960mg (granule) of 26ms; 21ms and 22ms respectively.

Thus the predicted ΔΔ mean max QTcF remains 13, 15 and 18ms for PQP doses of 640, 960 and 1440mg respectively of the Phase IIb formulations (i.e. no dose adjustment required). Note ΔΔQTcF is on average 3-5ms lower than ΔQTcF.

Tolerability Endpoints

OZ439 exposures up to an approximate mean Cmax of 2000ng/mL and AUCinf of 29000ng.h/mL (associated with OZ439 doses of 800mg) and PQ exposures up to an approximately mean Cmax of 600ng/mL and AUCinf of 30000ng.h/mL (associated with a PQP dose of 1440mg co-administered with OZ439 up to a dose of 800mg) have been demonstrated to have acceptable tolerability, which supports evaluation of similar exposures in Phase IIb.

In healthy volunteer studies of OZ439 alone or co-administered with PQP, no clinically meaningful changes were observed in clinical laboratory variables, physical examination and vital signs. No clinically meaningful changes in ECGs were observed with OZ439 administered alone.

The most frequently reported treatment-related AEs on administration of OZ439 alone or co-administered with PQP were gastrointestinal; predominantly nausea and occasionally vomiting. Diarrhoea, constipation, gastrointestinal hypermotility and abdominal pain have also been reported infrequently. No treatment-related SAEs have been reported.

In study MMV_OZ439_13_002, Cohort A, the frequency and severity of nausea and vomiting was higher than in Cohorts B and C in the same study and in other studies. The reason for the high level of gastrointestinal events in this study is unclear, however may be related to the taste of the TPGS formulation and the close proximity of the subjects dosed in Cohort A in the study. However, the tolerability observed in study MMV_OZ439_13_004 in which the bioavailability of the Phase IIb formulations was evaluated, was considered acceptable.

In the Phase IIa study in Asian patients, OZ439 (alone) was well tolerated, with no particular pattern of AEs was discernible. One patient on active treatment was withdrawn due to an event of vaso-vagal syncope that was considered to be treatment-related. No treatment-related SAEs have been reported.

3.5. Study Rationale

OZ439 has demonstrated acute efficacy (up to 36 hours post-dose) against *Plasmodium falciparum* and *Plasmodium vivax* malaria in a proof of concept (PoC) study in adult Asian patients (MMV_OZ439_10_002) with an estimated PRR48 of approximately 3, leading to a 97.9% reduction in parasite concentrations by 36 hours for all doses investigated (200 to 1200 mg). PK exposure was similar to that in healthy subjects.

PQP is a compound that is well established clinically, most recently in combination with dihydroartemisinin (DHA), Eurartesim® which is administered once daily for 3 days. The adult dose of *Eurartesim* contains 960 mg PQP. The formulations used in the planned study achieved exposures in the range of those published for PQP (Tarning *et al* 2012).

PK/PD modelling suggests that a single dose of OZ439 plus PQP administered in combination may be efficacious.

This study investigates the efficacy exposure-response of OZ439/PQP combination in the target populations and if it meets its efficacy objectives, will inform dose setting for Phase III studies.

3.6. Study Population Rationale

The majority of deaths due to *P. falciparum* malaria occur in young children, of around 5 years or less in Africa and this high death rate is believed to be partly linked to low immunity levels to the parasite coupled with greater vulnerability to the infection. In addition, older patients in geographical regions in which *P. falciparum* malaria is seasonal e.g. Asia and Latin America are believed to have lower immunity than patients greater than 5 year in highly endemic areas of Africa, where immunity is known to be greater.

Thus the study aims to recruit predominantly patient populations with the highest medical need i.e. patients of 5 years or less in Africa (60 to 80% of recruited patients). In addition, significant numbers of patients of all ages in Asia and Latin America will also be recruited (target 18 to 36% of recruited patients). Patients greater than 5 years in Africa will comprise approximately 10% of the patients (see Section 5.1 and 6.5 for further details).

3.7. Dose Rationale

Doses for this study were selected based on predicted efficacy and predicted QTc prolongation as discussed below.

As described in Section 3.3, PK/PD Summary, a concentration dependent relationship (PK/PD model) was established following administration of both OZ439 alone and PQP alone in the Malaria Challenge Model.

A range of dose combinations were 'tested', where a sufficient result was one where the predicted parasitaemia decreased by $>10^{11}$-fold on average for the lower 2.5th percentile of the population. Inclusion of the lower 2.5th percentile was to ensure that the predicted effect was to achieve efficacy of the drug combination for 97.5% of the population.

Doses were chosen to span an exposure-response for a predicted ACPR28 of >95% to <90%, in order to provide sufficient opportunity to determine the minimum effective dose combination.

Based on the above, the doses selected for this study and predicted PCR-adjusted ACPR28 (2.5 and 97.5th percentiles) are:

Dose OZ439/PQP formulation	Predicted PCR-adjusted ACPR28	2.5 and 97.5th percentiles
800mg/640mg	0.84	0.82 - 0.86
800mg/960mg	0.95	0.93 - 0.96
800mg/1440mg	0.99	0.97 - 1.00

For patients less than 35kg down to 5kg, doses will be adjusted by scaling clearance to achieve a similar predicted drug exposure as a 60kg patient (using the relationship CL = $(W/70)^{0.7}$ (Tarning *et al* 2012; See Section 10 3, Table 5 for further details).

In order to minimise the effects of PQP on QT prolongation, the highest well tolerated dose of OZ439 (800 mg) was chosen for all combinations in order to minimise the dose of PQP. The predicted ΔΔmean maximum QTcF was 13, 15 and 18ms for PQP doses of 640, 960 and 1440mg respectively.

OZ439 and PQ exposures in this study are predicted not to exceed the approximate exposures achieved in previous studies i.e. for OZ439 an approximate mean Cmax of 2000ng/mL and mean AUCinf of 29000ng.h/mL (associated with OZ439 doses of 800mg) and for PQ, an approximate mean Cmax of 600ng/mL and mean AUCinf of 30000ng.h/mL (associated with PQP dose of 1440mg, co-administered with OZ439 (PIB with milk) up to a dose of 800mg). These exposures have been demonstrated to have an acceptable safety and tolerability profile.

4. STUDY OBJECTIVES

4.1 Primary Objective

- To determine whether a single dose combination of OZ439/PQP is an efficacious treatment for uncomplicated *P. falciparum* malaria in adults and children

4.2 Secondary and Exploratory Objectives

- To evaluate the efficacy of OZ439/PQP:
  - To determine the incidence of recrudescence and re-infection
  - To determine the time to relief of fever and parasite clearance
- To further explore efficacy of OZ439/PQP:
  - To evaluate the proportion of patients with gametocytes at each assessment
  - To characterise gametocyte carriage
  - To examine the relationship between ACPR and exposure to OZ439/PQP (using logistic regression)
  - To determine parasite clearance kinetics
  - To examine the relationship between parasite genotypes of interest and parasite clearance kinetics/efficacy
- To evaluate the pharmacokinetics of OZ439/PQP:
  - To determine Cmax, Tmax and AUC of OZ439 and PQP in patients ≥35 kg

  - To characterise the pharmacokinetics and potential covariates in all patients (using population PK analysis)
- To evaluate the safety and tolerability of OZ439/PQP

5 OVERALL STUDY DESIGN

5.1 Study Design

A randomised, double-blind single-dose (loose combination) study in patients spanning the age range greater than or equal to 6 months to less than 70 years, with uncomplicated *Plasmodium falciparum* malaria. Three OZ439/PQP treatment arms will be included (for patients ≥ 35 kg), with doses scaled to target similar exposures in lighter patients (as mentioned above).

The study aims to recruit predominantly patient populations with the highest probability of having 'lower immunity' to *P. falciparum*, while also including patients with a probability of having ‘higher immunity’. Hence the study will recruit across a wide age range (see above) and across geographical regions (Africa, Asia and possibly Latin America). The underlying assumption is that children of 5 years or less in Africa and all ages in Asia/Latin America will have the lower immunity and hence potentially require the highest drug exposure to achieve effective treatment. For this reason, patients of 5 years or less in Africa will form the largest proportion of the population while patients of greater than 5 years in Africa will form the lowest proportion of the population (see Section 6.5 for further details).

The study will test for efficacy/futility at Day 28 (including data from patients discontinuing prior to Day 28) through interim analyses (see Figure 1 and Sections 8.3 and 11), using Bayesian methodology. Only data from patients in Asia/Latin America and patients of 5 years or less in Africa (the ‘lower immunity’ population; see Figure 1 and Section, 6.5, and 8.1.1) will be included in the interim analyses. Interim assessment of efficacy and futility will occur after recruitment of approximately 50 evaluable patients per dose cohort and thereafter depending on the rate of recruitment relative to the time required to perform the interim analyses..
Note the ISMB may request an early evaluation of the requirement for interim analysis to be performed before 50 patients per dose cohort have been recruited (see below).

Independently of the analyses for efficacy/futility, the safety of OZ439/PQP treatment arms will be assessed at scheduled time points by an Independent Safety Monitoring Board (ISMB).
Adults and children will be included through progressive step-down in age range following safety evaluation (see Figure 1 and Section 6.5 and 8.1.).

At approximately the same time as the first interim assessment for efficacy / futility, a preliminary PKPD analysis of the study data will be performed to assist clinical development. This additional modelling verification will not influence the conduct of the study.

The Independent Safety Monitoring Board (ISMB) can request an earlier first efficacy/ futility interim analysis (i.e. at <50 evaluable patients per arm) if there is evidence of

possible high treatment failure based on the data reviewed by the ISMB e.g frequency and timing of rescue medication administration.

Following Screening and informed consent, patients will receive study drug and will be followed for clinical signs of malaria (parasitaemia and temperature), safety assessments and pharmacokinetics up to Day 42 following dosing (Day 63 at selected sites).

5.2 Study Plan

Patients will be admitted to the Clinical Unit for Screening and if they fulfil the Inclusion/ Exclusion criteria and give informed consent (or assent where applicable), will be recruited to the study. Following drug administration, patients will remain in the Clinical Unit for a minimum of 48 hours post-dose (African patients greater than 5 years) or 72 hours post-dose (All Asian/Latin American patients and African patients ≤ 5 years) and for a maximum of 7 days (to Day 7) post-dose (all patients). All patients will have assessments on Day 3, 5 and Day 7 (patients discharged prior to Day 7 will return for assessment on Day 3, 5 and Day 7).

Discharge Criteria are given in Section 5.6.

Following discharge, patients will return to the Clinical Unit for further assessment on Days 3, 5, 7, 10, 14, 21, 28 and 42. At selected sites, patients will also return on Day 63 for assessment. Study days up to and including Day 14 must be performed on the specified correct day. Days 21 and 28 can be ± 2 days. Day 42 and 63 can be ± 3 days. Patients will have one additional safety check in the period between Day 14 and 21 and between Day 21 and Day 28. Patients can either return to the Clinical Unit for these tests or tests can be performed in the field by a trained person.

African adults and older African children (greater than 5 years) may be discharged from the Clinical Unit at 48 hours post-dose and all Asian/Latin American patients and younger African children (less than or equal to 5 years) may be discharged at 72 hours post-dose. Discharge is at the Investigators' discretion provided parasite and fever clearance has been achieved (see Section 5.6). Patients may remain in the Clinical Unit until Day 7 procedures are completed if more convenient.

5.3 Pre-dose Activities and Assessments

5.3.1 Informed Consent

The patient information and informed consent document will be used to explain the risks and benefits of study participation to the adult patient, or parent or guardian of the minor patient in simple terms before the patient can be entered into the study. Patients cannot undergo any study-related procedures until the informed consent form has been signed and personally dated by the patient, the patient's parent(s) or legally acceptable representative (LAR) if a minor, by an impartial witness (if the patient or patient's LAR is illiterate), and by the medically qualified Investigator. If an adult patient, or parent or LAR of a minor patient is illiterate then they may make their mark on the informed consent form and the form must be signed and personally dated by an impartial witness. The impartial witness must be a person, who is independent of the study, who cannot be unfairly influenced by people involved with the study, who attends the informed consent process if the patient or

the patient's LAR cannot read, and who reads the informed consent form and any other written information supplied to the patient. The informed consent document contains a statement that the consent is freely given, that the patient, or parent or guardian of the patient, is aware of the risks and benefits of entering the study, and that the patient is free to withdraw from the study at any time. Children will be asked to provide assent where appropriate. The age from which this will be sought will be defined by local legislation.

The Investigator is responsible for ensuring that informed consent is obtained from each legal representative for minors if required by local customs, and for obtaining the appropriate signatures and dates on the informed consent document. A copy of the signed and dated informed consent form will be given to the adult patient or minor patient's LAR. The signed and dated original consent form will be retained with the study records.

5.3.2 Screening

Patients will be assigned a Screening number and undergo Screening procedures to determine eligibility for the study. Screening procedures will include taking Informed Consent and assessment of eligibility according to the Inclusion/Exclusion criteria, full physical examination, collection of demography, medical history, prior and concomitant medication, pregnancy test (See Sections 2 and 7) and FSH (all women suspected of being post-menopausal). Samples for clinical laboratory safety tests, parasite counts and parasite species identification will be taken and single 12-lead ECG, vital signs and axillary temperature measurements.

Pregnancy Test should be confirmed negative, prior to concluding eligibility for the study.

For all Pregnancy Tests, where feasible a quantitative serum HCG test will be performed, failing which a qualitative serum HCG test will be performed, failing which a qualitative urine HCG test will be performed. A urine HCG test is the minimum acceptable test.

A Screening log, including Screening number, initials, date of birth and any subsequent reason(s) for exclusion from the study, will be completed for all patients who sign an Informed Consent Form.

5.3.3 Pre-dose Assessments

Prior to dosing, a full physical examination will be performed and signs and symptoms of malaria recorded. Where applicable, a pregnancy test should be confirmed negative, prior to dosing.

Triple 12-Lead ECG, single vital signs and temperature measurements, parasite counts (blood films), blood spot samples for qPCR, parasite genotyping (PCR correction and analysis of any parasite genotypes of interest associated with drug resistance), and blood samples for RT-PCR gametocyte detection and pharmacokinetic samples will be taken according to Section 7 (Patient Assessments) and Section 2 (Study Schedule of Assessments). Note, if insufficient bloodspot samples available at baseline, alternative blood spots collected as specified in the schedule of assessments may be used.

5.4 Post-dose Activities and Assessments

5.4.1 Treatment

This study involves the administration of a single loose combination dose of OZ439/PQP following a minimum of 3 hours fasting from food and milk.

Patients ≥ 35 kg will be randomised to one of 3 treatment arms and will receive one of 3 loose combination treatments. Patients < 35 kg will receive one of three, loose combination treatments predicted to achieve similar systemic drug exposure to that achieved in patients ≥ 35 kg (see Section 10.3, Dosage, Duration and Compliance).

Following drug administration, patients should be fasted ideally for 3 hours, although it is accepted that this may not be possible for young children.

If a patient vomits during dose, refer to Section 5.5.

5.4.2 Assessments post-dose up to and including Day 14

Physical examinations, assessment of signs and symptoms of malaria, clinical laboratory safety, parasite counts, blood spot samples for qPCR and parasite genotyping and blood samples for RT-PCR gametocyte detection, triple 12-lead ECGs, single vital signs and temperature measurements and pharmacokinetic samples will be taken up to Day 14 according to the Schedule of Assessments (See Section 2). Details of concomitant medication and adverse events should be collected. For details of the assessments including procedure, scheduled time-points, sequence of data recording and baseline definitions see Section 7. If a positive blood film is obtained (after initial parasite clearance), parasite genotyping will be performed by the central laboratory on the time-matched blood spot sample.

5.4.3 Assessments between Day 14 up to and including Day 28

Patients will attend the Clinical Unit on Days 21 and 28. Assessments will be performed according to the Study Schedule of Assessments (Section 2) and Section 7 (Patient Assessments).

If a positive blood film is obtained (after initial Parasite clearance), parasite genotyping will be performed by the central laboratory on the time-matched blood spot sample.

Details of concomitant medication and AEs should be collected.

An additional safety assessment will be made between Days 14 and 21 and between Days 21 and 28. Patients will either return to the Clinical Unit, or where feasible will have assessments made remotely to the Clinical Unit. If returning to the Clinical Unit, blood films (thick and thin) and temperature should be taken, and a blood sample for genotyping in the event of a re-emergence of parasites.

For patients remaining in the field, a Malaria Rapid Diagnostic Test (RDT) and temperature should be taken by a trained person. Patients feeling unwell, with increased temperature (axillary temperature ≥37.5 °C) and/ or a positive RDT should return to the Clinical Unit for assessments.

5.4.4 Assessments on Day 42 (and Day 63 at selected sites)

Patients will attend the Clinical Unit on Day 42 and at selected sites on Day 63 for Parasite counts, blood spots, assessment of malaria signs and symptoms and physical examination, vital signs and temperature. Assessments will be performed according to the Study Schedule of Assessments (Section 2) and Section 7 (Patient Assessments). Details of concomitant medication and AEs should be collected. If a positive blood film is obtained (after initial parasite clearance), parasite genotyping will be performed by the central laboratory on the time-matched blood spot sample.

5.5 Re-dosing in the Event of Vomiting

For patients <24kg i.e. receiving aqueous suspension which contains both OZ439 and PQP :

- If a patient vomits within 5 minutes of the start of dosing with the suspension, they should be re-dosed with a freshly prepared new suspension. A new 'individual subject treatment pack' should be assigned via IWRS.
- If vomiting occurs from 5 minutes to 35 minutes post start of dosing, the patient should continue to take the dose (if any is left), but should **not** be re-dosed.
- If a patient vomits after 35 minutes from the start of dosing, they should **not** be re-dosed.
- A re-dose should only be attempted once per patient.

For patients ≥24kg i.e. receiving aqueous suspension which contains OZ439 followed by PQP tablets:

- If a patient vomits within 5 minutes of the start of dosing with the suspension (but before taking any PQP tablets), they can be re-dosed with a freshly prepared new OZ439 suspension and then dosed with PQP tablets. A new 'individual subject treatment pack' should be assigned via IWRS.
- If vomiting occurs from 5 minutes to 35 minutes post start of administration of the OZ439 suspension, they should continue to take the remainder of the OZ439 suspension and the PQP tablets (if OZ439 and / or PQP dosing not completed), but should **not** be re-dosed.
- If a patient vomits after 35 minutes from the start of administration of the OZ439 suspension, they should **not** be re-dosed.
- A re-dose should only be attempted once per patient.

5.6 Discharge Criteria

- African study site patients (> 5 years) may be discharged at 48 hours post-dose at the Investigators discretion provided parasite and fever clearance have been achieved (see Section 7.7.2 and 7.5 respectively for definitions) and maintained for at least 24 hours. Patients may remain in the Clinical Unit until Day 7 procedures are completed if more convenient.

- All Asian/Latin American study site patients and African patients less than or equal to 5 years may be discharged at 72 hours post-dose at the Investigator's discretion provided parasite and fever clearance have been achieved (see Section 7.7.2 and 7.5

respectively for definitions) and maintained for at least 24 hours. Patients may remain in the Clinical Unit until Day 7 procedures are completed if more convenient.

5.7 Re-admission Criteria

For patients having assessments 'in the field', if axillary temperature is ≥37.5 °C and/or in the event of a positive RDT, or feeling unwell, patients must return to the Clinical Unit for assessment. Physical Examination, malaria signs and symptoms, blood films, blood spot samples for parasite genotyping, temperature, vital signs and a PK sample should be taken if re-emergence of parasitaemia is suspected. Other assessments may be made as deemed appropriate by the Investigator and should be documented as an unscheduled visit in the eCRF. An unscheduled visit can be performed at any time at the Investigator's discretion.

5.8 Circumstances for Established Anti-Malaria Treatment

Established malaria treatment will be administered in the following circumstances:

- At 24hours post dose:
    - For patients with baseline parasitaemia <40,000 parasites/uL:
      Increase of 5-fold relative to base line or >60,000 parasites /uL, whichever is the highest.
    - For patients with baseline parasitaemia >40,000 < 70,000 parasites/uL:
      Increase of 2-fold relative to base line or >100,000 parasites /uL, whichever is the highest.
    - For patients with baseline parasitaemia >70,000 parasites/uL:
      Increase of 1.5-fold relative to base line or >120,000 parasites /uL, whichever is the highest.
- At 48 hours post dose: parasitaemia higher than baseline parasitaemia irrespective of axillary temperature.
- At 72 hours post dose: more than 25% of the baseline parasitaemia irrespective of axillary temperature or any detectable parasitaemia with axillary temperature ≥37.5°C.
- At 96 hours post dose: failure to achieve parasite clearance irrespective of axillary temperature.
- At any time: danger signs of severe malaria with parasitaemia.
- On recrudescence or re-infection at any time up to Day 42 (Day 63 at selected sites)

Blood films and blood sampling for qPCR, parasite genotyping and RT-PCR gametocyte detection must be taken before giving the Established Anti-malarial Treatment. Following administration of Established Anti-malarial Treatment, patients should be managed according to local standard of care practices. Safety data should continue to be collected in the eCRF, according to the study schedule, and up to the end of the study even if re-emergence is confirmed. Blood films and blood sampling for qPCR and parasite genotyping should not be entered into the eCRF after an established anti-malarial is given.

5.9 Blinding and Randomisation

Once written informed consent has been given, the patient will be given a unique Screening number.

On Day 0, all eligible patients will be randomised via the Interactive Web Response System (IWRS) 1:1:1 to one of three treatment arms. The Investigator or his/her delegate will contact the IWRS after confirming that the patient fulfils all the inclusion/exclusion criteria. The IWRS will assign a unique patient identification (ID) number which is associated in the IWRS system with the treatment arm to which the patient has been assigned. The IWRS system will specify individual subject treatment pack number for the package of study treatment to be prepared for the patient. All treatment assignments will be blinded and concealed from patients and all Investigator staff. Since only the PQP dose differs, the packaging for this will be masked and the labelling will not indicate the dosage in order to maintain the blind of the staff preparing the drug.
A patient randomisation list will be produced by the IWRS provider using a validated system. Each patient randomisation number is associated with one of the treatment arms. Patients who discontinue participation will not be replaced.

5.10 Endpoint

5.10.1 Primary Efficacy Endpoint

PCR-adjusted adequate clinical and parasitological response (ACPR) at Day 28.

5.10.2 Secondary and Exploratory Endpoints

- Secondary Efficacy Endpoints
  - PCR - adjusted ACPR at Day 42 and 63*.
  - PCR - crude ACPR at Day 28, 42 and 63*.
  - Kaplan Meier presentation for incidence rate of re-emergence, recrudescence and re-infection at Day 28, 42 and 63*.
  - Parasite clearance time (PCT).
  - Fever clearance time (FCT).
  - PRR.

- Exploratory Endpoints
  - Kaplan-Meier presentation of the risk of having gametocytes for:
    - Patients with gametocytes at baseline to time to clearance of gametocytes
    - Patients with no gametocytes at baseline to time to appearance of gametocytes
  - Integrated number of gametocytes (AUC) at 28 and 42 days for (pre-specified endpoints calculated and reported outside of CSR):
    - Patients with gametocytes at baseline.
    - Patients with no gametocytes at baseline that develop gametocytes during the study.
  - Correlation between response (ACPR at Day 28 and 42) and exposure (Day 7) to OZ439/PQP
  - Correlation between Kelch-13 genotype status and additional parasite genotypes of interest that may be identified, and parasite clearance kinetics/efficacy.

- Exposure response evaluation including pharmacokinetic / pharmacodynamics modelling

- Pharmacokinetic Endpoints
  - Non-compartmental analysis (NCA) of concentrations of OZ439 and piperaquine:
    - Performed on patients whose PK data describe the Cmax and elimination phase**.
    - Parameters are: Cmax, Tmax, AUC, CL/f, elimination half-life ($t_{1/2}$)**.
    - Concentration at Day 7, 14, 28, 42 and 63* (summarised from patients who have those time points sampled)**.
  - Non-linear mixed effect modelling (pre-specified endpoints calculated and reported outside of CSR)
    - Performed across all patients.
    - An extension of existing PK models - where weight is a dosing covariate.
      - Including analysis of covariates for allometric scaling by weight, gender, disease, race, site effects and others if deemed relevant.

- Safety and Tolerability Endpoints
  - Incidence of adverse events.
  - Laboratory variables including change from baseline.
  - Haemoglobin drop
    - Hb drop > 2 g/dL from baseline
    - Hb ≤ 5g/dL
  - Absolute Neutrophil count < 1000/µL
  - Proportion of patients meeting the Hy's law definition (see Section 9.1).
  - LFT changes:
    - Any ALT or AST ≥5x ULN
    - Any AST or ALT ≥3x ULN with the appearance of fatigue, nausea, vomiting, right upper quadrant pain or tenderness, fever, rash and/or eosinophilia (eosinophil percent or count above the ULN)
    - ALT ≥3x ULN persisted for >4 weeks

  - Clinically significant ECG abnormalities including incidence of QT/QTc as follows:
    - QT/QTc < 450 ms.
    - 450 ms ≤ QT/QTc < 480 ms.
    - 480 ms ≤ QT/QTc < 500 ms.
    - QT/QTc ≥ 500 ms.

    And change from baseline in QT/QTc:
    - < 30 ms increase from baseline.
    - ≥ 30 ms and < 60 ms increase from baseline.
    - ≥ 60 ms increase from baseline.
  - Vital signs including change from baseline.
  - Physical examination and clinical signs and symptoms related to uncomplicated *P. falciparum* malaria (Fever, Dizziness, Headache, Nausea, Anorexia, Vomiting, Diarrhoea, Itching, Urticaria, Skin Rash, Abdominal Pain, Joint Pain, Muscle Pain,

Palpitations, Sleep Problems, Confusion, Hearing Problems, Vision Problems, Fatigue).

** Data at Day 63 collected at selected sites only.*
*** Patients <35kg will have 'sparse sampling' and may have insufficient data.*

6 SELECTION OF PATIENTS

6.1 Number of Patients

The study will be performed using interim assessments of efficacy/futility. The estimated number of patients that will be required to demonstrate exposure-response is 120 patients per treatment arm.

However to allow for potentially higher variability, the target maximum number of patients recruited will be 165 per treatment arm consisting of 150 patients from the 'exposure-response' population i.e. populations expected to have 'lower' immunity (i.e. African patients less than and equal to 5 years and all Asian/ Latin American patients) who will be included in the interim analyses for efficacy/futility and an approximate additional 15 African patients greater than 5 years who will not be included in the interim analyses for efficacy/futility. Recruitment would be capped at 150 plus approximately 15 patients per treatment arm.

6.2 Recruitment Methods

Patients will be recruited following locally acceptable procedures.

6.3 Inclusion Criteria

1. Male or female patient age $\geq$6 months <70 years.
2. Body weight $\geq$5 kg $\leq$90 kg
3. Presence of mono-infection of *P. falciparum* with:
   a. Fever, as defined by axillary temperature ≥ 37.5°C or oral/rectal/tympanic temperature ≥ 38°C, or history of fever in the previous 24 hours (history of fever must be documented) and,
   b. Microscopically confirmed parasite infection, in range 1,000 to 100,000 asexual parasites /µL of blood.
4. Written informed consent provided by the adult patient, or parent or legally acceptable representative (LAR) of the minor patient or by an impartial witness (if the patient or patient’s LAR is illiterate), stating that the information has been read and/or is understood, and by the medically qualified Investigator. Children will be asked to provide assent where appropriate. The age from which this will be sought will be defined by local legislation.

6.4 Exclusion Criteria

1. Presence of severe malaria (according to WHO definition – WHO 2013)
2. Anti-malarial treatment:
   a. With piperaquine -based compound, mefloquine, naphthoquine or sulphadoxine/pyrimethamine (SP) within the previous 6 weeks (after their inhibition of new infections has fallen below 50%).
   b. With amodiaquine or chloroquine within the previous 4 weeks.

   c. With quinine, halofantrine, lumefantrine, artemisinin-based compounds and any other anti-malarial treatment or antibiotics with anti-malarial activity (including cotrimoxazole, tetracyclines, quinolones and fluoroquinolones, and azithromycin) within the past 14 days.

For other concomitant medication restrictions see Section 7.4

3. Known history or evidence of clinically significant disorders such as, cardiovascular (see 4, 5, 6 & 7 below), respiratory (including active tuberculosis), hepatic, renal, gastrointestinal, immunological (including active HIV-AIDS), neurological (including auditory), endocrine, infectious, malignancy, psychiatric, history of convulsions or other abnormality (including head trauma).
4. Family history of sudden death or of congenital or clinical conditions known to prolong QTcB or QTcF interval or e.g. family history of symptomatic cardiac arrhythmias, with clinically relevant bradycardia or severe cardiac disease.
5. History of symptomatic cardiac arrhythmias or with clinically relevant bradycardia or with severe cardiac disease.
6. Any predisposing cardiac conditions for arrhythmia such as severe hypertension, left ventricular hypertrophy (including hypertrophic cardiomyopathy) or congestive cardiac failure accompanied by reduced left ventricle ejection fraction.
7. QTcB or QTcF >450ms at Screening (Note patients with QTcB or QTcF >450ms pre-dose should be withdrawn prior to dosing)
8. Electrolyte disturbances, particularly hypokalaemia, hypocalcaemia or hypomagnesaemia.
9. Any treatment which can induce a lengthening of QT interval, such as:
   a. Antiarrhythmics (e.g. amiodarone, disopyramide, dofetilide, ibutilide, procainamide, quinidine, hydroquinidine, sotalol),
   b. Neuroleptics (e.g. phenothiazines, sertindole, sultopride, chlorpromazine, haloperidol, mesoridazine, pimozide, or thioridazine),
   c. Anti-depressive agents, certain antimicrobial agents, including agents of the following classes macrolides (e.g. erythromycin, clarithromycin), fluoroquinolones (e.g. moxifloxacin, sparfloxacin), imidazole and triazole antifungal agents, and also pentamidine and saquinavir,
   d. Certain non-sedating antihistamines (e.g. terfenadine, astemizole, mizolastine), cisapride, droperidol, domperidone, bepridil, diphemanil, probucol, levomethadyl, methadone, vinca alkaloids, arsenic trioxide.
   e. Anti-emetics with known QT prolongation potential such as domperidone
10. Mixed *Plasmodium* infection
11. Severe vomiting, defined as more than three times in the 24 hours prior to enrolment in the study or inability to tolerate oral treatment, or severe diarrhoea defined as 3 or more watery stools per day
12. Severe malnutrition (defined for subjects aged ten years or less as the weight-for-height being below -3 standard deviation or less than 70% of median of the NCHS/WHO normalised reference values, and for subjects aged greater than ten years, a body mass index (BMI) of less than 16 (WFP Manual, Chapter 1)).
13. Known history of hypersensitivity, allergic or adverse reactions to piperaquine or other aminoquinolines or to OZ439 or OZ277
14. Known active Hepatitis A IgM (HAV-IgM), Hepatitis B surface antigen (HBsAg) or Hepatitis C antibody (HCV Ab).
15. If Total Bilirubin is normal, exclude the patient if liver function tests AST/ALT ≥ 2xULN.

16. If Total Bilirubin is > 1 and ≤ 1.5xULN, exclude the patient if AST/ALT >1.5xULN.
17. Total Bilirubin > 1.5XULN
18. Haemoglobin level below 8 g/dL.
19. Serum creatinine levels ≥2 x ULN
20. Female patients of child bearing potential must be neither pregnant (as demonstrated by a negative pregnancy test) nor lactating, and must be willing to take measures not to become pregnant during the study period and safety follow-up period.
21. Have received an investigational drug within the past 4 weeks.
22. Previous participation in any malaria vaccine study or received malaria vaccine in any other circumstance.
23. Refusal to participate and to provide written or witnessed informed consent or assent.

6.5 Patient Population

Lower Immunity Population
This is the target population of interest. The study aims to recruit predominantly patients with lower potential for immunity defined as all age groups ≥ 6 months to < 70 years in Asia or Latin America and children ≥ 6 months to ≤ 5 years in Africa. Only patients from this population will be included in the interim analyses of efficacy/futility.

Higher Immunity Population
Patients with higher potential for immunity will also be recruited (African patients >5 years to <70 years).

6.5.1 Patient Sub-populations

- African Patients > 5 years old:
  - Target of an additional 10% to the patients required to demonstrate efficacy. The target minimum and maximum patients per treatment arm are 12 and 15 respectively (based on a total of 120 to150 patients per arm for the efficacy/futility analysis), (i.e. 36 and 45 patients total).

This will give a total maximum number of patients (assuming no treatment arm is dropped 'early' for confirmed efficacy or futility) of 132 to 165.

- African Patients ≤ 5 years old :
  - A targeted minimum of 60% of total number of patients recruited to the lower immunity population i.e. approximately 79 to 99 patients per treatment arm (if total numbers per arm are 132 to165)

- African Patients ≤ 2 years old :
  - A targeted minimum of 10% of the total number of patients recruited i.e. approximately 13 to17 patients per treatment arm (if total numbers per arm are 132 to 165).

- Asian/Latin American Patients:

- A target of 18% of total number of patients recruited i.e. approximately 24 to 30 patients per treatment arm (if total numbers per arm are 132 to165).

- Maximum of 36% of total number of patients recruited i.e. approximately 48 to 60 patients per treatment arm (if total numbers per arm are 132 to 165).

6.5.2 Age Range Step-down Procedure

Safety (up to Day 14 assessments) in the older age range (from Africa and Asia/ L. America) will be assessed before proceeding down to the younger age range (see Figure 1 and Section 8).

- 30 patients (>15 years) will be assessed for safety up to Day 14 before opening recruitment in the > 5 and ≤ 15 years age range.

- 20 additional patients (> 5 to ≤ 15 years) will be assessed for safety up to Day 14 before opening recruitment in the > 2 and ≤ 5 year age range.

- 20 additional patients (>2 to ≤ 5 years) will be assessed for safety up to Day 14 before opening recruitment in the ≥ 6 month to ≤2 year age range

The safety evaluation will be performed by an Independent Safety Monitoring Board (ISMB).

6.6 Patient Restrictions

- Able to remain in the clinic for a minimum of 48 to 72 hours (dependent of age, geographical region and parasitaemia).
- Able to attend post discharge visits according to the protocol up to Day 42 (Day 63 at selected sites).
- Food (including milk) excluded from 3 hours prior to dosing, ideally for 3 hours post dose.
- Concomitant medications prohibited.
- Drugs of abuse prohibited.
- Alcohol intake restricted (particularly within 3 days of treatment)
- Contraception instructions.

6.7 Contraceptive Requirements for Male and Female Patients

Female patients must be willing to take measures not to become pregnant for the three months following enrolment in the study, and pregnancies occurring up to study Day 42 should be reported.

All sexually active male patients must agree to measures to prevent pregnancy in their partners for the three months following enrolment in the study through the effective use of barrier contraception. Any pregnancies in female partners occurring up to study Day 42 should be reported.

7 PATIENT ASSESSMENTS

7.1 Demographic and Baseline Variables

At Screening, demographic information will be recorded as follows:

Date of birth; gender; if female, childbearing potential; race; weight (kg); Height (m); and Malaria history over the last 12 months (0, 1 or >1).

7.2 Medical History

At Screening, confirm the presence of uncomplicated symptomatic mono-infection *P. falciparum* as indicated in the inclusion criteria. Record relevant Medical History including diagnostic results, condition or surgery, (drug) allergies and relevant physical examination findings.
Signs and symptoms of uncomplicated malaria should be recorded: Fever, Dizziness, Headache, Nausea, Anorexia, Vomiting, Diarrhoea, Itching, Urticaria, Skin Rash, Abdominal Pain, Joint Pain, Muscle Pain, Palpitations, Sleep Problems, Confusion, Hearing Problems, Vision Problems, Fatigue.

History of clinically significant disorders such as cardiac, respiratory (including active tuberculosis), hepatic, renal, gastrointestinal, immunological, neurological (including auditory), endocrine, infectious, malignancy, psychiatric, convulsions or other abnormality (including head trauma).

7.3 Prior Medication

Record prior medication including anti-malarial treatment and antibiotics taken within the timeframes specified in Section 6.4. Ensure that no excluded medications have been taken in line with the restrictions in Exclusion Criteria (Section 6.4) and Section 7.4 below.

7.4 Concomitant Medication

Concomitant medication is defined as any medication, other than the Investigational Medicinal Product (IMP), which is taken during the study, including prescription and over-the-counter medicines, and any traditional or herbal remedies.

The only additional medication likely to be given will be paracetamol (15 mg/kg 4 hourly as required) as an antipyretic, and metopimazine for repeated vomiting (or if not available, any other antiemetic which is not known to prolong QT and/or cause torsade de pointes).

Metoclopramide is contraindicated from the period prior to screening to Day 5 post-dose (120 hours).

Beta-lactam antibiotics can be given in case of a bacterial infection appearing after enrolment. All the other antibiotics, new-quinolones included, should be avoided where possible. All concomitant medication taken while the patient is participating in the study will be recorded.

Excluded medication (both in the period prior to Screening and during the patient's participation) is listed below:

4-Aminoquinolines chloroquine and piperaquine
Arylaminoalcohols quinidine
Quinine
Mefloquine
9-phenanthrene methanol halofantrine

Dibutyl-amino ethanol lumefantrine
Artemisinin derivatives artemether, arteether, artesunate and dihydroartemisinin
Antimetabolites proguanil
Chlorproguanil
Methotrexate and other folate antagonists
Sulfalene
Pyrimethamine
Sulfonamides sulfadoxine
Sulfisoxazole
Sulfadiazine
Sulfasalazine
8-Aminoquinolines primaquine, tafenoquine
Hydroxynaphtolquinone atovaquone
Antibiotics, various classes doxycycline
Antibiotics, various classes new quinolones
Azythromycin
Erythromycin
Pentamidine
Clindamycin
Rifampin
Dapsone
Combinations of sulfadoxine pyrimethamine
Trimethoprim sulfamethoxazole
Atovaquone
Pyronaridine
Metoclopramide (period prior to screening to Day 5 post-dose (120 hours)).

Traditional and herbal remedies are not permitted from 7 days prior to dosing and during the study.

The patient must not participate in any other clinical study of an investigational product or device whilst participating in this study.

Where time-points coincide, body temperature, vital signs and ECG measurements should be performed prior to any blood sampling. The PK sample should be taken at the nominal time.

7.5 Body Temperature

Single measurements of axillary temperature will be measured at Screening, pre-dose (within 1 hour of dosing) and at the following time points post-dose: 1, 2, 6, 12, 18, 24, 30, 36, 48 and 72 (± 10 minutes for first 24 hours, then ± 30 minutes up to 72 hours) and at Day 5, 7, 10, 14, 21, 28, 42 and 63* (Day 21 and 28 can be ± 2 days, Day 42 and 63 can be ± 3 days). Body temperature measurements will be taken prior to any blood sampling. Between Days 14 and 21 and Day 21 and 28, one measurement of body temperature may be taken 'in the field', or at the Clinical Unit.

Additional (unscheduled) measurements may be taken if required to confirm Fever Clearance as described below.

Axillary temperature should be recorded in degrees Celsius (°C) and to an accuracy of one decimal place. If the axillary method is not possible, an alternative route (oral, tympanic, rectal) may be used. The alternative route shall be recorded in the eCRF. Within an individual patient the same method of temperature measurement (axillary, oral, tympanic, rectal) should be used throughout the entire study period.

Fever Clearance

Fever clearance is defined (in patients with an increased temperature at baseline) as the time of the first measurement of axillary temperature of <37.5 °C (or <38.0 °C for alternative routes). This measurement must be confirmed by a second measurement, taken within 6 to 12 hours of the first. Fever clearance will be concluded following confirmation of temperature <37.5 °C on the second measurement.

Patients entered in the study on the basis of history of fever and who do not subsequently have an increased body temperature measurement indicating presence of fever pre-dose, will not be included in the analysis of fever clearance time.

7.6 Vital Signs (Blood Pressure / Pulse Rate)

Single measurements of blood pressure (systolic/diastolic) and pulse rate will be taken after the patient has rested supine for at least 10 minutes, and prior to any blood sampling. Measurements will be taken at Screening/pre-dose (within 1 hour of dosing) and at the following time-points: 2, 6, 12, 24, at discharge (48 or 72) hours (± 10 minutes) post-dose and on Days 7, 14, 21, 28, 42 and 63*. (Day 21 and 28 can be ± 2 days, Day 42 and 63 can be ± 3 days). (* At selected sites)

7.7 Parasitaemia (Thick and Thin Blood Films)

Blood sampling for parasitology can be done usually by means of finger prick except when the timing for parasitology assessments coincide with time for clinical laboratory tests, in which case, blood films can be taken from the venous blood collected for clinical laboratory analyses. Blood spot samples will also be taken for qPCR, genotyping and Kelch-13 analysis and a blood sample for RT-PCR gametocyte detection (see sections 7.8 and 7.9).

For full details of slide preparation, determination of parasitaemia and QC, refer to the Study Procedures Manual.

7.7.1 Screening and Pre-dose Blood Films

For parasitology, Screening and pre-dose are to be considered as one time point. Three slides (two thick films and one thin film) should be prepared in this period for this time point and should be from samples taken within 4 hours of dosing.

The first thick film slide is to be rapidly stained with 10% Giemsa stain for a period of 10 to 15 minutes. The parasite count from this slide is then used to calculate the Screening parasitaemia value.

The second thick film slide must be used only if the patient meets all entry criteria and is being recruited into the study. This second thick film slide should be stained with 2.5 to 3% Giemsa stain for a period of 45 to 60 minutes. The slide from this slower but more

accurate staining technique should be used to calculate more accurate pre-dose parasitaemia counts (asexual and gametocytes).

The thin film slide is used specifically for parasite speciation. Only patients with *P. falciparum* mono-infection should be recruited to the study.

7.7.2 Post-dose Blood Films

Three slides (two thick films and one thin film) should be taken at each time point. The first thick film slide should be stained with 2.5 to 3% Giemsa stain for a period of 45 to 60 minutes and used to determine parasite counts. The second thick film slide should be stained and kept as contingency should the first thick film slide become damaged prior to reading it. The thin film slide is used specifically for parasite speciation which should be recorded at each time point.

Thus for patients ≥35kg: thick (x2) and thin (x1) blood films will be prepared from blood samples prepared at Screening / pre-dose (as described above) and at 6, 12, 18, 24, 30, 36, 48 and 72 hours post-dose (± 10 minutes for 24 hours then ± 30 minutes until 72 hours). For patients <35kg samples will be prepared at Screening/pre-dose, 6, 18, 36, 48 and 72 hours only. For all patients, samples will be prepared at Day 5, 7, 10, 14, 21, 28 and 42. If parasites have not cleared by 72 hours, blood films should continue to be taken according to site standard practice (or at minimum every 8 hours) until parasite clearance is shown or the criteria for rescue medication are met (see section 5.8). If these additional blood smears are taken they should be recorded as unscheduled visits in the eCRF.

Films will also be taken at Day 63 at selected sites. Day 21 and 28 can be ± 2 days and Day 42 and 63 can be ± 3 days. Additional unscheduled films may be taken to confirm Parasite clearance as described below.

Details of slide preparation, staining, examination, calculation methods and quality control are given in the Study Procedures Manual. No deviation from the Sponsor guidelines will be accepted.

During the intervening periods between Day 14 and 21 and Day 21 and 28, blood films may be taken in the Clinical Unit, or alternatively a rapid diagnostic test (RDT) may be used 'in the field'.

Definition of Parasite Clearance (by Microscopy)

A blood film will be considered 'negative' when the examination of 1000 white blood cells reveals no asexual parasites (see Study Procedures Manual for more detail). Parasite clearance time is defined as the time of the first negative film. This negative film must be confirmed by a second negative film, taken within 6 to 12 hours of the first. Parasite clearance will be concluded following confirmation of the second negative film. Parasite density, expressed as the number of parasites per microlitre (µL) of blood, for asexual parasite and for gametocyte counts will be recorded separately throughout the study.

Two qualified microscopists should independently read one of the thick film slides and parasite densities will be calculated by averaging the two counts. Blood smears with non-concordant results (differences between the two microscopists in species diagnosis, or differences in parasite density of > 50%) will be re-examined by a third, independent

microscopist and parasite density will be calculated by averaging the two most concordant counts.

7.8 Dry Blood Spot Samples for Quantitative PCR, Parasite Genotyping (PCR Correction) and Analysis of Parasite Genotypes of Interest that may be Associated with Drug Resistance

Parasite genotyping (PCR Correction):
Genotyping at the central laboratory will be performed at the following time points: on the pre-dose and 18 or 24 hour samples, and the sample corresponding to the time point when recrudescence or re-infection after initial parasite clearance was shown on the blood slide. Therefore, for patients with body weight ≥35kg - Screening/pre-dose and at 24, 30, 36, 48 and 72 hours post-dose (± 10 minutes for 24 hours then ± 30 minutes until 72 hours). and at Day 5, 7, 10, 14, 21, 28, 42 and 63 (Day 21 and 28 can be ± 2 days and Day 42 and 63 can be ± 3 days).
For patients <35kg, the schedule up to 72 hours will be Screening/pre-dose, 18, 36, 48 and 72 hours post-dose, thereafter the same as the adults.
During the intervening periods between Day 14 and 21 and Day 21 and 28, blood for genotyping will only be taken if the patient returns to the Clinical Unit for assessment. A genotyping blood sample will not be taken if the patient has a RDT 'in the field'. Detail of the processing and technique will be provided in the Study Procedures Manual.

qPCR analysis:
Blood spot samples for qPCR and genotyping will be applied to the same card, according to the Schedule of Assessments (Section 2) and the instructions within the Study Procedures Manual and sent to the central laboratory for analysis. qPCR analysis will be performed by the central laboratory only on samples from patients ≥ 35kg at each timepoint up to and including 72 hours. Therefore, for patients with body weight ≥35kg - Screening/pre-dose and at 6, 12, 18, 24, 30, 36, 48 and 72 hours post-dose (± 10 minutes for 24 hours then ± 30 minutes until 72 hours). Thus parasitaemia by qPCR will be measured from pre-dose to 72 hours, and in line with the measurement of parasitaemia by blood film.

Kelch 13 and additional parasite genotypes associated with drug resistance:
An analysis of the Kelch-13 and additional parasite genotypes of interest associated with drug resistance will be performed on dry blood spot samples.

7.9 Gametocyte Detection (RT-PCR)

Blood samples for RT-PCR gametocyte detection will be taken only from patients ≥ 35kg from screening up to and including 72 hours, and at Day 7. Samples will be taken into EDTA microtainers and mixed with RNAprotect prior to analysis by the central laboratory. Samples will be taken according to the Schedule of Assessments and the instructions within the Study Procedures Manual.

Thus samples for RT-PCR gametocyte detection will be measured at the time intervals shown in Section 2, and in line with the measurement of asexual and gametocyte parasite counts.

Therefore, for patients with body weight ≥35kg - Screening/pre-dose and at 6, 12, 18, 24, 30, 36, 48, 72 hours and on Day 7 post-dose (± 10 minutes for 24 hours then ± 30 minutes until 72 hours).
Detail of the processing and technique will be provided in the Study Procedures Manual.

7.10 Malaria Rapid Diagnostic Test

A malaria rapid diagnostic test (RDT) may be used by trained study personnel in the field when following up patients discharged from the Clinical Unit. The purpose of the test is to screen for *Plasmodium* infection, occasioned either by recrudescence or re-infection. Patients with a positive RDT must be referred to the study site for further investigation and management. Rapid diagnostic tests must be used in accordance with the manufacturers´ instructions, and study personnel using these tests must undergo documented training in their use, including awareness of the time period since parasite clearance in which these tests remain positive. A negative test result in a patient should not preclude referral to the Clinical Unit in instances where the study personnel believe malaria may be responsible for the patient´s illness. The RDTs used must have a detection rate at >200 parasites/µL of >97%, details will be specified in the Study Procedures Manual. Blood is collected for the RDT via a lancet and hence the volume required is minimal.

7.11 Physical Examination and malaria signs and symptoms

A standard full physical examination will be performed at Screening/pre-dose and 28. Only significant changes in Physical Examination findings will be recorded at: 12, 24, 36, 48 and 72 hours post-dose, and at Days 7, 42 and 63. Any change of concern should be recorded as an adverse event.

The Physical Examination will include: general appearance, head and eyes, ears, nose and throat, chest and lungs, cardiovascular, abdomen, neurological, lymphatic and musculoskeletal and any additional body system considered of relevance by the Investigator.

A full assessment of malaria signs and symptoms will be made alongside the physical examination. Therefore at Screening/pre-dose, 12, 24, 36, 48 and 72 hours post-dose and at Days 7, 28, 42 and 63.

7.12 12-Lead ECG

Patients should rest supine for 10 minutes prior to taking an ECG. All ECG measurements will be taken prior to any blood sampling. A single 12-lead ECG will be recorded at Screening and triplicate12-lead ECG will be obtained pre-dose (within 1 hour prior to dosing) and at 2, 6, 12, 24, discharge (48 or 72) hours post-dose, and at Day 7.

Care must be taken to ensure that the leads are placed in the correct place. ECG recordings will be taken after the patient has been supine for at least 10 minutes and triplicates will be measured within 5 minutes of the first measurement of the triplicate.

In Study Calculation of QTc Parameters
Mean values, calculation of correction factors (e.g. Fridericia) and calculation of QTc increase should be according to standard practice at the site.

Each ECG should be inspected at the time of collection to ensure that it is of sufficient quality for interpretation (i.e. there is minimum artefact). If the quality is not adequate the recording should be repeated so that three recordings of acceptable quality are obtained. These should be transmitted to the central ECG reviewer.

All ECG recordings should be read and interpreted by the Investigator. The Investigator should assess whether there are clinically significant changes from baseline including prolongation of QTc and abnormalities of rhythm, and if so, record the findings on the eCRF. Any change of concern should be recorded as an adverse event. The ECG central reviewer may provide assistance with this interpretation on request.

Any ECG with an increase in QTcB or QTcF > 30 ms or any abnormality of rhythm will be sent to the central reviewer for a report. A report on the central review will be sent back to the Clinical Unit and will help the Investigator to determine if an AE has occurred and to inform the management of the patient.

Abnormal ECGs at both Screening and pre-dose should be considered in terms of whether the patient is eligible for the study. Patients with a QTcB or QTcF greater than 450 ms or clinically significant abnormalities of rhythm at Screening are not eligible. Patients with a pre-dose baseline value > 450 ms should be withdrawn from the study prior to dosing.

Additional ECGs should be collected as judged by the Investigator. Clinical indications for further ECG recordings would be the finding of obvious abnormalities of rhythm, prolonged QTc (> 450 ms) and/or obvious change from the previous ECG or if the patient experiences cardiac-type symptoms such as chest pain, palpitation, light-headedness, syncope or shortness of breath.

[Note, the QTc parameters in the final analysis will be calculated from the raw data received by the central reviewer. This process will be detailed in the Statistical Analysis Plan.]

One copy of each recording should be made. Further details can be found in the ECG Procedures Manual.

7.13 Laboratory Safety Evaluations

Standard blood clinical laboratory tests are listed in Table 3 below. Clinical laboratory tests (haematology and clinical chemistry) should be performed at Screening and at 48 and 72 hours post-dose and at Days 5, 7, 14 and 28. For children ≤5 years the schedule may be reduced to: Screening and Day 2, 7, 14 and 28. D3 should be done if results are abnormal at D2, and D5 should be done if results are abnormal on D3.

One (1) mL of whole blood will be required for haematology tests and 1 mL for clinical chemistry tests. The tubes should be labelled with the patient ID number, protocol scheduled timepoint, date, time and type of sample. Full details of processing will be supplied in the Study Procedures Manual. If required tests cannot be performed using these minimal volumes due to machine restrictions in local areas, local protocol amendment or clarifications will be written.

Table 3: Clinical Laboratory Blood Tests

**Clinical Chemistry (serum):**
Total bilirubin (also Direct, when total bilirubin is ≥ ULN)
Albumin
Alanine aminotransferase (ALT)
Aspartate aminotransferase (AST)
Plasma haptoglobin
LDH
Creatine kinase
Alkaline phosphatase (ALP)
Urea
Creatinine
Sodium
Potassium
Magnesium
Calcium
Glucose
Pregnancy test (plasma/serum HCG) for females
FSH for females (where required)
**Haematology:**
Haematocrit
Haemoglobin
Absolute reticulocytes
Erythrocyte count (RBC)
Platelet count
Leukocytes (WBC) with differential count including eosinophils
**Urinalysis Dipstick:**
Specific gravity, pH, glucose, protein, bilirubin, ketones, leukocytes and blood
**Urinalysis Microscopy:**
WBC, RBC

Laboratory values that fall outside a clinically accepted reference range must be evaluated by the Investigator and graded according to the DMID Toxicity Scale for Determining Severity of Adverse Events (Attachment 1). Clinically significant abnormalities that represent a change from baseline and could be considered to be detrimental are to be recorded as Adverse Events. It is anticipated that a fall in haematological parameters such as haemoglobin may occur during treatment. A fall of up to 2 g/dL need not be reported as adverse events unless the fall is considered to be treatment related and/or detrimental to the patient. Detrimental changes in differential white cell count (other than clinically significant falls in neutrophils) should only be reported as adverse events if the absolute value is outside normal ranges.

Urine Sample

A mid-stream urine sample (approximately 30mL) will be obtained for Urinalysis at Screening and at 48 and 72 hours post-dose and at Day 5, 7, 14 and 28 (where possible in patients >5 years).

A semi-quantitative ‘dipstick’ evaluation for the following variables will be performed:

Specific gravity, pH, glucose, protein, bilirubin, ketones, leukocytes and blood. If the dipstick result is positive for protein (greater than trace) leucocytes and/or blood, the

sample will be sent for microscopic analysis of WBC and RBC. At the Investigator´s discretion, samples can be analysed further for the presence of bacteria.

7.13.1 Pregnancy Test

Pregnancy tests will be applicable to all post-pubescent females. Tests will be performed at Screening and at Day 28.

When performed at Screening/pre-dose, the result of this test must be confirmed negative before the patient may be dosed.

The patient´s menstrual and contraceptive history will be taken, and a test for the presence of HCG will be performed at Screening, to exclude pregnancy. Where feasible a quantitative serum HCG will be performed, failing which a qualitative serum HCG will be performed, failing which a qualitative urine HCG will be performed; a urine HCG is the minimum acceptable test. If pregnancy is detected, a quantitative test will be performed to determine the gestational age and the patient followed up until delivery according to the local practice.

7.13.2 FSH

The sample of blood taken for clinical chemistry analysis at Screening from females who are potentially of 'non-child bearing potential' may be tested for FSH.

7.14 Pharmacokinetics

Blood samples (0.5 mL) will be collected from patients for measurement of OZ439 and piperaquine (PQ) plasma concentration according to the Schedule of Assessments (the sampling times for patients <35kg is given under footnote k). Blood samples will be collected within ± 10 minutes of time-point for first 24 hours then ± 30 minutes until 72 hours. The Day 7 sample should be taken with 12 hours of the nominal time. All other samples should be taken within the time window allowance specified for each visit.

Blood samples will also be collected at time of failure (if applicable) to determine concentration of OZ439 and piperaquine.

Blood samples will be collected in appropriately sized K2-EDTA tubes from a vein using an indwelling cannula with switch valve or by direct venipuncture.

The samples will be stored in an ice bath (4 °C) until centrifugation where possible. At a minimum, samples must be stored and centrifuged below 23°C and processed in less than 60 minutes. After centrifugation (10 min, 4 °C, approximately 2000 x gravity), the supernatant plasma will be immediately pipetted off, and split between two polypropylene tubes (tubes should be filled with a minimum 0.1 mL plasma), pre-labelled and deep frozen at a temperature below –20 °C as soon as possible. Time span between blood sampling and freezing of the samples should not exceed 60 minutes. Plasma will be stored in frozen state at or below –20 °C until time of analysis. Pharmacokinetic plasma concentrations will be determined at the time points specified in the study schedule Section 2. The Study Procedures Manual will contain full details of sample processing.

7.15 Blood sample volumes

Table 4 Blood Sample Volumes

Assessment		volume (mL)	No. of samples	Total volume (mL)	No. of samples	Total volume (mL)	No. of samples	Total volume (mL)	No. of samples	Total volume (mL)
**Patient weight**			**≥35kg**		**≥20<35kg**		**≥10<20kg**		**≥5<10kg***	
**PK drug levels**		0.5	15/16	7.5/8	10	5	6	3	3	1.5
**Safety**	**Clinical chemistry**	1	7	7	5-7	5-7	5	5	5	5
	**Haematology**	1	7	7	5-7	5-7	5	5	5	5
**Total**				**21.5/22**		**15-19**		**13**		**11.5**

Other assessments (see Section 2) require negligible volumes of blood obtained via pin-prick. If all required tests cannot be performed using these minimal volumes due to machine restrictions in local areas, local protocol amendment or clarifications will be written.

7.16 Adverse Events (AE)

An AE is any untoward medical occurrence in a patient or clinical study subject administered a medicinal product and which does not necessarily have a causal relationship with this treatment. An AE can therefore be any unfavourable and unintended sign (including an abnormal laboratory finding), symptom, or disease temporally associated with the use of an IMP, whether or not considered related to the IMP.

All AEs that occur (whether treatment-related or not) will be recorded at each visit, for the duration of the study.

7.16.1 Adverse Event Description

As far as possible, each AE must be described by:

- A single diagnosis or symptom;
- Duration i.e., start date and time, and stop date and time (if start and/or stop date is uncertain, the information may be provided as text, e.g., ongoing or intermittent);
- Severity (Section 7.16.2);
- An assessment of causality (Section 7.16.3);
- Whether specific action or therapy was required;
- The outcome of the AE (recovered/resolved, recovered with sequelae, ongoing, recovering, death) and whether specific treatment or therapy, e.g., details of any medication given or surgical procedure performed, was required.

For the purposes of the study, AEs will be followed up according to local practice until the event has stabilised or resolved, or the Day 42 (or 63 where relevant) Visit, whichever is the sooner. SAEs will be recorded throughout the study.

7.16.2 Severity of Adverse Events

The Investigator will assess the severity/intensity of the adverse reactions and clinical laboratory changes using the DMID Toxicity Grading Scale for Determining the Severity of Adverse Events, see Attachment 1.

For abnormalities NOT found elsewhere in the Toxicity Tables use the scale below to estimate grade of severity:

**GRADE 1: Mild**	Transient or mild discomfort (< 48 hours); no medical intervention/therapy required
**GRADE 2: Moderate**	Mild to moderate limitation in activity - some assistance may be needed; no or minimal medical intervention/therapy required
**GRADE 3: Severe**	Marked limitation in activity, some assistance usually required; medical intervention/therapy required, hospitalisations possible
**GRADE 4: Life-threatening**	Extreme limitation in activity, significant assistance required; significant medical intervention/therapy required, hospitalisation or hospice care probable

Serious or life-threatening AEs: ANY clinical event deemed by the clinician to be serious or life-threatening should be considered a grade 4 event. Clinical events considered to be serious or life-threatening include, but are not limited to: seizures, coma, tetany, diabetic ketoacidosis, disseminated intravascular coagulation, diffuse petechiae, paralysis, acute psychosis, severe depression.

Clarification of the difference in meaning between ‘severe’ and 'serious’
The term ‘severe’ is often used to describe the intensity (severity) of a specific event (as in mild, moderate, or severe myocardial infarction); the event itself, however, may be of relatively minor medical significance (such as severe headache). This is not the same as ‘serious’, which is based on the outcome or criteria defined under the serious adverse event definition. An event can be considered serious without being severe if it conforms to the seriousness criteria, similarly severe events that do not conform to the criteria are not necessarily serious. Seriousness (not severity) serves as a guide for defining regulatory reporting obligations.

7.16.3 Causality of Adverse Events

All available evidence for the cause of the AE should be considered, such as the pharmacology of the IMP, the nature of the event, timing with respect to IMP administration and other causes. Possible other causes could include:

- The subject’s medical history
- Lack of efficacy or worsening of the treated condition
- Other treatment, concomitant or previous

- Treatment error
- Protocol-related procedure

The causal relationship between the IMP and the AE should be indicated:
Related or not related/levels of association, for example but not limited to:

Not related
The adverse event:
• does not follow a reasonable temporal sequence from drug administration
• is readily explained by the subject's clinical state or by other modes of therapy administered to the subject

Possibly
The adverse event:
• follows a reasonable temporal sequence from drug administration
• could have been produced by the subject's clinical state or by other factors

Probably
The adverse event:
• follows a reasonable temporal sequence from drug administration
• abates upon discontinuation of the drug
• cannot be reasonably explained by the known characteristics of the subject's clinical state
• is consistent with the known pharmacological or AE profile of the drug

Definitely
The adverse event:
• Event or laboratory test abnormality, with plausible time relationship to drug intake
• Cannot be explained by disease or other drugs
• Response to withdrawal plausible (pharmacologically, pathologically)
• Event definitive pharmacologically or phenomenologically (i.e. anobjective and specific medical disorder or a recognised pharmacological phenomenon)
• Rechallenge satisfactory, if necessary

7.16.4 Adverse Events of Special Interest (AESI)

Certain adverse events should be considered Adverse Events of Special Interest (AESI) and should be submitted to Quintiles Lifecycle Safety with 24 hours. If these are also SAEs, the SAE form should be used for notification (see section 7.16.5):

1. Hepatic
   - Possible Hy's law case: defined as a subject with any value of ALT or AST >3x ULN together with an increase in total bilirubin >2x ULN (>35% direct) and not associated with an ALP value >2x ULN
   - Any ALT or AST ≥5x ULN
   - Any elevation in total bilirubin ≥2.5x ULN (>35% direct)
   - Any AST or ALT ≥3x ULN with the appearance of fatigue, nausea, vomiting, right upper quadrant pain or tenderness, fever, rash and/or eosinophilia (eosinophil percent or count above the ULN)
   - ALT ≥3x ULN persisted for ≥4 weeks
2. Cardiac

- QTcF prolongation from baseline of >60 ms
- QTcF at any time >450 ms
- T wave liability, or T wave morphologic changes during therapy
- Bundle branch block
- Any arrhythmia

3. Haematological
    - Hb drop > 2 g/dL from baseline
    - Hb ≤ 5g/dL
    - Absolute neutrophil count <1000 /µL
4. Pregnancy
    - Pregnancy in treated patient or in partner of a treated male

7.16.5 Serious Adverse Events

An SAE is defined as any untoward medical occurrence that, at any dose:

- Results in death
- Is life-threatening*
- Requires hospitalisation or prolongation of existing inpatient's hospitalisation
- Results in persistent or significant disability or incapacity
- Is a congenital abnormality or birth defect
- Medical judgement should be exercised in deciding whether an adverse event/reaction is serious in other situations. Important adverse events/reactions that are not immediately life-threatening, or do not result in death or hospitalisation but may jeopardise a subject, or may require intervention to prevent one of the other outcomes listed in the definition above should also be considered serious
- Is a suspected case of drug induced liver toxicity (Hy's Law)

* 'Life-threatening' in the definition of 'serious' refers to an event in which the subject was at risk of death at the time of the event; it does not refer to an event which hypothetically might have caused death if it were more severe.

Reporting Serious Adverse Events
The Investigator is required to notify Quintiles LCS within 24 hours of becoming aware of the occurrence of an SAE or serious adverse reaction. All serious adverse events occurring during the study must be recorded on the SAE Form.

8 INTERIM ASSESSMENTS

8.1 Safety Evaluations for Independent Safety Monitoring Board (ISMB)

An Independent Safety Monitoring Board will be established to review emerging safety data, prior to the decision to step-down in age as detailed in Section 6.5.2 and Figure 1
The committee will be made up of at least one physician with expertise in paediatrics, one physician with expertise in cardiac conductance and one statistician. The Committee membership and the Charter of the Committee will be finalised prior to recruitment of the first patient.

8.2 Interim Extraction of PK/PD Data

At approximately the same time as the first interim assessment for efficacy / futility, a preliminary PKPD analysis of the study data will be performed to assist clinical development. This additional modelling verification will not influence the conduct of the study.

8.3 Efficacy/Futility Interim Analyses

Analyses will be performed for efficacy/futility on a regular basis in patients* randomised to the ' lower immunity population*' per treatment arm (lower immunity population i.e. Asian/ Latin American patients and African children ≤ 5 years). Interim assessment of efficacy and futility will occur after recruitment of approximately 50 evaluable patients per dose cohort and thereafter dependant on recruitment rate relative to the time required to perform the interim analyses.

At the early stages of the study, recruitment will continue during these analyses, however during later analyses, recruitment may be paused dependent on recruitment rate at the time. Recruitment of sub-populations will be stopped when the pre-determined numbers are reached or when a dose is determined to be futile. Details of the Interim Analyses are given in Section 11.

** Lower Immunity population includes all Asian / Latin American patients and African patients less than 5 years of age. During Interim Analyses for efficacy, a minimum of approximately 50% of patients per treatment arm must come from the population of African patients younger than 5 years of age.*

9 PATIENT FOLLOW UP IN THE EVENT OF HEPATIC ENZYME ABNORMALITIES

9.1 Circumstance requiring close monitoring of Hepatic Safety

1. Hy’s law (ALT or AST ≥3x upper limit of normal (ULN) and bilirubin >2x ULN and (>35% direct bilirubin) in the absence of a serum alkaline phosphatase level >2x ULN. [If fractionation is unavailable, record presence of detectable urinary bilirubin on dipstick indicating direct bilirubin elevations and suggesting liver injury.]
2. ALT ≥5x ULN.
3. ALT ≥3xULN if associated with the appearance or worsening of symptoms of hepatitis or symptoms such as fatigue, nausea, vomiting, right upper quadrant pain or tenderness, fever, rash or eosinophilia.
4. ALT ≥3x ULN persisted for ≥4 weeks.

When any of the conditions are met, the following must take place:

- Event must be considered an AESI (also possibly an SAE) and reported according to Section 7.16.4 and 7.16.5

- Follow up assessments must be performed (see below), and the patient monitored until the abnormality has resolved, stabilised, or returned to baseline values. Follow up is dependent on the original circumstances.

For patients meeting Hy's Law criteria:
Every reasonable attempt should be made to have patients return to clinic within 24 hours for repeat liver enzyme measurements and liver event follow up assessment. Patients should be monitored twice weekly until liver enzymes increases (ALT, AST, alkaline phosphatase, bilirubin) resolve, stabilise or return to within baseline values. A specialist or hepatology consultation is recommended where possible. For the check list of actions and assessment to be done in case of Hy’s law, refer to Attachment 2.

For patients meeting criteria requiring close monitoring of Hepatic Safety but not meeting Hy's Law criteria:
Every reasonable attempt should be made to have patients return to clinic within 24 to 72 hours for repeat liver enzyme measurements and liver event follow-up assessments. Patients should be monitored weekly until liver enzyme increases (ALT, AST, alkaline phosphatase, bilirubin) resolve, stabilise or return to within baseline values.

Every reasonable attempt should be made to carry out liver event follow-up assessments as described below:

Viral hepatitis panel including:
- Hepatitis A IgM antibody, Hepatitis B surface antigen and Hepatitis B Core Antibody (IgM), Hepatitis C RNA, Hepatitis E IgM antibody
- Cytomegalovirus IgM antibody
- Epstein-Barr viral capsid antigen IgM antibody (or if unavailable, heterophile antibody or monospot testing obtained)
- Serum creatine CPK and lactate dehydrogenase (LDH)
- Use of concomitant medications, acetaminophen, traditional and herbal remedies, other over the counter medications, or putative hepatotoxins, must be recorded on the concomitant medications report form.
- Alcohol use to be recorded

9.2 Premature Discontinuation of Individual Patients

Patients will be discontinued from the study for any of the following reasons:

- Withdrawal of consent
- If, in the Investigators’ opinion, it is not in the best medical interest of the patient to continue the study
- At the discretion of the Investigator or if the patient is not sufficiently co-operative
- Occurrence of an AE, which, in the opinion of the Investigator, warrants discontinuation of the patient from the study;
- If a patient is lost to follow-up
- Administrative reasons

Patients will be informed that they are free to withdraw from the study at any time and for any reason and without the need to provide a reason. The date the patient discontinued from the study and the reason for discontinuation will be recorded in the eCRF.

If the patient discontinues following dosing, the patient should be encouraged to attend the Clinical Unit for safety assessments up to Day 7 where possible.

10 TREATMENT

10.1 Investigational Medicinal Product Details

The following Investigational Medicinal Products (IMPs) are manufactured and tested by Penn Pharmaceutical Services Limited (Tredegar, Gwent, NP22 3AA, UK) in compliance with Good Manufacturing Practice (Annex 13) and all relevant regulations.

Individual sachets containing OZ439 + TPGS granules for oral suspension; dose strengths 800 mg, 600 mg, 400 mg, 300 mg and 200 mg

Individual sachets containing piperaquine phosphate granules for oral solution; dose strengths 720 mg, 540mg, 480mg, 360mg, 320mg, 240mg and 160mg

Individual sachets containing sucrose granules; dose strengths 19.43 g, 15.00g, 14.80g, 11.25g, 9.71g

Piperaquine phosphate tablets in individual blister packaging; dose strengths 320 mg and 80 mg

Placebo to match piperaquine phosphate tablets in individual blister packaging

The IMPs will be provided to a second party who will package the IMPs into 'individual subject treatment packs' and release the 'individual subject treatment packs' for clinical use. The packaging, labelling and release of the 'individual subject treatment packs' will be performed in compliance with Good Manufacturing Practice (Annex 13) and all relevant regulations.

10.2 Labelling, Storage and Dispensing

'Individual subject treatment packs' will be provided for this study. Each 'individual subject treatment pack' will comprise a carton, with tamper proof seals, which will contain all the IMPs which will be administered to an individual patient for the single dose treatment of OZ439 and piperaquine phosphate.

For patients with body weight $\geq$ 24 kg the 'individual subject treatment pack' will contain:

- One sachet of OZ439 + TPGS granules for oral suspension.
- One sachet of sucrose granules.
- Piperaquine phosphate tablets in individual blisters and/ or placebo to match piperaquine phosphate tablets in individual blisters. The number and dose strengths of active tablets, and the number of placebo tablets required to maintain the treatment blind, in the 'individual subject treatment pack' are determined by the treatment dose.

For patients with body weight ≤ 23.9 kg the individual patient treatment pack will contain:

- One sachet of OZ439 + TPGS granules for oral suspension.
- One sachet of sucrose granules.
- One sachet of piperaquine phosphate granules for oral solution.

Labelling of the 'individual subject treatment packs' and the IMPs within the 'individual subject treatment pack' will be performed in accordance with all applicable country specific regulatory requirements.

The 'individual subject treatment packs' must be stored at 15 to 30°C in a secure area. The Investigator or designee must monitor the temperature of the storage area where the 'individual subject treatment packs' are kept. Storage of the 'individual subject treatment packs' and dispensing of the IMPs will be the responsibility of the Investigator or designee.

Patients with body weight ≥24kg with receive OZ439 as an oral suspension prepared from the OZ439 + TPGS granules for oral suspension, sucrose granules and water. They will receive piperaquine phosphate in the form of tablets.

Patients with body weight ≤ 23.9kg with receive OZ439 and piperaquine phosphate as an oral suspension prepared from the OZ439 + TPGS granules for oral suspension, piperaquine phosphate granules for oral solution, sucrose granules and water.

Details for preparation of the oral suspensions will be provided in the Study Procedures Manual.

10.3 Dosage, Duration and Compliance

All patients will receive a single dose of study IMP on Day 0, once the inclusion and exclusion criteria are verified. The individual subject treatment pack, as indicated by the IWRS system, will be selected and the batch number, treatment number and date of expiry will be recorded. Individual subject treatment packs will contain treatments A, B or C as detailed below, however only the dosage of the OZ439 will be specified.

Table 5: Treatments and Dosage

**Weight Band**	**Treatment A**	**Treatment B**	**Treatment C**	**Volume of suspension**
≥35kg	800mg OZ439 administered as an oral suspension also containing 15g sucrose, 1440mg PQP administered as tablets	800mg OZ439 administered as an oral suspension also containing 15g sucrose, 960mg PQP administered as tablets	800mg OZ439 administered as an oral suspension also containing 15g sucrose, 640mg PQP administered as tablets	240mL
24-34.9kg	600mg OZ439 administered as an oral suspension also containing 11.25g	600mg OZ439 administered as an oral suspension also containing 11.25g	600mg OZ439 administered as an oral suspension also containing 11.25g	180mL

	sucrose, 1120mg PQP administered as tablets	sucrose, 720mg PQP administered as tablets	sucrose, 480mg PQP administered as tablets	
15-23.9kg	400mg OZ439 and 720mg PQP administered as an oral suspension also containing 19.43g sucrose	400mg OZ439 and 480mg PQP administered as an oral suspension also containing 19.43g sucrose	400mg OZ439 and 320mg PQP administered as an oral suspension also containing 19.43g sucrose	210mL
10-14.9kg	300mg OZ439 and 540mg PQP administered as an oral suspension also containing 14.80g sucrose	300mg OZ439 and 360mg PQP administered as an oral suspension also containing 14.80g sucrose	300mg OZ439 and 240mg PQP administered as an oral suspension also containing 14.80g sucrose	160mL
5-9.9kg	200mg OZ439 and 360mg PQP administered as an oral suspension also containing 9.71g sucrose	200mg OZ439 and 240mg PQP administered as an oral suspension also containing 9.71g sucrose	200mg OZ439 and 160mg PQP administered as an oral suspension also containing 9.71g sucrose	105mL

Prior to initiating treatment the required equipment should be assembled: Label for the plastic cup which will hold the OZ439 oral suspension; Pot and label which will hold the tablets (patients >24kg); appropriately sized plastic cup; plastic spoon; tin foil; measuring cylinders or syringes for measuring the sterile water; sterile water; scissors; tweezers.

Plastic cups will be labelled for the OZ439 oral suspension and the time at which preparation begins noted, as the suspension must be consumed within 2 hours and 20 minutes of the start of preparation.

For patients over 24kg: The foil on the tablet blisters should be carefully opened using clean scissors or tweezers and taking care not to damage the tablets. The tablets will be placed in labelled pots.

The required amount of sterile water will be measured into the plastic cup. The sachet containing the OZ439 + TPGS granules has two compartments. These will be cut open with clean scissors and the full contents emptied into the cup. The inside of both compartments of the sachet will be rinsed with a small volume of sterile water and this will be added to the plastic cup. The mixture will be stirred with a plastic spoon for 1-2 minutes so that the granules are wetted. The spoon will be left in the cup and the top

loosely covered with tin foil and left to stand for 20 - 30 minutes. The sucrose will then be added, stirred and the plastic spoon removed.

The oral suspension should then be given to the patient to drink if there are at least 30 minutes remaining before the suspension expires. The patient must drink all the oral suspension as quickly as possible and at least within a 30 minute period.

The cup will be rinsed with 100mL of drinking water which the patients ≥24kg will use to help swallow the PQP tablets. All the tablets must be taken and additional water may be used if necessary.

For patients <24kg, the OZ439 and PQP will be administered in a combined oral suspension. Plastic cups will be labelled for this and the time at which preparation begins noted, as the suspension must be consumed within 2 hours and 20 minutes of the start of preparation. The required amount of sterile water will be measured into the plastic cup.

Using clean scissors, the top of the sachet containing the piperaquine phosphate granules should be cut to open the sachet and the contents added to the cup, and the sachet rinsed.

Both compartments of the sachet containing OZ439 + TPGS granules should be cut open and added to the cup, and then the sachet rinsed and the rinse water added.

The mixture should be stirred with a plastic spoon for 1-2 minutes so that the granules are wetted, the top loosely covered with tin foil (leaving the spoon in place) and the mixture left to stand for 20 - 30 minutes. The sucrose from the sachet should be added and the mixture stirred for a further 1-2 minutes and then the plastic spoon removed.

The OZ439 and piperaquine phosphate combined oral suspension will then be administered to the patient, ensuring that at least 30 minutes is available before the mixture expires.

The patient must drink all the oral suspension as possible but if necessary it can be drunk over a 30 minute period. If there is any residue remaining in the cup, it should be rinsed with drinking water and administered to the patient.

Dosing of the suspension (and tablets) should be directly observed to ensure the full dose is taken. A dosing syringe may be used to dose the suspension if required.

The IMP should be dosed in a sitting position where possible to minimise the risk of vomiting.

Patients should have fasted from food and milk for 3 hours before administration.

10.4 Accountability

On receiving a shipment of the IMP at the site, the Investigator or designee will conduct an inventory check and confirm via IWRS that the shipment has been received.

During the study, the Investigator or designee will record the 'individual subject treatments pack' numbers dispensed to the patients on the dose preparation forms and in accordance with the IWRS. Drug accountability will be checked by the Investigator or designee who is responsible for maintaining an accurate inventory and accountability record for the IMP received and dispensed by the site. This will be confirmed by the CRA.
At the end of the study the sponsor or designee will arrange for all unused 'individual subject treatments packs' to be returned to the sponsor or designee who will also arrange for destruction. Written proof of destruction will be kept in the sponsors or designee files

10.5 Code-breaking

This is a randomised, double-blind study with limited access to the randomisation code. Study medication and placebo tablets will be identical in physical appearance. The treatment each patient will receive will not be disclosed to the Investigator, study centre staff, subject, Sponsor, Quintiles or the ISMB.
The process for breaking the blind will be handled through the IWRS. Investigators are strongly discouraged from requesting the blind be broken for an individual patient, unless there is a patient safety issue that requires unblinding and would change patient management. Any centre that breaks the blind under inappropriate circumstances may be asked to discontinue its participation in the study. If the blind is broken, it may be broken for only the patient in question.
The Sponsor and Quintiles must be notified immediately if a patient and/or Investigator is unblinded during the course of the study. Pertinent information regarding the circumstances of unblinding of a patient's treatment code must be documented in the source documents and electronic case report forms (eCRFs).

10.6 Treatment of Overdose

No cases of overdose have been reported. In cases of suspected overdose, symptomatic and supportive therapy should be given as appropriate, including ECG monitoring because of the possibility of QTc interval prolongation.

11 STATISTICAL METHODS AND DATA MANAGEMENT

11.1 Statistical Analysis Plan

Listing and table shells will be created before the first safety evaluation and details concerning the evaluations will be documented in a separate ISMB Charter.

A detailed statistical analysis plan (SAP), describing the purpose, timing, presentation of results and access to results will be provided by Quintiles before Database lock.

If a change in the planned analyses is considered necessary after finalisation of the protocol (except if protocol requires modifications), such changes will be described and justified in the SAP.

This study, being part of a multicenter clinical study, is not powered to compare treatment arms.

11.2 Analysis Sets

All Patients Enrolled Analysis Set

All patients who provide written informed consent prior to performing any specific study-related procedures.

All Patients Randomised Analysis Set

All patients enrolled who are randomised to one of the three OZ439/PQP randomised treatment arms.

For the purpose of statistical analyses and presentations based on the randomised analysis set, patients will be classified according to randomised treatment arm, regardless of actual treatment received.

Safety Analysis Set

All patients randomised who received the single dose combination of OZ439/PQP study treatment.

All safety analyses will be based on the Safety analysis set according to actual treatment received.

Intent to Treat (ITT) Analysis Set

All patients included in the Safety analysis set for whom baseline and at least one post-baseline efficacy assessment (clinical and parasitological data) are available. For the purpose of analyses based on this analysis set, patients will be classified according to planned treatment arm, regardless of actual study treatment received.

Per Protocol (PP) Analysis Set

All patients completing the study up to Day 28 without major protocol deviations.

Patients experiencing unsatisfactory treatment effect will be included in the PP analysis set (PPS), if otherwise valid. Major protocol deviations are defined as any factor that may have an effect on the efficacy outcome or the treatment of a patient.

Patients are characterised by the following valid course criteria (ICH-E9):

- Sufficient evidence of the study indication, i.e. acute, uncomplicated *P. falciparum* malaria.
- Overall treatment compliance.
- Adherence to the visit schedule (specifics will be detailed in the SAP).
- Eligible in accordance with the clinical study protocol’s specified inclusion and exclusion criteria. Specifically criteria which could affect the efficacy outcome or the treatment effect:

- Use of prohibited concomitant medications.
- Presence of prohibited medical conditions.
- Signs/symptoms of severe/complicated malaria.
- Mixed *Plasmodium* infection.
- Severe vomiting or severe malnutrition.

PK Analysis Set

There will be two PK analysis sets defined by the amount of data and ability to perform the analyses.

A richer dataset is required for non-compartmental analysis and this dataset will be comprised of patients older than 5 years. These patients will be included providing they are included in the Safety analysis set, have at least three (to possibly include Tmax) measured PK plasma concentrations after the start of study treatment and had no major protocol deviations defined as protocol deviations affecting the integrity of the PK data.

The second dataset will be compiled for the population PK analysis and will include all patients with the same criteria expressed above except that the patients will be included in this dataset if they have at least one measured PK plasma concentrations after the start of study treatment.

For the purpose of analyses based on this analysis set, patients will be classified according to actual treatment received, regardless of randomised treatment arm.

11.3 Sample Size

This protocol is adaptive allowing reassessment of predicted efficacy (probability of achieving a PCR-adjusted ACPR28 > 95%) at pre-specified interim points during the conduct of the study (see Section 11.8.2). At each interim time point, tests for futility probability of achieving ACPR28 <90% to large) or efficacy (overwhelming probability of achieving ACPR28 > 95%) will be performed. Recruitment will cease to a particular treatment arm if the test for futility is positive. See Section 8.3 for numbers required for each interim analysis.

Given the adaptive nature of the study design, the total number of patients to be recruited to this study can only be estimated. Prior studies indicate that an ACPR28 for an active combination can be detected with 106 patients per treatment arm (Van Vugt *et al.* 1999).

Simulations evaluating a range of predicted efficacy values for the combinations suggest that a maximum number of patients required to categorise each treatment arm is 208. However, study numbers will be capped at 150 per treatment arm (primary lower immunity population of interest - to characterise the exposure-response relationship) plus an approximate additional 10% African patients older than 5 years (likely to have some immunity and therefore not included in the efficacy/futility estimates), giving an estimated total maximum number of patients of 165 per treatment arm.

Recruitment will therefore continue until each treatment arm is deemed to be futile or until a maximum of 150 patients per treatment arm is reached. Thus the adaptive criteria will be applied up to a maximum number of 150 patients at which point the ACPR28 percentage will be calculated regardless, in a small number of predicted outcomes, the treatment arms not being clearly futile or successful.

Trial simulations suggested that a treatment arm size of 120* should be required if the dose combination is effective, a finding consistent with earlier trials (Van Vugt *et al.* 1999).

The target recruitment will therefore range from 120 per treatment arm (for the efficacy/futility analysis) plus an additional 12 African patients > 5 years (i.e. a total 132 per arm) to a maximum number per treatment arm based on 150 per treatment arm (for the efficacy/futility analysis) plus an additional 15 African patients >5 years that is 165 patients.

Note, lower numbers of patients per treatment arm may be recruited in the event of a poorly efficacious dose or doses (i.e. futility).

Thus the targeted maximum number of patients recruited to this study will be 495 that is 3×165.

11.4 Data Analysis

All relevant data obtained in this study and documented in the eCRF will be listed individually and, tabulated. All statistical analyses will be performed using SAS® (version 9.4 or higher).

Data will be summarised as follows: Continuous variables by descriptive statistics (number of patients [n], mean, standard deviation [SD], minimum, median and maximum, and categorical variables by absolute and relative frequencies (n and %) or contingency tables.

Descriptive statistics will also be provided for subgroups such as population (lower immunity, total), region, age group (adult, child) and gender.

Unless indicated otherwise, summary statistics will be reported for observed data only, by treatment arm, and missing data will not be imputed. If a baseline value is missing, no change from baseline will be calculated.

Unless otherwise specified, baseline is defined as the last available assessment (scheduled/unscheduled) prior to the administration of the single dose combination of OZ349/PQP study treatment. The only exceptions are temperature, electrolytes (sodium, potassium) and glucose where the first Screening assessment is to be regarded as baseline.

<u>Common Calculations</u>

For quantitative measurements, change from baseline will be calculated as: (Test value at Visit X – baseline value).

For numerical values > 0 where the logarithmic transformation is required, the logarithm value will be calculated as: $\log_{10}$(Value).

11.5 Missing, Unused and Spurious Data

Missing safety, efficacy and PK data will not be imputed for this study. Missing data will be identified at time of data transfer. All analyses will be performed based on data received; unless otherwise specified no imputation of missing values will be performed prior to statistical analysis.

Data listings will include scheduled, unscheduled, retest and prematurely discontinuation data.

Deviations from the Statistical Plan

Any deviation(s) from the final version of the SAP following database lock and routine study unblinding will be described and justified in the clinical study report.

11.6 Statistical Criteria for Treatment Arm and Study Termination

Interim analyses will be performed for efficacy/futility by an unblinded statistician (independent from the project team and blinded statisticians) initially when 50 patients per treatment arm have reached Day 28 or prematurely discontinued and thereafter every time an additional 25 patients per treatment arm have reached Day 28 or prematurely discontinued (Low immunity population i.e. Asian/Latin American patients and African children < 5 years); see Section 11.8.2 below.

At the early stages of the study, recruitment will continue during these analyses, however during later analyses, recruitment may be paused dependent on recruitment rate at the time. Recruitment of sub-populations will be stopped when the pre-determined numbers are reached. Recruitment will cease to a particular treatment arm if the test for futility is positive.

11.7 Baseline Characteristics

Patient Disposition

Patient disposition, discontinued/completed status, included in the relevant analysis sets, reason for exclusions from the relevant analysis sets and major protocol deviations data will be summarised for patients randomised to study treatment and presented in data listings for all patients enrolled.

Demographics and Protocol Compliance

Demographic data (age, gender, and race/ethnicity) and other baseline characteristics will be summarised for the Safety analysis set, and presented in data listings for all patients enrolled.

Medical History

Medical history will be summarised for the Safety analysis set, and presented in data listings for all patients enrolled.

Concomitant Medications

Concomitant medications will be summarised for the Safety analysis set, and presented in data listings for all patients enrolled.

Study Drug Exposure and Compliance

Exposure and compliance to study treatment will be summarised for the Safety analysis set and presented in data listings for all patients randomised to study treatment.

11.8 Efficacy Analysis

11.8.1 Primary efficacy analysis

The primary endpoint to determine the efficacy/futility of OZ439/PQP will be the PCR-adjusted adequate clinical and parasitological response (ACPR) at Day 28.

PCR-adjusted ACPR applies only to recrudescence (re-emergence of the original clone of parasite that is present at baseline). Recrudescence is distinguished from re-infection by genotyping the parasite clone.

For Interim Analysis:

Interim analysis of efficacy/futility will be performed ongoing during the study for up to 150 patients per treatment arm, at which stage recruitment would be capped and an ACPR28 calculated regardless.

The following null hypothesis will be tested

$$\boldsymbol{H_0}: \boldsymbol{p} \leq \mathbf{0.9} = \boldsymbol{p_0}$$

against the one-sided alternative

$$\boldsymbol{H_1}: \boldsymbol{p} \geq \mathbf{0.95} = \boldsymbol{p_1}$$

where $\boldsymbol{p}$ is the probability of ACPR at Day 28.

The study will follow a group sequential design with up to 5 interim analyses based on the posterior probability methodology as discussed by Lee and Liu (2008).

At each interim analysis, one of the following potential decisions is to be made:

- The treatment arm is stopped for futility if the posterior probability of $\boldsymbol{H_0}$ (that is, response rate is below 90%) given the data accumulated at the look for the treatment arm in question is too large, that is,

$$\mathbf{Pr}(\boldsymbol{H_0} | \boldsymbol{data}) \geq \mathbf{0.3}$$

- Efficacy will be concluded if the posterior probability of $\boldsymbol{H_1}$ (that is, response rate is above 95%) given the data accumulated at the look for the treatment arm in question is large, that is,

$$\mathbf{Pr}(\boldsymbol{H_1}|\, \boldsymbol{data}) \geq \mathbf{0.9}$$

- The treatment arm advances to the next stage if neither of the above conditions is met.

In the calculation of posterior probabilities, the Beta (9.5,0.5) distribution will be used as prior distribution for the response rate.

The procedure's operating characteristics were evaluated through simulations for a range of plausible scenarios for the true value of p (ranging from 0.85 to 0.98) and the maximum sample size was chosen to correspond to the most pessimistic of the aforementioned scenarios (yielding a maximum sample size of 208 per treatment arm, and corresponding average sample size of 104 per treatment arm).

These outcomes, taken with numbers used in prior malaria combination studies (106 per arm), indicated that recruitment of 120 to 150 patients per treatment arm would be prudent. If the combination is efficacious, 120 per arm should suffice. A total of 150 per treatment arm provides an upper limit of recruitment when results are consistently between the futility and success criteria. Analysis will be detailed further in the SAP.

For Final Analysis:

On a specified Day (D), the PCR-adjusted ACPR is the proportion of patients that have no evidence of recrudescence as determined microscopically and genotypically as an absence of the same asexual parasitaemia (clone) as the original infection.

The proportion of patients with PCR-adjusted ACPR at Day 28 will be summarised descriptively by treatment arm. Standard descriptive statistics as well as 95% confidence intervals will be provided. Exploratory subgroup analysis will be discussed further in the SAP.

11.8.2 Secondary Endpoints

Secondary efficacy variables will be summarised descriptively with incidence rates or standard descriptive methods, including 95% confidence intervals, as appropriate. Time to event variables will be summarised with Kaplan-Meier estimates of the survival function and Cox regression analysis.

11.8.2.1 Efficacy Analysis

<u>PCR - adjusted ACPR at Day 42 and 63*:</u>

The methods used for the analysis of the PCR-adjusted ACPR at Days 42 and 63 will be the same as those for the primary efficacy endpoint (at Day 28). The number of measurements will be smaller as patients who experience primary conversion but then have a reversion to failure at one or both of the repeat tests will not be counted.

PCR – crude ACPR at Day 28, 42 and 63*

PCR- crude ACPR does not distinguish between re-infection (by a new clone of parasite) and recrudescence (re-emergence of the original clone of parasite that is present at baseline).

On a specified Day (D), the PCR-crude ACPR is the proportion of patients that have no evidence of asexual parasitaemia as detected microscopically, independent of whether any parasitaemia is due to re-infection or recrudescence.

The methods used for the analysis of these endpoints will be equivalent to those used to determine the PCR-adjusted ACPR with the exception that PCR-crude data will be used.

Re-emergence, recrudescence and re-infection at Day 28, 42 and 63*

The risk of recrudescence and re-infection over 28, 42 and 63 days will be estimated by the Kaplan-Meier method. Time to recrudescence and re-infection will be censored at the time of the last recorded visit, if recrudescence and re-infection respectively have not been observed by that time.

Parasite clearance time (PCT)

Parasite clearance time is defined as the time from dosing to the first negative (no parasites) film. This negative film must be confirmed by a second negative film, taken within 6 to 12 hours of the first. Parasite clearance will be concluded following confirmation of the second negative film.

Kaplan-Meier estimates will be used to evaluate parasite clearance time of baseline parasitaemia.

As an exploratory analysis, the time to microscopic 50, 90 and 99% reduction of asexual parasites will be determined. For patient data that supports extrapolation, the parasitaemia will be estimated by log-linear extrapolation of the parasitaemia curve.

Fever clearance time (FCT)

FCT is defined (in patients with an increased temperature at baseline) as the time of the first measurement of axillary temperature of < 37.5 °C (or < 38.0 °C for alternative routes). This measurement must be confirmed by a second measurement, taken within 6 to 12 hours of the first. Fever clearance will be concluded following confirmation of temperature < 37.5 °C on the second measurement. Patients entered in the study on the basis of history of fever and who do not subsequently have an increased body temperature measurement indicating presence of fever pre-dose, will not be included in the analysis of fever clearance time

Kaplan-Meier estimates will be used to evaluate fever clearance time.

Parasite Reduction Rate (PRR)

The parasite reduction rate is calculated as the slope of the linear portion of the regression fit of natural log parasitaemia (per mL) versus time (in hours).

The calculations and modeling for the PRR will be further discussed in the SAP. This will be calculated using microscopic and qPCR determined parasitaemia.

Interim Extraction of PK/PD Data

At approximately the same time as the first interim assessment for efficacy / futility a preliminary PKPD analysis of the study data will be performed to assist clinical development. This additional modelling verification will not influence the conduct of the study.

11.8.2.2 Exploratory Analysis

Gametocyte carriage

- For the patients with gametocytes at baseline the time to gametocyte clearance will be estimated through the Kaplan-Meier method.

- Patients without gametocytes at baseline that develop gametocytes during the study.

  The risk that gametocytes are detected for patients who are gametocyte negative at baseline up to time of gametocyte appearance will be estimated through the Kaplan-Meier method.

  The log parasite counts over time will be summarised descriptively by treatment arm and visit.

Integrated number of gametocytes (AUC) at 28 and 42 days

Integrated number of gametocytes (AUC) at 28 and 42 days for:

- Patients with gametocytes at baseline.
- Patients without gametocytes at baseline that develop gametocytes during the study.

The Integrated number of gametocytes (AUC) at 28 and 42 days will be done as part of the data analysis but will not form part of the CSR and will be reported separately. Details of relevant methods will be described in a separate analysis plan.

Correlation between exposure (Day 7) and response (Day 28) of OZ439/PQP

All patients included in the Per Protocol analysis set and for whom PK concentration data are available at Day 7.

The relationship between PCR-adjusted ACPR28 and plasma OZ439 and plasma piperaquine concentrations on Day 7 will be assessed through logistic regression, with the binary variable ACPR28 as outcome variable and plasma OZ439 and plasma piperaquine concentrations as linear predictor variables (possibly after logarithmic transformation of the plasma concentration). If this generalised linear model proves to be inadequate, a generalised additive model (GAM) will be fitted to explore the exposure response relationship.

Additional analyses of the relationship between ACPR28 and plasma OZ439 and plasma piperaquine concentrations will form an exploratory analysis Section detailed in the SAP.

An exploratory evaluation of the relationship between Kelch-13 genetic status and parasite clearance half-life, and possibly other clinical endpoints, will be carried out as detailed in the SAP. Additional parasite genotypes of interest associated with drug resistance may be carried out if considered appropriate.

11.8.2.3 Pharmacokinetic Analysis

Non-compartmental analysis

The following PK parameters will be estimated from patients' individual plasma concentrations of OZ439 and piperaquine by applying a non-compartmental approach using WinNonlin Professional. Concentration values reported as below the limit of quantitation will be set to zero where appropriate. PK parameters will be estimated for patient's having sufficient data to calculate the parameters, namely patients above 5 years of age.

- Cmax: The maximum concentration observed in the concentration time course following administration of the drug. Unit = ng/mL
- Tmax: The time at which the Cmax is observed. Unit = hours
- AUC: The area under the concentration time curve from time = 0 hours until a defined endpoint. The AUC will be calculated using linear interpolation up to Cmax followed with log-linear interpolation until the last time point. Last denotes the last measured time point. Infinity denotes extrapolation of the AUC to infinity. Unit = hr.ng/mL.

$$AUC_{0-last} = \sum_{t=last}^{t=0} \left[\left(\frac{C_{i+1} + C_i}{2} \right) \times (t_{i+1} - t_i) \right]$$

Where i denotes a time point, t=0 $\leq$ i $\leq$ t=last

$$AUC_{0-inf} = AUC_{0-last} + \frac{C_{last}}{\lambda_z}$$

- λ_z : The log linear gradient determined over the log linear elimination period following Tmax. Unit = 1/hour.
- Elimination half-life ($t_{1/2}$): The transformed log linear elimination phase gradient. Unit = hour

$$t_{1/2} = \frac{\ln(2)}{\lambda_z}$$

- Clearance (CL/f): The clearance estimated following oral administration. The clearance explicitly includes bioavailablity (f) within its estimate. Unit = L/hour

$$\frac{CL}{f} = \frac{Dose\ (mg)}{AUC_{0-inf}}$$

Descriptive statistics including mean, standard deviation, coefficient of variation, median, minimum and maximum, geometric mean and geometric mean CV% will be calculated for each PK parameter by treatment arm. Descriptive statistics will also be provided for some subgroups which will be discussed further in the SAP.

Mean and median concentration-versus-time graphs will be presented.

Non-linear mixed effects modelling

Non-linear mixed effects modelling will be done as part of the data analysis but will not form part of the CSR and will be reported separately. Details of relevant methods will be described in a separate analysis plan.

11.9 Safety Analysis

Adverse events

All adverse events will be coded using the Medical Dictionary for Regulatory Activities (MedDRA) and will be presented by Preferred Term within each MedDRA System Organ Class (SOC).

Treatment-emergent adverse events (TEAEs) are defined as AEs which started at or after the administration of IMP (study treatment) and includes those events started prior to the first administration of IMP but which worsened after the dose intake, until the last scheduled assessment will be regarded as treatment-emergent, but before established anti-malarial treatment is administered, if required (see Section 5.7).

Treatment-related AEs are those rated by the Investigator as 'definite', 'probable' or 'possible'. In case relationship is unknown or missing the worst case will be assumed and the AE will be considered to be treatment-related.

All AEs will be listed, and will include the Investigator term, the preferred term, start and end date of AE, duration (days), severity, drug relationship, action taken and outcome.

An overview of the following TEAEs will be presented:

- TEAEs.
- TESAEs
- TESAEs leading to death.
- TESAEs of drug induced liver toxicity (Hy's law).
- TEAEs leading to premature study discontinuation.
- TEAEs leading to study drug discontinuation.
- TEAEs related to study drug.
- Severe or life-threatening TEAEs.

Summaries will consist of the number of patients with at least one TEAE in each category (patients with multiple TEAEs in each category are counted only once in each category) presented by treatment arm for the Safety analysis set. The percentage of patients with at least one TEAE in each category will be calculated relative to the total number of patients in the Safety analysis set.

Laboratory Data

Summary statistics of absolute values and change from baseline over time for quantitative measurements and frequencies and percentages for qualitative data will be presented for all safety laboratory variables.

Quantitative laboratory measurements will be compared with the relevant laboratory reference ranges and categorised as low, normal and high, and presented in data listings.

The shifts of quantitative (“low”, “normal” and “high”) safety laboratory assessments from baseline to each relevant post-baseline scheduled visit will be presented.

The proportion of patients with clinically significant abnormal values, patients with DMID toxicity scale of Grade 3 or 4 and patients meeting the Hy’s law definition will be provided by laboratory variable and treatment arm.

Possible Hy's law case is defined as a patient with any value of ALT or AST above 3x upper limit of normal (ULN) together with an increase in bilirubin to a value higher than 2xULN (>35% direct) and NOT associated to an ALP value higher than 2xULN (FDA, 2009).

Other laboratory results (apart from haematology, clinical chemistry and urinalysis laboratory tests) will also be listed.

ECG

QT intervals will be adjusted using Fridericia’s, Bazett’s and Wernicke’s correction formulas. ECG results (heart rate, RR interval, PR interval, QRS interval, QT interval and QTc interval), including observed and change from baseline, will be presented by treatment arm and scheduled visits.

Frequency counts will be used to summarise the ECG results classified as normal, abnormal clinical significant and abnormal not clinical significant (Investigator’s assessment) by treatment arm and scheduled visit.

Post-baseline QT/QTc intervals will be classified into the following categories:

- QT/QTc < 450 ms
- 450 ms ≤ QT/QTc < 480 ms
- 480 ms ≤ QT/QTc < 500 ms
- QT/QTc ≥ 500 ms

QTc change from baseline will be classified into the following categories:

- < 30 ms increase from baseline,
- ≥ 30 ms and < 60 ms increase from baseline, and
- ≥ 60 ms increase from baseline.

Frequency counts will be used to summarise the number of patients according to the above categories.

Vital Signs

Supine blood pressure and heart rate (absolute values and change from baseline) will be summarised by treatment arm and scheduled visit.

Specifications of all data analysis including any further safety analysis will be detailed in the SAP.

11.10 Data Management

All data will be documented by means of an Internet-based electronic CRF (InForm), with automatic plausibility and completeness checks performed online. Access to study data is only possible using a personal username and password combination, which the user must keep strictly confidential. Electronic communication between the InForm server and the user's personal computer is encrypted without transmission of protected data (patient names, address, social security number etc.). A complete audit trail of all changes of study data will be available. Laboratory data will be verified using center and gender-dependent normal ranges.

A third step of data cleaning will take place centrally at the InForm website by the data management personnel. Descriptive and logical data checks are performed on the evolving database according to a pre-specified data validation plan. Data queries will be issued electronically within the documentation system, answered by the Investigator and handled by appropriately trained staff at Quintiles Data Management and Biostatistics.

Verbatim entries (adverse events, prior or concomitant diseases and medications) will be coded by means of the following coding systems:
Adverse events: MedDRA (version 16.1 or higher)
Concomitant medication: WHO Drug Reference List (Whodrug 01Dec2013 or higher)
Concomitant diseases and diagnoses: MedDRA (version 16.1 or higher).

12 REGULATORY, ETHICAL AND LEGAL OBLIGATIONS

12.1 Declaration of Helsinki

The Investigator will ensure that this study is conducted in full conformity with the current revision of the Declaration of Helsinki.

12.2 Good Clinical Practice

The study will be conducted according to the protocol and to Standard Operating Procedures (SOPs) that meet the current regulatory requirements and guidelines laid down by the International Conference on Harmonisation for Good Clinical Practice (ICH GCP) in clinical studies.

12.3 Independent Ethics Committee/Institutional Review Board Approval

12.3.1 Initial Approval

Prior to the shipment of IMP and the enrolment of patients, the IEC/IRB must provide written approval of the conduct of the study at named sites, the protocol and any amendments, the Patient Information Sheet and Consent Form, any other written information that will be provided to the patients, any advertisements that will be used and any patient compensation.

12.3.2 Approval of Amendments

Proposed amendments to the protocol and aforementioned documents must be submitted to the Sponsor for review and submitted to the IEC/IRB for approval as instructed by the

Sponsor. Amendments requiring IEC/IRB approval may be implemented only after a copy of the IEC/IRB's approval letter has been transmitted to the Sponsor.

Amendments that are intended to eliminate an apparent immediate hazard to patients may be implemented prior to receiving Sponsor or IEC/IRB approval. However, in this case, approval must be obtained as soon as possible after implementation.

12.3.3 Reporting of Suspected Unexpected Serious Adverse Reactions

Suspected unexpected serious adverse reactions (SUSARs; also known as serious adverse drug reactions) occurring during the study at any investigational site will also be reported to the IEC/IRB within the required timelines. The actual reporting of the events will be performed as instructed by the Sponsor.
If any IEC/IRBs have the requirement to receive only unblinded SUSARs, to prevent the unblinding of investigators and the Sponsor/CRO, an independent group may be required to perform the reporting and this will be detailed in the Lifecycle Safety plan [which is in accordance with ICH-GCP, National laws and site specific requirements]

12.3.4 Periodic Safety Reports and End of Study Notification

The IEC/IRB will be sent periodic/annual safety updates in order to facilitate their continuing review of the study (reference. ICH GCP Section 3.1.4) according to the requirements of each of the countries involved and will also be informed about the end of the study, within the required timelines.

12.4 Regulatory Authority Approval

The study will be performed in compliance with each country's regulatory requirements. As with the IEC/IRB, clinical study authorisation from the appropriate Regulatory Authorities must be obtained prior to the start of the study. In addition, the Regulatory Authorities must approve amendments (as instructed by the Sponsor), receive SUSAR reports, and be notified of the end of the study.

12.5 Other Required Approvals

In addition to IEC/IRB and regulatory authority approval, any other locally required approvals will be obtained prior to recruitment of patients into the study and shipment of the IMP.

12.6 Insurance

The Sponsor has civil liability insurance, which covers this study in all participating countries.

12.7 Pre-study Documentation Requirements

The documents required before the IMP can be shipped to the investigational site will be collected in accordance with the SOPs of the contracted CRO and the requirements of each country.

12.8 Informed Consent

It is the Investigator's responsibility to obtain written informed consent from the patient/patient's LAR after adequate explanation of the aims, methods, anticipated benefits, and potential hazards of the study and before any study procedures are commenced. The patient/patient's LAR should be given a copy of the Patient Information Sheet and Informed Consent in their native language. The original copy of the signed and dated informed consent must be retained in the institution's records, and is subject to inspection by representatives of the Sponsor, or representatives from Regulatory Authorities. See Section 5.3.1.

12.9 Contact with General Practitioner/Primary Care Physician

It is the Investigator's responsibility to inform the patient's General Practitioner/Primary Care Physician (where applicable) by letter that the patient is taking part in the study provided the patient agrees to this, and information to this effect is included in the Patient Information Sheet and Informed Consent. A copy of the letter should be filed in the Investigator Site File.

12.10 Patient Confidentiality

The Investigator must ensure that the patient's privacy is maintained. On the eCRF or other documents submitted to the Sponsors, patients will be identified by a patient ID number only. Documents that are not submitted to the Sponsor (e.g., signed informed consent form) should be kept in a strictly confidential file by the Investigator.

The Investigator shall permit direct access to patients' records and source document for the purposes of monitoring, auditing, or inspection by the Sponsor, authorised representatives of the Sponsor, Regulatory Authorities and IECs/IRBs.

12.11 Data Protection

All personnel involved in the study will observe or work within the confines of the local data protection regulations.

12.12 End of Study

The end of the study shall be defined as the Last Patient Last Visit.

12.13 Study Documentation and Data Storage

The Investigator must retain a comprehensive and centralised filing system of all study-related documentation that is suitable for inspection by the Sponsor and representatives of Regulatory Authorities.

The Investigator must retain essential documents until notified by the Sponsor, and at least for five years after study completion, as per Directive 2005/28/EC Article 17. Patient files and other source data (including copies of protocols, eCRFs, original reports of test results, IMP dispensing logs, correspondence, records of informed consent, and other documents pertaining to the conduct of the study) must be kept for the maximum period of time permitted by the institution. Documents should be stored in such a way that they can be accessed/data retrieved at a later date. Consideration should be given to security and environmental risks.

No study document will be destroyed without prior written agreement between the Sponsor and the Investigator. Should the Investigator wish to assign the study records to another party or move them to another location, written agreement must be obtained from the Sponsor.

12.14 Disclosure of Information

Information concerning the study, patent applications, processes, scientific data or other pertinent information is confidential and remains the property of the Sponsor. The Investigator may use this information for the purposes of the study only.

It is understood by the Investigator that the Sponsor will use information developed in this clinical study in connection with the development of the IMP and, therefore, may disclose it as required to other clinical Investigators and to Regulatory Authorities. In order to allow the use of the information derived from this clinical study, the Investigator understands that he/she has an obligation to provide complete test results and all data developed during this study to the Sponsor.

Verbal or written discussion of results prior to study completion and full reporting should only be undertaken with written consent from the Sponsor.

12.15 Publication

All information concerning OZ439 and the Sponsor’s operations, such as the Sponsor patent applications, formulas, manufacturing processes, basic scientific data, or formulation information, supplied by the Sponsor and not previously published is considered confidential information.
The information developed during the conduct of this clinical study is also considered confidential and will be used by the Sponsor in connection with the development of OZ439. This information may be disclosed as deemed necessary by the Sponsor. To allow for the use of the information derived from this clinical study and to ensure complete and thorough analysis, the Investigator is obligated to provide the Sponsor with complete test results and all data developed in this study.
This confidential information shall remain the sole property of the Sponsor, shall not be disclosed to others without the written consent of the Sponsor, and shall not be used except in the performance of this study.
The Sponsor encourages publication of study results. However no study data shall be disclosed prior to the final analysis of the data and only on express permission of the Sponsor. Should an Investigator wish to publish results from this study, a copy of the manuscript must be provided to the Sponsor for approval, at least 30 days before the intended date of submission to the intended publisher.

13 ADMINISTRATIVE OBLIGATIONS

13.1 Source Data

Where source documents (such as laboratory reports, medical records or ECGs) or laboratory databases exist, all relevant data will be transcribed into the eCRF, transferred electronically to the study database or entered into the study database directly from source documents. Where no source documents exist, data will be written directly into the eCRF.

The Investigator/institution will permit study-related monitoring, audits/inspections, IEC/IRB review and regulatory inspection providing direct access to source documents.

13.2 Language

eCRFs will be in English. Generic names for concomitant medications should be recorded in the eCRF wherever possible. All written material to be used by patients must use vocabulary that is clearly understood, and be in the language appropriate for the study site.

13.3 Data Collection

All data will be entered electronically into an eCRF. The Investigator will indicate a thorough inspection of the data in the eCRF has taken place, and will certify the content.

All laboratory reports (if applicable) will be reviewed, signed and dated by the Investigator.

13.4 Monitoring

It is understood that monitors, and any authorised personnel contracted to the Contract Research Organisation may contact and visit the Investigator, and that they will be allowed to inspect the various records of the study on request (eCRFs and other pertinent data), provided that patient confidentiality is maintained, and that the inspection is conducted in accordance with local regulations.

It is the monitor’s responsibility to inspect the eCRFs at regular intervals throughout the study to verify adherence to the protocol, the completeness, accuracy and consistency of the data, and adherence to regulatory requirements and ICH GCP guidelines.

The Investigator agrees to co-operate with the monitor to ensure that any problems detected during the course of these monitoring visits are resolved.

13.5 Quality Control and Quality Assurance

Quality Control will be performed according to the CRO’s internal procedures. The study will be audited (if applicable) by a Quality Assurance representative of the Sponsor. All necessary data and documents will be made available for inspection.

14 REFERENCES

Ariey F et al (2014) A molecular marker of artemisinin-resistant Plasmodium falciparum malaria. *Nature 505, 50–55.*

Ding XC, Beck H-P, Raso G (2011). Plasmodium sensitivity to artemisinins: magic bullets hit elusive targets. *Trends in Parasitology* 27 (2) 73-81.

Food and Drug Administration, Center for Drug Evaluation and Research (CDER) Center for Biologics Evaluation and Research (CBER) Guidance for Industry Drug-Induced Liver Injury: Premarketing Clinical Evaluation (2009).

Jambou R, Le Bras J, Randrianarivelojosia M. (2011). Pitfalls in new artemisinin-containing antimalarial drug development. *Trends Parasitol*. 2011 Feb;27(2):82-90. Epub 2010 Oct 26.

Lee, J. J., & Liu, D. D. (2008). A predictive probability design for phase II cancer clinical trials. *Clinical Trials*, *5*(2), 93-106.

OZ439 Clinical Investigator's Brochure (Current Edition)

Price, R. N., Hasugian, A. R., Ratcliff, A., Siswantoro, H., Purba, H. L. E., Kenangalem, E., ... & Tjitra, E. (2007). Clinical and pharmacological determinants of the therapeutic response to dihydroartemisinin-piperaquine for drug-resistant malaria. *Antimicrobial agents and chemotherapy*, *51*(11), 4090-4097.

Tarning, J., Zongo, I., Somé, F. A., Rouamba, N., Parikh, S., Rosenthal, P. J., ... & Lindegardh, N. (2012). Population pharmacokinetics and pharmacodynamics of piperaquine in children with uncomplicated falciparum malaria. *Clinical Pharmacology & Therapeutics*, *91*(3), 497-505.

Tarning, J., Rijken, M. J., McGready, R., Phyo, A. P., Hanpithakpong, W., Day, N. P., ... & Lindegardh, N. (2012). Population pharmacokinetics of dihydroartemisinin and piperaquine in pregnant and nonpregnant women with uncomplicated malaria. *Antimicrobial agents and chemotherapy*, *56*(4), 1997-2007.

Van Vugt, M., Wilairatana, P., Gemperli, B., Gathmann, I., Phaipun, l., Brockman, A., Luxemburger, C.,White, N. J. & Nosten, F. (1999). Efficacy of six doses of artemether benflumetol in the treatment of multi-drug resistant falciparum malaria. *American Journal of Tropical Medicine and Hygiene*, 60, 936± 942.

WHO guidelines (WHO 2010): www.int/malaria

WHO guidelines (WHO 2013): www.int/malaria

WHO 2013: Management of severe malaria: a practical handbook – 3rd ed. ISBN 978 92 4 154852 6

WFP (2005) World Food Programme and Centers for Disease Control and Prevention. A Manual: Measuring and Interpreting Malnutrition and Mortality: www.micronutrient.org

ATTACHMENT 1: TOXICITY GRADING SCALES FOR DETERMINING SEVERITY OF ADVERSE EVENTS

Division Of Microbiology And Infectious Diseases (DMID) Adult Toxicity Tables

Abbreviations: Abbreviations utilised in the Table:

ULN = Upper Limit of Normal LLN = Lower Limit of Normal

R_x = Therapy Req = Required

Mod = Moderate IV = Intravenous

ADL = Activities of Daily Living Dec = Decreased

Estimating Severity Grade

For abnormalities NOT found elsewhere in the Toxicity Tables use the scale below to estimate grade of severity:

GRADE 1 Mild Transient or mild discomfort

(< 48 hours); no medical intervention/therapy required

**GRADE 2 Moderate**	Mild to moderate limitation in activity - some assistance may be needed; no or minimal medical intervention/therapy required
**GRADE 3 Severe**	Marked limitation in activity, some assistance usually required; medical intervention/therapy required, hospitalisations possible
**GRADE 4 Life-threatening**	Extreme limitation in activity, significant assistance required; significant medical intervention/therapy required, hospitalisation or hospice care probable

Serious or Life-threatening AEs

ANY clinical event deemed by the clinician to be serious or life-threatening should be considered a grade 4 event. Clinical events considered to be serious or life-threatening include, but are not limited to: seizures, coma, tetany, diabetic ketoacidosis, disseminated intravascular coagulation, diffuse petechiae, paralysis, acute psychosis, severe depression.

HAEMATOLOGY				
	Grade 1	Grade 2	Grade 3	Grade 4
Haemoglobin	9.5 - 10.5 gm/dL	8.0 - 9.4gm/dL	6.5 - 7.9 gm/dL	< 6.5 gm/dL
Absolute Neutrophil Count	1000-1500/mm^3	750-999/mm^3	500-749/mm^3	<500/mm^3
Platelets	75,000-99,999/mm^3	50,000-74,999/mm^3	20,000-49,999/mm^3	<20,000/mm^3
WBCs	> 13,000/ mm^3	13,000-15,000 /mm^3	15,000-30,000/mm^3	>30,000 or <1,000 /mm^3
% Polymorphonuclear Leucocytes + Band Cells	> 80%	90 – 95%	>95%	----------
Abnormal Fibrinogen	Low: 100-200 mg/dL High: 400-600 mg/dL	Low: <100 mg/dL High: >600 mg/dL	Low: < 50 mg/dL ----------	Fibrinogen associated with gross bleeding or with disseminated coagulation
Fibrin Split Product	20-40 µg/mL	41-50 µg/mL	51-60 µg/mL	> 60 µg/mL
Prothrombin Time (PT)	1.01 - 1.25 x ULN	1.26-1.5 x ULN	1.51 -3.0 x ULN	>3 x ULN
Activated Partial Thromboplastin (APPT)	1.01 -1.66 x ULN	1.67 - 2.33 x ULN	2.34 - 3 x ULN	> 3 x ULN
Methaemoglobin	5.0 - 9.9 %	10.0 - 14.9 %	15.0 - 19.9%	> 20.0 %

CHEMISTRIES				
	Grade 1	Grade 2	Grade 3	Grade 4
Hyponatraemia	130-135 mEq/L	123-129 mEq/L	116-122 mEq/L	< 116 mEq/L or abnormal sodium with mental status changes or seizures
Hypernatraemia	146-150 mEq/L	151-157 mEq/L	158-165 mEq/L	> 165 mEq/L or abnormal sodium with mental status changes or seizures
Hypokalaemia	3.0 - 3.4 mEq/L	2.5 - 2.9 mEq/L	2.0 - 2.4 mEq/L<br>or intensive replacement therapy or hospitalisatio n required	< 2.0 mEq/L or abnormal potassium with<br>paresis, ileus or life-threatening arrhythmia
Hyperkalaemia	5.6 - 6.0 mEq/L	6.1 - 6.5 mEq/L	6.6 - 7.0 mEq/l	> 7.0 mEq/L<br>or abnormal potassium with life-threatening arrhythmia

**CHEMISTRIES (Continued)**				
	Grade 1	Grade 2	Grade 3	Grade 4
Hypoglycaemia	55-64 mg/dL	40-54 mg/dL	30-39 mg/dL	<30 mg/dL or abnormal glucose with mental status changes or coma
Hyperglycaemia<br>(nonfasting and no prior diabetes)	116 - 160 mg/dL	161- 250 mg/dL	251 - 500 mg/dL	> 500 mg/dL or abnormal glucose with ketoacidosis<br>or seizures
Hypocalcaemia<br>(corrected for albumin)	8.4 - 7.8 mg/dL	7.7 - 7.0 mg/dL	6.9 - 6.1 mg/dL	< 6.1 mg/dL<br>or abnormal calcium with life threatening arrhythmia or tetany
Hypercalcaemia<br>(correct for albumin)	10.6 - 11.5 mg/dL	11.6 - 12.5 mg/dL	12.6 - 13.5 mg/dL	> 13.5 mg/dL or abnormal calcium with life<br>threatening<br>arrhythmia
Hypomagnesaemia	1.4 - 1.2 mEq/L	1.1 - 0.9 mEq/L	0.8 - 0.6 mEq/L	< 0.6 mEq/L or abnormal magnesium with life-threatening arrhythmia
Hypophosphataemia	2.0 - 2.4 mg/dL	1.5 -1.9 mg/dL or<br>replacement Rx required	1.0 -1.4 mg/dL<br>intensive therapy or hospitalisation required	< 1.0 mg/dL or abnormal phosphate with life-threatening arrhythmia

Hyperbilirubinaemia	1.1 - 1.5 x ULN	1.6 - 2.5 x ULN	2.6 - 5 x ULN	> 5 x ULN
BUN	1.25 - 2.5 x ULN	2.6 - 5 x ULN	5.1 - 10 x ULN	> 10 x ULN
Hyperuricaemia (uric acid)	7.5 – 10.0 mg/dL	10.1 – 12.0 mg/dL	12.1 – 15.0 mg/dL	>15.0 mg/dL
Creatinine	1.1 - 1.5 x ULN	1.6 - 3.0 x ULN	3.1 - 6 x ULN	> 6 x ULN or dialysis required

**ENZYMES**				
	Grade 1	Grade 2	Grade 3	Grade 4
AST (SGOT)	1.25 - 2.5 x ULN	2.6 - 5 x ULN	5.1 - 10 x ULN	> 10 x ULN
ALT (SGPT)	1.25 - 2.5 x ULN	2.6 - 5 x ULN	5.1 - 10 x ULN	> 10 x ULN
GGT	1.25 -2.5 x ULN	2.6 - 5 x ULN	5.1 - 10 x ULN	> 10 x ULN
Alkaline Phosphatase	1.25 - 2. 5 x ULN	1.6 - 5 x ULN	5.1 - 10 x ULN	> 10 x ULN
Amylase	1.1 - 1.5 x ULN	1.6 - 2.0 x ULN	2.1 - 5.0 x ULN	> 5.1 x ULN
Lipase	1.1 - 1.5 x ULN	1.6 - 2.0 x ULN	2.1 - 5.0 x ULN	> 5.1 x ULN

**URINALYSIS**				
	Grade 1	Grade 2	Grade 3	Grade 4
Proteinuria	1+<br>or<br>200 mg - 1 gm loss/day	2-3+<br>or<br>1- 2 gm loss/day	4+<br>or<br>2-3.5 gm loss/day	nephrotic syndrome<br>or<br>> 3.5 gm loss/day
Haematuria	microscopic only<br><10 rbc/hpf	gross, no clots<br>>10 rbc/hpf	gross, with or without clots, OR red blood cell casts	obstructive or required transfusion

**CARDIOVASCULAR**				
	Grade 1	Grade 2	Grade 3	Grade 4
Cardiac Rhythm		asymptomatic, transient signs, no<br>Rx required	recurrent/persistent;<br>symptomatic Rx required	unstable dysrhythmia; hospitalisation and treatment required
Hypertension	transient increase<br>> 20 mm/Hg; no treatment	recurrent, chronic increase<br>> 20mm/Hg.<br>/treatment required	acute treatment required; outpatient treatment or hospitalisation possible	end organ damage or hospitalisation required
Hypotension	transient orthostatic hypotension with heart rate increased by <20 beat/min or decreased by <10 mm Hg systolic BP, No treatment required	symptoms due to orthostatic hypotension or BP decreased by <20 mm Hg systolic; correctable with oral fluid treatment	requires IV fluids; no hospitalisation required	mean arterial pressure<br><60mm/ Hg or end organ damage or shock; requires hospitalisation and vasopressor treatment

Pericarditis	minimal effusion	mild/moderate asymptomatic effusion, no treatment	symptomatic effusion; pain; EKG changes	tamponade; pericardiocentesis or surgery required
Haemorrhage, Blood Loss	microscopic/occult	mild, no transfusion	gross blood loss;<br>1-2 units transfused	massive blood loss; > 3 units transfused

**RESPIRATORY**				
	Grade 1	Grade 2	Grade 3	Grade 4
Cough	transient- no treatment	persistent cough;<br>treatment responsive	Paroxysmal cough; uncontrolled with treatment	--------------------
Bronchospasm, Acute	transient; no treatment;<br>70% - 80% FEV_1<br>of peak flow	requires treatment; normalises with bronchodilator;<br>FEV_1 50% - 70%<br>(of peak flow)	no normalisation with bronchodilator;<br>FEV_1 25% - 50%<br>of peak flow; or retractions present	cyanosis: FEV_1<br>< 25%<br>of peak flow or intubation necessary
Dyspnoea	dyspnoea on exertion	dyspnoea with normal activity	dyspnoea at rest	dyspnoea requiring Oxygen therapy

**GASTROINTESTINAL**				
	Grade 1	Grade 2	Grade 3	Grade 4
Nausea	mild or transient; maintains reasonable intake	moderate discomfort; intake decreased significantly; some activity limited	no significant intake; requires IV fluids	hospitalisation required;
Vomiting	1 episode in 24 hours	2-5 episodes in 24 hours	>6 episodes in 24 hours or needing IV fluids	physiologic consequences requiring hospitalisation or requiring parenteral nutrition
Constipation	requiring stool softener or dietary modification	requiring laxatives	obstipation requiring manual evacuation or enema	obstruction or toxic megacolon
Diarrhoea	mild or transient; 3-4 loose stools/day or mild diarrhoea last < 1 week	moderate or persistent; 5-7 loose stools/day or diarrhoea lasting >1 week	>7 loose stools/day or bloody diarrhoea; or orthostatic hypotension or electrolyte imbalance or >2L IV fluids required	hypotensive shock or physiologic consequences requiring hospitalisation
Oral Discomfort/ Dysphagia	mild discomfort; no difficulty swallowing	some limits on eating/drinking	eating/talking very limited; unable to swallow solid foods	unable to drink fluids; requires IV fluids

NEUROLOGICAL				
	Grade 1	Grade 2	Grade 3	Grade 4
Neuro-Cerebellar	slight incoordination dysdiadochokinesis	intention tremor, dysmetria, slurred speech; nystagmus	locomotor ataxia	incapacitated
Psychiatric	mild anxiety or depression	moderate anxiety or depression; therapy required; change in normal routine	severe mood changes requiring therapy; or suicidal ideation; or aggressive ideation	acute psychosis requiring hospitalisation; or suicidal gesture/attempt or hallucinations
Muscle Strength	subjective weakness<br>no objective symptoms/ signs	mild objective<br>signs/symptoms<br>no decrease in function	objective weakness function limited	paralysis
Paresthesia (burning, tingling, etc.)	mild discomfort; no treatment required	moderate discomfort; non-narcotic analgesia required	severe discomfort; or narcotic analgesia required with symptomatic improvement	incapacitating; or not responsive to narcotic analgesia
Neuro-sensory	mild impairment in sensation (decreased sensation, e.g., vibratory, pinprick, hot/cold in great toes) in focal area or symmetrical distribution; or change in taste, smell, vision and/or hearing	moderate impairment (mod decreased sensation, e.g., vibratory, pinprick, hot/cold to ankles) and/or joint position or mild impairment that is not symmetrical	severe impairment (decreased or loss of sensation to knees or wrists) or loss of sensation of at least mod degree in multiple different body areas (i.e., upper and lower extremities)	sensory loss involves limbs and trunk; paralysis; or seizures

MUSCULOSKELETAL				
	Grade 1	Grade 2	Grade 3	Grade 4
Arthralgia (joint pain)	mild pain not interfering with function	moderate pain, analgesics and/or pain interfering with function but not with activities of daily living	severe pain; pain and/or analgesics interfering with activities of daily living	disabling pain
Arthritis	mild pain with inflammation, erythaema or joint swelling – but not interfering with function	moderate pain with inflammation, erythaema or joint swelling – interfering with function, but not with activities of daily living	severe pain with inflammation, erythaema or joint swelling – and interfering with activities of daily living	permanent and/or disabling joint destruction
Myalgia	myalgia with no limitation of activity	muscle tenderness (at other than injection site) or with moderate impairment of activity	severe muscle tenderness with marked impairment of activity	frank myonecrosis

**SKIN**				
	Grade 1	Grade 2	Grade 3	Grade 4
Mucocutaneous	erythaema; pruritus	diffuse, maculo papular rash, dry desquamation	vesiculation or moist desquamation or ulceration	exfoliative dermatitis, mucous membrane involvement or erythaema, multiforme or suspected Stevens-Johnson or necrosis requiring surgery
Induration	< 15mm	15-30 mm	>30mm	
Erythaema	< 15mm	15-30 mm	>30mm	
Oedema	< 15mm	15-30 mm	>30mm	
Rash at Injection Site	< 15mm	15-30 mm	>30mm	
Pruritus	slight itching at injection site	moderate itching at injection extremity	itching over entire body	

**SYSTEMIC**				
	Grade 1	Grade 2	Grade 3	Grade 4
Allergic Reaction	pruritus without rash	localised urticaria	generalised urticaria; angioedema	anaphylaxis
Headache	mild, no treatment required	transient, moderate; treatment required	severe; responds to initial narcotic therapy	intractable; requires repeated narcotic therapy
Fever: oral	37.7 - 38.5 C or 100.0 - 101.5 F	38.6 - 39.5 C or 101.6 - 102.9 F	39.6 - 40.5 C or 103 - 105 F	> 40 C or > 105 F
Fatigue	normal activity reduced < 48 hours	normal activity decreased 25-50% > 48 hours	normal activity decreased > 50% can't work	unable to care for self

Division Of Microbiology And Infectious Diseases (DMID) Pediatric Toxicity Tables

ABBREVIATIONS: Abbreviations utilized in the Table:

ULN = Upper Limit of Normal	LLN = Lower Limit of Normal
R_x = Therapy	Req = Required
Mod = Moderate	IV = Intravenous
ADL = Activities of Daily Living	Dec = Decreased

ESTIMATING SEVERITY GRADE

For abnormalities NOT found elsewhere in the Toxicity Tables use the scale below to estimate grade of severity:

**GRADE 1**	**Mild** Transient or mild discomfort (< 48 hours); no medical intervention/therapy required
**GRADE 2**	**Moderate** Mild to moderate limitation in activity - some assistance may be needed; no or minimal medical intervention/therapy required
**GRADE 3**	**Severe** Marked limitation in activity, some assistance usually required; medical intervention/therapy required, hospitalizations possible
**GRADE 4**	**Life-threatening** Extreme limitations in activity, significant assistance required; significant medical intervention/therapy required, hospitalization or hospice care probable
**GRADE 5**	**Death**

SERIOUS OR LIFE-THREATENING AEs

ANY clinical event deemed by the clinician to be serious or life-threatening should be considered a grade 4 event. Clinical events considered to be serious or life-threatening include, but are not limited to: seizures, coma, tetany, diabetic ketoacidosis, disseminated intravascular coagulation, diffuse petechiae, paralysis, acute psychosis, severe depression.

- For parameters not included in the following Toxicity Tables, sites should refer to the "Guide For Estimating Severity Grade" located above.

Division of Microbiology and Infectious Diseases (DMID) Pediatric Toxicity Tables, Greater Than 3 Months of Age:

LOCAL REACTIONS				
	**GRADE 1**	**GRADE 2**	**GRADE 3**	**GRADE 4**
Induratio	<	10-25	26-50mm	>50mm
Erythema	<	10-25	26-50mm	>50mm
Edema	<	10-25	26-50mm	>50mm
Rash at Injection Site	< 10mm	10-25 mm	26-50mm	>50mm
Pruritus	Slight itching at injection site	Moderate itching at injection extremity	Itching at injection extremity and other sites	Itching over entire body

HEMATOLOGY				
	**GRADE 1**	**GRADE 2**	**GRADE 3**	**GRADE 4**
Hemoglobin for children greater than months and less than 2 years of age	9.0-9.9 gm/dL	7.0-8.9 gm/dL	<7.0 gm/dL	Cardiac Failure secondary to anemia
Hemoglobin for children greater than 2 years of age	10-10.9 gm/dL	7.0-9.9 gm/dL	<7.0 gm/dL	Cardiac Failure secondary to anemia
Absolute Neutrophil Count	750-1200/ mm^3	400-749/ mm^3	250-399/ mm^3	<250/ mm^3
Platelets	-- --	50,000-75,000/ mm^3	25,000-49,999/ mm^3	<25,000/ mm^3

Prothrombin Time (PT)	1.1-1.2 x ULN	1.3 -1.5 x ULN	1.6 -3.0 x ULN	>3.0 x ULN
Partial Thromboplastin Time (PTT)	1.1-1.6 x ULN	1.7-2.3 x ULN	2.4 -3.0 x ULN	>3.0 x ULN

GASTRO INTESTINAL				
	**GRADE 1**	**GRADE 2**	**GRADE 3**	**GRADE 4**
Bilirubin (when accompanied by any increase in other liver function test)	1.1 - <1.25 x ULN	1.25 - <1.5 x ULN	1.5 – 1.75 x ULN	> 1.75 x ULN
Bilirubin (when other liver function are in the normal range)	1.1 - <1.5 x ULN	1.5 - <2.0 x ULN	2.0 – 3.0 x ULN	> 3.0 x ULN
AST (SGOT)	1.1 - <2.0 x ULN	2.0 – <3.0 x ULN	3.0 – 8.0 x ULN	> 8 x ULN
ALT (SGPT)	1.1 - <2.0 x ULN	2.0 – <3.0 x ULN	3.0 – 8.0 x ULN	> 8 x ULN
GGT	1.1 - <2.0 x ULN	2.0 – <3.0 x ULN	3.0 – 8.0 x ULN	> 8 x ULN
Pancreatic Amylase	1.1-1.4 x ULN	1.5-1.9 x ULN	2.0-3.0 x ULN	>3.0 x ULN
Uric Acid	7.5-9.9mg/dL	10-12.4 mg/dL	12.5-15.0 mg/dL	>15.0 mg/dL
CPK	See Neuro muscular Toxicity			

Appetite	----------------	Decreased appetite	Appetite very decreased, no solid food taken	No solid or liquid taken
Abdominal Pain	Mild	Moderate- No Treatment Needed	Moderate- Treatment Needed	Severe- Hospitalized for treatment
Diarrhea	Slight change in consistency and/or frequency of s tools	Liquid stools	Liquid stools greater that 4x the amount or number normal for this child	Liquid stools greater than 8x the amount or number normal for this child

**GASTRO INTESTINAL (continued)**				
	**GRADE 1**	**GRADE 2**	**GRADE 3**	**GRADE 4**
Constipation	Slight change in the consistency/frequency of stool	Hard, dry stools with a change in frequency	Abdominal pain	Distention and Vomiting
Nausea	Mild	Moderate- Decreased oral intake	Severe-Little oral intake	Unable to ingest food or fluid for more than 24 hours
Vomiting	1 episode/day	2-3 episodes per day	4-6 episodes per day	Greater than 6 episodes per day or Intractable Vomiting

ELECTROLYTES				
	**GRADE 1**	**GRADE 2**	**GRADE 3**	**GRADE 4**
CREATININE				
3 Months -2 Years of age	0.6-0.8 x ULN	0.9-1.1 x ULN	1.2-1.5 x ULN	>1.5 x ULN
2 Years- 12 Years of age	0.7-1.0 x ULN	1.1-1.6 x ULN	1.7-2.0 x ULN	>2.0 x ULN
Greater than 12 Years of age	1.0-1.7 x ULN	1.8-2.4 x ULN	2.5-3.5 x ULN	>3.5 x ULN

ELECTROLYTES				
	**GRADE 1**	**GRADE 2**	**GRADE 3**	**GRADE 4**
Hypernatremia		<145-149 mEq/L	150-155 mEq/L	>155 mEq/L or abnormal sodium AND mental status changes
Hyponatremia		130-135 mEq/L	129-124 mEq/L	<124 mEq/L or abnormal sodium AND mental status changes
Hyperkalemia	5.0-5.9 mEq/L	6.0-6.4 mEq/L	6.5-7.0 mEq/L	>7.0 mEq/L or abnormal potassium AND cardiac arrhythmia
Hypokalemia	3.0-3-5 mEq/L	2.5-2.9 mEq/L	2.0-2.4 mEq/L	<2.0 mEq/L or abnormal potassium AND cardiac arrhythmia
Hypercalcemia	10.5-11.2mg/dL	11.3-11.9 mg/dL	12.0-12.9 mg/dL	>13.0 mg/dL

Hypocalcemia	7.8-8.4 mg/d L	7.0-7.7 mg/d L	6.0-6.9 mg/d L	<6.0 mg/dL
Hypomagnese mia	1.2-1.4 mEq/ L	0.9-1.1 mEq/ L	0.6-0.8 mEq/ L	<0.6 mEq/ L or abnormal magnesium AND cardiac arrhythmia
Hypoglycemia	55-65 mg/dL	40-54 mg/dL	30-39 mg/dL	<30 mg/d L or abnormal glucose AND mental status changes
Hyperglycemia	116-159 mg/dL	160-249 mg/dL	250-400 mg/dL	>400 mg/dL or ketoacidos is
Proteinuria	Tr-1+ Or <150 mg/day	2+ Or 150-499 mg/day	3+ Or 500-1000 mg/day	4+ Or Nephrotic syndrome >1000 mg/day
Hematuria	Microscopic <25	Microscopic >25		Gross hematuria

CENTRAL NERVOUS SYSTEM (CNS)				
	**GRADE 1**	**GRADE 2**	**GRADE 3**	**GRADE 4**
Generalized CNS Symptoms			Dizziness	Hypotonic, hyporesponsive episodes; Seizures; Apnea/Bradycardia; Inconsolable crying > 3 hrs;
Headache	Mild	Moderate, Responds to non-narcotic analgesia	Moderate to Severe, Responds to narcotic analgesia	Intractable

Level of Activity		Slightly irritable OR slightly subdued	Very irritable OR Lethargic	Inconsolable OR Obtunded
Visual		Blurriness, diplopia, or horizontal nystagmus of < 1 hour duration, with spontaneous resolution	More than 1 episode of Grade 2 symptoms per week, or an episode of Grade 2 symptoms lasting more than 1 hour with spontaneous resolution by 4 hours or vertical nystagmus	Decrease in visual acuity, visual field deficit, or oculogyric crisis
Myelopathy		None	None	Myelopathic/spinal cord symptoms, such as: pyramidal tract weakness and dis inhibition, sensory level, loss of proprioception, bladder/bowel dysfunction

PERIPHERAL NERVOUS SYSTEM				
**PARAMETER**	**GRADE 1**	**GRADE 2**	**GRADE 3**	**GRADE 4**
Neuropathy/ Lower Motor Neuropathy		Mild transient Paresthesia only	Persistent or progressive paresthesias, burning sensation in feet, or mild dysesthesia; no weakness; mild to moderate deep tendon reflex changes; no sensory loss	Onset of significant weakness, decrease or loss of DTRs, sensory loss in "stocking glove" distribution, radicular sensory loss, multiple cranial nerve involvement; bladder or bowel dysfunction, fasciculations, respiratory embarrassment from chest wall weakness
Myopathy or Neuro muscular Junction Impairment	Normal or mild (<2 x ULN) CPK elevation	Mild proximal weakness and/or atrophy not affecting gross motor function. Mild myalgias, +/- mild CPK elevation (<2 x ULN)	Proximal muscle weakness and/or atrophy affecting motor function +/- CPK elevation; or severe myalgias with CPK >2 x ULN;	Onset of myasthenia- like symptoms (fatigable weakness with external, variable ophthalmoplegia and/or ptosis), or neuromuscular junction blockade (acute paralysis) symptoms

OTHER				
	**GRADE 1**	**GRADE 2**	**GRADE 3**	**GRADE 4**
Allergy	Pruritus without Rash	Pruritic Rash	Mild Urticaria	Severe Urticaria Anaphylaxis, Angioedema
Drug Fever (Rectal)	.	38.5-40C 101.3 – 104.0F	Greater than 40.0C Greater than 104.0F	Sustained Fever: Equal or greater than 40C (104.0F) for longer than 5 days
Cutaneous	Localized rash	Diffuse maculopapular Rash	Generalized urticaria	Stevens-Johnson Syndrome or Erythema multiforme
Stomatitis	Mild discomfort	Painful, difficulty swallowing, but able to eat and drink	Painful: unable to swallow solids	Painful: unable to swallow liquids; requires IV fluids
Clinical symptoms *not otherwise specified* in this table	No therapy; monitor condition	May require minimal intervention and monitoring	Requires medical care and possible hospitalization	Requires active medical intervention, hospitalization, or hospice care

Laboratory values *not otherwise specified* in this table	Abnormal, but requiring no immediate intervention; follow	Sufficiently abnormal to require evaluation as to causality and perhaps mild therapeutic intervention, but not of sufficient severity to warrant immediate changes in study drug	Sufficiently severe to require evaluation and treatment, including at least temporary suspension of study drug	Life–threatening severity; Requires immediate evaluation, treatment, and usually hospitalization; Study drug must be stopped immediately and should not be restarted until the abnormality is clearly felt to be caused by some other mechanism than study drug

ATTACHMENT 2: HY'S LAW PROCEDURES:

Check List of actions to be done in case of drug induced liver serious reactions **(Hy's law)**

1. Stop herbal remedies and concomitant medications if applied and are not medically necessary.
2. Notify appropriate lines
   a. Ethics committees
   b. Regulatory Authority
   c. Local Medical Monitor and DSMB
   d. Quintiles Lifecycle Safety
3. Urgently admit patient and collect urine, blood for PK (within 24 hours of last dose) for diagnostic tests
4. Request liver imaging (ultrasound, computerised tomography or magnetic resonance imaging)
5. Obtain additional history and review of medical records
6. Discuss details with investigator, in particular any concomitant medications, OTC medications, herbal remedies, prior exposure, previous episodes etc.
7. Documentation of laboratory values, ECG tracings, pathology reports, PK etc.
8. Ensure appropriate medical attention, including move to the ICU and referral to a more specialised hospital.
9. Arrange appropriate follow up for investigations.
10. DSMB to review aggregate data from available sources for underlying trends.
11. Contact external experts for advice if possible.
12. Documentation needed in the event of a clinically significant elevation in one or more liver function tests
13. Liver function test results that are codified as adverse events, and/or elevations that occur rapidly or exceed multiples of the upper limits of normal, merit further inquiry on the part of the investigator. The laboratory tests that are relevant in this regard include:
   a. ALT (SGPT), AST (SGOT), GGT, and alkaline phosphatase (ALP). Examples of elevations that may require more intensive inquiry on the part of the investigator include an ALT value that exceeds 5.0 times the upper limit of normal or an ALT value that exceeds 3.0 times the upper limit of normal
   b. Total bilirubin value that exceeds 2 times the upper limit of normal (Hy's law). With massive hepatic injury, the ability of the liver to excrete conjugated (or direct) bilirubin diminishes.
   c. Therefore, the presence of ALT >3xULN and concomitant bilirubin >2xULN (>35% direct), in the absence of elevated alkaline phosphatase or biliary injury, suggests significant liver injury, <u>mandating the need for specialist consultation, and follow up</u> of liver chemistries and prothrombin time twice weekly until values normalise or substantively improve.
14. If laboratory values are returned that meet the above-described criteria, the investigator should obtain a viral hepatitis panel (see below). In addition, the investigator should attempt to document the following in the eCRF prior to contacting the sponsor. The documentation should have the following details:
   a. The date (and visit number) on which the blood sample was obtained.

b. The Randomisation date as well as the dose level that the patient is currently taking, and the duration of exposure to that dose level. The exact date on which the patient took the study medication and the confirmation of the patient's compliance with the study medication.

c. The specific abnormal laboratory values, as well as those of each of the other LFTs noted above (regardless of value) and, when relevant, results of isoenzyme or fractionation analyses. The investigator should also document the corresponding laboratory values at screening/baseline.

d. Other notable abnormalities in laboratory values (*e.g.*, complete blood count, eosinophilia, or electrolyte abnormalities, if present).

e. The dates and nature of any relevant adverse events (*e.g.*, jaundice) that occurred since Randomisation, with particular attention to hypotension, fever, rash, hepatitis symptoms (e.g. appearance or worsening of fatigue, nausea, anorexia, nausea, emesis, abdominal pain), or other adverse events that might have occurred in close proximity to the elevation in the laboratory value(s) of interest.

f. Any associated physical findings (including results of any exams or evaluations, including heart rate, blood pressure, temperature, and abdominal exam).

g. The use of concomitant medications (*e.g.*, acetaminophen, traditional and herbal remedies, other over the counter medications, or putative hepatotoxins) since Randomisation, as well as the dates of exposure to the concomitant medication(s). Please include any nutritional supplements, vitamins and/or herbal preparations that the patient might have taken during this time frame.

h. A statement concerning whether the patient has consumed alcohol since the time of Randomisation, with a description of frequency and intensity, if relevant. A blood alcohol level should be obtained if the patient's history and/or clinical presentation suggest proximal use or intoxication with alcohol.

i. Any history on the patient's part of prior elevations in any of the relevant laboratory values. Provide actual dates and laboratory values.

j. Past or recent history of exposure to known factors that can cause, or are associated with, elevations in liver function tests. Examples of these factors include alcohol abuse and/or dependence, hepatitis (infectious or chemical), infectious mononucleosis, gallbladder disease, liver disease of any kind, jaundice, myocardial infarction, heart failure and/or episodes of hypotension.

k. Any family history of hepatitis (from any cause) or hepatotoxicity from medications.

15. Please contact the medical monitor if you have questions about the most appropriate course of action, and/or if you have questions as to whether the patient should be referred to a specialist for further evaluation.
16. It is anticipated that the investigator will follow any patient with clinically significant elevations of one or more liver function tests until there is clear evidence that the value(s) have stabilised and/or normalised. In addition, an explanation should be provided for any patients that are lost to follow up.

Viral hepatitis panel:

a. Hepatitis A IgM antibody, Hepatitis B surface antigen and Hepatitis B Core Antibody (IgM), Hepatitis C RNA, Hepatitis E IgM antibody
b. Cytomegalovirus IgM antibody
c. Epstein-Barr viral capsid antigen IgM antibody (or if unavailable, heterophile antibody or monospot testing obtained)
d. Serum creatine CPK and lactate dehydrogenase (LDH)

ATTACHMENT 3: SEVERE FALCIPARUM MALARIA

Management of severe malaria: a practical handbook – 3rd, World Health Organization, 2012 ISBN 978 92 4 154852 6

Malaria infections may cause vital organ dysfunction and death. Severe malaria is defined by clinical or laboratory evidence of vital organ dysfunction. Nearly all deaths from severe malaria result from infections with *P. falciparum*. Strict definitions of severe malaria have been published for epidemiological and research purposes, but, in practice, there should be a low threshold for starting parenteral treatment in any patient about whom a health care worker is concerned. Even if some of the laboratory measures are not available immediately, this should not delay the start of intensive treatment. A general overview of the features of severe malaria is shown below. Note that these manifestations can occur singly or, more commonly, in combination in the same patient.

Clinical features of severe malaria:
• Impaired consciousness (including unrousable coma);
• Prostration, i.e. generalised weakness so that the patient is unable to sit, stand or walk without assistance;
• Multiple convulsions: more than two episodes within 24h;
• Deep breathing and respiratory distress (acidotic breathing);
• Acute pulmonary oedema and acute respiratory distress syndrome;
• Circulatory collapse or shock, systolic blood pressure < 80mm Hg in adults and < 50mm Hg in children;
• Acute kidney injury;
• Clinical jaundice plus evidence of other vital organ dysfunction; and
• Abnormal bleeding.

High parasitaemia is undoubtedly a risk factor for death from falciparum malaria, but the relation between parasitaemia and prognosis varies according to the level of malaria transmission. In low-transmission areas, mortality from acute falciparum malaria begins to increase with parasite densities over 100 000/µl (~2.5% parasitaemia), whereas in areas of higher transmission much higher parasite densities may be well tolerated. Parasitaemia > 20% is associated with a high risk in any epidemiological context.

Laboratory and other findings:
• Hypoglycaemia (< 2.2mmol/l or < 40mg/dl);
• Metabolic acidosis (plasma bicarbonate < 15mmol/l);
• Severe normocytic anaemia (haemoglobin < 5g/dl, packed cell volume < 15% in children; <7g/dl, packed cell volume < 20% in adults);
• Haemoglobinuria;
• Hyperlactataemia (lactate > 5mmol/l);
• Renal impairment (serum creatinine > 265µmol/l); and
• Pulmonary oedema (radiological).

Who is at risk?
In high-transmission areas, the risk for severe falciparum malaria is greatest among young children and visitors (of any age) from nonendemic areas. In other areas, severe malaria is more evenly distributed across all age groups. Risk is increased in the second and third trimesters of pregnancy, in patients with HIV/AIDS and in people who have undergone splenectomy.
