# Supplementary material for: A randomised, double-blind clinical phase II trial of the efficacy, safety, tolerability and pharmacokinetics of a single dose combination treatment with artefenomel and piperaquine in adults and children with uncomplicated Plasmodium falciparum malaria
Source: BMC Med. 2017 Oct 9;15:181. doi: 10.1186/s12916-017-0940-3 (PMC5632828; doi:10.1186/s12916-017-0940-3)
Supplement: Supplementary file 2 — Statistical analysis plan. (PDF 1515 kb) [file 12916_2017_940_MOESM2_ESM.pdf]

STATISTICAL ANALYSIS PLAN

PROTOCOL NUMBER: MMV_OZ439_13_003

A Randomised, Double-blind, Phase IIb Study to Investigate the Efficacy, Safety, Tolerability and Pharmacokinetics of a Single Dose Regimen of Artefenomel (OZ439) in Loose Combination with Piperaquine Phosphate in Adults and Children with Uncomplicated *Plasmodium falciparum* Malaria

AUTHOR: ILLZE CROUS

VERSION NUMBER AND DATE: VX.**X, DDMMMYYYY**

Copyright © 2012 Quintiles Transnational Corp. All rights reserved.
The contents of this document are confidential and proprietary to Quintiles Transnational Corp. Unauthorized use, disclosure or reproduction is strictly prohibited.

Statistical Analysis Plan Signature Page

Statistical Analysis Plan V2 (Dated 04DEC2015) for Protocol MMV_OZ439_13_003.

	Name	Signature	Date
Author:	Illze Crous	[signature]	7 Dec 2015
Position:	Statistical Team Lead		
Company:	Quintiles		

Upon review of this document, the undersigned approves this version of the Statistical Analysis Plan, authorising that the content is acceptable for the reporting of this study.

	Name	Signature	Date
Approved By:	Fiona Macintyre	F. Macintyre	7 Dec 2015
Position:	Project Director		
Company:	Medicines for Malaria Venture		
Approved By:	Helen Demarest	[signature]	07 DEC 2015.
Position:	Clinical Operations Lead		
Company:	Medicines for Malaria Venture		

Copyright © 2012 Quintiles Transnational Corp. All rights reserved.
The contents of this document are confidential and proprietary to Quintiles Transnational Corp. Unauthorized use, disclosure or reproduction is strictly prohibited.

MODIFICATION HISTORY

Unique Identifier for this Version	Date of the Document Version	Author	Significant Changes from Previous Authorised Version
1.0	20NOV2015	Illze Crous	Not Applicable. First Version.
2.0	04DEC2015	Illze Crous	Updated the Intent to Treat and the Per Protocol analysis set definitions based on decisions at the final blind data review meeting.<br><br>Added qPCR and RT-PCR parasitaemia analysis to the SAP.

Copyright © 2012 Quintiles Transnational Corp. All rights reserved.
The contents of this document are confidential and proprietary to Quintiles Transnational Corp. Unauthorized use, disclosure or reproduction is strictly prohibited.

TABLE OF CONTENTS

1. INTRODUCTION 8

2. STUDY OBJECTIVES 8

2.1. Primary Objective 8

2.2. Secondary Objectives 8

2.3. Exploratory Objectives 8

2.4. Pharmacokinetic Objectives 9

2.5. Safety Objective 9

2.6. Objectives/Endpoints to be Handled Outside of this SAP 9

3. STUDY DESIGN 10

3.1. General Description 10

3.2. Schedule of Events 11

3.3. Additional Clarification and/or Changes to Analysis from Protocol 11

4. PLANNED ANALYSES 13

4.1. Independent Safety Monitoring Board (ISMB) 13

4.2. Interim Analysis 14

4.3. Final Analysis 14

5. ANALYSIS SETS 15

5.1. All Patients Randomised Analysis Set 15

5.2. Safety Analysis set 15

5.3. Intent to Treat Analysis Set [ITT] 15

Copyright © 2012 Quintiles Transnational Corp. All rights reserved.
The contents of this document are confidential and proprietary to Quintiles Transnational Corp. Unauthorized use, disclosure or reproduction is strictly prohibited.

5.4. Per Protocol [PP] Analysis Set 16

5.5. Modified Per protocol [mPP] Analysis Set 16

5.6. Pharmacokinetic [PK] Analysis Set 17

6. GENERAL ANALYSIS DEFINITIONS AND STATISTICAL CONSIDERATIONS 18

6.1. Reference Start Date and Study Day 18

6.2. Baseline 18

6.3. Post-baseline 18

6.4. Retests, Unscheduled Visits and Premature Discontinuation Data 19

6.5. Study Period Definitions and Visits 19

6.6. Statistical Tests 21

6.7. Common Calculations 22

6.8. Software Version 22

7. STATISTICAL CONSIDERATIONS 23

7.1. Adjustments for Covariates and Factors to be Included in Analyses 23

7.2. Multicentre Studies 23

7.3. Missing data 23

7.4. Multiple Comparisons/Multiplicity 24

7.5. Examination of Subgroups 24

8. TABLE, LISTING AND FIGURE PRESENTATIONS 26

9. DISPOSITION AND WITHDRAWALS 27

9.1. Derivations 28

10. ANALYSIS SETS AND MAJOR PROTOCOL DEVIATIONS 28

Copyright © 2012 Quintiles Transnational Corp. All rights reserved.
The contents of this document are confidential and proprietary to Quintiles Transnational Corp. Unauthorized use, disclosure or reproduction is strictly prohibited.

10.1. Derivations 28

11. DEMOGRAPHIC AND OTHER BASELINE CHARACTERISTICS 29

11.1. Derivations 30

12. MEDICAL HISTORY 31

12.1. Derivations 31

13. CONCOMITANT MEDICATIONS 32

13.1. Derivations 32

14. STUDY DRUG ADMINISTRATION AND COMPLIANCE 33

14.1. Derivations 34

15. EFFICACY OUTCOMES 35

15.1. Objectives 35

15.2. Definitions 36

15.3. Crude and PCR-adjusted Outcome Derivation 38

15.4. Primary Efficacy Endpoint 40

15.5. Secondary Efficacy Endpoints 42

15.6. Exploratory Efficacy Endpoints 47

16. SAFETY OUTCOMES 50

16.1. Adverse Events 50

16.2. Laboratory Evaluations 54

16.3. ECG Evaluations 57

16.4. Vital Signs 60

16.5. Physical Examination 61

Copyright © 2012 Quintiles Transnational Corp. All rights reserved.
The contents of this document are confidential and proprietary to Quintiles Transnational Corp. Unauthorized use, disclosure or reproduction is strictly prohibited.

17. REFERENCES 62

APPENDIX 1.REQUIREMENTS AND SPECIFICATIONS FOR PROGRAMMING AND PRESENTATION OF TABLES, LISTINGS AND FIGURES (TLFS) 63

APPENDIX 2.STUDY PERIOD ALLOCATION AND DATE IMPUTATIONS 65

APPENDIX 3.QUANTITATIVE SAFETY LABORATORY ASSESSMENTS 66

APPENDIX 4.QUALITATIVE LABORATORY ASSESSMENTS: CATEGORISATION 67

Copyright © 2012 Quintiles Transnational Corp. All rights reserved.
The contents of this document are confidential and proprietary to Quintiles Transnational Corp. Unauthorized use, disclosure or reproduction is strictly prohibited.

1. INTRODUCTION

The statistical analysis plan (SAP) with reference to the most recent version of the tables, listings, and figure shells describes the rules and conventions to be used and the planned presentation in the analysis of efficacy and safety data for protocol MMV OZ439_13_003 as performed by Quintiles Biostatistics.

This SAP is based on the approved final protocol MMV_OZ439_13_003, dated 30JAN2014, including Amendment 1 dated 26MAY2014, Amendment 2 dated 27AUG2014 and Amendment 4 dated 17JUL2015. Amendment 3 dated 07JAN2015 was retracted. In addition, it is based on the electronic case report form (eCRF) Version 2, dated 24JUL2014 and the latest version of the blind data review (BDR) plan.

This SAP does not address the Pharmacokinetics (PK) or the PK/Pharmacodynamics (PD) analyses and reporting thereof, as these endpoints are to be performed and reported by BEL Pharm Consulting.

2. STUDY OBJECTIVES

2.1. PRIMARY OBJECTIVE

The primary objective is to determine whether a single dose combination of OZ439/PQP is an efficacious treatment for uncomplicated *P. falciparum* malaria in adults and children.

2.2. SECONDARY OBJECTIVES

The secondary objectives are:

- To determine the incidence of recrudescence and new infection.
- To determine the time to relief of fever and parasite clearance.

2.3. EXPLORATORY OBJECTIVES

The exploratory objectives are:

- To evaluate the proportion of patients with gametocytes at each assessment.
- To characterise gametocyte carriage.
- To examine the relationship between adequate clinical and parasitological response (ACPR) and exposure to OZ439/Piperaquine Phosphate (PQP) (using logistic regression).
- To determine parasite clearance kinetics.
- To examine the relationship between Kelch-13 genotype and additional parasite genotypes of interest that may be identified and parasite clearance kinetics/efficacy.

Refer to Section 2.6: Objectives/Endpoints to be Handled Outside of this , for the list of objectives and endpoints which are not addressed in this SAP and are reported outside of the clinical study report (CSR).

Copyright © 2012 Quintiles Transnational Corp. All rights reserved.
The contents of this document are confidential and proprietary to Quintiles Transnational Corp. Unauthorized use, disclosure or reproduction is strictly prohibited.

2.4. Pharmacokinetic Objectives

The pharmacokinetic objectives are:

- To determine c_{max}, t_{max} and area under the curve (AUC) of OZ439 and PQP in patients $\geq$35 kg.
- To characterise the pharmacokinetics and potential covariates in all patients (using population PK analysis).

2.5. Safety Objective

The safety objective is:

- To evaluate the safety and tolerability of OZ439/PQP

2.6. Objectives/Endpoints to be Handled Outside of this SAP

The following exploratory objectives and endpoints are to be analysed and included in the final PK/PD report as an appendix to the CSR:

  - The relationship between ACPR and exposure to OZ439/PQP (using logistic regression).
  - Correlation between response (ACPR at Day 28 and 42) and exposure (Day 7) to OZ439/PQP.
  - Exposure response evaluation including pharmacokinetic/pharmacodynamics modelling.

The following exploratory objectives and endpoints are to be analysed and reported outside of the clinical study report (CSR):

- To characterise gametocyte carriage.
  - Integrated number of gametocytes (AUC) at 28 and 42 days for:
    - Patients with gametocytes at Baseline.
    - Patients with no gametocytes at Baseline that develop gametocytes during the study.
- To examine the relationship between parasite genotypes of interest that may be identified (other than Kelch-13 genotype) and parasite clearance kinetics/efficacy.
  - Correlation between Kelch-13 genotype status and additional parasite genotypes of interest that may be identified, and efficacy.

Copyright © 2012 Quintiles Transnational Corp. All rights reserved.
The contents of this document are confidential and proprietary to Quintiles Transnational Corp. Unauthorized use, disclosure or reproduction is strictly prohibited.

3. STUDY DESIGN

3.1. GENERAL DESCRIPTION

This is a randomised, double-blind single-dose (loose combination) study in patients aged ≥ 6 months to < 70 years, with uncomplicated *Plasmodium (P.) falciparum* malaria.

Given the adaptive nature of the study design, the total number of patients recruited to this study is to be capped at 150 per treatment arm for the primary lower immunity population of interest plus an approximate additional 10% African patients > 5 years, giving an estimated total maximum number of patients of 165 per treatment arm.

- Three randomised OZ439/PQP treatment arms are to be evaluated for patients ≥35 kg, with weight-adjusted doses scaled to target similar exposures in patients <35 kg:
    - OZ439 800 mg: PQP 1440 mg.
    - OZ439 800 mg: PQP 960 mg.
    - OZ439 800 mg: PQP 640 mg.
- The study aims to recruit the following patient populations (refer to Section 6.5 Patient Population of the protocol for further details):
    - Lower immunity population:
        - This is the primary population of interest.
        - Patients with the highest probability of having lower immunity to *P. falciparum.*
        - Asian patients of all age groups.
        - African patient's ≤ 5 years.
    - Higher immunity population:
        - Patients with the probability of having higher immunity to *P. falciparum.*
        - African patients >5 years to <70 years.
- Safety in the older age range (Asian and African) is to be assessed before proceeding down to the younger age range (refer to Figure 1: Step-down Procedure in the protocol):
    - Thirty (30) patients (> 15 years) are to be assessed for safety before opening recruitment in the > 5 and ≤ 15 year age range.
    - Twenty (20) additional patients (> 5 to ≤ 15 years) are to be assessed for safety before opening recruitment in the > 2 and ≤ 5 year age range.
    - Twenty (20) additional patients (> 2 to ≤ 5 years) are to be assessed for safety before opening recruitment in the ≥ 6 month to ≤ 2 year age range.
- The study schedule comprises of:
    - Pre-Treatment Period (Screening/Pre-dose):
        - Patients admitted to the Clinical Unit for Screening, considered eligible and who provided informed consent and/or assent (as applicable) are to be recruited to the study.

Copyright © 2012 Quintiles Transnational Corp. All rights reserved.
The contents of this document are confidential and proprietary to Quintiles Transnational Corp. Unauthorized use, disclosure or reproduction is strictly prohibited.

- Treatment Period:
  - Following Screening and informed consent, patients are to receive the single administration of study drug and followed up for clinical signs of malaria (parasitaemia and temperature), safety assessments and PK to Day 42 following study drug administration (Day 63 at selected centres).
  - Following study drug administration, patients are to remain in the Clinical Unit for a minimum of 48 or 72 hours (depending on region, age, parasitaemia and temperature). Patients may remain in the Clinical Unit until Day 7 procedures are completed if more convenient.
  - The overall duration of the study (Screening to final assessment) ranges from 42 days to 63 days depending on whether recruited at a centre performing assessments to Day 42 or Day 63. The number of visits therefore range from approximately 9 to 12 depending on time of discharge and whether the patient returns to the Clinical Unit for assessments during intervening periods.
  - For safety analysis purposes only, scheduled assessments up to and including Day 28 are to be included in the summary tables. All assessments (scheduled and unscheduled) are to be included in the by-patient listings.
  - For efficacy analysis purposes only, assessments up to date of study completion at Day 42 or 63 (selected centres only), premature study discontinuation, including switch to established anti-malarial treatment or start of any other treatment with anti-malarial activity, whichever is earliest are to be included in the efficacy analyses. The efficacy analyses include parasitaemia, temperature and clinical signs and symptoms of uncomplicated malaria assessments.
- Follow-up Period:
  - For concomitant medication and safety analysis purposes only, assessments after Day 28 are to be presented in by-patient listings.

3.2. SCHEDULE OF EVENTS

Refer to Section 2: Schedule of Assessments, Screening to Day 63 of the protocol.

3.3. ADDITIONAL CLARIFICATION AND/OR CHANGES TO ANALYSIS FROM PROTOCOL

- Recruitment was stopped per protocol, following the results of the first interim analysis that was made available to the unblinded MMV team by the Quintiles Biostatistics unblinded team.
- In Section 3.6: Study Population Rationale of the protocol it is noted that the study is to recruit across geographical regions (Africa, Asia and possibly Latin America) and across a wide age range. No patients for Latin America and or patients younger than or equal to (≤) 5 years in Asia were however recruited.
- In Section 5.2: Study Plan of the protocol the visit windows for Day 28, Day 42 and Day 63 are ± 2 days. The visit windows agreed for the analysis are: -2/+3 days for the Day 14, Day 21, Day 28 endpoints and -3/+5 for the Day 42 and Day 63 endpoints in relation to the derivation of the crude and PCR-adjusted ACPR analysis. Date of agreement of change 04NOV2015.

Copyright © 2012 Quintiles Transnational Corp. All rights reserved.
The contents of this document are confidential and proprietary to Quintiles Transnational Corp. Unauthorized use, disclosure or reproduction is strictly prohibited.

- In Section 11.2: Analysis Sets of the protocol, an All Patients Enrolled analysis set is defined. The All Patients Enrolled analysis set is to be removed given that only randomised patient's data are captured in the database. Additionally a subset of the Intent to Treat analysis set and a modified Per Protocol (mPP) analysis set are defined in the SAP for analysis purposes. Date of agreement of change 22OCT2015.
- At the time of the final blind data review meeting the criteria defining the ITT analysis set was revised from Section 11.2: Analysis Sets of the protocol to include patients with no post-baseline efficacy data. The PP analysis set definition was revised to exclude the patients with no post-baseline efficacy data. Date of agreement of change 03DEC2015.
- In Section 11.8.2.1: Efficacy Analysis, it is noted that microscopic and qPCR determined parasitaemia are to be used for the calculation of parasite reduction ratio (PRR). PRR calculated from qPCR is an exploratory endpoint. The clearance rate constant, PC half-life and time to 50 %, 90 % and 99 % reduction of parasitaemia are to be estimated per patient, utilising the world wide antimalarial resistance network (WWARN) parasite clearance estimator (PCE) based on the linear part of the individual natural log parasitaemia-time profiles. The PRR24 and PRR48 results are to be calculated from the clearance rate constant (1/hours) obtained from the WWARN PCE. Date of agreement of change is 06NOV2015 for utilising the WWARN calculator.

- In Section 11.8.2.1: Efficacy Analysis of the protocol, it is noted that the primary efficacy endpoint, PCR-adjusted ACPR at Day 28, is to be repeated for endpoints Day 42 and Day 63, and Crude ACPR endpoints Day 28, Day 42 and Day 63. Following comments received and the SAP discussion meeting, two additional endpoints, i.e. Day 14 and Day 21 are to be added for the crude and PCR-adjusted endpoint analysis. Date of agreement of change 22OCT2015.
- The following terminology updates were confirmed on 05NOV2015:
    - PCR-crude ACPR to Crude ACPR.
    - Re-infection to New infection.
    - Parasite reduction rate to Parasite reduction ratio.
- Following the SAP discussion meeting dated 05NOV2015, a Treatment Period for safety assessments up to and including Day 28 (based on nominal visit) and Follow-up Period for safety assessments recorded after Day 28 are to be derived. For the Safety table presentations only the analysable assessments recorded during the Treatment Period are to be included., however all data recorded are to be presented in by-patient listings
- Invalid haemoglobin results obtained at Centre 252: For the period of 05JUN2015 to 27AUG2015, the print out result from the study machine was noted with "INVALID RESULT, DO NOT REPORT". MMV confirmed on 02NOV2015 that these invalid results are to be excluded from the haemoglobin analysis presented in the relevant summary tables. All assessments are however to be included in the by-patient listings.

Copyright © 2012 Quintiles Transnational Corp. All rights reserved.
The contents of this document are confidential and proprietary to Quintiles Transnational Corp. Unauthorized use, disclosure or reproduction is strictly prohibited.

4. Planned Analyses

The following analyses are to be performed for this study:

- Four unblinded Independent Safety Monitoring Board (ISMB) safety analyses.
- Unblinded interim analysis.
- Final analysis.

4.1. Independent Safety Monitoring Board (ISMB)

Four unblinded ISMB safety reviews were performed, on each of the four age groups on all data available, for the minimum specified patients reaching up to Day 14 for this study in order to ensure patients safety. For the unblinded ISMB analyses, the Quintiles Biostatistics blinded team produced all required programs and blinded outputs. These programs and outputs were transferred to the Quintiles Biostatistics unblinded team. The Quintiles Biostatistics unblinded team then produced all outputs based on the actual randomisation schedule and using the same programs from the Quintiles Biostatistics blinded team. Quintiles Biostatistics unblinded team was responsible for providing the unblinded outputs to the ISMB members as specified in the latest version of the Biostatistics Blinded to Unblinded Team handover document (CS_TP_BS0214) for the MMV_OZ439_13-003 study. Refer to the latest version of the unblinding plan for details with regards to the handling of the unblinded information.

A set of ISMB safety review tables, listings and figure (TLF) shells was created specifically for the ISMB analyses. Refer to the ISMB charter (Version 1.2 dated 20MAY2015) for additional information regarding the ISMB analyses.

Copyright © 2012 Quintiles Transnational Corp. All rights reserved.
The contents of this document are confidential and proprietary to Quintiles Transnational Corp. Unauthorized use, disclosure or reproduction is strictly prohibited.

4.2. Interim Analysis

One interim efficacy analysis was performed on the PCR-adjusted ACPR at Day 28 for this study. Refer to separate interim SAP dated 04AUG2015.

An unblinded interim analysis was conducted to test for efficacy/futility at Day 28, using Bayesian methodology (refer to Figure 1: Step-down Procedure, Sections 8.3: Efficacy/Futility Interim Analysis and 11.8.1: Primary Efficacy Analysis of the protocol), following MMV's authorisation of the interim SAP and interim blind data review report including patient assignment to the relevant analysis sets. Only data from patients in the lower immunity population, i.e., patients in Asia of all age groups, and patients ≤ 5 years of age in Africa were included in the interim analysis (refer to Sections 6.5: Patient Population and 8.3: Efficacy/Futility Interim Analysis of the protocol). The same process was followed by the Quintiles Biostatistics blinded and unblinded teams as described for the ISMB analyses.

The unblinded Quintiles Biostatistician then provided the unblinded MMV recipient with the required summary tables as specified in the latest version of the Biostatistics Blinded to Unblinded Team handover document and unblinding plan for the OZ439_13_003 study.

A decision to stop recruitment for all three treatment arms was made by the unblinded MMV team following review of the interim analysis results. Recruitment was officially stopped on 04SEP2015 and it was agreed that all the patients already recruited in the study were to complete up to Day 42 (Day 63 selected centres only).

4.3. Final Analysis

All final, planned analyses identified in this SAP are to be performed by Quintiles Biostatistics following MMV authorisation of this SAP, relevant set of the TLF shells, the final analysis set assignments, final database lock and routine study unblinding.

The final analysis is to be performed on a clean database:

- All outstanding data issues and queries resolved.
- All unresolvable data issues documented in the data handling report (DHR) from Data Management.
- All coding of medications and adverse events (AEs) completed.
- Serious adverse events (SAE) reconciliation completed.
- All reconciliation of vendor data with the eCRF data completed successfully.

Copyright © 2012 Quintiles Transnational Corp. All rights reserved.
The contents of this document are confidential and proprietary to Quintiles Transnational Corp. Unauthorized use, disclosure or reproduction is strictly prohibited.

5. Analysis Sets

The five analysis sets, All Patients Randomised analysis set, Safety analysis set, Intent to treat (ITT), Per protocol (PP) and modified PP (mPP) analysis set are described in detail in the latest version of the BDR plan, including the criteria to be used in defining each of these analysis sets.

Prior to the locking of the database, agreement of patients' assignment to each of the analysis sets (excluding the PK analysis set which is specified in a separate PK/PD report) and identification of major protocol deviations are to be determined in collaboration with MMV. The authorisation of analysis sets is required prior to the final locking of the database and routine study unblinding.

The analysis sets are defined for the study below.

5.1. All Patients Randomised Analysis Set

The All Patients Randomised analysis set is to include all patients who provide written informed consent and/or assent (as applicable) prior to performing any specific study-related procedures, and who are randomised within the Interactive Web Response System (IWRS) at pre-dose to one of the three OZ439/PQP treatment arms.

For the purpose of statistical analyses and presentations based on the All Patients Randomised analysis set, patients are to be analysed according to the randomised treatment, regardless of actual treatment received.

Note that only randomised patients are entered into the clinical study database and hence available for review.

5.2. Safety Analysis set

The Safety analysis set defined below is in accordance with International Conference on Harmonization (ICH)-E9 (Statistical Principles for Clinical Trials). According to ICH as well as the protocol, the Safety analysis set is to be analysed according to the actual treatment received.

The Safety analysis set is to include all patients considered valid for the All Patients Randomised analysis set, who received the single dose combination of OZ439/PQP study drug (or part thereof).

The Safety analysis set is regarded as primary for the safety analyses.

5.3. Intent to Treat Analysis Set [ITT]

The ITT analysis set defined below is in accordance with ICH-E9. According to ICH, the ITT analysis set is to be analysed according to the randomised treatment, regardless of actual treatment received.

The Intent to Treat (ITT) analysis set is to include all patients considered valid for the Safety analysis set, who have a Baseline (confirmed positive blood film for *P. falciparum* asexual parasitaemia at inclusion).

The intent to treat principle is preserved, despite the exclusion of patients randomised who did not take the study drug, because the decision of whether or not to begin the treatment could not be influenced by knowledge of the randomised treatment arm as the study is blinded.

Copyright © 2012 Quintiles Transnational Corp. All rights reserved.
The contents of this document are confidential and proprietary to Quintiles Transnational Corp. Unauthorized use, disclosure or reproduction is strictly prohibited.

5.3.1. Intent to Treat Analysis Set: Subset

The subset of the Intent to Treat (ITT) analysis set is to include all patients in the ITT analysis set with the exception of those patients not administered the full dose of study drug (initial and/or re-dose administration).

5.4. Per Protocol [PP] Analysis Set

The PP analysis set is to include all patients considered valid for the ITT analysis set with no identified major protocol deviations in view of the valid course criteria defined below and as reviewed and confirmed by MMV prior to analysis.

Major protocol deviations are defined as any factor affecting the efficacy outcome or the treatment of the patient.

The PP analysis set is further defined by the following valid course criteria (ICH-E9):

- Essential efficacy data: At least one post-baseline parasitaemia assessment.
- Sufficient evidence of the study indication that is, acute, uncomplicated *P. falciparum* malaria.
- Overall treatment compliance ,i.e. patients who did not consume the full dose of study without talking vomiting into-consideration
- Adherence to the visit schedule.
- Eligible in accordance with the clinical study protocol's specified inclusion and exclusion criteria. Specifically criteria which could affect the efficacy outcome or the treatment of the patient:
    - Use of prohibited concomitant medications with antimalarial activity.
    - Presence of prohibited medical conditions that may affect the efficacy outcome or treatment of the patient.
    - Signs/symptoms of severe/complicated malaria.
    - Mixed *Plasmodium* infection before study drug administration.

For the purpose of statistical analysis based on this analysis set, patients are to be analysed in accordance with actual treatment received.

5.5. Modified Per protocol [mPP] Analysis Set

The modified Per Protocol (mPP) analysis set is to include all patients in the PP analysis set who did not vomit between (>) 5 minutes and (≤) 4 hours after study drug administration, i.e. patients who vomited and were not re-dosed successfully are excluded.

All patients considered valid for the PP analysis set and in addition:

- Did not vomit between (>) 5 minutes and (≤) 4 hours following the initial administration of study drug, or
- Successfully re-dosed following vomiting within (≤) 5 minutes of initial administration of study drug are to be included in the modified PP analysis set.

Copyright © 2012 Quintiles Transnational Corp. All rights reserved.
The contents of this document are confidential and proprietary to Quintiles Transnational Corp. Unauthorized use, disclosure or reproduction is strictly prohibited.

5.6. PHARMACOKINETIC [PK] ANALYSIS SET

The PK analyses are to be performed by BEL Pharm Consulting, hence the Quintiles Biostatistician is to provide Appendix 1 of the BDR report containing the patients' assignment to the aforementioned analysis sets to BEL Pharm Consulting.

Copyright © 2012 Quintiles Transnational Corp. All rights reserved.
The contents of this document are confidential and proprietary to Quintiles Transnational Corp. Unauthorized use, disclosure or reproduction is strictly prohibited.

6. General Analysis Definitions and Statistical Considerations

6.1. Reference Start Date and Study Day

Study day is to be calculated from the reference start date, and is used to show start/stop day of assessments and events.

Reference start date is defined as the date of the of study drug administration and is referred to as Day 0. Study day is to appear in every listing where an assessment date or event date appears. Patients randomised who discontinue study participation prior to receiving study drug administration, have no reference start date and subsequently no study day is calculated.

In all calculations start date of study drug administration is the date of the initial dose, regardless of subsequent re-dosing due to vomiting.

Study day is to be calculated as follows:

- If the date of the assessment/event is prior to/on or after the reference start date then:

  Study day = (date of assessment/event – reference date)

In the situation where the event date is partial or missing, the date is to appear partial or missing in the listings, and study day, and any corresponding durations are to be presented based on the imputations specified in Appendix 2; Partial Date Conventions.

6.2. Baseline

Unless otherwise specified, Baseline is defined as the last non-missing assessment (scheduled or unscheduled) obtained before study drug administration. The only exceptions are temperature, electrolytes (sodium, potassium) and glucose where the earliest Screening assessment is to be regarded as the Baseline value and blood film parasitaemia assessments where Baseline is defined as the second thick blood film results obtained at Screening before study drug administration.

In the case where the last non-missing assessment and the reference start date coincide, the assessment is to be considered pre-baseline unless already designated a specific scheduled timepoint (i.e., 4 hours post-dose). Thus, if laboratory or electrocardiogram assessments fall on the date of study drug administration and the time of the assessment is missing, the applicable assessment is to be considered as Baseline. However, AEs starting on the reference start date (date of study drug administration) are to be considered as treatment-emergent; therefore post-baseline.

6.3. Post-baseline

Unless otherwise specified, post-baseline is defined as any assessment (scheduled or unscheduled) obtained after the administration of study drug.

Copyright © 2012 Quintiles Transnational Corp. All rights reserved.
The contents of this document are confidential and proprietary to Quintiles Transnational Corp. Unauthorized use, disclosure or reproduction is strictly prohibited.

6.4. Retests, Unscheduled Visits and Premature Discontinuation Data

In general, for by-visit summaries, data recorded at the scheduled visit are to be presented. Unscheduled assessments are not to be included in by-visit summaries, but may contribute to the Baseline value.

In the case of a retest (same visit number assigned), the last available test result as provided in the data transfer for that visit/scheduled timepoint is to be used for by-visit summaries.

Listings are to include scheduled, unscheduled and premature discontinuation data. No mapping of premature discontinuation data is to be performed. If a patient discontinues at a scheduled visit, the visit label is to reflect the scheduled visit name as obtained from the eCRFs. If a patient discontinues at an unscheduled visit, the visit label as is to be and presented as ‘Unscheduled’.

6.5. Study Period Definitions and Visits

Study periods for the efficacy analysis are to be defined as follows and identified in the tables, listings and figures in accordance with the proposed period label:

Study Period Label	Start Date and Time	End Date and Time
Pre-treatment Period	Date and time of signing the informed consent.	Start of study drug administration (OZ439/PQP).
Treatment Period	Start date and time of study drug administration (OZ439/PQP).	Last date of assessment (scheduled or unscheduled) up to and including date of study completion, premature study discontinuation, including switch to established anti-malarial treatment or start of any other treatment with anti-malarial activity as captured on the Prior and Concomitant Medications eCRF, whichever is earliest.

Completed: Defined as patients completing the study up to Day 42 or Day 63 (selected centres only) without the need for intervention per protocol.

Premature study discontinuation: Discontinued after being randomised to one of three treatment arms, including switch to established anti-malarial treatment subsequent to meeting the protocol-specified criteria (refer to Section 5.8: Circumstances for Established Anti-malarial Treatment of the protocol).

In view of the safety analyses the following periods are defined as ‘worst case’ approach to the assignment of treatment-emergent events and assessments:

Study Period Label	Start Date and Time	End Date and Time
Pre-treatment Period	Date and time of signing the informed consent.	Start of study drug administration (OZ439/PQP).
Treatment Period	Start date and time of study drug administration (OZ439/PQP).	Up to and including Day 28 (based on nominal visit).
Follow-up Period	Started after Day 28 (based on nominal visit).	End of Study, Day 42 or Day 63 (selected centres only).

Copyright © 2012 Quintiles Transnational Corp. All rights reserved.
The contents of this document are confidential and proprietary to Quintiles Transnational Corp. Unauthorized use, disclosure or reproduction is strictly prohibited.

The logical ordering of the periods and visits are as follows:

- Pre-treatment Period:
  - Screening.
  - Randomisation.
  - Pre-dose.
- Treatment Period:
  - Day 0/0 hours (start of study drug administration)
  - Day 0/1 hours.
  - Day 0/2 hours.
  - Day 0/6 hours
  - Day 0/12 hours.
  - Day 0/18 hours.
  - Day 0/24 hours.
  - Day 0/30 hours.
  - Day 0/36 hours.
  - Day 0/48 hours.
  - Day 0/72 hours.
  - Day 5.
  - Day 7.
  - Day 10.
  - Day 14.
  - Day 15 to 20.
  - Day 21.
  - Day 22 to 27.
  - Day 28.
  - Day 42.
  - Day 63 (selected centres only).

Visits not related to a specific scheduled timepoint:

- Baseline.
- Unscheduled.

Copyright © 2012 Quintiles Transnational Corp. All rights reserved.
The contents of this document are confidential and proprietary to Quintiles Transnational Corp. Unauthorized use, disclosure or reproduction is strictly prohibited.

6.6. STATISTICAL TESTS

Unless otherwise specified, the default summary statistics are as follows:

- Quantitative variables:
  - The number of patients (n) with missing results for quantitative variables are to be presented as part of a 'Missing' statistic where applicable. If there are no missing values, do not present the row indicating number of missing assessments.
  - Number of patients with assessments available (n).
  - Mean.
  - Standard deviation (SD).
  - Median.
  - Minimum.
  - Maximum.
- Qualitative variables:
  - Number of patients in each category (n).
  - The percentage of patients in each category (%) can be presented relative to either one of the following:
    - The total number of patients in the relevant analysis set.
    - The total number of patients in the relevant analysis set, with assessments available (observed cases).
    - In the event of missing assessments, a 'Missing' category showing the number of patients with missing assessments at each level of summarisation is to be presented.

The total number of patients (N) per treatment arm and in total across treatment arms per relevant analysis set is to be presented in each table.

- The default significance level is 5%; confidence intervals are 95% and all tests two-sided, unless otherwise specified in the description of the analyses.

Copyright © 2012 Quintiles Transnational Corp. All rights reserved.
The contents of this document are confidential and proprietary to Quintiles Transnational Corp. Unauthorized use, disclosure or reproduction is strictly prohibited.

6.7. COMMON CALCULATIONS

6.7.1. CHANGE FROM BASELINE

For quantitative assessments, change from Baseline is to be calculated as:

- Change from Baseline at visit X = (Result at visit x – Baseline result)

If a result/value is missing, change from Baseline result is to be presented as missing in the listing; hence no imputation for a missing change from Baseline is to be performed.

6.7.2. ADJUSTED BODY TEMPERATURE AND FEVER

If an alternative route of temperature measurement, i.e. oral, tympanic or rectal is recorded, the temperature is adjusted to a standard axillary route by subtracting 0.5 °C from the temperature recorded based on oral, tympanic or rectal measurement.

Adjusted body temperature needs to be presented in degrees Celsius (°C) and to an accuracy of one decimal place.

Fever (Yes/No) is to be derived based on the adjusted axillary body temperature as:

- 'Yes', if adjusted body temperature ≥ 37.5 ^{o}C.
- 'No', if adjusted body temperature < 37.5 ^{o}C.

6.8. SOFTWARE VERSION

All analyses described in this SAP are to be conducted using SAS® Version 9.4.

Copyright © 2012 Quintiles Transnational Corp. All rights reserved.
The contents of this document are confidential and proprietary to Quintiles Transnational Corp. Unauthorized use, disclosure or reproduction is strictly prohibited.

7. STATISTICAL CONSIDERATIONS

7.1. ADJUSTMENTS FOR COVARIATES AND FACTORS TO BE INCLUDED IN ANALYSES

Not applicable.

7.2. MULTICENTRE STUDIES

This study is to be conducted by multiple Investigators at multiple centres in Asia and Africa. Randomisation to treatment arm is not stratified by centre. No adjustment for centre effect is to be made. The following centres are included in this study:

Table 1: Study Centres

Region	Country	Centre Number	Investigator
Africa	Burkina Faso	111	Dr Halidou Tinto
Africa	Burkina Faso	112	Dr Alfred Tiono
Africa	Burkina Faso	113	Dr Alfred Tiono
Africa	Benin	171	Dr Saadou Issifou
Africa	Democratic Republic of Congo (DRC)	181	Prof Antoinette K. Tshefu
Africa	Gabon	121	Prof Michael Ramharter
Africa	Gabon	122	Prof Marielle Bouyou-Akotet
Africa	Mozambique	131	Prof Quique Basset
Africa	Uganda	141	Dr Adoke Yeka
Asia	Vietnam	251	Dr Tran Thanh Duong
Asia	Vietnam	252	Dr Tran Thanh Duong
Asia	Vietnam	253	Dr Tran Thanh Duong
Asia	Vietnam	254	Dr Tran Thanh Duong

All centres are to be pooled for analysis purposes.

7.3. MISSING DATA

For the handling of missing data see the relevant domain sections within this SAP. For the handling of partial AE dates refer to Appendix 2: Study Phase Allocation and Date Imputations, of this SAP.

The presentation of missing statistics/categories in descriptive summaries are to be presented as described in Section 6.6:

Copyright © 2012 Quintiles Transnational Corp. All rights reserved.
The contents of this document are confidential and proprietary to Quintiles Transnational Corp. Unauthorized use, disclosure or reproduction is strictly prohibited.

Statistical Tests.

7.4. Multiple Comparisons/Multiplicity

Not applicable.

7.5. Examination of Subgroups

Most of the analyses are to be performed by region/age group, however for the study drug compliance and ACPR table outputs, presentation by weight group and region/centre are also to be performed. The presentations for ECG are to be performed by age group.

The following subgroups are defined from data included in the randomisation file as assessed at Screening and obtained from Cenduit Interactive web response system (IWRS), in view of producing analyses per subgroup as required:

- Region:
  - Asia.
  - Africa.
- Age group:
  - > 5 years.
  - ≤ 5.0 years.
    - > 2.0 to ≤ 5.0 years.
    - ≥ 0.5 to ≤ 2.0 years.
- Weight group:
  - ≥ 35 kg.
  - ≥ 24 to < 35 kg.
  - ≥ 15 to < 24 kg.
  - ≥ 10 to < 15 kg
  - ≥ 5 to < 10 kg.
- Asia centre:
  - 251.
  - 252.
  - 253.
  - 254.

Copyright © 2012 Quintiles Transnational Corp. All rights reserved.
The contents of this document are confidential and proprietary to Quintiles Transnational Corp. Unauthorized use, disclosure or reproduction is strictly prohibited.

		Subgroup					
**Domain/Variable**	**Analysis Set**	**All**	**Region**	**Age:** > 5.0 years ≤ 5.0 years	**Age subgroup** > 2.0 to ≤ 5.0 years ≥ 0.5 to ≤ 2.0 years	**Weight**	**Centre**
Crude and ACPR-adjusted	ITT, PP and mPP	Y	Y	Y	Y	Y	N
ETF, LCF and LPF	ITT, PP and mPP	Y	Y	Y	Y	Y	N
Fever Clearance Time	ITT and PP	Y	Y	Y	N	N	N
Kaplan-Meier for re-emergence, recrudescence and new infection	ITT: Subset	Y	Y	Y	N	N	N
PCT, PRR48, PRR24, PC half-life	PP	Y	Y	Y	N	N	Y (Asia only)
Kelch-13 genotype	PP	Y	Y	N	N	N	Y (Asia only)
Relationship between PC half-life) and Kelch-13 genotype	PP	Y	Y	N	N	N	Y (Asia only)
Asexual plots	PP	Y	Y	Y	N	N	N
Qualitative Parasitaemia Assessments (Absent/present)	ITT and PP	Y	Y	Y	N	N	N
Study drug Compliance	Safety	Y	Y	Y	N	Y	Y
Adverse events	Safety analysis set	Y Overview table summary only)	Y (Overview table summary only)	Y(Overview table summary only)	N	N	N
Laboratory	Safety	Y (Markedly abnormal criteria table and figures)	Y (Markedly abnormal criteria table and figures)	Y (Markedly abnormal criteria table and figures)	N	N	N
ECG	Safety	Y	N	Y	N	N	N
Vital Signs	Safety	Y	N	N	N	N	N

ACPR: Adequate clinical and parasitological response. ECG: Electrocardiogram. ETF: Early treatment failure. LCF: Late clinical failure. LPF: Late parasitological failure. ITT: Intent to Treat. PC half-life: Parasite clearance half-life. PCT: Parasite clearance time. PP: Per Protocol. PRR: Parasite reduction ratio. mPP: Modified Per Protocol.

Copyright © 2012 Quintiles Transnational Corp. All rights reserved.
The contents of this document are confidential and proprietary to Quintiles Transnational Corp. Unauthorized use, disclosure or reproduction is strictly prohibited.

8. TABLE, LISTING AND FIGURE PRESENTATIONS

APPENDIX 1 Requirements and Specifications for Programming and Presentation of Tables, Listings and Figures (TLFs) describe the conventions for presentation of data in outputs.

The TLF shells document provided together with this SAP describes the presentation of data for the final analysis of this study and therefore the format and content of the summary TLFs to be provided by Quintiles Biostatistics.

Note that verbatim terms, specifications (e.g., the reason a specific assessment was not performed) and all variables in the TLF shells that contain the suffix (eCRF) contain verbatim text that may include spelling mistakes. Verbatim text is to be presented in the listings 'as is' and no manual 'hard-coding' corrections of such data are to be made.

Copyright © 2012 Quintiles Transnational Corp. All rights reserved.
The contents of this document are confidential and proprietary to Quintiles Transnational Corp. Unauthorized use, disclosure or reproduction is strictly prohibited.

9. Disposition and Withdrawals

All patients who provided informed consent, and were randomised to a treatment arm are to be accounted for in this study until study completion or premature study discontinuation.

Patient disposition (completion/premature study discontinuation) and major protocol deviations (as defined in Section 6.3.1.9: Summary of Criteria of the latest BDR plan), including inclusion and exclusion criteria exceptions are to be presented for the All Patients Randomised analysis set.

- The disposition milestones presented in the by-patient data listing include:
  - Informed consent and assent, date and time of consent as obtained from the Informed Consent eCRF.
  - Screening, date and time as obtained from the Date of Visit eCRF.
  - Date and time of randomisation, randomisation number as obtained from the Randomisation eCRF.
  - Date and time of study drug administration as obtained from the Dosing eCRF.
  - Inclusion/Exclusion criteria exceptions as obtained from the Inclusion/Exclusion Criteria eCRF.
  - Completion/Premature study discontinuation including switch to an established anti-malarial treatment, date, time and primary reason, including the specification as obtained from the Established Anti-malarial Treatment/Premature Discontinuation/End of Study eCRF.
- The primary reason for premature study discontinuation is as follows:
  - Met criteria for established anti-malarial treatment, including the main timepoint and associated reason:
    - At 24 hours post-dose: Reason.
      - Baseline parasitaemia < 40 000 /µL: Increase 5 X Baseline or parasites > 60 000 /µL.
      - Baseline parasitaemia > 40 000 /µL < 70 000 /µL: Increase 2 X Baseline or parasites > 100 000 /µL.
      - Baseline parasitaemia > 70 000 /µL: Increase 1.5 X Baseline or parasites > 120 000 /µL.
    - At 48 hours: parasites > Baseline parasitaemia.
    - At 72 hours: parasites > 25% of Baseline or any detectable parasites with fever.
    - At 96 hours: failure to achieve parasite clear.
    - Any time: danger signs of severe malaria with parasitaemia.
    - Any time up to Day 42/Day 63: recrudescence or new infection.
  - Study drug discontinued (Dosing of study drug incomplete).
  - Withdrawal of consent.
  - In the Investigators' opinion, it is not in the best medical interest of the patient to continue the study.
  - At the discretion of the Investigator or if the patient is not sufficiently co-operative/non-compliant.
  - Adverse event.
  - Lost to follow-up.
  - Administrative reasons.
  - Other.
- Descriptive statistics are to be presented for the following by region/age group:
  - Patient randomisation: Number of patients randomised by region, country, centre and Investigator.
  - Number of patients randomised, treated, completed, prematurely discontinued study participation including the primary reason for premature study discontinuation as obtained from the End of Study eCRF.

Copyright © 2012 Quintiles Transnational Corp. All rights reserved.
The contents of this document are confidential and proprietary to Quintiles Transnational Corp. Unauthorized use, disclosure or reproduction is strictly prohibited.

9.1. Derivations

Based on the above assessments and with reference to Section 6: General Analysis Definitions and Statistical Considerations and Section 7: Statistical Considerations, the following variable is to be derived:

- Study day:

  Calculated relative to start of study drug administration, Day 0.

10. Analysis Sets and Major Protocol Deviations

- Major protocol deviations resulting in the exclusion of patients from one or more of the analysis sets, as identified during the conduct of the study in accordance with the most recent version of the BDR plan, as reviewed and finalised by MMV prior to planned analysis at the final blind data review meeting are to be summarised as follows:
  - Number of patients in each analysis set with reason(s) for exclusion from the relevant analysis set. Patients may be counted in more than one major protocol deviation category (reason for exclusion). Percentages are calculated relative to the total of number of randomised patients as entered in the clinical study database.

The following data are to be presented in by-patient listings:

  - Analysis set assignment.
  - Major protocol deviations. All patients with at least one major protocol deviation as finalised and authorised in collaboration with MMV are to be presented.
  - Inclusion and exclusion criteria exceptions and eligibility verification. Only patients who do not fulfil at least one of the criteria, or patients with a missing response to any of the criteria are to be presented.

10.1. Derivations

Not applicable.

Copyright © 2012 Quintiles Transnational Corp. All rights reserved.
The contents of this document are confidential and proprietary to Quintiles Transnational Corp. Unauthorized use, disclosure or reproduction is strictly prohibited.

11. DEMOGRAPHIC AND OTHER BASELINE CHARACTERISTICS

The following demographic and patient characteristic variables, as presented in the planned TLF shells, are to be recorded at Screening:

- Date of birth, age, gender, female of childbearing potential, race, history of drug abuse, alcohol, malaria incidents, date of first diagnosis of current malaria infection and patient history of fever in the last 24 hours as obtained from the Demography Details eCRF.
- Height, weight and BMI as obtained from the Vital Signs eCRF. The weight and BMI are obtained from the Cenduit system and integrated into the eCRF.
- Body temperature as obtained from Temperature eCRF (refer to Section 6.7.2 Adjusted Body Temperature and Fever).
- Confirmation of *P. falciparum* Mono-infection, rapid stain total parasitaemia, asexual count, gametocytes (absent or present) and counts as obtained from the Thick & Thin Malaria Blood Films eCRF.
- Discharge date and time, including response to ‘Was the patient parasitaemia and fever free and was this state maintained for at least 24 hours prior to discharge’, as obtained from the Hospital/Clinical Care Facility eCRF.

Demographic data and other patient characteristics are to be summarised for the Safety, PP and mPP analysis sets.

Descriptive statistics are to be presented for the following variables:

- Demographic and other patient characteristics at Screening are to be presented by region.
  - Age at randomisation (years).
  - Age group (years) as assigned by IWRS.
  - Gender.
  - Female of childbearing potential.
  - Race.
  - Height (cm) at Screening.
  - Weight (kg) at Screening.
  - Weight group (kg) as assigned by IWRS.
  - BMI (kg/m^2), as derived by data management (DM).
  - History of drug abuse.
  - Alcohol intake restricted for 3 days.
- Disease characteristics of *P. falciparum* malaria are to be presented by region/age group and weight group:
  - Malaria history:
    - Malaria incidents in the last 12 months.
    - Time since diagnosis of current malaria infection (days).
    - History of fever in the last 24 hours.
    - Screening adjusted body temperature (˚C) (refer to Section 6.7.2 Adjusted Body Temperature and Fever).
  - Derived fever.

Copyright © 2012 Quintiles Transnational Corp. All rights reserved.
The contents of this document are confidential and proprietary to Quintiles Transnational Corp. Unauthorized use, disclosure or reproduction is strictly prohibited.

- Screening malaria blood films:
    - Thin film *P. falciparum* Mono-infection.
    - Screening total parasitaemia (thick film rapid stain) (/µL).
    - Thick film:
        - Asexual parasites (Baseline value) (/µL).
        - Gametocytes (/µL).
- Status at discharge:
    - Parasitaemia and fever free.

The following data are to be presented in by-patient listings for the All Patients Randomised analysis set.

- Demographics and other patient characteristics at Screening.
- Disease characteristics of *Plasmodium falciparum* malaria, including malaria history, Screening malaria blood films and status at discharge.

11.1. DERIVATIONS

Based on the above assessments and with reference to Section 6: General Analysis Definitions and Statistical Considerations and Section 7: Statistical Considerations, the following variables are to be derived:

- Study day:
    - Calculated relative to start of study drug administration, Day 0.
- Age at randomisation (years).
    - Age is to be calculated relative to the date of randomisation using the following SAS® code:

      Age (Years) = int((intck('month ', <date of birth>, <date of randomisation>) - (day(<date of randomisation>) < day(<date of birth>))) / 12)

  Reason for age derivation: To present the data in the same unit for summary purposes.
- Time since diagnosis of current malaria infection (days) = (Date of Screening – Date of first diagnosis of current malaria infection).

  If relevant, partial diagnosis of current malaria infection start dates are to be imputed:

    - An unknown day only is to be imputed as the 15th of the collected month and year.
    - An unknown day and month is to be imputed as 30JUN of the collected year.
- Baseline parasitaemia for asexual parasites and gametocytes (refer to Section 6.2 Baseline).
- Adjusted axillary body temperature (°C) and fever (Yes/No) (refer to Section 6.7.2: Adjusted Body Temperature and Fever).
- Derived fever:

  Derived fever = 'Yes' if history of fever in the last 24 hours indicated as 'Yes', Screening adjusted body temperature ≥ 37.5 °C or fever indicated on the Clinical Signs/Symptoms of Uncomplicated Malaria eCRF as 'Present'.

Copyright © 2012 Quintiles Transnational Corp. All rights reserved.
The contents of this document are confidential and proprietary to Quintiles Transnational Corp. Unauthorized use, disclosure or reproduction is strictly prohibited.

12. MEDICAL HISTORY

Relevant medical history conditions are to be recorded on the Medical History eCRF.

Note that clinical signs and symptoms of current uncomplicated malaria are to be obtained from the Malaria eCRF and not the Medical History eCRF.

All medical history conditions are coded using MedDRA Version 17.0.

No partial date imputations for medical history start/stop dates are to be performed and subsequently no study day calculated. If no stop date is reported it is assumed that the medical history condition is ongoing.

Medical history conditions are to be listed for the All Patients Randomised analysis set and summarised by Medical Dictionary for Regulatory Activities (MedDRA) System Organ Class (SOC) and Preferred Term (PT) for the Safety analysis set as.

- The number (n) and percentage (%) of patients with medical history conditions by treatment arm. If a medical history condition occurs more than once for a patient the patient is only counted once per MedDRA SOC or PT. The aforementioned excludes the current malaria infection.
- A separate by-patient data listing is to be produced for medical history conditions.

12.1. DERIVATIONS

Based on the above assessments and with reference to Section 6: General Analysis Definitions and Statistical Considerations and Section 7: Statistical Considerations, the following variable is to be derived:

- Study day:
    - Calculated relative to start of study drug administration, Day 0.

Copyright © 2012 Quintiles Transnational Corp. All rights reserved.
The contents of this document are confidential and proprietary to Quintiles Transnational Corp. Unauthorized use, disclosure or reproduction is strictly prohibited.

13. CONCOMITANT MEDICATIONS

Medications taken prior to and during the conduct of the study are to be obtained from the Concomitant Medications eCRF.

All medications are coded using WHO-DD Version 01Jun2014E.

Concomitant medications and established anti-malarial treatments are to be presented separately:

- The following tables are to be presented:
  - The number (n) and percentage (%) of patients with at least one concomitant medications taken during the Treatment Period (CT), excluding established anti-malarial treatments given for persistent parasitaemia and re-emergence, are to be presented for each treatment arm by Anatomic Therapeutic Class (ATC) Level 1 and within ATC Level 1 by ATC Level 3, and within ATC Level 3 by WHO-DD Drug name for the Safety analysis set.
  - The number (n) and percentage (%) of patients with at least one established anti-malarial treatment which started on or after study drug administration or is ongoing, and is given for persistent parasitaemia and re-emergence are to be presented for each treatment arm by Anatomic Therapeutic Class (ATC) Level 1 and within ATC Level 1 by ATC Level 3, and within ATC Level 3 by WHO-DD Drug name where ATC Level 3 is equal to ANTIMALARIALS for the Intent to Treat analysis set.
- The following data are to be presented in by-patient listings:
  - Prior and concomitant medications (excluding established anti-malarial medications given for persistent parasitaemia and re-emergence) including the derived P/CT/CF flag for the All Patients Randomised analysis set.
  - All established anti-malarial treatments taken during the Treatment Period (CT) or Follow-up Period (CF) given for persistent parasitaemia and re-emergence either after switch to established anti-malarial treatment, or premature discontinuation due to other malaria species for the Safety analysis set.

13.1. DERIVATIONS

Based on the above assessments and with reference to Section 6: General Analysis Definitions and Statistical Considerations and Section 7: Statistical Considerations, the following variables are to be derived:

- Study day: Calculated relative to start of study drug administration, Day 0.
- Medications are derived and allocated to a study period as follows:
  - Prior medication (Screening Period) (P):

    Any medication which started and stopped before the start of administration of study drug.
  - Concomitant medications taken during the Treatment Period (CT):

    Any medication which started or stopped on or after study drug administration up to and including Day 28, or is ongoing.
  - Concomitant medications taken during the Follow-up Period (CF):

    Any medication which started or stopped after Day 28, or is ongoing.

Copyright © 2012 Quintiles Transnational Corp. All rights reserved.
The contents of this document are confidential and proprietary to Quintiles Transnational Corp. Unauthorized use, disclosure or reproduction is strictly prohibited.

- Established anti-malarial treatment given for persistent parasitaemia and re-emergence:

  ATC Level 3 text equal to 'ANTIMALARIALS' and given during the Treatment Period (CT) or during the Follow-up Period (CF) for persistent parasitaemia and re-emergence.

Incomplete dates (day and/or month and/or year missing):

- In case of a partial start date, the medication is allocated to a study period using the available partial information, no imputation is done. If, for a medication start date, only month and year is available, this date is compared with the month and year information of the study period.
- In case of a completely missing start date, the medication is considered as having started before the study. In case of a completely missing stop date, the medication is considered as ongoing at the end of the study.

14. STUDY DRUG ADMINISTRATION AND COMPLIANCE

The individual subject treatment pack number and weight-adjusted dose are to be collected from the Randomisation eCRF and randomisation Excel file obtained from Cenduit IWRS.

Study drug is to be administered on Day 0, 0 hours, according to different weight bands. Refer to protocol Table 5: Treatments and Dosage for more information. Patient's ≥ 5 kg and < 35 kg are to receive weight-adjusted doses predicted to achieve similar exposure ranges to patient's ≥ 35 kg.

Definitions:

- Re-dosed: Only patients who vomited within (≤) 5 minutes of start of study drug administration are to be re-dosed.
- Successfully re-dosed: Consumed total volume of study drug prepared and did not vomit within (≤) 4 hours after study drug re-administration, according to protocol.
- Compliant: Patient who consumed total volume of study drug prepared and did not vomit or was re-dosed successfully according to protocol.

Study drug administration and compliance are to be listed and summarised for the Safety analysis set by region/age group, weight group and region/centre as follows:

- Descriptive statistics are to be provided for the following:
  - Patients compliant.
  - Initial study drug administration:
    - Total volume of study drug administered.
    - Vomited after administration started:
      - ≤ 5 minutes of administration start (eCRF).
    - Time to vomiting since administration start group:
      - ≤ 5 minutes after administration.
      - > 5 minutes to ≤ 4 hours after administration.
      - > 4 hours after administration.
    - Time to vomiting since administration start (minutes).

Copyright © 2012 Quintiles Transnational Corp. All rights reserved.
The contents of this document are confidential and proprietary to Quintiles Transnational Corp. Unauthorized use, disclosure or reproduction is strictly prohibited.

- Re-dose:
    - Total volume of study drug administered.
    - Vomited after administration started:
        - ≤ 5 minutes of administration start (eCRF).
    - Time to vomiting since administration start group:
        - ≤ 5 minutes after administration.
        - > 5 minutes to ≤ 4 hours after administration.
        - > 4 hours after administration.
    - Time to vomiting since administration start (minutes).

- The data are also to be presented in the following by-patient listing:
    - Study drug administration and compliance.

14.1. Derivations

Based on the above assessments and with reference to Section 6: General Analysis Definitions and Statistical Considerations and Section 7: Statistical Considerations, the following variables are to be derived:

- Study day: Calculated relative to start of study drug administration, Day 0.
- Duration (minutes) of study drug administration: Calculated as the difference between start and end time of study drug administration:
    - Duration (minutes) = (End date and time of study drug administration - Start date and time of study drug administration).
- Time to vomiting since administration start (minutes): Calculated as the difference in minutes between start date and time of vomiting and the start date and time of study drug administration. Only to be calculated if patient vomited = 'Yes'.

  Time to vomiting since administration start (minutes) = (Start date and time of vomiting – Start date and time of study drug administration).
- Successful re-dosing: 'Yes', if patient consumed total volume of study drug prepared and did not vomit within (≤) 4 hours after start of administration.
- Compliant:
    - 'Yes', if the patient received the total volume of study drug prepared and did not vomit within (≤) 4 hours or was successfully re-dosed according to protocol. If a patient vomited after (>) 4 hours the patient is to be regarded as compliant to study drug administration.
    - 'No', if the patient did not receive the total volume of study drug or vomited within (≤) 4 hours and was not successfully re-dosed according to protocol.

Copyright © 2012 Quintiles Transnational Corp. All rights reserved.
The contents of this document are confidential and proprietary to Quintiles Transnational Corp. Unauthorized use, disclosure or reproduction is strictly prohibited.

15. Efficacy Outcomes

15.1. Objectives

The following objectives are to be analysed by Quintiles Biostatistics defining the scope of the efficacy outcomes below.

- Primary efficacy objective:
    - The primary objective is to determine whether a single dose combination of OZ439/PQP is an efficacious treatment for uncomplicated *P. falciparum* malaria in adults and children.
        - PCR-adjusted ACPR at Day 28.
- The secondary objectives are:
    - To determine the incidence of recrudescence and new infection.
        - PCR-adjusted ACPR at Day 14, 21, 42 and 63 (selected centres only).
        - Crude ACPR at Day 14, 21, 28, 42 and 63 (selected centres only).
        - Kaplan-Meier analysis presentation for incidence rate up to Day 42 or 63 (selected centres only):
            - Re-emergence.
            - Recrudescence.
            - New infection.
    - To determine the time to relief of fever and parasite clearance.
        - Parasite clearance time (PCT).
        - Fever clearance time (FCT):
            - Patients with fever present at Baseline.
            - Patients with fever at Baseline, excluding patients receiving paracetamol.
        - Parasite reduction ratio (PRR).
            - PRR24 and PRR48.
- The exploratory objectives:
    - To evaluate the proportion of patients with gametocytes at each assessment.
        - Kaplan-Meier presentation of the risk of having gametocytes for:
            - Patients with gametocytes at Baseline to time of clearance of gametocytes.
            - Patients with no gametocytes at Baseline to time of appearance of gametocytes.
    - To examine the relationship between Kelch-13 genotype parasite clearance kinetics.
        - To determine parasite clearance kinetics (referred to as parameters from here on) are estimated using the WWARN PCE:
            - PC half-life, time to 50%, 90% and 99% parasite reduction.
        - Correlation between Kelch-13 genotype status and parasite clearance kinetics:
            - Kelch-13 and PC half-life.
- All efficacy analyses are to be presented for the following subgroups (refer to Section 7.5 Examination of Subgroups) as relevant:
    - Region/Age group.
    - Weight group.
    - Region/Centre.

Copyright © 2012 Quintiles Transnational Corp. All rights reserved.
The contents of this document are confidential and proprietary to Quintiles Transnational Corp. Unauthorized use, disclosure or reproduction is strictly prohibited.

15.2. DEFINITIONS

Treatment outcome is to be established according to the following modified standard World Health Organisation (WHO) classification. Refer to Glossary of the protocol, according to a modification of the standard WHO classification, WHO Methods of Surveillance of antimalarial drug efficacy, 2009:

- Early treatment failure (ETF) (Day 1 to 3) = 'Yes' if any of the following criteria are met:
  - Danger signs or severe malaria on Day 1, 2 or 3 in the presence of parasitaemia.
  - Parasitaemia on Day 2 higher than on Day 0, irrespective of axillary temperature.
  - Parasitaemia on Day 3 with axillary temperature ≥ 37.5 ºC.
  - Parasitaemia on Day 3 ≥ 25% of count on Day 0.
- Late clinical failure (LCF) (Day 4 to (D)) = 'Yes' if any of the following criteria are met:
  - Danger signs or severe malaria in the presence of parasitaemia on any day between Day 4 and Day (D) in patients who did not previously meet any of the criteria of ETF.
  - Presence of parasitaemia on any day between Day 4 and Day (D) with axillary temperature ≥ 37.5 ºC (or history of fever) in patients who did not previously meet any of the criteria of ETF.
  - At 96 hours (Day 4) post-dose: Failure to achieve parasite clearance irrespective of axillary temperature in patients who did not previously meet any of the criteria of ETF.
- Late parasitological failure (LPF) = 'Yes' if any of the following criteria are met:
  - Presence of parasitaemia on any day between Day 7 and Day (D) and axillary temperature < 37.5 ºC in patients who did not previously meet any of the criteria of ETF or LCF.
- Treatment failure: Defined as patients who met any of the criteria for early treatment failure, late clinical failure or late parasitological failure.
- ACPR: Defined as absence of parasitaemia on Day (D), irrespective of axillary temperature, in patients who did not previously meet any of the criteria of treatment failure.
- Crude ACPR: Defined as APCR with no evidence of re-emergence of asexual parasites up to a given endpoint (D).
- PCR-adjusted ACPR: Defined as ACPR with no evidence of recrudescence of asexual parasites based on PCR-analysis of genotype up to a given endpoint Day (D).

Copyright © 2012 Quintiles Transnational Corp. All rights reserved.
The contents of this document are confidential and proprietary to Quintiles Transnational Corp. Unauthorized use, disclosure or reproduction is strictly prohibited.

The following variables are to be used in the definitions of both crude and PCR-adjusted ACPR at Day (D):

- *P. falciparum* asexual parasite count and other species results as obtained from the Thick and Thin Malaria Blood Film eCRF.
    - Parasitaemia refers to *P. falciparum* asexual parasites present (count > 0 parasites/µL).
    - Day 0 refers to Baseline *P. falciparum* asexual parasites (/µL).
    - Body temperature as obtained on the Temperature eCRF (refer to Section 6.7.2 Adjusted Body Temperature and Fever).
    - Presence of signs and symptoms of complicated/severe malaria as obtained from the Clinical Signs/Symptoms of Uncomplicated Malaria eCRF.
    - PCR-adjusted result: The genotype/clone result of the *P. falciparum* parasites present at the time of re-emergence after Day 7 compared to the Baseline genotype/clone, only if applicable. Recrudescence is distinguished from new infection by genotyping the parasite clone by PCR-analysis.
    - PCR-analysis results:
        - Recrudescence: Re-emergence of asexual parasites with a genotype identical to that of parasites present at Baseline based on PCR-analysis of genotype.
        - New infection: Re-emergence of asexual parasites with a genotype different to that of parasites present at Baseline based on PCR-analysis of genotype.
        - Indeterminate. If the genotype of the parasite cannot be analysed following re-emergence due to a missing sample or unusable sample The reasons for the missing data must be indicated; i.e., sample not taken, missing follow-up sample, no PCR result or not interpretable result.
        - Negative. 'No PCR result', hence 'No' *P. falciparum* asexual parasites present for genotyping.
- In general:
    - Baseline: Defined as the second thick blood film result obtained at Screening before study drug administration.
    - Absence of parasites: Defined as absence of *P. falciparum,* count = 0 (/µL), hence parasite free. Negative parasite film - when no parasites are seen while the full number of white cells have been counted then the parasite count is recorded as zero. Of course this means only that the count is below the limit of detection, although it is often reported or modelled as 0 /µL.
    - Presence of parasites defined as *P. falciparum* asexual forms count > 0 (/µL).
    - Initial clearance: Defined as the absence of asexual parasites for two consecutive negative films taken within an interval of (≥) 6 to (≤) 12 hours.
    - Re-emergence: Defined as the appearance of asexual parasites after clearance of initial infection irrespective of genotype.
    - Appearance (gametocytes): Defined as patients without gametocytes present at Baseline who develop gametocytes during the study.

Copyright © 2012 Quintiles Transnational Corp. All rights reserved.
The contents of this document are confidential and proprietary to Quintiles Transnational Corp. Unauthorized use, disclosure or reproduction is strictly prohibited.

- Parasite clearance parameters:

  The following parasite clearance parameters are estimated using the WWARN parasite clearance estimator based on the linear part of the individual natural log parasitaemia-time profiles for both microscopic and qPCR determined parasitaemia:

  - Clearance rate constant (1/hours): Defined as the minus slope of the final model fitted after exclusion of outliers, lag phase and tail.
  - PC half-life (hours): Estimated time for parasitaemia to decrease by half.
  - PCXX (hours): Estimated time for parasitaemia to reduce by XX% relative to Baseline value.
  - PRRXX: Calculated as the natural log change (clearance rate constant) in parasitaemia over a time interval (24 and 48 hours) based on the linear part of the natural log regression profile.

15.3. Crude and PCR-adjusted Outcome Derivation

The below table summarises the treatment outcome assignment for both crude and PCR-adjusted ACPR at Day (D). Possible treatment outcome for crude and PCR-adjusted ACPR at Day (D) are:

- 1 = Cure.
- 2 = Failure.
- 3 = Missing.

Table 2 Derivation Criteria of Treatment Outcome Crude ACPR and PCR-adjusted ACPR at Day (D)

**Criteria**	**PP Analysis Set**		**ITT Analysis Set**	
	**Treatment Outcome Crude ACPR**	**Treatment Outcome PCR-adjusted ACPR**	**Treatment Outcome Crude ACPR**	**Treatment Outcome PCR-adjusted ACPR**
Completed up to Day (D) without re-emergence (new infection/ recrudescence) of parasites after initial clearance, thus ACPR at Day (D).	Cure	Cure	Cure	Cure
Missing assessment on Day (D), but parasite free (No *P. falciparum* asexual parasites) after Day (D).	Cure	Cure	Cure	Cure
Missing assessment on Day (D), no more assessments thereafter.	Missing	Missing	Failure	Failure
Missing assessment on Day (D), recrudescence (confirmed by PCR-adjusted result) at first assessment after Day (D).	Failure	Failure	Failure	Failure
Missing assessment on Day (D), new infection (confirmed by PCR-adjusted result) at first assessment after Day (D).	Failure	Cure	Failure	Cure
New infection before Day (D) (thus on or after Day 7 to before (D))	Failure	Missing	Failure	Failure
New infection on Day (D).	Failure	Cure	Failure	Cure
Recrudescence before or on Day (D) (thus on or after Day 7 to [D])	Failure	Failure	Failure	Failure

Copyright © 2012 Quintiles Transnational Corp. All rights reserved.
The contents of this document are confidential and proprietary to Quintiles Transnational Corp. Unauthorized use, disclosure or reproduction is strictly prohibited.

Re-emergence before or on Day (D) but PCR-adjusted result is indeterminate, negative or missing.	Failure	Missing	Failure	Failure
Late clinical failure from Day 4 to Day 6.	Failure	Failure	Failure	Failure
Early treatment failure from Day 1 to Day 3.	Failure	Failure	Failure	Failure
Other *Plasmodium* species before Day (D) (in the absence of *P. falciparum*).	Missing	Missing	Failure	Failure
Other *Plasmodium* species on Day (D) (in the absence of *P. falciparum*).	Cure	Cure	Cure	Cure
Other *Plasmodium* species before or on Day (D) (in the presence of *P. falciparum*), PCR-analysis result missing, negative or indeterminate.	Failure	Missing	Failure	Failure
Prematurely study discontinued from the study before Day (D) and received SoC.	Failure	Missing	Failure	Failure
Prematurely discontinued from the study before Day (D) and no record of SoC.	Missing	Missing	Failure	Failure

ACPR: Adequate clinical and parasitological response. D = Endpoint Day. ITT: Intent to Treat. PP: Per Protocol. PCR: Polymerase chain reaction. SoC: Standard of care.

Patients included in the ITT analysis set with no post-baseline efficacy data available are to be regarded as failure for both the crude and PCR-adjusted ACPR at Day (D) treatment outcome assignments.

- Based on the above definitions and derivations according to the criteria set out in Table2: Derivation Criteria of Treatment Outcome Crude ACPR and PCR-adjusted ACPR at Day (D), crude and PCR-adjusted ACPR at Day (D) are to be derived talking into account the following analysis visit windows for Day (D).

Table 3: Crude and PCR-adjusted ACPR analysis endpoints and Windows:

**Endpoint*<br>Day (D)**	**Analysis Timepoint (Numeric Version)**	**Window**	**Analysis Visit Window (Days)**	**Unscheduled to be Used Only if Endpoint Day (D) Not Available**
Day 14	18	-2/+3 days	[12,17]	14
Day 21	20	-2/+3 days	[19,24]	21
Day 28	22	-2/+3 days	[26,31]	28
Day 42	23	- 3/+5 days	[39,47]	42
Day 63	24	- 3/+5 days	[60,68]	63

Endpoint Day (D): On or after study drug administration up to time of Day (D) taking into account the visit window, premature study discontinuation, switch to established anti-malarial treatment or start of any other treatment with anti-malarial activity as captured on the Prior and Concomitant Medications eCRF, whichever is earliest.

Preference is to be given to the actual endpoint Day (D) scheduled visit result, if it falls within the predefined visit window. In cases where there is no endpoint visit, and an unscheduled visit falls within the analysis window of Day (D), the unscheduled data is to be used as the endpoint result. If neither exists the endpoint result at Day (D) is regarded as missing and subsequently the crude and PCR-adjusted ACPR is to be derived using supporting data as detailed in Table2: Derivation Criteria of Treatment Outcome Crude ACPR and PCR-adjusted ACPR at Day (D).

Copyright © 2012 Quintiles Transnational Corp. All rights reserved.
The contents of this document are confidential and proprietary to Quintiles Transnational Corp. Unauthorized use, disclosure or reproduction is strictly prohibited.

15.4. Primary Efficacy Endpoint

The primary efficacy variable is PCR-adjusted ACPR treatment outcome at Day 28. For the purpose of the derivations the efficacy endpoints at Day 14, 21, 28, 42 and 63 are to be referred to as Day (D).

15.4.1. Primary Analysis of Primary Efficacy Variable(s)

The primary analysis is to be performed for the PP analysis set, and repeated for the ITT and mPP analysis sets.

- For analysis purposes the PCR-adjusted ACPR at Day 28 is to be categorised as:
  - 1 = Cure.
  - 0 = Failure.
- For the PP and mPP analysis sets:
  - Only a definite 'Cure' or 'Failure' result as derived per Table2: Derivation Criteria of Treatment Outcome Crude ACPR and PCR-adjusted ACPR at Day (D) is to be used in the analysis.
  - n = Number of patients with the treatment outcome of 'Cure'.
  - r = Total number of patients in the relevant analysis set with a definite response of 'Cure' or 'Failure'.
  - % = Percentage of patients in each category calculated relative to the total number of patients in the relevant analysis set with a definite response.
  - PCR-adjusted ACPR rate*at Day (D) = (Number of patients with the treatment outcome 'Cure')/(Number of patients in the relevant analysis set with a definite result) * 100.
- For the ITT analysis set:
  - Missing response as derived per Table2: Derivation Criteria of Treatment Outcome Crude ACPR and PCR-adjusted ACPR at Day (D) is to be set to failure and included in the analysis.
  - n = Number of patients with the treatment outcome of 'Cure'.
  - N = Total number of patients in the relevant analysis set.
  - % = Percentage of patients in each category to be calculated relative to the total number of patients in the analysis set.
  - PCR-adjusted ACPR rate at Day (D) = (Number of patients with the treatment outcome 'Cure'/Number of patients in the ITT analysis set) * 100.
- Incidence calculation: Based on analysable results up to and including the date of study completion, premature study discontinuation, including switch to established anti-malarial treatment or start of any other treatment with anti-malarial activity as captured on the Prior and Concomitant Medications eCRF, whichever is earliest.

Copyright © 2012 Quintiles Transnational Corp. All rights reserved.
The contents of this document are confidential and proprietary to Quintiles Transnational Corp. Unauthorized use, disclosure or reproduction is strictly prohibited.

Method 1

A 95% Clopper-Pearson 2-sided CI is to be constructed around the single binomial proportion per treatment arm and total.

The following SAS® code is to be used to calculate the 95% Clopper-Pearson 2-sided CI for a single binomial proportion:

```
Proc freq data = SETS1;
  by ASETN ASET SORT1 TRT01A;
  tables AVAL / out = count binomial (all) alpha = .05;
  ODS OUTPUT binomialCLs = METH1;
run;
```

- Descriptive statistics for the ITT, PP and the mPP analysis set by each of the following subgroups:
  - Region/age group.
  - Weight group.
- Refer to Section 7.5: Examination of Subgroups for a detailed description of the levels of stratification within each subgroup.
- The crude and PCR adjusted ACPR at additional timepoints are to be analysed and presented in a similar manner as described for the overall primary efficacy ACPR rate.
- The following tables are to be presented for the ITT, PP and mPP analysis sets:
  - The number and percentage (%) of patients with a treatment failure outcome, including ETF, LCF and LPF up to Day (D).
  - The number and percentage (%) of patients with a PCR-adjusted result following re-emergence of asexual parasites, i.e. recrudescence, new infection, indeterminate and negative up to Day (D).
  - Descriptive statistics and 95% confidence intervals (calculated according to Clopper-Pearson method, Method 1) are to be calculated for the Crude and PCR-adjusted ACPR rate at Day (D).
- The following bar chart is to be presented for the ITT, PP and mPP analysis sets:
  - Separate bar charts of crude and PCR-adjusted cure rate over time.
- The following by-patient listing is to be presented for the ITT, PP and mPP analysis set:
  - Crude and PCR-adjusted ACPR and treatment failure (ETF, LCF and LPF).

Copyright © 2012 Quintiles Transnational Corp. All rights reserved.
The contents of this document are confidential and proprietary to Quintiles Transnational Corp. Unauthorized use, disclosure or reproduction is strictly prohibited.

15.5. SECONDARY EFFICACY ENDPOINTS

- The secondary efficacy endpoints are:
    - PCR-adjusted ACPR at Day 14, 21, 42 and 63 (only selected centres).
    - Crude ACPR at Day 14, 21, 28, 42 and 63 (only selected centres).
    - Kaplan-Meier analysis up to Day 42 or 63 (only selected centres) of:
        - Re-emergence.
        - Recrudescence.
        - New infection.
    - Parasite clearance time (PCT).
    - Fever clearance time (FCT).
    - Parasite reduction ratio (PRR).

15.5.1. PCR-ADJUSTED ACPR AT DAY 14, 21, 42 AND 63, AND CRUDE ACPR AT DAY 14, 21, 28, 42 AND 63

The PCR-adjusted and crude ACPR at Day 14, 21, 42 and 63 are defined, analysed and presented in a similar manner as for the primary efficacy endpoint PCR-adjusted ACPR at Day 28 (refer to Section 15.3: Crude and PCR-adjusted Outcome Derivation).

15.5.2. KAPLAN-MEIER ANALYSIS FOR TIME TO RE-EMERGENCE, RECRUDESCENCE AND NEW INFECTION

- The following time to event variables are to be derived:
    - Time to re-emergence (days): Defined as the time to appearance of asexual parasites after clearance of initial infection irrespective of genotype.
    - Time to recrudescence (days): Defined as the time to appearance of asexual parasites after clearance of initial infection with a genotype identical to that of parasites present at Baseline.
    - The time to new infection (days): Defined as the time to appearance of asexual parasites after clearance of initial infection with a genotype different to that of parasites present at Baseline.
    - Time to event (day): Calculated as the difference in days between date of event and date of study drug administration.
- Censored: Patients with no event are censored at the time of study completion, premature study discontinuation, including switch to established anti-malarial treatment or start of any other treatment with anti-malarial activity as captured on the Prior and Concomitant Medications eCRF, whichever is earliest.
- The time to event is calculated from date of study drug administration to the onset of the event in days.
- Calculated as the difference in days between date of event and date of study drug:

  Time to event (days) = [Date of event – Start date of study drug administration]

- The median time to event and 95% CI for each treatment arm is to be estimated using the Kaplan-Meier method described in Method 2.
- The following is to be presented for the for the ITT analysis set: Subset by region/age group:

Copyright © 2012 Quintiles Transnational Corp. All rights reserved.
The contents of this document are confidential and proprietary to Quintiles Transnational Corp. Unauthorized use, disclosure or reproduction is strictly prohibited.

- o Kaplan-Meier estimates of time to re-emergence, recrudescence and new infection of asexual parasites in days.
- o Kaplan-Meier plots of the survival function and cumulative incidence for each treatment arm is to be presented for time to re-emergence, recrudescence and new infection of asexual parasites in days.

- **Method 2:**

The following SAS® code is to be used to calculate the Kaplan-Meier and median time to event:

```
Proc sort SET1;
  By Sort1 (Region, Age group, TRT, TTE (hours/days))
run;

Proc lifetest data = SETS1 OUTSURV = SETS2
 Conftype = linear Method = KM
 Alpha = 0.05 Alphaqt = 0.05 atrisk;
 Time TTE * CNSR (0);
  by Sort2 ; /*Same as dataset proc sort excluding TTE*/
run;
```

Where:

- o TTE = Time to onset of event.
- o CNSR (or STATUS) = 0/1
- o Sort2 = Region, Age group, TRT.

15.5.3. PARASITE CLEARANCE TIME

The following clearance time variables are to be derived:

- Parasite clearance time (PCT) (hours):
  - o Defined as the time (in hours) from start of study drug administration until the time of first negative film (no asexual parasites). This negative film is to be confirmed by a second negative film, taken within (≥) 6 to (≤) 12 hours of the first. Parasite clearance is concluded following confirmation of the second negative film. A deviation of 2 hours is allowed for the time interval.
  - o Time to clearance is to be calculated as the time in hours from time of study drug administration to the time of clearance and is to be used to show actual time of clearance.

    Time to clearance (hours) = [Date and time of clearance – Start date and time of study drug administration]
  - o Censored: Patients who did not have asexual parasite clearance are censored at the time of 96 hours, premature study discontinuation, including switch to established anti-malarial treatment or start of any

Copyright © 2012 Quintiles Transnational Corp. All rights reserved.
The contents of this document are confidential and proprietary to Quintiles Transnational Corp. Unauthorized use, disclosure or reproduction is strictly prohibited.

other treatment with anti-malarial activity as captured on the Prior and Concomitant Medications eCRF, whichever is earliest.

- The proportion of patients who have parasite clearance after study drug administration are to be calculated and the 95% confidence intervals for the single binomial proportion is to calculated according to Clopper-Pearson method (Method 1).
- The parasite clearance time is to be analysed using Kaplan-Meier estimates (refer to Method 2). The median and quartiles clearance time is to be presented together with the corresponding 95% confidence intervals for the median.
- Percentage parasite clearance achieved:
  - At 24 hours (Day 1): number of patients with PCT ≤ 24 hours.
  - At 48 hours (Day 2): number of patients with PCT ≤ 48 hours.
  - At 72 hours (Day 3): number of patients with PCT ≤ 72 hours.
- The following is to be presented for the PP analysis set by region/age group and region/centre (Asia centres only):
  - Kaplan-Meier estimates of time to asexual parasite clearance time in hours, including the proportion of patients (%) who have cleared parasites at 24 hours (Day 1), 48 hours (Day 2) and 72 hours (Day 3).
  - Kaplan-Meier plots of the survival function and cumulative incidence for each treatment arm over time to parasites clearance in hours.

15.5.4. FEVER CLEARANCE TIME

- Fever clearance time (FCT) (hours):
  - Only patients with measured fever (adjusted body temperature ≥ 37.5 °C) present at Baseline are to be included. Patients entered in the study on the basis of history of fever and who do not subsequently have an increased body temperature measurement indicating presence of fever at pre-dose, are not be included in the analysis of fever clearance time.
  - Calculated as the time from start of study drug administration to the first assessment of adjusted body temperature < 37.5 ^{o}C. This assessment is to be confirmed by a second assessment, taken within (≥) 6 to (≤) 12 hours of the first. Fever clearance is to be concluded for the first assessment, following confirmation of temperature < 37.5 °C on the second assessment.

    Time to clearance (hours) = [Date and time of clearance – Start date and time of study drug administration]
  - Censored: Patients who did not have fever clearance are censored at 96 hours, premature study discontinuation, including switch to established anti-malarial treatment or start of any other treatment with anti-malarial activity as captured on the Prior and Concomitant Medications eCRF, whichever is earliest.
- The proportion of patients who have fever clearance after study drug administration are to be calculated and the 95% confidence intervals for the single binomial proportion is to be calculated according to Clopper-Pearson method (Method 1).
- The fever clearance time is to be analysed using Kaplan-Meier estimates (refer to Method 2). The median and quartiles clearance time is to be presented together with the corresponding 95% confidence intervals for the median.

Copyright © 2012 Quintiles Transnational Corp. All rights reserved.
The contents of this document are confidential and proprietary to Quintiles Transnational Corp. Unauthorized use, disclosure or reproduction is strictly prohibited.

- The following tables are to be presented for the ITT and PP analysis sets by region and age group:
  - Kaplan-Meier estimates of time to fever clearance in hours for patients with measured fever (adjusted body temperature ≥ 37.5 °C) present at Baseline.
  - Kaplan-Meier estimates of time to fever clearance in hours for patients with measured fever at Baseline, excluding Only patients with measured (adjusted body temperature ≥ 37.5 °C) fever present at Baseline, excluding patients who received paracetamol on day of study drug administration until 96 hours after study drug administration are to be included.
- The following figures are to be presented for the ITT and PP analysis sets by region and age group:
  - Kaplan-Meier plots of the survival function and cumulative incidence for each treatment arm over time for patients with measured fever at Baseline to parasites clearance in hours.
  - Kaplan-Meier plots of the survival function and cumulative incidence for each treatment arm over time for patients with measured fever at Baseline to parasites clearance in hours, excluding patients receiving paracetamol during the first 96 hours after study drug administration.

15.5.5. PARASITE CLEARANCE PARAMETERS

- The following parasite clearance parameters are to be estimated using the WWARN parasite clearance estimator based on the linear part of the individual natural log parasitaemia-time profiles for both microscopic and qPCR determined parasitaemia.
  - Clearance rate constant (1/hours).
  - PC half-life.
  - Time to 50%, 90% and 99% parasite reduction (PC50, PC90 and PC99)
- Analysable data: Identifies the data per scheduled timepoint to be used for the PCE especially in view of patients switching to established anti-malarial treatment or start of any other treatment with anti-malarial activity as captured on the Prior and Concomitant Medications eCRF. This flag excludes those test results obtained whilst on alternative treatment and only data up to 168 hours post study drug administration are to be included for the parasite parameter analysis.
- Analysable result: Identifies the parasite clearance parameter results to be used in the summary tables. Results from patients with a poor fit to the linear model ($r^2 < 0.75$) or with < 3 data points are excluded from the analysis.
- For asexual parasite assessments equal to ‘Absent’, the WWARN calculator use 8 /µL as the limit of detection.
- Parasite reduction rate (PRR) at 24 and 48 hours derivations:

  The clearance rate constant (1/hours) obtained from the WWARN PCE calculator and defined as the minus slope of the final model fitted after exclusion of outliers, lag phase and tail is to be used to calculate PRR24 and PRR48. Hence PRR24 and PRR48 is the drop in log units over 24 and 48 hours.

  - PRR24 = ln (clearance rate constant [1/hours]*24 hours).
  - PRR48 = ln (clearance rate constant [1/hours]*48 hours).

Copyright © 2012 Quintiles Transnational Corp. All rights reserved.
The contents of this document are confidential and proprietary to Quintiles Transnational Corp. Unauthorized use, disclosure or reproduction is strictly prohibited.

- The following table is to be presented for the PP analysis set by region/age group and region/centre (Asia centres only).
  - o Descriptive statistics (n, minimum, 25th percentile, median, 75st percentile, maximum) for the parasite clearance parameters, i.e. PRR24, PRR48, PC half-life, PC50, PC90 and PC99 as estimated with the use of the PCE developed by WWARN and the calculated PRR24 and PRR48.
- The following result are to be presented in a by-patient listing for the ITT analysis set:
  - o The parasite clearance parameters, i.e. clearance rate constant (1/hours), PC half-life, PC50, PC90 and PC99, including the calculated PRR24 and PRR48 results.

15.5.6. PARASITE DENSITY CHARACTERISTICS

Summary statistics: Based on analysable results up to and including the date of study completion, premature study discontinuation, including switch to established anti-malarial treatment or start of any other treatment with anti-malarial activity as captured on the Prior and Concomitant Medications eCRF, whichever is earliest.

- The following table is to be presented for the ITT and the PP analysis set by region/age group:
  - o The proportion of patients with *P. falciparum* asexual and other species present at each scheduled timepoint are to be summarised. The percentage (%) of patients in each category are to be calculated relative to the total number of patients in the relevant analysis set with thick film results available at the relevant visit.
  - o The proportion of patients with gametocytes present at each scheduled timepoint are to be summarised. The percentage (%) of patients at each scheduled visit are to be calculated relative to the total number of patients in the relevant analysis set with thick film results available at the relevant visit.
- The following by-patient listing is to be presented for the Intent to Treat analysis set:
  - o Asexual parasites and gametocytes assessments, other species assessment results for all scheduled and unscheduled visits.
  - o qPCR and RT-PCR parasitemia results for all scheduled and unscheduled visits.
- The following figures is are to be presented for the Per Protocol analysis set:
  - o A line graph of the median asexual parasite counts (/µL) over time on the natural log scale up to Day 63
  - o A line graph of the median asexual parasite counts (/µL) over time on the natural log scale up to 96 hours.
  - o A bar chart of percentage of patients with gametocytes over time per scheduled visit up to Day 63.

The SAS® procedure GPLOT is to be used with the following code:

For the median log asexual parasites over time on the natural log scale:

```
Proc gplot data = DD;
        Plot x = median / group = TRT01A;
Run;
```

- In view of log-transformation, the asexual assessment results equal to 'Absent' are to be set to 8 /µL as the limit of detection before Log-transformation (as handled by the WWARN PCE calculator).
- **Media**= Median of natural log transformed asexual parasites, $\log_e$ (Asexual parasites).
- **TRT01A** = Actual treatment arm.

Copyright © 2012 Quintiles Transnational Corp. All rights reserved.
The contents of this document are confidential and proprietary to Quintiles Transnational Corp. Unauthorized use, disclosure or reproduction is strictly prohibited.

15.6. Exploratory Efficacy Endpoints

15.6.1. Kaplan-Meier Analysis for the Risk of Having Gametocytes

The following time to event variables are to be derived:

- The time to appearance (days):
  - Only patients without gametocytes at Baseline are to be included, i.e. gametocytes equal to 'Absent' at Screening (Baseline).
  - Calculated from the start of study drug administration until the appearance of gametocytes.

    Time to appearance of gametocytes (days) = (Date of appearance of gametocytes – Date of study drug administration).
  - Censored: Patients who did not have an event of appearance of gametocytes are censored at the time of study completion, premature study discontinuation, including switch to established anti-malarial treatment or start of any other treatment with anti-malarial activity as captured on the Prior and Concomitant Medications eCRF, whichever is earliest.
- Gametocyte clearance time (PCT) (days):
  - Only patients with gametocytes present at Baseline are to be included, i.e. gametocytes assessment equal to 'Present' at Screening (Baseline) with a gametocyte count greater than zero.
  - Calculated as the time from start of study drug administration to the first negative (no gametocytes detected) film. This negative film is to be confirmed by a second negative film, taken within (≥) 6 to (≤) 12 hours of the first. Gametocyte clearance is concluded following confirmation of the second negative film.

    Time to gametocytes clearance (days) = (Date of gametocytes clearance – Date of study drug administration).
  - Censored: Patients who did not have gametocyte clearance are censored at the time of study completion, premature study discontinuation, including switch to established anti-malarial or start of any other treatment with anti-malarial activity as captured on the Prior and Concomitant Medications eCRF, whichever is earliest.
- The median time to event and 95% CI for each treatment are to be estimated using the Kaplan-Meier described in Method 2.
- The following tables are to be presented for the PP analysis set by region/age group:
  - Kaplan-Meier estimates of time to appearance of gametocytes in days, for patients with no gametocytes at Baseline. The percentage of patients in each category are to be calculated relative to the total number of patients in the relevant analysis set without gametocytes at Baseline.
  - Kaplan-Meier estimates of gametocyte clearance time in days, for patients with gametocytes at Baseline. The percentage (%) of patients in each category are to be calculated relative to the total number of patients in the relevant analysis set with gametocytes present at Baseline.

Copyright © 2012 Quintiles Transnational Corp. All rights reserved.
The contents of this document are confidential and proprietary to Quintiles Transnational Corp. Unauthorized use, disclosure or reproduction is strictly prohibited.

- The following figures are to be presented:
  - Kaplan-Meier plots of the survival function and cumulative incidence for each treatment arm is to be presented for time to appearance of gametocytes for patients without gametocytes present at Baseline.
  - Kaplan-Meier plots of the survival function and cumulative incidence for each treatment arm is to be presented for gametocytes clearance time for patients with gametocytes present at Baseline.

15.6.2. KELCH-13 GENOTYPE

Analysis of K13 genotype associated with Artemisinin resistance is to be carried out on the pre-dose blood spot samples. If insufficient blood spot samples at Baseline, alternative blood spots collected as specified in the schedule of assessments are to be used.

- Binary Classification:
  - Per patient: Kelch-13 data is to be categorised as either true wild type or mutation, where:
    - True wild type (WT): Defined for patient with no mutations at any of the tested loci.
    - Mutation: Defined for patient with at least one mutation of any tested loci.
- The following table is to be presented for the PP analysis set by region/centre:

  Number (n) and percentage (%) of patients in each category are to be calculated relative to the total number of patients in the relevant analysis set with an evaluable result (‘wild type’ or ‘mutation’) at any of the tested loci per patient overall and per tested loci.
- The following data are to be presented in a by-patient for the intent to Treat analysis set:
  - Kelch-13 mutations and wild types.
- The following figures are to be presented by region/ centre:
  - Stacked bar chart of the frequency of occurrence of Kelch-13 of wild type and mutations. The percentage (%) of patients in each category are to be calculated relative to the total number of patients in the relevant analysis set with an evaluable result (‘wild type’ or ‘mutation’ at any of the tested loci.
  - Scatter plot of the correlation of parasite clearance half-life and kelch-13 wild type and mutations. PC half-life are estimated using the WWARN parasite clearance estimator.

15.6.3. BODY TEMPERATURE AND FEVER

Body temperature as obtained from the Temperature eCRF.

- Baseline: Defined as the earliest assessment before study drug administration.
- Summary statistics: Based on analysable results up to and including the date of study completion, premature study discontinuation, including switch to established anti-malarial treatment or start of any other treatment with anti-malarial activity as captured on the Prior and Concomitant Medications eCRF, whichever is earliest.
- The following table is to be presented for the PP analysis set by region/age group:
  - Descriptive statistics for adjusted body temperature assessments, including change from Baseline.
  - Number (n) and percentage (%) of patients having fever per scheduled visit, calculated relative to the total number of patients in the relevant analysis set.

Copyright © 2012 Quintiles Transnational Corp. All rights reserved.
The contents of this document are confidential and proprietary to Quintiles Transnational Corp. Unauthorized use, disclosure or reproduction is strictly prohibited.

- The following by-patient listing is to be presented for the Intent to Treat analysis set:
  - Adjusted body temperature, including change from Baseline and fever (refer to Section 6.7.2: Adjusted Body Temperature and Fever).

15.6.4. CLINICAL SIGNS AND SYMPTOMS OF MALARIA

Presence of clinical signs and symptoms of complicated/severe malaria and each of the clinical signs and symptoms of uncomplicated malaria as obtained from the Clinical Signs/Symptoms of Uncomplicated Malaria eCRF.

- The following table is to be presented for the PP analysis set by region/age group:
  - Number (n) and percentage (%) of patients having any signs and symptoms of complicated/severe malaria as well as any signs and symptoms (including other) for uncomplicated malaria per scheduled visit, calculated relative to the total number of patients in the relevant analysis set.
- The following by-patient listing is to be presented for the Intent to Treat analysis set:
  - Clinical signs and symptoms of malaria, including information on clinical signs and symptoms of uncomplicated malaria present.

Copyright © 2012 Quintiles Transnational Corp. All rights reserved.
The contents of this document are confidential and proprietary to Quintiles Transnational Corp. Unauthorized use, disclosure or reproduction is strictly prohibited.

16. Safety Outcomes

All safety analyses are based on the Safety analysis set.

Only descriptive summaries of the safety data are to be provided as detailed in the latest version of the TLF shells document. All available safety data are to be presented in by-patient listings.

16.1. Adverse Events

16.1.1. Definitions

An AE is defined as any untoward medical occurrence in a patient administered study drug that does not necessarily have a causal relationship with the treatment. An AE can therefore be any unfavourable and unintended sign (i.e., an abnormal laboratory finding), symptom (i.e., rash, pain, discomfort, fever, dizziness, etc.), disease (i.e., peritonitis, bacteraemia, etc.) or outcome of death temporally associated with the use of study drug, whether or not considered causally related to the study drug.

Adverse events are coded using MedDRA Version 17.0.

For the purpose of the analysis, adverse events (AEs) are allocated to the study periods as follows. Refer to Section 3.1 General Description.

Flags are to be derived to indicate if the adverse event is a pre-treatment adverse event (P), treatment-emergent adverse event (TEAE) (T) or follow-up adverse event (F) based on the start date of the event as follows:

- Pre-treatment adverse event (P): Defined as any AE which started or worsened on or after informed consent and before study drug administration.
- TEAE (T): Defined as any AE which started or worsened on or after study drug administration up to and including Day 28.
- Follow-up adverse event (F): Defined as any AE which started or worsened after Day 28.
- The flags are mutually exclusive, meaning AEs can only be assigned to one period.

In general:

- All AEs recorded on the Adverse Events eCRF.
- A serious AE is defined as an AE with the result ‘Yes’ answered for the question ‘Serious event’ in the Adverse Events eCRF.
- AEs leading to death are defined as AEs with an outcome recorded as ‘Death’ or serious event criteria recorded as ‘Death’ on the Adverse Events eCRF. AEs leading to premature discontinuation of study are defined as AEs with action taken as ‘Patient withdrawn from study’ on the Adverse Events eCRF.
- AEs related to study drug are defined as an AE with the result ‘definitely related’ or ‘probably related’ or ‘possibly related’ answered for the question ‘Relationship to study drug’ on the Adverse Events eCRF. Any other result for this question is considered not related to study drug. Any AE with a missing relationship is considered related to study drug.
- Severe or life-threatening AE is defined as an AE with the result ‘Grade 3 (Severe)’ or ‘Grade 4 (Life-threatening’ answered for the question ‘AE severity’ or missing severity on the Adverse Events eCRF.

Copyright © 2012 Quintiles Transnational Corp. All rights reserved.
The contents of this document are confidential and proprietary to Quintiles Transnational Corp. Unauthorized use, disclosure or reproduction is strictly prohibited.

- AEs of special interest are defined in Section 7.16.4 Adverse Events of Special Interest of the protocol.

16.1.2. ANALYSIS METHODS

- An overview is to be provided for the following treatment-emergent adverse events (TEAEs) per the defined Treatment Period together with the actual number of mentions (events):
  - TEAEs.
  - TEAEs leading to premature study discontinuation.
  - TEAEs leading to study drug discontinuation.
  - Grade 3 (severe) or grade 4 (life-threatening) TEAEs.
  - TEAE of special interest (TEAESI) (eCRF).
  - No TEAEs (number of patients for whom no AEs are reported within the defined Treatment Period).
  - Treatment-emergent serious adverse events (TESAE).
    - TESAEs leading to death.
    - TESAEs of drug-induced liver toxicity (Hy's Law).
    - TESAEs leading to premature study discontinuation.
    - TESAEs leading to study drug discontinuation.
    - Grade 3 (severe) or grade 4 (life-threatening) TESAEs.
    - TESAESI (eCRF).
  - TEAEs related to study drug.
    - Related TESAE.
      - Related TESAEs leading to death.
      - Related TESAEs of drug-induced liver toxicity (Hy's Law).
    - Related TESAEs leading to premature study discontinuation.
    - Related TESAEs leading to study drug discontinuation.
    - Grade 3 (severe) or grade 4 (life-threatening) related TEAEs.
    - Related TEAESI (eCRF).

- Relationship to study drug (OZ439/PQP) is to be judged by the Investigator as:
  - Definitely related.
  - Probably related.
  - Possibly related.
  - Not related.

- For presentation purposes related to study drug includes:
  - Definitely related.
  - Probably related.
  - Possibly related.
  - Or missing relationship.

- Serious adverse events include the following (refer to Section 7.16.5 Serious Adverse Events of the protocol):
  - Results in death.
  - Is life-threatening.
  - Requires initial or prolonged hospitalisation.
  - Results in persistent or significant disability/incapacity.
  - Is a congenital anomaly/birth defect.

Copyright © 2012 Quintiles Transnational Corp. All rights reserved.
The contents of this document are confidential and proprietary to Quintiles Transnational Corp. Unauthorized use, disclosure or reproduction is strictly prohibited.

- Medical judgement should be exercised.
- Suspected case of drug-induced liver toxicity (Hy's Law).

- Adverse event reporting, including an assessment of severity grade using the general categorical descriptors outlined below:
  - Attachment 1: Toxicity Grading Scales for Determining Severity of Adverse Events.
  - For abnormalities NOT found elsewhere in the toxicity tables the scale below is to be used to estimate grade of severity:

Table 4: Toxicity Scale

GRADE 1	Mild	Transient or mild discomfort (<48 hours); no medical intervention/treatment required.
GRADE 2	Moderate	Mild to moderate limitation in activity; some assistance may be needed; no or minimal medical intervention/treatment required.
GRADE 3	Severe	Marked limitation in activity; some assistance usually required; medical intervention/treatment required; hospitalizations possible.
GRADE 4	Life threatening	Extreme limitation in activity; significant assistance required; significant medical intervention/treatment required; hospitalization or hospice care probable.

Missing severity is to be seen as worst-case and thus grade 4, life-threatening.

- Adverse events of special interest include:
  - Certain AEs should be considered Adverse Events of Special Interest (AESI) and should be submitted to Quintiles Lifecycle Safety with 24 hours. If these are also SAEs, the SAE form should be used for notification (refer to Section 7.16.5 Serious Adverse Events of the protocol for more detail):
    - Hepatic.
    - Cardiac.
    - Haematological.
    - Pregnancy.

- In addition to the overview of TEAEs, the following summaries are to be presented by SOC and PT:
  - Incidence of TEAEs.
  - Incidence of TESAEs.
  - Incidence of TESAEs leading to death.
  - Incidence of TEAEs leading to premature study discontinuation.
  - Incidence of TEAEs related to study drug.
  - Incidence of grade 3 (severe) or grade 4 (life-threatening) TEAE.
  - Incidence of TEAEs of special interest.
  - Incidence of related TEAEs of special interest.

If an AE occurs more than once the patient is to be counted once per MedDRA SOC and PT as relevant. Percentage of patients in each category is calculated relative to the total number of patients in the Safety analysis set, implying all patients who received the study drug administration or part thereof. All mentions (events) are to be included in each level of presentation, as relevant.

Copyright © 2012 Quintiles Transnational Corp. All rights reserved.
The contents of this document are confidential and proprietary to Quintiles Transnational Corp. Unauthorized use, disclosure or reproduction is strictly prohibited.

The AEs data are to be presented in the following by-patient listings and include all AEs as reported from the signing of informed consent until the end of the study:

- All AEs: General and dictionary coding.
- Serious AEs (including death).
- Adverse events leading to study drug discontinuation.
- Adverse events leading to premature study discontinuation.
- Adverse events of special interest.

16.1.3. DERIVATIONS

Based on the above assessments and with reference to Section 6: General Analysis Definitions and Statistical Considerations and Section 7: Statistical Considerations, the following variables are to be derived:

- Study day: Calculated relative to start of study drug administration, Day 0.
- Adverse events are derived and allocated to a study period as follows:
  - Pre-treatment adverse event (Screening Period) (P):

    Any AE which started or worsened on or after informed consent and before study drug administration.
  - TEAE (Treatment Period) (T):

    Any AE which started or worsened on or after study drug administration up to and including Day 28.
  - Follow-up adverse event (Follow-up Period) (F):

    Any AE which started or worsened after Day 28.
  - The flags are mutually exclusive, meaning AEs can only be assigned to one period.

Refer to Appendix 2: Study Period Allocation and Date Imputations regarding the handling of the allocation of the study periods and incomplete dates.

Copyright © 2012 Quintiles Transnational Corp. All rights reserved.
The contents of this document are confidential and proprietary to Quintiles Transnational Corp. Unauthorized use, disclosure or reproduction is strictly prohibited.

16.2. LABORATORY EVALUATIONS

Blood samples for haematology, and clinical chemistry and urine samples for urinalysis are to be collected. The Investigator is to review the laboratory report promptly, document this review, and record any clinically relevant changes occurring during the study on the Adverse Events eCRF. The laboratory reports must be filed with the source documents.

- Invalid results at Centre 252:
  - For the period of 05JUN2015 to 27AUG2015, the print out result from study machine was noted with "INVALID RESULT, DO NOT REPORT". Hence, it was found some results were invalid because of the haemoglobin sensor. The samples were however not reran with the other available machine at the centre, which produced valid results.
  - MMV confirmed on 02NOV2015 that these invalid results are to be excluded from the haemoglobin analysis. A list of the invalid results is to be provided to BIOS for exclusion from the analysis.

Quantitative laboratory measurements reported as "< X", i.e. BLQ, or "> X", i.e. above the upper limit of quantification (ULQ), are to be converted to X for the purpose of quantitative summaries, but are to be presented as recorded, i.e. as "< X" or "> X" in the by-patient listings.

All clinical laboratory summaries are based on patients included in the Safety analysis set, implying all patients who received the study drug administration or part thereof.

For list of quantitative clinical chemistry laboratory assessments refer to Appendix 3: Quantitative Safety Laboratory Assessments: Categorisation of the SAP.

For list of qualitative urinalysis (including microscopy), serology and pregnancy (serum/urine) variables and categorisation refer to Appendix 4: Qualitative Urinalysis Assessments: Categorisation of the SAP.

16.2.1. DEFINITIONS

Based on the above assessments and with reference to Section 6: General Analysis Definitions and Statistical Considerations and Section 7: Statistical Considerations, the following variables are to be derived:

- Study day: Calculated relative to start of study drug administration, Day 0.
- Baseline: Defined as the last available assessment (scheduled or unscheduled) before study drug administration.
- Post-baseline: Defined as any assessment (scheduled or unscheduled) obtained after study drug administration.
- Treatment Period: On or after study drug administration up to and including Day 28 (based on nominal visit).
- The following flags are to be derived for laboratory test results obtained during the Treatment Period.
  - * Analysable result: Identifies assessment results per scheduled timepoint up to and including Day 28 (based on nominal visit), excluding invalid data affected by Centre 252's haematology machine to be included in the summary tables.
  - ! Worst post-baseline result: Worst post-baseline Division of Microbiology and Infectious Diseases (DMID) toxicity grade: Assessed up to and including Day 28 (based on nominal visit) (refer to Section 16.2.3 Toxicity Grading Criteria for Laboratory Data).

Copyright © 2012 Quintiles Transnational Corp. All rights reserved.
The contents of this document are confidential and proprietary to Quintiles Transnational Corp. Unauthorized use, disclosure or reproduction is strictly prohibited.

- + Invalid haematology results recorded at Centre 252.

- Markedly abnormal: Derived criteria for laboratory-related adverse events of special interest (refer to Section 16.2.2 Laboratory Reference Ranges and Markedly Abnormal Criteria).
- Incidence calculation: Based on analysable results up to and including Day 28 (based on nominal visit).

The following tables are to be presented for the Safety analysis set:

- Descriptive statistics (observed and change from Baseline) for are to be presented for each quantitative haematology, clinical chemistry and urinalysis laboratory variable at Baseline and at each post-baseline analysis visit.
- Shift in reference range classification at each post-baseline visit within the Treatment Period versus Baseline is to be presented for quantitative haematology and clinical chemistry laboratory assessments categorised (low, normal, high) according to reference range and qualitative urinalysis (negative, positive) laboratory assessments. For the shift the percentage (%) of patients in each category calculated relative to the total number of patients in the relevant analysis set with assessments available at Baseline and the relevant post-baseline visit within the Treatment Period per laboratory variable.
- In addition a shift from Baseline to worst toxicity grade post-baseline visit within the Treatment Period versus Baseline is to be presented. For the incidence of post-baseline worst toxicity grade the percentage (%) of patients in each category calculated relative to the total number of patients in the relevant analysis set with assessments available at Baseline and at least one post-baseline assessment per laboratory variable is to be calculated.
- Post-baseline markedly abnormal criteria for derived criteria for laboratory-related adverse events of special interest (refer to Section 16.2.2 Laboratory Reference Ranges and Markedly Abnormal Criteria) by region and age group. For the incidence of post-baseline markedly abnormal criteria the percentage (%) of patients in each category calculated relative to the total number of patients in the relevant analysis set with at least one post-baseline assessment per laboratory variable is to be calculated.

The following laboratory data are to be presented in by-patient listings for the All Patients Randomised analysis set:

- Quantitative haematology laboratory assessments.
- Quantitative clinical chemistry laboratory assessment.
- Quantitative urinalysis laboratory assessments.
- Qualitative laboratory assessments.

- The following laboratory figures are to be presented for the Safety analysis set:
  - A box-whisker plot is to be produced to show the mean, median, 25th and 75th percentiles, most extreme values within Interquartile range (IQR) and outliers over time for the following laboratory variables:
    - Haemoglobin (g/dL).
    - Neutrophils (/µL).
    - Total bilirubin (µmol/L).
    - Direct bilirubin (µmol/L).
    - Alanine aminotransferase (ALT) (U/L.
    - Aspartate aminotransferase (AST) (U/L).
    - Plasma haptoglobin (g/L).
    - Lactate dehydrogenase (LDH) (U/L).
    - Alkaline phosphatase (ALP).

Copyright © 2012 Quintiles Transnational Corp. All rights reserved.
The contents of this document are confidential and proprietary to Quintiles Transnational Corp. Unauthorized use, disclosure or reproduction is strictly prohibited.

- A mean plot is to be produced to show the mean over time for the following laboratory variables:
  - Haemoglobin (g/dL).
  - Neutrophils (/µL).
  - Total bilirubin (µmol/L).
  - Direct bilirubin (µmol/L).
  - ALT (U/L).
  - AST (U/L).
  - Plasma haptoglobin (g/L).
  - LDH (U/L).
  - ALP (U/L).

The SAS® procedure GPLOT is to be used with the following code:

```
Proc gplot data = DD;

Plot x = MEAN / group = TRT01A;

Run;
```

MEAN = Mean.

TRT01A = Actual treatment arm.

- A Scatter plot of maximum post-baseline total bilirubin (µmol/L) versus maximum post-baseline ALT (U/L).

16.2.2. LABORATORY REFERENCE RANGES AND MARKEDLY ABNORMAL CRITERIA

- Quantitative laboratory measurements are to be compared with the relevant laboratory reference ranges in standard international (SI) units and categorised as:
  - Low: Below the lower limit of the laboratory reference range.
  - Normal: Within the laboratory reference range (upper and lower limit included).
  - High: Above the upper limit of the laboratory reference range.
- Incidence of markedly abnormal criteria are to be derived and presented for the following variables (refer to Section 7.16.4 Adverse events of Special Interest of the Protocol):
  - Haemoglobin (g/dL):
    - < 5 g/dL.
    - Decrease from Baseline > 2 g/dL.
  - Neutrophils (/µL) :
    - < 1000 / µL.
  - Total bilirubin (umol/L) :
    - > 2x ULN, > 2.5x ULN
  - Alanine aminotransferase (ALT)(U/L):
    - > 3x ULN, > 5x ULN.
  - Aspartate aminotransferase (AST) (U/L):
    - > 3x ULN, > 5x ULN.

Copyright © 2012 Quintiles Transnational Corp. All rights reserved.
The contents of this document are confidential and proprietary to Quintiles Transnational Corp. Unauthorized use, disclosure or reproduction is strictly prohibited.

16.2.3. TOXICITY GRADING CRITERIA FOR LABORATORY DATA

- The toxicity grades are to be assigned by the Investigators based on the criteria as specified in Attachment 1: Toxicity Grading Scales for Determining Severity of Adverse Events of the protocol.
- Toxicity grading is based on a clinical judgment and is therefore not assigned for the purpose of analysis but used as reported in the clinical study database.
- A Grade 0 is to be presented in the shift tables and is defined as patients with an assessment result not complying with Grade 1 to 4 of the toxicity criteria for a given laboratory variable.
- The higher the grade number, the worse the grade, i.e. grade 1 is worse than a grade 0. Thus from best to worst grade: Grade 0, Grade 1, Grade 2, Grade 3, Grade 4.

16.3. ECG EVALUATIONS

Electrocardiogram (ECG) data recorded by the Investigator as on the ECG eCRF as well as obtained from the central ECG Reading Centre are to be included in the reporting of this study.

The following electronic ECG parameters are to be reported for this study:

- Parameters:
  - Mean heart rate (bpm).
  - Mean PR (ms).
  - Mean RR (ms).
  - Mean QRS (ms).
  - Mean QT (ms).
  - Mean QTc (ms).
  - Mean QTcB (ms).
  - Mean QTcF (ms).
  - Mean QTcW (ms).

16.3.1. DEFINITIONS

Based on the above assessments and with reference to Section 6: General Analysis Definitions and Statistical Considerations and Section 7: Statistical Considerations, the following variables are to be derived:

- Study day: Calculated relative to start of study drug administration, Day 0.
- Baseline: Defined as the last available assessment (scheduled or unscheduled) before study drug administration.
- Post-baseline: Defined as any assessment (scheduled or unscheduled) obtained after study drug administration.
- Treatment Period: After study drug administration up to and including Day 28 (based on nominal visit).
- The following flags are to be derived for ECG results obtained during Treatment Period:
  - * Analysable result: Identifies assessment results per scheduled timepoint as used in the summary tables up to and including Day 28 (based on nominal visit).

Copyright © 2012 Quintiles Transnational Corp. All rights reserved.
The contents of this document are confidential and proprietary to Quintiles Transnational Corp. Unauthorized use, disclosure or reproduction is strictly prohibited.

  - Triplicate ECG assessments: To be obtained at all scheduled visits except for Screening.
- Summary statistics are based on the average of the available analysable triplicate assessments at each visit during the Treatment Period.
- Markedly abnormal: Derived criteria for adverse events of special interest (refer to Section: 16.3.3: Markedly Abnormal Criteria).
- Incidence calculation: Based on analysable results up to and including Day 28. n = Number of patients with at least one event occurring post-baseline in each category (patients with multiple events in each category are counted only once in each category). r = Total number of patients in the relevant analysis set with at least one post-baseline assessment available. N = Total number of patients in the relevant analysis set. % = Percentage of patients with at least one event occurring post-baseline in each category calculated relative to the total number of patients in the relevant analysis set with at least one post-baseline assessment available.
- Investigator's judgement of clinical significance:
  - Normal.
  - Abnormal, clinically significant.
  - Abnormal not clinically significant.

16.3.2. ANALYSIS METHODS

- Descriptive statistics (observed and change from Baseline) are to be presented for each ECG parameter at Baseline and at each post-baseline analysis visit up to and including Day 7:
  - Quantitative safety ECG assessments (12-lead).
  - For the incidence of post-baseline markedly abnormal criteria the Safety analysis set is to be used and the percentage (%) of patients with at least one event occurring post-baseline in each category relative to the total number of patients in the relevant analysis set, with at least one post-baseline assessment available is to be calculated.
  - Quantitative ECG assessments: Post-baseline markedly abnormal criteria.
  - Shift in of the Investigator's assessment at each post-baseline visit within the Treatment Period versus Baseline is to be presented. For shift the Safety analysis set is to be used and the percentage (%) of patients calculated relative to the total number of patients in the relevant analysis set with assessments available at Baseline and the relevant post-baseline visit.
- The following ECG data are to be presented in by-patient listings for the All Patients Randomised analysis set:
  - Quantitative Safety ECG Assessments (12-lead), including change from Baseline and derived markedly abnormal criterion.
  - Abnormal qualitative safety ECG assessments (12-lead).
- A box-whisker plot is to be produced to show the mean, median, 25th and 75th percentiles, most extreme values within IQR and outliers over time for the following ECG parameters:
  - QTcB interval Bazett's correction (QTcF) (ms).
  - QTcF interval Fridericia's correction (QTcF) (ms).
  - QTcW interval Wernicke's correction QTcW (ms).
  - PR (ms).

Copyright © 2012 Quintiles Transnational Corp. All rights reserved.
The contents of this document are confidential and proprietary to Quintiles Transnational Corp. Unauthorized use, disclosure or reproduction is strictly prohibited.

- A mean plot is to be produced to show the mean over time for the following ECG parameters:
  - QTcB (ms).
  - QTcF (ms).
  - QTcW (ms).
  - PR (ms).
- The SAS® procedure GPLOT is to be used with the following code:

Proc gplot data = DD;

Plot x = MEAN / group = TRT01A;

Run;

MEAN = Mean.

TRT01A = Actual treatment arm.

16.3.3. Markedly Abnormal Criteria

The following abnormal electrocardiogram (ECG) criteria have been predefined as:

- Markedly abnormal criteria to be presented for the following variables (refer to Section 7.16.4 Adverse events of Special Interest of the protocol):
  - QTcF prolongation change from Baseline of >60 ms.
  - QTcF at any time >450 ms ().
  - T wave liability, or T wave morphologic changes during therapy.
  - Bundle branch block.
  - Any arrhythmia.

- Mean QTcB (ms).
  - Markedly abnormal criteria:
    - QTcB > 450 ms, > 480 ms and > 500 ms.
    - Increase from Baseline in QTcB > 30 ms to ≤ 60 ms.
    - Increase from Baseline in QTcB > 60 ms.
- Mean QTcF (ms).
  - Markedly abnormal criteria:
    - QTcF > 450 ms, > 480 ms and > 500 ms.
    - Increase from Baseline in QTcF > 30 ms to ≤ 60 ms.
    - Increase from Baseline in QTcF > 60 ms.
- Mean QTcW (ms).
  - Markedly abnormal criteria:
    - QTcW > 450 ms, > 480 ms and > 500 ms.
    - Increase from Baseline in QTcW > 30 ms to ≤ 60 ms.
    - Increase from Baseline in QTcW > 60 ms.

Copyright © 2012 Quintiles Transnational Corp. All rights reserved.
The contents of this document are confidential and proprietary to Quintiles Transnational Corp. Unauthorized use, disclosure or reproduction is strictly prohibited.

16.4. VITAL SIGNS

Blood pressure and pulse rate assessments should be preceded by at least 10 minutes of rest in a supine position.

All vital signs summaries are based on patients included in the Safety analysis set, implying all patients who received the study drug administration or part thereof.

The following vital signs variables are to be analysed:

- Blood pressure (mmHg):
    - Systolic blood pressure (mmHg).
    - Diastolic blood pressure (mmHg).
- Pulse rate (bpm).

16.4.1. DEFINITIONS

Based on the above assessments and with reference to Section 6: General Analysis Definitions and Statistical Considerations and Section 7: Statistical Considerations, the following variables are to be derived:

- Study day: Calculated relative to start of study drug administration, Day 0.
- Baseline: Defined as the last available assessment (scheduled or unscheduled) before study drug administration.
- Post-baseline: Defined as any assessment (scheduled or unscheduled) obtained after study drug administration.
- Treatment Period: After study drug administration up to and including Day 28 (based on nominal visit).
- The following flag is to be derived for ECG results obtained during Treatment Period:
    - * Analysable result: Identifies assessment results per scheduled timepoint as used in the summary tables up to and including Day 28 (based on nominal visit).

16.4.2. ANALYSIS METHODS

- Descriptive statistics (observed and change from Baseline) are to be presented for each vital signs variable at Baseline and at each post-baseline analysis visit:
    - Quantitative safety vital signs assessments.

The following vital signs listing is to be presented for the All Patients Randomised analysis set:

- Quantitative safety vital signs assessments (including change from Baseline).

Copyright © 2012 Quintiles Transnational Corp. All rights reserved.
The contents of this document are confidential and proprietary to Quintiles Transnational Corp. Unauthorized use, disclosure or reproduction is strictly prohibited.

16.5. Physical Examination

To evaluate the patient's eligibility, a complete physical examination is to be performed at Screening. In addition, a targeted physical examination is to be performed at several timepoints throughout the study.

Physical examination assessments as recorded on the Physical Examination eCRF.

The following physical examination listing is to be presented for the All Patients Randomised analysis set:

- Abnormal physical examination assessments.

16.5.1. Physical Examination Specific Derivations

Based on the above assessments and with reference to Section 6: General Analysis Definitions and Statistical Considerations and Section 7: Statistical Considerations, the following variable is to be derived:

- Study day: Calculated relative to start of study drug administration, Day 0.

Copyright © 2012 Quintiles Transnational Corp. All rights reserved.
The contents of this document are confidential and proprietary to Quintiles Transnational Corp. Unauthorized use, disclosure or reproduction is strictly prohibited.

17. REFERENCES

MMV_OZ439_13_003 Study Protocol Version Amendment 4.0, dated 17JUL2015.

MMV_OZ439_13_003 Electronic Case Report Form Version 5.0, dated 24JUL2014.

OZ439_PQP Phase IIb ISMB Team Charter v1.2_20May15.

MMV_OZ439_13_003 Blind Data Review Plan Version 1.0, dated 07AUG2015.

MMV_OZ439_13_003 Interim Statistical Analysis Plan Version 4.0, dated 04AUG2015.

World Wide Antimalarial Resistance Network's (WWARN) calculation. www.wwarn.org/tools-resources/toolkit/.../parasite-clearance-estimator.

Copyright © 2012 Quintiles Transnational Corp. All rights reserved.
The contents of this document are confidential and proprietary to Quintiles Transnational Corp. Unauthorized use, disclosure or reproduction is strictly prohibited.

APPENDIX 1. REQUIREMENTS AND SPECIFICATIONS FOR PROGRAMMING AND PRESENTATION OF TABLES, LISTINGS AND FIGURES (TLFS)

The following details the requirements and specifications for the programming and presentation of the tables, listings and figures (TLFs) as set out in the TLF shells document. The requirements and specifications are a prerequisite for ensuring the inclusion of the TLFs in the integrated clinical study report (CSR) to be produced by Quintiles Medical Writing:

- Each TLF is to be produced as a separate stand-alone document. Also, if separate presentations are required for analysis sets then these should be presented as stand-alone.
- All spelling is to adhere to English (U.K.).
- Depending on data available, dates and times are to take the form DDMMMYYYY/HH:MM.
- All TLFs are to be presented in a landscape format, as far as possible, in order to facilitate the appendices of the CSR.
- The margin, page size and line size specifications stipulated below is to be used for the presentation of all TLFs:

	**Landscape**	**Portrait**
Paper Size	A4 (8.3x11.7 inch)	A4 (8.3x11.7 inch)
Margins (Inches):<br>Top<br>Bottom<br>Left<br>Right	 1.25<br>1<br>1<br>1	 1<br>1<br>1.25<br>1
Header (Inches)	1.25	0.5
Footers (Inches)	0.75	0.5
SAS® Specifications<br>PAGESIZE<br>LINESIZE	 48<br>144	 63<br>89

- The standard font size and font type is ‘8 point’, ‘Courier New’ for all TLFs.
- The content of all tables and listings as available in the raw database is to be uppercase (excluding titles, column headers and footnotes).
- Each TLF is to include a header indicating the Customer name (that is Medicines for Malaria Venture) and the protocol number (that is Protocol Number: OZ439_13_003).
- Each TLF is to contain a footer indicating the program name, Quintiles QID and the date and time when the output was produced.
- Each page of each TLF is to contain a page number written in the format ‘Page N of M’.

Copyright © 2012 Quintiles Transnational Corp. All rights reserved.
The contents of this document are confidential and proprietary to Quintiles Transnational Corp. Unauthorized use, disclosure or reproduction is strictly prohibited.

- Each TLF is to be identified by at least three titles. The first title is to contain the TLF number. The second title is to contain the TLF description. The third title to appear on a given TLF is a description of the analysis set or subset of patients used for the particular presentation of the data.
- Each TLF with a by-group presentation is to include a fourth title with the by-group specification, i.e. the category of the laboratory tests ('Haematology', 'Clinical Chemistry', etc.).
- The following treatment arm labels are to be used for all TLFs in the following order:

Treatment Arm	For Tables	Graphs	For Listings
OZ439 800 mg: PQP 640 mg	800: 640	640	OZ439 800 mg: PQP 640 mg
OZ439 800 mg: PQP 960 mg	800: 640	960	OZ439 800 mg: PQP 960 mg
OZ439 800 mg: PQP 1440 mg	800: 640	1440	OZ439 800 mg: PQP 1440 mg
Total	Total		Total

- Footnotes are to flow continuously instead of each starting on a new line. Abbreviations are to be listed first and separated by a full stop. Individual footnotes are to end with a full stop. The logical ordering of the footnotes are as follows:
  - Abbreviations, separated by a full stop.
  - n = Number of patients... N = Total number of patients... % = Percentage of patients... etc.
  - E = Number of events ... R = Number at risk... etc.
  - Definitions.
  - Footnotes pertaining to statistical methodology.
  - If coding is presented, the version of the coding dictionary used.
  - Any table-specific footnotes that need to be added to clarify data points within the specific presentation.
  - Any footnotes added to a TLF to explain the TLF contents, are to be left aligned and presented as set out in the TLF shells.
- All computed percentages (%) are to be presented using one decimal place.
- All values are to be rounded using the SAS® function ROUND, as the last step prior to presentation.
- If the original data has N decimal places (as derived from the raw data), then the summary statistics are to contain the following number of decimal places (with a maximum of 3 decimal places):
  - The minimum and maximum: N.
  - The mean and median: (N+1).
  - The standard error and standard deviation: (N+2).
  - Note: The TLF shells depicting the number of decimal places for each TLF are only generic examples and the decimal precision (N) must be derived from the raw data.

Copyright © 2012 Quintiles Transnational Corp. All rights reserved.
The contents of this document are confidential and proprietary to Quintiles Transnational Corp. Unauthorized use, disclosure or reproduction is strictly prohibited.

APPENDIX 2. STUDY PERIOD ALLOCATION AND DATE IMPUTATIONS

Allocation to the Study Periods and Date Imputation of Adverse Events of Interest

Adverse events (AEs) present are allocated to study periods based on their start date. If the start date of an event falls on or after the start date but before the stop date of a study period, the AE is attributed to that study period.

Incomplete dates are to be handled as follows:

Partial start or stop dates:

- The events are allocated to the study periods using the available partial information on start and stop date, no imputation is to be done. If, for instance, for the AE start date only month and year are available, these data are compared with the month and year information of the study periods.

Completely missing start date:

- The event is allocated to the Treatment Period, except if the end date of the AE falls before the start of the Treatment Period then it is assigned to the Screening Period.

.

Copyright © 2012 Quintiles Transnational Corp. All rights reserved.
The contents of this document are confidential and proprietary to Quintiles Transnational Corp. Unauthorized use, disclosure or reproduction is strictly prohibited.

APPENDIX 3. QUANTITATIVE SAFETY LABORATORY ASSESSMENTS

Quantitative safety laboratory assessments are to be presented in the following order and in the units specified:

System	Variable	Unit
Haematology	Haematocrit	V/V
	Haemoglobin	g/dL
	Absolute reticulocytes	cells/µL
	Erythrocytes/Red blood cells (RBC)	/µL
	Platelets	/µL
	Leucocytes/White blood cells (WBC)	/µL
	White blood cell differentials:	
	Lymphocytes	/µL and %
	Monocytes	/µL and %
	Eosinophils	/µL and %
	Basophils	/µL and %
	Neutrophils	/µL and %
Clinical Chemistry	Total bilirubin	µmol/L
	Direct bilirubin	µmol/L
	Albumin	g/L
	Alanine aminotransferase (ALT)	U/L
	Aspartate aminotransferase (AST)	U/L
	*Plasma haptoglobin	g/L
	Lactate dehydrogenase (LDH)	U/L
	Creatine kinase (CK)	U/L
	Alkaline phosphatase (ALP)	U/L
	Urea (BUN)	mmol/L
	Creatinine	µmol/L
	Sodium	mmol/L
	Potassium	mmol/L
	Glucose	mmol/L
	Magnesium	mmol/L
	Calcium	mmol/L
Urinalysis	Specific gravity	NA
	pH	NA

ALP: Alkaline phosphatase (ALP). ALT: Alanine aminotransferase. AST: Aspartate aminotransferase. CK: Creatine kinase. LDH : Lactate dehydrogenase. RBC Red blood cells. WBC: White blood cells.

Copyright © 2012 Quintiles Transnational Corp. All rights reserved.
The contents of this document are confidential and proprietary to Quintiles Transnational Corp. Unauthorized use, disclosure or reproduction is strictly prohibited.

APPENDIX 4. QUALITATIVE LABORATORY ASSESSMENTS: CATEGORISATION

Qualitative safety laboratory assessments are to be presented in the following order:

System	Urinalysis	Result	Category
Urinalysis	Glucose	Negative, 0, trace, positive, 1+, 2+, 3+, etc.	NEGATIVE: Negative, 0, trace<br>POSITIVE: Positive, 1+, 2+, 3+, etc.
	Protein	Negative, 0, trace, positive, 1+, 2+, 3+, etc.	NEGATIVE: Negative, 0, trace<br>POSITIVE: Positive, 1+, 2+, 3+, etc.
	Bilirubin	Negative, 0, trace, positive, 1+, 2+, 3+, etc.	NEGATIVE: Negative, 0, trace<br>POSITIVE: Positive, 1+, 2+, 3+, etc.
	Ketones	Negative, 0, trace, positive, 1+, 2+, 3+, etc.	NEGATIVE: Negative, 0, trace<br>POSITIVE: Positive, 1+, 2+, 3+, etc.
	Leukocytes	Negative, 0, trace, positive, 1+, 2+, 3+, etc.	NEGATIVE: Negative, 0, trace<br>POSITIVE: Positive, 1+, 2+, 3+, etc.
	Blood	Negative, 0, trace, positive, 1+, 2+, 3+, etc.	NEGATIVE: Negative, 0, trace<br>POSITIVE: Positive, 1+, 2+, 3+, etc.
Urinalysis *(Microscopy)	Casts*	To be presented in a by-patient listing as reported.	
	White blood cells		
	Red blood cells		
Serology	CMV IgM	Negative, Indeterminate, positive	NEGATIVE<br>POSITIVE
	EBV VCA Igm	Negative, positive	NEGATIVE<br>POSITIVE
	HBsAg	Negative, positive	NEGATIVE<br>POSITIVE
	Hepatitis E IgM	Negative, positive	NEGATIVE<br>POSITIVE
	Anti-HAV IgM	Negative, positive	NEGATIVE<br>POSITIVE
	Anti-HBc IgM	Negative, positive	NEGATIVE<br>POSITIVE
Pregnancy	Pregnancy (urine /serum)	Negative, positive	NEGATIVE<br>POSITIVE

*: Qualitative safety laboratory assessments not routinely assessed at all visits, are only to be listed. Microscopy (RBC: Red blood cells; WBC: White blood cells).

If the dipstick result is positive for protein (greater than trace) leukocytes and/or blood, the sample is to be sent for microscopic analysis of WBC and RBC. The results of this examination are only to be presented in by-patient listings, where applicable.

Copyright © 2012 Quintiles Transnational Corp. All rights reserved.
The contents of this document are confidential and proprietary to Quintiles Transnational Corp. Unauthorized use, disclosure or reproduction is strictly prohibited.
