# Supplementary material for: A randomised, double-blind clinical phase II trial of the efficacy, safety, tolerability and pharmacokinetics of a single dose combination treatment with artefenomel and piperaquine in adults and children with uncomplicated Plasmodium falciparum malaria
Source: BMC Med. 2017 Oct 9;15:181. doi: 10.1186/s12916-017-0940-3 (PMC5632828; doi:10.1186/s12916-017-0940-3)
Supplement: Supplementary file 3 — Pharmacokinetic analysis details. (DOCX 714 kb) [file 12916_2017_940_MOESM3_ESM.docx]

**S3 Pharmacokinetic Analysis Details**

Note. Some of the figures and tables refer to “OZ” or “OZ439” which are the same as artefenomel.

**METHODS**

**Deviations from the study protocol**

The protocol specified that the individual exposures (Cmax, Tmax and AUC) of artefenomel and piperaquine would only be estimated for patients >35kg (those that have rich PK sampling) using non-compartmental analysis. The other patients, those with sparse PK samples, would be analysed using non-linear mixed effect analysis. The analysis was changed to one single non-linear mixed effect analysis including all patients, after which the individual estimated PK parameters were used to calculate the individual exposures in all patients. The non-linear mixed effect analysis allowed the estimation of the exposures, and notably Cday7, for all patients.

**Bio-analysis**

For the study (MMV_OZ439_13_003), a total of 3508 human plasma samples were analysed for artefenomel and piperaquine by LC MS/MS. The limit of quantification for both drugs was 1.00ng/mL. The descriptive statistics of the quality control samples (QC) for artefenomel showed that the inter batch precision was between 4.8 % and 7.3 % whereas the inter batch accuracy was in the range from 97.5 % to 99.0 % of nominal concentration. The descriptive statistics of the QC for piperaquine showed that the inter‑batch precision was between 4.5 % and 6.2 % whereas the inter‑batch accuracy was in the range from 93.5 % to 95.3 % of nominal concentration

**General population PK methods**

The non-linear mixed effect analyses were performed using the non-linear mixed effect modeling approach as implemented in either NONMEM (version 7.3 or later, ICON Development Solutions, Hanover, MD, USA, running under PsN (Perl-speaks-NONMEM) 3.7.6 or later on a grid of CentOS linux servers, and the intel Fortran compiler, version 12) or Monolix (version 4.3.3, Sep 2014) software. Data manipulation, additional calculations and graphics were performed using R (version 3.2.2 GUI 1.66 Mavericks build (6996), The R Foundation for Statistical Computing, 2014).

The selection of the structural models and residual error model was based on scientific judgment, the goodness-of-fit plots and the difference in objective function (approximately -2 x log likelihood) between hierarchical models (i.e. the likelihood ratio test) as well as the Akaike Information Criterion (AIC).

The models for artefenomel and piperaquine were developed separately.

Covariate analysis: *A priori*, the PK models assumed an allometric relationship between body weight and all clearance and volume parameters, and a covariate effect of vomiting (yes or no).

The individual-specific random effect estimates (η) for each parameter were plotted against the pre-selected variables to detect trends in the relationship between structural parameters and covariates. The addition of each covariate on the structural model parameter was compared to model without covariates (but including bodyweight and vomiting), using the Likelihood ratio and Wald test. At a significance level of 0.05, as determined by the chi-square distribution approximation, a change in objective function (OF) of at least 3.841 is required to select one additional parameter in a hierarchical model. All selected covariates were included to construct a full model from which a new value of objective function was obtained and used as a reference. From this full model, the final model was obtained after backward deletion of covariates one by one, starting from the weakest, until no covariate could be removed without a significant increase of the objective function (e.g. for p<0.005, an increase by at least 7.9).

**Artefenomel PK Analysis**

The pharmacokinetics of artefenomel were analysed using a non-linear mixed effect modeling approach using Monolix. Additional data from two monotherapy clinical phase II studies in adult Asian patients (MMV_OZ439_10_002 and MMV_OZ439_12_006 was included in order to extend the dose range (100mg – 1200mg).

**Table 1** Summary of the final PK analysis dataset for artefenomel

| **Study** | **MMV_OZ439­_10_002** | **MMV_OZ439_12_006** | **MMV_OZ439_13_003** | **Total** |
| --- | --- | --- | --- | --- |
| Type | Phase IIa | Phase IIa | Phase IIb |  |
| Dosing | 200, 400, 800, 1200 mg single dose | 100, 500 mg single dose | 800 mg OZ with either 640, 960, 1440 mg PQP single dose |  |
| Administration | PIB +milk | PIB +milk | TPGS fasted |  |
| Number of PK samples | rich; 0.5 hr post dose - 7 days | rich; 0.5 hr post dose - 28 days | 15-16 in adults, 3-10 in children 2 hr  post dose - 63 days |  |
| Total Conc Records | 1297 | 305 | 3311 | 4913 |
| Conc Records BQL | 37 (3%) | 42 (14%) | 797 (24%) | 876 (18%) |
| Dose Records | 81 | 22 | 427 | 530 |
| Population | Patients | Patients | Patients |  |
| n | 81 | 22 | 427 | 530 |
| Male/Female | 70/11 | 18/4 | 260/167 | 348/182 |
| Africa/Asia | 0/81 | 0/22 | 345/82 | 345/185 |
| Age^a^ (yrs) | 26 (18-60) | 37 (18-52) | 3 (0.5-60) | 4 (0.5-60) |
| Africa ≤5 years/ Africa > 5 years/ Asia >5 years | 0/0/81 | 0/0/22 | 281/64/82 | 281/64/185 |
| Body weight^a^ (kg) | 52 (42-89) | 54 (48-64) | 14.5 (5.6-85) | 17.5 (5.6-85) |
| Vomiters | 6 (7%) | 2 (9%) | 113 (26%) | 121 (23%) |
| Time of Vomiting^a^ (hr) | 3.9 (0.8-135) | 8.5 (5.4-11.5) | 0.47 (0.08-2.45) | 0.5 (0.08-135) |
| *P.f*/*P.v*/mixed^b^ | 40/40/1 | 22/0/0 | 427/0/0 | 489/40/1 |

^a^median (range)

*^b^P. falciparum (P.f) and P. vivax (P.v)*

Observations below the quantification limit (BQL) were included in the dataset. Monolix handles these (left-censored data) by including the simulation of the censored data with a truncated Gaussian distribution in the Markov Chain Monte Carlo (MCMC) procedure.

The pharmacokinetics of artefenomel in adult- and pediatric malaria patients could be described by a three-compartment disposition model with first-order absorption and a lag-time.

The following model elements were considered:

- Only first order absorption with lag time was evaluated. This was based on the previous modeling of artefenomel PK and the fact that the new data (study MMV_OZ439_13_003) did not include any time points before 2 hours post-dose. In addition, the model adequately described the absorption phase.
- The effect of vomiting on the relative bioavailability and its between subject variability was included from the beginning and were significant. The relative bioavailability of vomiters was 50% of non-vomiters, with slightly increased between subject variability (CV 86 vs 62%)
- Despite the considerable number of patients with only sparse sampling, a 3-compartment model provided a considerable better fit.
- Body weight was included allometrically on all disposition parameters using the theoretical exponents (0.75 for clearances and 1 for volumes). No residual body weight related effects remained after using these theoretical exponents. Furthermore when estimating the exponents for the parameters for which reasonable information was available (considering the sparse sampling in the children), such as the Cl/F, V1/F and V3/F, these estimates were not far from the theoretical values and improved the fit only modestly. The body weight was normalised to 50 kg.
- Between subject variability on tlag improved the model considerably.
- Between subject variability on V1 and Q2 was not supported: probably the result of the sparse sampling in a large proportion of the patients.
- Changing the residual error model to proportional + additive did not improve the fit, and the resulting value for the additive error was small.

The following covariates were identified:

- The relative bioavailability (F) was a function of age: 40% lower for a 1 year vs a 20 year old patient.
- The absorption rate constant decreased with the increasing actual administered dose: Ka was 2x at 100mg vs 800mg.
- Non-linear PK: Clearance decreased with increasing artefenomel dose group: Cl/F was 2.2 fold greater at 100mg vs 800mg

No covariate effects of study, region, sex, infection (*P. falciparum* (*P.f*) vs *P. vivax (P.v))*, protocol defined non-compliance, PQP dose, or formulation (in studies MMV_ OZ439_10_002 and MMV_ OZ439_12_006 artefenomel was dosed using different formulation to MMV_OZ439_13_003) were identified.

For the final model no remaining body weight or age effects can be observed (see figure 1).

**Figure 1** Random effects versus normalized bodyweight (tBW) and age (tAGE)


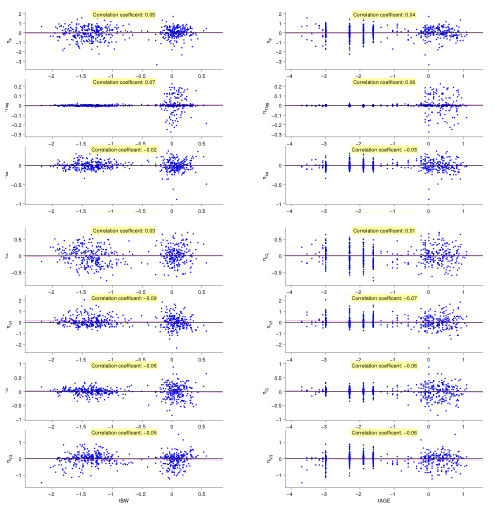


The final artefenomel popPK model is summarised in table 2.

**Table 2** Parameter estimates artefenomel population PK model in Patients

| **Parameter** |  | **Estimate^a^** | **BSV^b^ (%)** |
| --- | --- | --- | --- |
| F | Relative Oral Bioavailability | $1*\left(\frac{AGE}{20}\right)^{0.19(12)}$ | 62 (4) |
| Fvom | Relative Oral Bioavailability in Vomiters | $0.51(9)*\left(\frac{AGE}{20}\right)^{0.19(12)}$ | 86 (8) |
| tlag (hr) | Absorption lag time | 0.41 (1) | 14 (8) |
| ka (1/hr) | Absorption rate constant | $0.17(2)*\left(\frac{ODOS}{800}\right)^{-0.34(8)}$ | 22 (8) |
| Cl/F (l/hr) | Apparent Clearance | $49.2(2)*\left(\frac{BW}{50}\right)^{0.75}\left(\frac{ODGP}{800}\right)^{-0.37(11)}$ | 33 (5) |
| V1/F (l) | Apparent central volume of distribution | $135(5)*\left(\frac{BW}{50}\right)^{1}$ | 73 (6) |
| Q/F (l/hr) | Apparent inter compartmental Clearance 1 | $9.7(4)*\left(\frac{BW}{50}\right)^{0.75}$ | 36 (8) |
| V2/F (l/hr) | Apparent peripheral volume of distribution 1 | $269(5)*\left(\frac{BW}{50}\right)^{1}$ | - |
| Q2 (l/hr) | Apparent inter compartmental Clearance 2 | $7.0(3)*\left(\frac{BW}{50}\right)^{0.75}$ | - |
| V3 (l) | Apparent peripheral volume of distribution 2 | $1130(4)*\left(\frac{BW}{50}\right)^{1}$ | 51 (2) |
| residual | proportional | 0.26 (2) | - |

^a^Estimate, with between brackets the RSE (Relative Standard Error %)

^b^Between Subject Variability

AGE=age (years); ODOS=actual administered dose (mg);ODGP=adult equivalent dose (mg); BW= body weight (kg)

The observed artefenomel plasma concentrations and summaries with the model prediction interval for study MMV_OZ439_13_003 are shown in figures 2 (all data over first 180 hours) and 3 (by body weight band). The model predicted the distribution of the observed plasma concentrations of artefenomel well, in particular across the body weight bands. Both the median and the 80% interval were well predicted.

The non-linear PK was modeled as a function of dose, rather than concentration (eg Michaelis – Menten elimination), which would be preferred. This approximation was considered adequate, however, considering the main objectives of the popPK analysis: to estimate the individual exposures and to use these to evaluate the exposure – response relationship. For simulations, especially extrapolations, this approximation may be a limitation of the current model. However, the model performed adequately for the dose range included in the analysis (100 – 1200 mg), including the main dose level of interest for single encounter treatment, 800 mg.

**Figure 2** Visual Predictive Check. Observed artefenomel plasma concentrations and their summaries (emp. Prctile) over time with the model prediction interval (P.I) for study MMV_OZ439_13_003: first 180 hours.


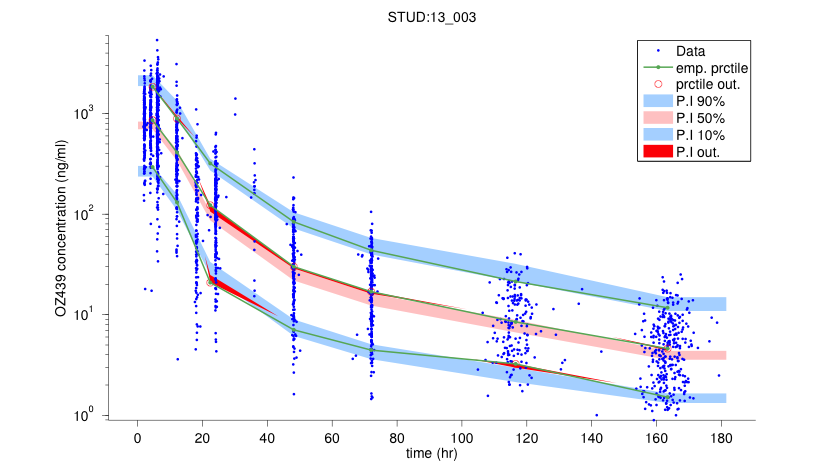


**Figure 3** Visual Predictive Check. Observed artefenomel plasma concentrations (ng/ml) and their summaries (emp. Prctile) over time (hr) with the model prediction interval (P.I) for study MMV_OZ439_13_003: first 180 hours by body weight band.


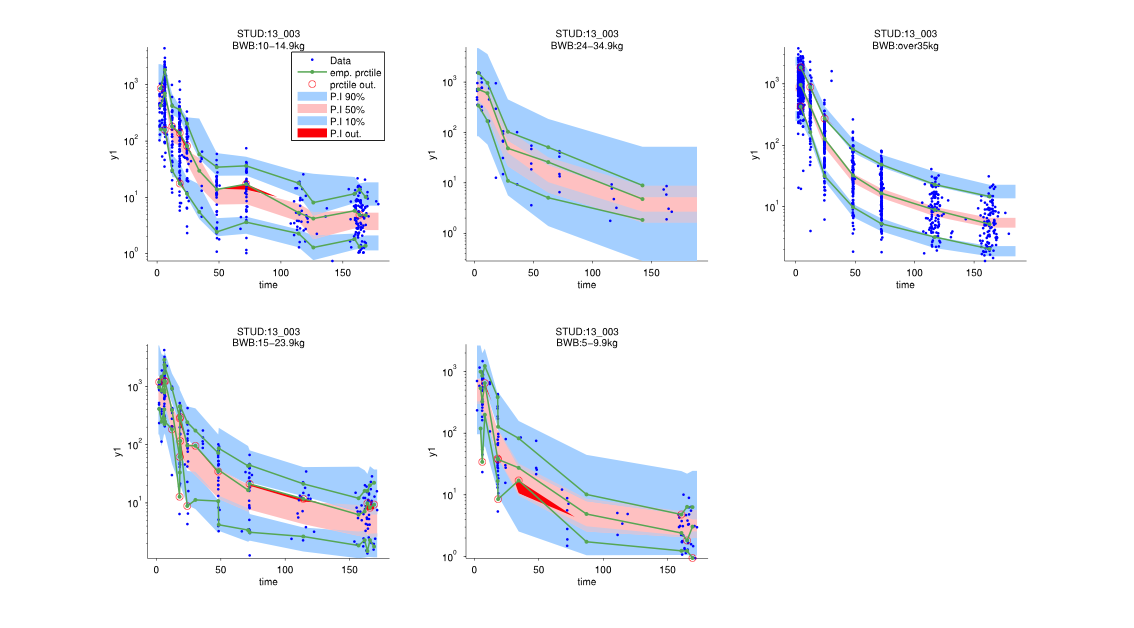


The model predicted the individual concentrations well (not shown), including around day 7 (figure 4).

**Figure 4** Observed vs individual predicted artefenomel concentrations (ng/ml) around day 7 (between 144 and 192 hrs post dose).

**Piperaquine PK Analysis**

The pharmacokinetics of piperaquine were analysed using a non-linear mixed effect modeling approach using NONMEM.

**Table 3** Summary of the final PK analysis dataset for piperaquine

| **Study** | **MMV_OZ439_13_003** |
| --- | --- |
| Type | Phase IIb |
| Dosing | 640, 960, 1440 mg PQP all with 800 mg OZ single dose |
| Administration | TPGS fasted |
| PK samples | 15-16 in adults, sparse (3-10) in children; 2 hr  post dose - 63 days |
| Total Conc Records | 3302 |
| Conc Records BQL | 0 (0%) |
| Dose Records | 426 |
| Population | Patients |
| n | 426 |
| Male/Female | 259/167 |
| Africa/Asia | 344/82 |
| Age^a^ (yrs) | 3 (0.5-60) |
| Africa ≤5 years/ Africa > 5 years/ Asia >5 years | 280/64/82 |
| Body weight^a^ (kg) | 14.5 (5.6-85) |
| Vomiters | 114 (27%) |
| Time of Vomiting^a^ (hrs) | 0.47 (0.08-2.45) |
| *P.f*/*P.v*/mixed^b^ | 426/0/0 |

^a^median (range)

*^b^P. falciparum (P.f) and P. vivax (P.v)*

Observations below the quantification limit (BQL; 5%) were removed from the dataset before analysis. The population PK for piperaquine in malaria patients was developed based on the published model by Tarning *et al*. [1]. The PK could be described by a 3-compartment disposition model that incorporated a fixed (n=3) number of absorption transit compartments.

The initial models developed accounted for weight as a fixed allometric function. Once a tentative base model was developed, the effect of estimating the allometric exponents in comparison to fixing the exponents was evaluated via the relative contribution to the model fit (drop in objective function value (OF)). A relatively large drop in the OF implied that estimating the allometric exponents provided a better description of the data compared to fixing them to their theoretical values.

Whether the patients vomited or not was expected to impact bioavailability (F) and therefore F was fixed to 1 for non-vomiters and estimated for vomiters. The relative bioavailability of vomiters was about 70% of non-vomiters. An effect of vomiting on the between subject variability in relative bioavailability was not evaluated.

Between subject variability (BSV) was initially included on F and Cl, since these key parameters play a role in calculating individual exposure metrics e,g., AUC. A common between subject variability was estimated for both non-vomiters and vomiters. Relative contribution of including between subjects variability on other parameters was sequentially tested. Once BSV was included on F, Cl and V2, only MTT led to a further significant drop in OF. While inclusion of BSV on more than 4 parameters proved computationally problematic, the results suggested that it was unlikely to have any further significant improvement in the model fit.

After including bodyweight and vomiting into the model, no covariate effects of region, age, sex, non-compliance or actual or adult equivalent PQP dose were identified.

The final piperaquine model is summarised in table 4.

**Table 4** Parameter estimates piperaquine population PK model in Patients

| **Parameter** |  | **Estimate^a^** | **BSV^b^ (%)** |
| --- | --- | --- | --- |
| F | Relative Oral Bioavailability | 1 | 74 (8) |
| Fvom | Relative Oral Bioavailability in Vomiters | 0.68 (11) | (74 (8)) |
| MTT (hr) | Mean transit absorption time (3 compartments) | 1.76 (0.001) | 65 (2) |
| Ktr^c^ (1/hr) | Transit absorption rate constant | 2.27 | (65) |
| Cl/F (l/hr) | Apparent Clearance | $151(5)*\left(\frac{BW}{70}\right)^{0.71(11)}$ | 28 (29) |
| V1/F (l) | Apparent central volume of distribution | $9626(11)*\left(\frac{BW}{70}\right)^{0.64(14)}$ | 64 (16) |
| Q/F (l/hr) | Apparent inter compartmental Clearance 1 | $332(6)*\left(\frac{BW}{70}\right)^{0.71(11)}$ | - |
| V2/F (l/hr) | Apparent peripheral volume of distribution 1 | $15770(11)*\left(\frac{BW}{70}\right)^{0.64(14)}$ | - |
| Q2 (l/hr) | Apparent inter compartmental Clearance 2 | $172(6)*\left(\frac{BW}{70}\right)^{0.71(11)}$ | - |
| V3 (l) | Apparent peripheral volume of distribution 2 | $51300(11)*\left(\frac{BW}{70}\right)^{0.64(14)}$ | - |
| residual | Additional (log domain) | 0.45 (0.1) | - |

^a^Estimate, with between brackets the RSE (Relative Standard Error %)

^b^Between Subject Variability

^c^Ktr=4/MTT; ka=Ktr

BW= body weight (kg)

A summary of the observed piperaquine plasma concentrations with the model prediction for MMV_OZ439_13 003 is shown in figures 5 (by treatment arm and thus PQP dose) and 6 (by body weight band). The model predicted the distribution of the observed plasma concentrations of piperaquine well, in particular across the body weight bands.

**Figure 5** Visual Predictive Check. Summaries of the observed piperaquine plasma concentrations and their summaries (lines; median and 95% interval) over time with the model prediction interval (shaded areas; median and 95% interval with 95% CI): By treatment arm (adult equivalent PQP dose).


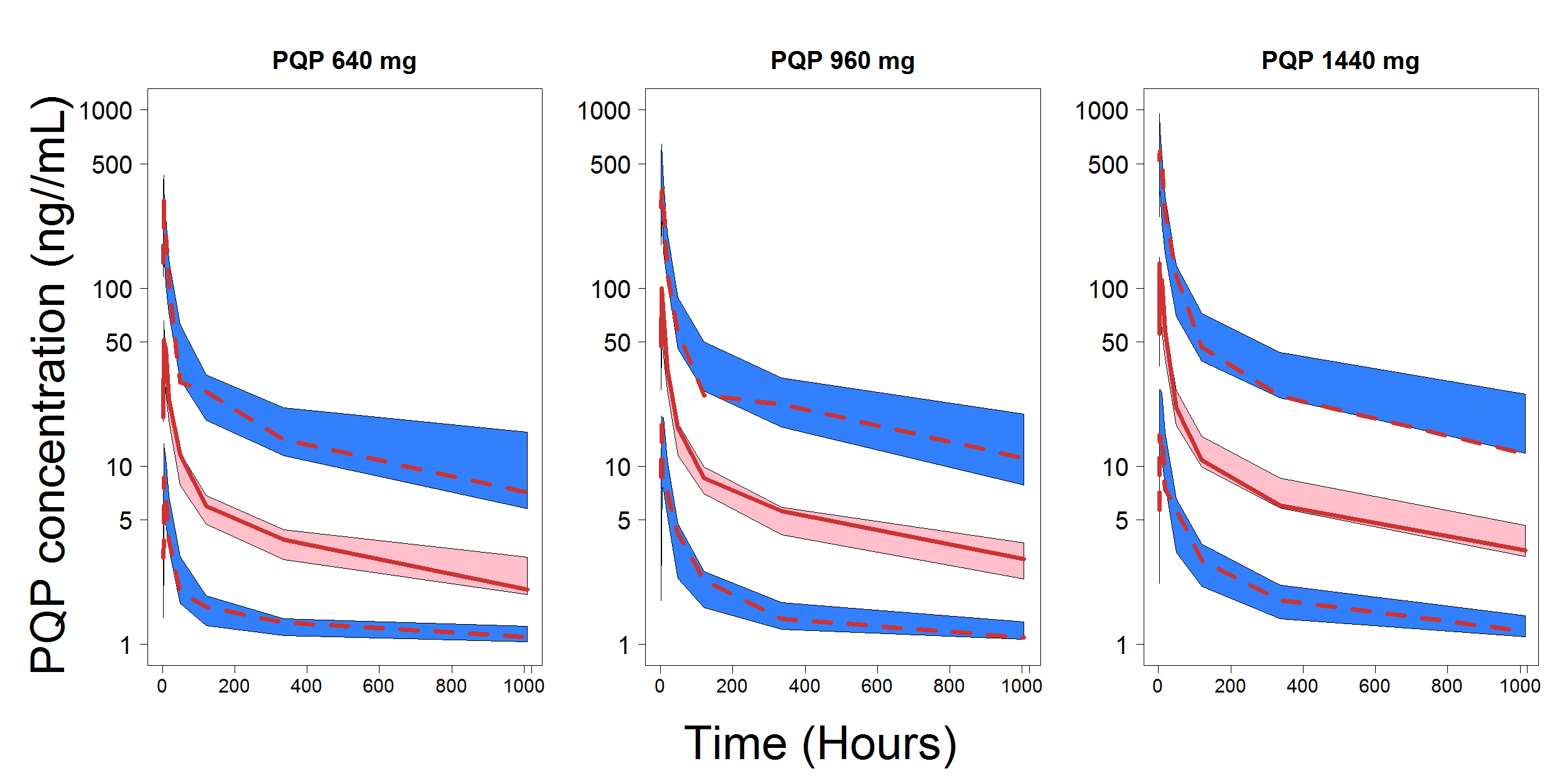


**Figure 6** Visual Predictive Check. Summaries of the observed piperaquine plasma concentrations and their summaries (lines; median and 95% interval) over time with the model prediction interval (shaded areas; median and 95% interval with 95% CI): By body weight band.


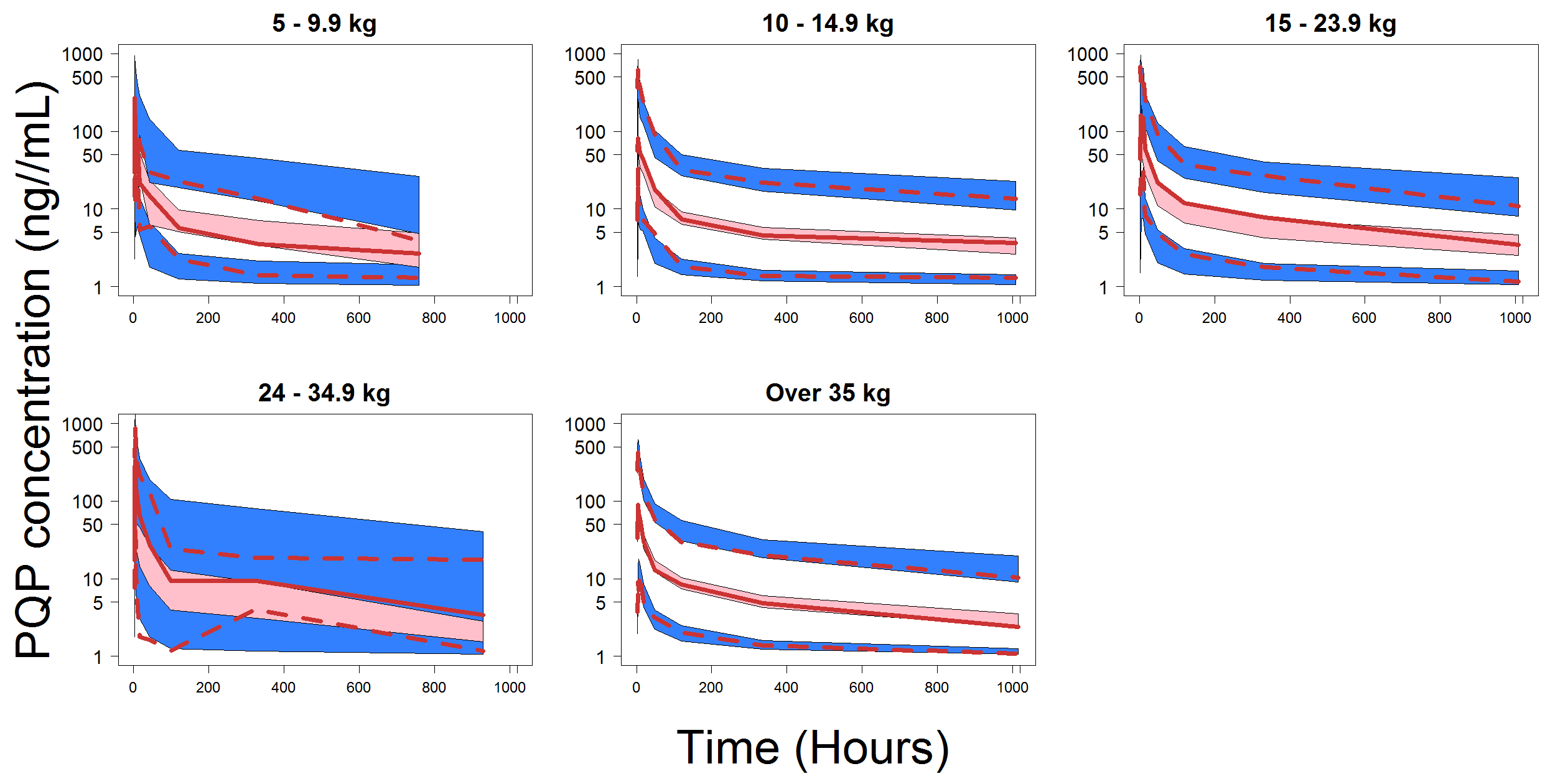


The model predicted the individual concentrations well (not shown), including around day 7 (figure 7).

**Figure 7** Observed vs individual predicted piperaquine concentrations (ng/ml) around day 7 (between 144 and 192 hrs post dose).

**Estimation of the individual plasma exposures**

The exposures of artefenomel and pipaquine for each patient in study MMV_OZ439_13_003 were derived from the individual PK parameters estimated (empirical Bayes estimates) in the population PK analysis. The individual fits were evaluated visually to confirm that the individual plasma profiles were adequately described. Individual concentration time profiles over 672 hrs (28 days) were simulated using the estimated individual model parameters and actual doses. Exposure parameters for each individual subject were then estimated as follows: Cmax, Tmax, Cday7, as observed from the simulated profiles, and AUCinf, calculated as Frel*Dose/(apparent CL).

Tables 5 and 6 summarise the estimated individual exposures for both artefenomel and piperaquine.

**Table 5** Selected summary statistics of the individual artefenomel plasma exposure estimates for study MMV_OZ439_13_003 for various sub-populations: 800 mg artefenomel.

| **Sub group** | **N** | **Cmax^a^** | **AUCinf^a^** | **Cday7^a^** |
| --- | --- | --- | --- | --- |
|  |  | **[ng/ml]** | **[μg*hr/ml]** | **[ng/ml]** |
| All Patients | 427 | 869 (78%) | 10.1 (107%) | 3.0 (147%) |
| **Vomiting Status** | | | | |
| Non-Vomiters | 314 | 1035 (63%) | 12.6 (89%) | 3.7 (130%) |
| Vomiters | 113 | 535 (86%) | 5.4 (105%) | 1.5 (133%) |
| **Treatment Arm** | | | | |
| 800mgOZ 640mgPQP | 142 | 941 (75%) | 11.6 (96%) | 3.5 (133%) |
| 800mgOZ 960mgPQP | 143 | 870 (74%) | 10.0 (106%) | 2.9 (152%) |
| 800mgOZ 1440mgPQP | 142 | 804 (85%) | 8.9 (117%) | 2.6 (153%) |
| **Body Weight Band** | | | | |
| 5-9.9kg | 59 | 748 (65%) | 6.8 (84%) | 1.5 (102%) |
| 10-14.9kg | 170 | 766 (86%) | 8.8 (111%) | 2.6 (153%) |
| 15-23.9kg | 64 | 1195 (84%) | 11.4 (113%) | 3.7 (153%) |
| 24-34.9kg | 7 | 848 (64%) | 11.0 (89%) | 3.6 (117%) |
| Over35kg | 127 | 1042 (70%) | 13.6 (93%) | 4.2 (121%) |
| **Region/Age Band** | | | | |
| Africa ≤ 5yr | 281 | 826 (82%) | 8.7 (108%) | 2.5 (152%) |
| ≥ 0.5 & ≤ 2yr | 161 | 759 (81%) | 7.6 (105%) | 2.0 (147%) |
| > 2 & ≤ 5yr | 120 | 927 (81%) | 10.4 (108%) | 3.3 (142%) |
| Africa > 5yr | 64 | 770 (87%) | 10 (111%) | 3.3 (141%) |
| Asia > 5yr | 82 | 1137 (48%) | 16.9 (66%) | 5.1 (95%) |

^a^Geometric Mean (CV%)

**Table 6** Selected summary statistics of the individual piperaquine plasma exposure estimates for study MMV_OZ439_13_003 for various sub-populations by treatment arm.

| **Treatment Arm** | **Sub group** | **N** | **Cmax^a^** | **AUCinf^a^** | **Cday7^a^** |
| --- | --- | --- | --- | --- | --- |
|  | **Total 426** |  | **[ng/ml]** | **[μg*hr/ml]** | **[ng/ml]** |
| 800mgOZ 640mgPQP | All Patients | 142 | 54 (63%) | 5.2 (79%) | 5.3 (76%) |
| 800mgOZ 960mgPQP | All Patients | 143 | 83 (89%) | 7.2 (84%) | 7.3 (82%) |
| 800mgOZ 1440mgPQP | All Patients | 141 | 125 (126%) | 9.7 (93%) | 9.9 (91%) |
| **Vomiting Status** | | | | | |
| 800mgOZ 640mgPQP | Non-Vomiters | 107 | 62 (9095 | 5.7 (73%) | 5.8 (68%) |
|  | Vomiters | 35 | 36 (122%) | 3.8 (83%) | 3.9 (63%) |
| 800mgOZ 640mgPQP | Non-Vomiters | 107 | 91 (102%) | 7.9 (85%) | 8.0 (71%) |
|  | Vomiters | 36 | 64 (129%) | 5.6 (75%) | 5.6 (113%) |
| 800mgOZ 1440mgPQP | Non-Vomiters | 98 | 135 (125%) | 10.6 (93%) | 10.9 (91%) |
|  | Vomiters | 43 | 105 (133%) | 7.8 (90%) | 7.9 (88%) |
| **Body Weight Band** | | | | | |
| 800mgOZ 640mgPQP | 5-9.9kg | 17 | 42 (70%) | 4.7 (53%) | 4.7 (53%) |
|  | 10-14.9kg | 62 | 59 (93%) | 5.4 (72%) | 5.5 (70%) |
|  | 15-23.9kg | 18 | 67 (91%) | 7.1 (70%) | 7.1 68%) |
|  | 24-34.9kg | 1 | 4 (-) | 1 (-) | 1.1 (-) |
|  | Over35kg | 44 | 51 (129%) | 4.6 (92%) | 4.8 (89%) |
| 800mgOZ 640mgPQP | 5-9.9kg | 23 | 52 (74%) | 4.8 (56%) | 4.9 (54%) |
|  | 10-14.9kg | 51 | 80 (100%) | 7.5 (80%) | 7.6 (76%) |
|  | 15-23.9kg | 24 | 120 (110%) | 10.2 (98%) | 10.3 (96%) |
|  | 24-34.9kg | 3 | 154 (52%) | 11.3 (46%) | 11.2 (44%) |
|  | Over35kg | 42 | 87 (129%) | 6.8 (85%) | 7.1 (83%) |
| 800mgOZ 1440mgPQP | 5-9.9kg | 20 | 63 (95%) | 7.0 (78%) | 7.1 (74%) |
|  | 10-14.9kg | 55 | 145 (127%) | 10.1 (99%) | 10.3 (97%) |
|  | 15-23.9kg | 22 | 172 (124%) | 12.0 (85%) | 12.3 (84%) |
|  | 24-34.9kg | 3 | 306 (40%) | 20.4 (37%) | 21.5 (38%) |
|  | Over35kg | 41 | 112 (121%) | 9.0 (93%) | 9.3 (91%) |
| **Region/Age Band** | | | | | |
| 800mgOZ 640mgPQP | Africa ≤ 5yr | 94 | 57 (90%) | 5.5 (70%) | 5.5 (68%) |
|  | ≥ 0.5 & ≤ 2yr | 51 | 50 (87%) | 4.9 (65%) | 5.0 (63%) |
|  | > 2 & ≤ 5yr | 43 | 66 (90%) | 6.2 (73%) | 6.3 (71%) |
|  | Africa > 5yr | 21 | 46 (178%) | 5.4 (120%) | 5.6 (113%) |
|  | Asia > 5yr | 27 | 51 (114%) | 4.1 (74%) | 4.2 (72%) |
| 800mgOZ 960mgPQP | Africa ≤ 5yr | 94 | 77 (104%) | 7.1 (85%) | 7.2 (82%) |
|  | ≥ 0.5 & ≤ 2yr | 52 | 63 (86%) | 5.8 (73%) | 5.9 (70%) |
|  | > 2 & ≤ 5yr | 42 | 99 (116%) | 9.1 (90%) | 9.2 (87%) |
|  | Africa > 5yr | 22 | 100 (178%) | 8.6 (108%) | 8.8 (105%) |
|  | Asia > 5yr | 28 | 90 (82%) | 6.6 (63%) | 6.9 (61%) |
| 800mgOZ 960mgPQP | Africa ≤ 5yr | 92 | 124 (130%) | 9.5 (95%) | 9.7 (93%) |
|  | ≥ 0.5 & ≤ 2yr | 59 | 110 (135%) | 8.9 (96%) | 9.0 (94%) |
|  | > 2 & ≤ 5yr | 33 | 153 (117%) | 10.7 (93%) | 10.9 (92%) |
|  | Africa > 5yr | 21 | 131 (183%) | 11.7 (103%) | 12.1 (103%) |
|  | Asia > 5yr | 28 | 124 (91%) | 9.0 (80%) | 9.3 (78%) |

^a^Geometric Mean (CV%)

**References**

[1] J. Tarning, P. Thana, A. P. Phyo, K. M. Lwin, W. Hanpithakpong, E. A. Ashley, N. P. J. Day, F. Nosten, and N. J. White. Population pharmacokinetics and antimalarial pharmacodynamics of piperaquine in patients with *plasmodium vivax* malaria in Thailand. CPT Pharmacometrics Syst Pharmacol, 3:e132, 2014.
