# Supplementary material for: A randomised, double-blind clinical phase II trial of the efficacy, safety, tolerability and pharmacokinetics of a single dose combination treatment with artefenomel and piperaquine in adults and children with uncomplicated Plasmodium falciparum malaria
Source: BMC Med. 2017 Oct 9;15:181. doi: 10.1186/s12916-017-0940-3 (PMC5632828; doi:10.1186/s12916-017-0940-3)
Supplement: Supplementary file 4 — Exposure–response analysis details. (DOCX 274 kb) [file 12916_2017_940_MOESM4_ESM.docx]

**S4 Exposure Response Analysis Details**

Note. Some of the figures and tables refer to “OZ” which stands for OZ439 and is the same as artefenomel.

**Exposure Response Analysis**

Examples of the observed concentrations of artefenomel and piperaquine in two individual patients with different ACPR28 outcomes, together with the model fits as well as the time course of the parasitemia are shown in figure 1.

**Figure 1 Example of observed and model-predicted PK profiles for artefenomel and piperaquine with the observed parasitemia over time in two individual patients**

**
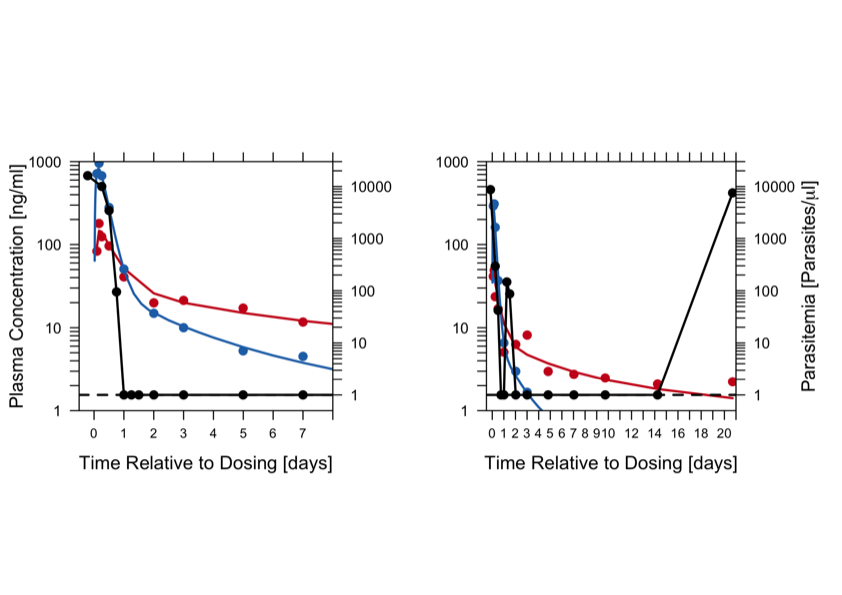
**Artefenomel: blue; Piperaquine: Red; Parasitemia: Black

LEFT) African Female Patient (16yrs, 59kg) receiving 800mg Artefenomel plus 1440mg PQP who achieved ACPR28

RIGHT) African Female Patient (34yrs, 77kg) receiving 800mg Artefenomel plus 960mg PQP who vomited and recrudesced.

The relationship between estimated artefenomel and piperaquine Cday7 and PCR-adjusted ACPR28 (success or failure) was evaluated statistically by logistic regression (statistical software R version 3.2.5). This relationship was clearly present in the observed data as shown in the exploratory plots (figures 6 and 8 in the main publication).

First, a base model that included the effect of artefenomel and piperaquine Cday7 was developed. Univariate effects of either artefenomel or piperaquine day7 were evaluated, relative to an intercept only model. Subsequently, the univariate effects of the other covariates were evaluated (in the presence of both artefenomel and piperaquine Cday7). The significant covariates were retained in the model after which interactions were explored.

The relative contribution of different effects to describing the data was quantified by p-values obtained from the likelihood ratio test (LRT) for nested models. The difference in twice negative loglikelihood (-2LL) values between a null/reduced model (where some parameters have been set to zero) and a full model is approximately chi-square distributed with the number of degrees of freedom being the difference in the number of parameters between the two models. A decrease of 3.84 points in -2LL is statistically significant at the 5% level of significance for one degree of freedom. The Akaike information criteria (AIC) was also used for comparing between models, in particular those that were not nested.

For the factor Kelch13 genotype various groupings were evaluated:

- True Wild Type (WT; no mutation) vs. any mutation (MUT)
- C580Y and I543T mutations (MUT) vs. WT for these 2 alleles: validated markers for partial artemisinin resistance.
- C580Y, I543T as well as P553L and V568G (MUT) vs. WT for these 4 alleles: validated or candidate markers for partial artemisinin resistance.

In addition a sensitivity analysis was performed in which concentration at Day 7 (for both artefenomel and piperaquine) in the final model was replaced with either concentration at Day 14 or concentration at Day 28.

Analysis dataset:

**Table 1.** Exposure - response dataset summary

| **Study** | **MMV_OZ439_13_003** |
| --- | --- |
| Population | Derived from ITT |
| Number of patients | 348 |
| Africa/ Asia | 269/79 |
| Median age, years (range) | 4.2 (0.5–58) |
| Africa ≤5 years/ Africa > 5 years/ Asia >5 years | 211 /58/79 |
| Baseline parasitemia (par/μL) | 11,640 (187–220,200) |
| K13^‡^ genotype WT/MUT/missing | 252/45/51 |
| PCR-adjusted ACPR28 (success/failure) | 254/94 |
| Estimated Cday7 artefenomel (ng/mL) | 3.5 (0.05–21.0) |
| Estimated Cday7 piperaquine (ng/mL) | 7.3 (0–16.2) |

^‡^ K13 genotype: C580Y, 1543T,P553L and V568G (MUT) versus others (WT for these four alleles).

The final model parameters are summarized in Table 2. All parameters except for the additional intercept for Asian patients are reasonably well estimated.

The sensitivity analysis (Cday7 vs. other, potentially relevant exposure variables Cday14 and Cday28) suggested that the model based on Cday7 was a better description of the data. However, the difference between these three models was small.

**Table 2: Parameter estimates for the relationship between Cday7 of artefenomel and piperaquine and ACPR28**

| **Parameter** | **Estimate^a^** | **95% CI** | **p-value** |
| --- | --- | --- | --- |
| Intercept | 3.23 (40) | (0.77 - 5.80) | 0.0115 |
| Slope Cday7 piperaquine (ng/ml) | 0.22 (27) | (0.10 - 0.35) | 0.0005 |
| Slope Cday7 artefenomel (ng/ml) | 0.73 (25) | (0.42 - 1.11) | <0.0001 |
| Slope log10(baseline parasitemia (par/µl)) | -1.27 (24) | (-1.87 - -0.70) | <0.0001 |
| Additional intercept Asia | 0.46 (135) | (-0.79 - 1.66) | 0.4614 |
| Additional slope Cday7 artefenomel Asia | -0.59 (32) | (-0.99 - -0.23) | 0.0021 |

^a^Estimate (Relative Standard Error %)

No significant effect of the factor Kelch13 genotype was identified. The results are summarized in table 3. The results suggest that there may be some effect (WT having a higher probability of ACPR28), but that the sample size was too small to identify it.

**Table 3: Summary of the evaluation of Kelch13 genotype added to a base model with artefenomel and piperaquine Cday7 included (complete case only analysis, n=297).**

| **Factor** | **Estimate (WT)** | **95% CI** | **p-value** |
| --- | --- | --- | --- |
| True Wild Type (WT; no mutation) vs. any mutation (MUT) | 0.78 | (-0.08 – 1.66) | 0.0760 |
| C580Y and I543T mutations (MUT) vs. WT for these 2 alleles | 0.89 | (-0.25 – 2.08) | 0.1298 |
| C580Y, I543T as well as P553L and V568G (MUT) vs. WT for these 4 alleles | 0.88 | (-0.07 – 1.86) | 0.0720 |

The model predictions and observed ACPR28 results for various sub populations of interest are shown in Figure 2. This figure shows that the model predicts the observed pACPR28 well.

**Figure 2. Observed and model predicted p(ACPR28) for the Phase IIb study population with 90%CI in various sub-populations. The simulations were similar to the simulation method described below but based on the actual patient population in each subgroup (i.e. estimated individual Cday7 and covariates in those patients) and summarised across 1000 replicates.**

**
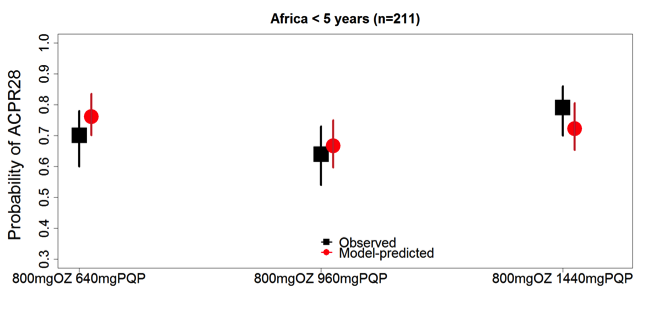
**

**
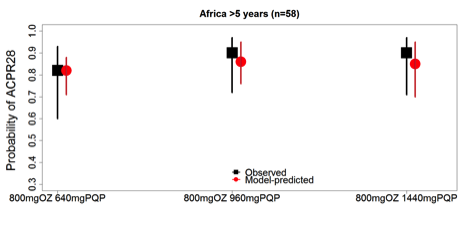
**

**
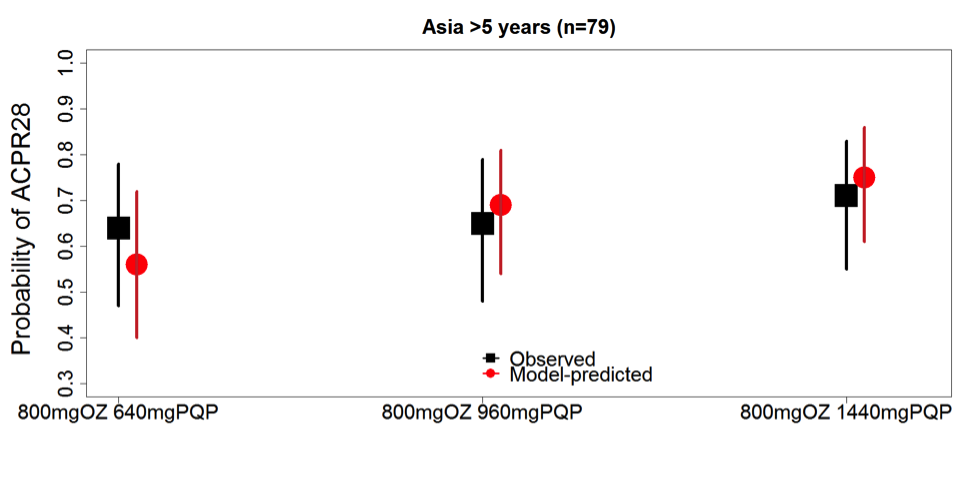
**

**Simulation method**

Two thousand patients per simulated treatment arm were sampled from the relevant study population, while maintaining the covariate structure within each sampled patient (e.g. body weight, age, baseline parasitemia). A set of population PK parameters and exposure-response parameters was sampled from their estimated uncertainty distributions. That is, each simulated replicate used a set of model parameters drawn from the multivariate normal distribution based on the estimated variance-covariance matrix (separately for each popPK and exposure-response model). Then, for each patient, a Cday7 for artefenomel and piperaquine and subsequently, a probability of ACPR28 followed by treatment outcome (success or failure) was simulated, using the developed models, the set of sampled parameters, each patient’s covariates, and the artefenomel and PQP doses. The treatment outcomes for the 2000 patients were then summarised by treatment (number of successes/2000) to generate an estimate of %ACPR28. These steps were repeated 750 times (replicates), each time sampling a new set of patients and parameters. For each simulated treatment arm the ACPR28 across replicates was summarised to generate a point estimate and 90% Confidence Interval (median, 5^th^ and 95^th^ percentile of the distribution across replicates).
